# Lipophilic mono-guanidines *versus* mono-(thio)ureas: anion transport, lipid binding, and antibacterial activity

**DOI:** 10.1039/d6ra04370d

**Published:** 2026-07-06

**Authors:** Randima D. De Silva Weerakonda Arachchige, Sarah R. Marshall, Hassan Gneid, Nathalie Busschaert

**Affiliations:** a Department of Chemistry, Tulane University New Orleans LA 70118 USA nbusschaert@tulane.edu

## Abstract

Synthetic anion transporters (anionophores) can collapse ion gradients and have been widely explored as candidate therapeutics for channelopathies and cancer, yet their potential as antibacterial agents remains comparatively underdevelopped. Here, we systematically compare lipophilic mono-guanidines with isosteric mono-ureas and mono-thioureas, evaluating anion binding, membrane transport, lipid association and antibacterial activity. Chloride transport experiments in POPC show that CF_3_-substituted guanidines generally outperform unfluorinated analogues, but remain less potent than their CF_3_-substituted urea and thiourea counterparts. Vesicle-based mechanistic studies suggest that the guanidines function as mobile carriers and can facilitate both electroneutral and electrogenic chloride transport. Interestingly, guanidine-mediated chloride transport increases with increasing pH, suggesting that transport is dominated by the neutral guanidine form and not the protonated guanidinium form—presumably due to the high polarity and limited membrane partitioning of the cationic guanidiniums. In addition, all compounds preferentially partition into anionic POPG membranes that mimic bacterial membranes. Several compounds therefore also display antibacterial activity against a variety of Gram-positive bacteria, with minimum inhibitory concentrations in the nanomolar range for the most potent compound and no measurable hemolytic activity. Bacterial mechanistic studies confirmed that the antibacterial activity primarily arises from ion-transport-driven membrane depolarization rather than nonspecific membrane lysis.

## Abstract 2

Guanidines and ureas are shown to possess anion transport, lipid binding and antibacterial activity. The ureas are superior due to the unusual pH dependence of the guanidines. Created in BioRender. Busschaert N. (2026) https://BioRender.com/3vm5vxp.

## Graphical abstract

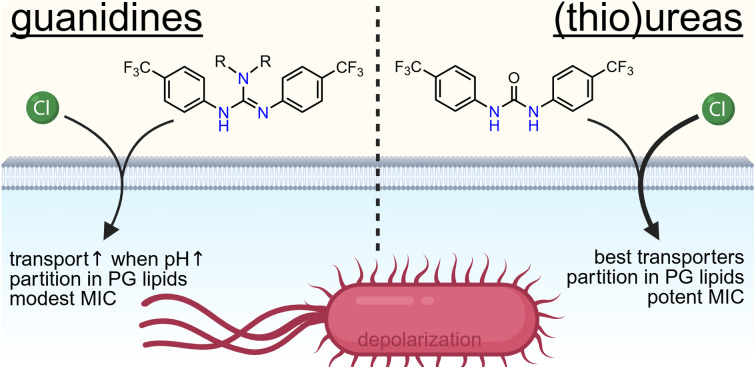

## Introduction

The development of synthetic anion transporters (anionophores) has emerged as a major focus within supramolecular chemistry,^1,2^ driven by their broad potential in medicine and other fields.^3,4^ To date, emphasis has been on the design of synthetic chloride transporters aimed at the treatment of channelopathies, such as cystic fibrosis,^5–8^ or as agents that induce apoptosis in cancer cells through disruption of ion homeostasis.^9–13^ By contrast, the use of synthetic anionophores as antibacterial agents remains comparatively underexplored.^14^ Because ion gradients are essential for bacterial viability,^15^ anion transport by synthetic molecules is expected to disturb ion homeostasis and lead to cell death. Importantly, selectivity for transport in bacterial cells over human cells could arise from the different lipid composition,^16,17^ larger negative membrane potential,^18,19^ and smaller size^20^ of bacteria compared to human cells. Moreover, compounds that target ion homeostasis are less prone to resistance development than agents that act on specific intracellular pathways, making them particularly attractive in the context of growing global antimicrobial resistance.^21^

So far, synthetic anionophores that have been shown to possess antibacterial activity have been based on (benz)imidazoles,^22–24^ protonatable amines,^25–27^ diamidocarbazoles,^28^ pyrroles,^29,30^ melamine,^31,32^ (thio)ureas^33–35^ and squaramides.^36,37^ Surprisingly, however, anionophores based on guanidines as the primary anion binding motif have not yet been explored in this context. This is somewhat surprising, given that guanidine groups typically display high p*K*_a_ values (≈9–13)^38–40^ and therefore exist predominantly as the protonated guanidinium cations at physiological pH. Guanidinium moieties are well known to promote strong interactions with anionic lipid headgroups, which are abundant in bacterial membranes but are largely absent from mammalian membranes which are dominated by zwitterionic and neutral lipids.^16,17^ As a result, many membrane-active antibacterial agents exploit guanidinium–lipid interactions to achieve selectivity for bacterial membranes – most notably the arginine-rich antimicrobial peptides such as indolicidin and tritrpticin.^41,42^

We have recently shown that selective lipid headgroup binding can be combined with anion transport activity,^43^ and therefore considered that guanidinium-based compounds might be well suited to combine selectivity for anionic membranes with anionophore activity. However, the anion transport activity of guanidines/guanidiniums has not been widely explored and remains poorly understood.^44,45^ In this work, we therefore present a systematic comparison of the anion transport efficiency, lipid binding affinity, and antibacterial activity of a series of lipophilic mono-guanidines and their well-established urea and thiourea isosteres. While several of the guanidine derivatives exhibited measurable anionophore and antibacterial activity, they were generally outperformed by the corresponding (thio)urea. Strikingly, however, we found that electrogenic anion transport by these systems is predominantly mediated by the neutral guanidine rather than the protonated guanidinium species. This unexpected result has important implications for the future design of guanidine-based anion transporters with biological activity, indicating that a potent guanidine anionophore should combine a low p*K*_a_ with sufficient anion affinity and lipophilicity in the neutral state.

## Results and discussion

### Design and synthesis

**Scheme 1:**
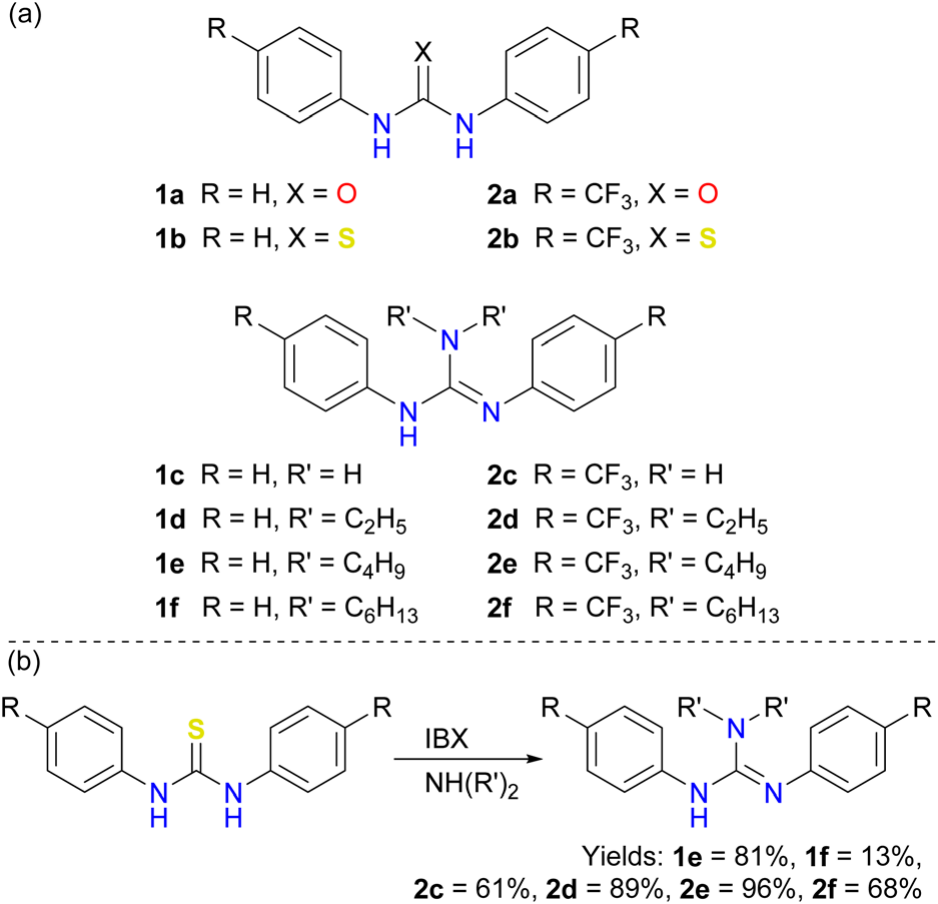
(a) Structure of the (thio)urea and guanidine compounds studied in this manuscript. (b) General method for the synthesis of guanidine derivatives with the resulting yield (IBX = 2-iodoxybenzoic acid).

To date, there have been only two reports of guanidinium-based compounds with anion transport ability: a guanidinium-appended calixarene by Tecilla and co-workers, and a series of tetradecylguanidinium isomers by Fyles and colleagues.^44,45^ In addition, the (semi)aza-bambusuril anionophores reported by Reany and co-workers are also technically guanidiniums.^46^ However, these studies focus on the presumed OH^−^/Cl^−^ antiport of the guanidinium (Cl^−^ transport combined with protonation and deprotonation of the guanidinium transporter), and do not provide detailed transport mechanisms, antibacterial activity or a comparison with established anion transporters. Because guanidinium groups can be viewed as cationic isosteres of urea and thiourea functionalities—motifs that are among the most frequently used hydrogen bond donors in anion transporter design—we sought to directly compare the properties of guanidines with those of their (thio)urea counterparts. As reference compounds, we selected mono-ureas 1a and 2a and mono-thioureas 1b and 2b (Scheme 1a). These compounds are easy to make, well-characterized and known to exhibit potent anion transport properties^47–49^ as well as antibacterial activity,^50,51^ even though a direct link between anion transport and antibacterial activity has not yet been firmly established. In contrast to these neutral (thio)urea reference compounds, guanidines are expected to exist predominantly in their protonated guanidinium form at physiological pH, rendering them significantly more polar. This increased polarity could impede membrane partitioning and, consequently, reduce anion transport efficiency. We therefore synthesized a small series of guanidine derivatives 1c–1f and 2c–2f bearing alkyl chains of varying chain length to systematically tune their lipophilicity (Scheme 1a). All guanidines were synthesized from the corresponding thiourea following the procedure described by Akamanchi *et al.*,^52^ as shown in Scheme 1b (full synthetic details and characterization are provided in the SI).

Two parallel series of compounds were prepared that differ in the presence or absence of a CF_3_ substituent on the aromatic ring (Scheme 1a). CF_3_ groups are frequently incorporated into anion transporters because they increase lipophilicity and enhance anion binding.^53^ At the same time, however, the addition of an electron-withdrawing CF_3_ group is also expected to lower the p*K*_a_ of the guanidinium group, thereby increasing the fraction of neutral guanidine that is present at physiological pH. This leads to two presumably competing effects: the neutral guanidines are more lipophilic and are expected to partition in the membrane better, while the cationic guanidiniums are expected to show stronger anion binding. To assess how these factors influence anion transport, the p*K*_a_ values for 1c–1f and 2c–2f were determined using UV-vis experiments in 1 : 1 MeCN : buffer solutions and extrapolated to pure water (see SI for details). The obtained values are given in Table 1, along with calculated log *D* values (pH 7.4) used to estimate lipophilicity. As expected, the p*K*_a_ value for the unfluorinated derivatives 1c–1f is approximately 10,^38–40^ whereas that of the fluorinated derivatives 2c–2f is significantly reduced to approximately 8. Furthermore, it has been suggested that the p*K*_a_ of guanidinium groups may be significantly lower in membrane environments compared to aqueous solutions,^54,55^ suggesting that a significant fraction of the compounds (especially the compounds containing a CF_3_ group) will be present in the membrane as the neutral guanidine.

**Table 1:** Overview of the physicochemical properties (p*K*_a_ and log *D*), and anion binding (*K*_a,Cl_), anion transport (EC50 and *n*), lipid binding (*K*_SV,PC_ and *K*_SV,PG_), and antibacterial (MIC) activity of compounds 1a–2f

Compound	p*K*_a_^a^	log *D*^b^ (pH 7.4)	*K*_a,Cl_ c (M^−1^)	EC_50_^d^ (mol%)	*n* e	*K*_SV,PC_ f (M^−1^)	*K*_SV,PG_ g (M^−1^)	MIC^h^ (*S. aureus*) (µM)
1a	n.d.^i^	3.12	—	>5	—	(10.8 ± 0.6) 10^3^	(19.8 ± 5.9) 10^3^	>128
1b	n.d.^i^	4.01	—	>5	—	(1.2 ± 0.1) 10^3^	(13.5 ± 2.4) 10^3^	>128
1c	9.96 ± 0.08	1.27	245	>5	—	(1.8 ± 0.9) 10^3^	(13.9 ± 1.0) 10^3^	>128
1d	10.17 ± 0.08	2.47	<5	>5	—	<1000	(2.8 ± 0.7) 10^3^	>128
1e	10.19 ± 0.08	4.41	<5	>5	—	(3.0 ± 0.3) 10^3^	(9.6 ± 1.7) 10^3^	>128
1f	9.49 ± 0.09	6.19	<5	4.3 ± 0.3	3.4 ± 0.6	(16.2 ± 1.6) 10^3^	(53.5 ± 8.7) 10^3^	32
2a	n.d.^i^	4.87	—	0.42^j^	2.2^j^	(56.5 ± 11.1) 10^3^	(70.0 ± 3.4) 10^3^	0.25–0.5
2b	n.d.^i^	5.76	—	0.22^j^	1.6^j^	(41.1 ± 2.2) 10^3^	(61.4 ± 3.4) 10^3^	1–2
2c	7.88 ± 0.09	3.85	1005	4.4 ± 0.1	1.8 ± 0.1	(26.7 ± 0.8) 10^3^	(60.8 ± 4.8) 10^3^	32–64
2d	8.00 ± 0.15	4.34	33	>5	—	(27.8 ± 1.0) 10^3^	(44.2 ± 1.5) 10^3^	> 128
2e	7.84 ± 0.15	6.23	24	3.8 ± 0.3	2.1 ± 0.3	(48.4 ± 2.3) 10^3^	(83.5 ± 8.3) 10^3^	n.d.^k^
2f	7.84 ± 0.10	8.01	31	1.2 ± 0.1	1.9 ± 0.2	(58.9 ± 16.4) 10^3^	(108.3 ± 12.4) 10^3^	n.d.^k^

a p*K*_a_ measured by UV-vis titrations in 1 : 1 MeCN : buffer and extrapolated to pure water.

b pH-dependent octanol–water partitioning constant at pH 7.4 calculated using Chemicalize.

c Association constant of the neutral guanidines with TBACl measured using ^1^H NMR titrations in CD_3_CN at 298 K.

d Concentration of transporter needed to achieve 50% chloride influx in 300 s.

e Hill coefficient.

f Stern–Volmer constant representing POPC binding measured using the NBD quenching assay.

g Stern–Volmer constant representing POPG binding measured using the NBD quenching assay.

h Minimal inhibitory concentration against *S. aureus* measured using broth microdilution methods.

i p*K*_a_ could not be determined experimentally.

j Previously published data (ref. 47).

k MIC could not be determined because the compounds precipitated under the experimental conditions.

### Anion binding in solution and solid state

Before evaluating anion transport, we first examined the anion binding properties of the guanidine and guanidinium compounds in solution and in the solid state. In solution, chloride association constants for the neutral guanidines 1c–1f and 2c–2f were determined by ^1^H NMR titrations in CD_3_CN with tetrabutylammonium chloride (TBACl) as the chloride source. The obtained *K*_a_ values for 1 : 1 binding are given in Table 1. The alkyl-substituted guanidine derivatives 1d–1f and 2d–2f possess only a single NH hydrogen bond donor in their neutral form and consequently exhibit very weak chloride binding. Association constants of <5 M^−1^ were obtained for the unfluorinated compounds 1d–1f, while slightly higher values of 24–33 M^−1^ were measured for the corresponding CF_3_-substituted compounds 2d–2f. In contrast, the unsubstituted guanidine derivatives 1c and 2c, which retain three NH hydrogen bond donors in their neutral form, display significantly stronger chloride affinities under identical conditions, with *K*_a_ values of 245 M^−1^ for 1c and 1005 M^−1^ for 2c.

We next sought to investigate the anion binding ability of the protonated guanidiniums. Because this inherently involves a competitive binding scenario between chloride and the counteranion of the acid used for protonation, a weakly coordinating anion was required. Protonation was therefore achieved using HPF_6_. In most cases, ^1^H NMR titrations conducted in the presence of HPF_6_ revealed a pronounced downfield shift of the guanidinium NH signals upon the addition of <1 equivalent TBACl, while the addition of further equivalents of TBACl did not produce any further changes (see SI)—indicating strong binding. However, the resulting data could not be satisfactorily fitted to simple 1 : 1, 1 : 2 or 2 : 1 binding models. The only exception was 2d, which showed *K*_a_ = 702 M^−1^ for 1 : 1 binding to Cl^−^ in the presence of HPF_6_ (see SI). This is more than 20× higher than the binding constant in the absence of HPF_6_ (*K*_a_ = 33 M^−1^, Table 1), confirming that protonation substantially enhances anion affinity.

**Fig. 1:**
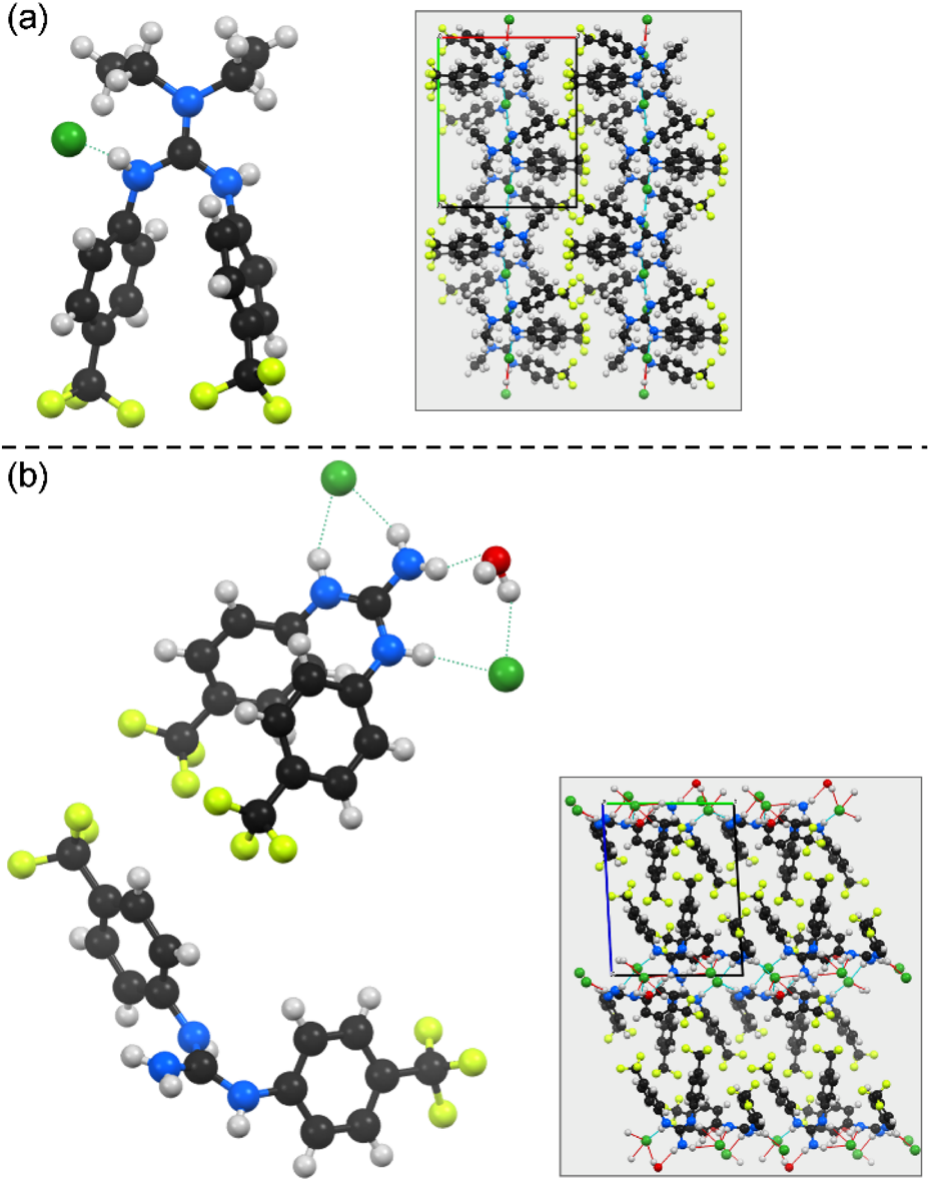
Single crystal X-ray diffraction structures of the HCl salts of 2d and 2c. Color scheme is as follows: H = light grey, C = black, N = blue, O = red, F = yellow, Cl = green. Dotted turquoise lines represent hydrogen bonds. Structures are shown as their asymmetric unit and the light grey insets show crystal packing. (a) Structure of 2d·HCl. (b) Structure of 2c·HCl.

To gain further insight into chloride binding by the cationic guanidiums, single crystals suitable for X-ray diffraction were obtained for 2c·HCl and 2d·HCl by slow evaporation of methanolic solutions containing a small amount of aqueous HCl (CCDC 2542395 and 2542396). Interestingly, 2d·HCl adopts an *anti*–*anti* conformation in the solid state (with the two NHs pointing away from each other) (Fig. 1a), while 2c·HCl crystallizes as a mixture of *syn*–*anti* and *anti*–*anti* conformers (Fig. 1b). Guanidiniums are conformationally flexible and *syn*–*syn*, *syn*–*anti* and *anti*–*anti* conformations have all been previously observed in crystal structures of related diphenylguanidinium salts.^56–58^ This suggests that the conformations observed in the crystal structures of 2c and 2d are not necessarily the conformations adopted by these compounds in the membrane. In the crystal structure of 2d·HCl, each chloride anion participates in 2 hydrogen bonds with 2 different guanidinium units, while each guanidinium engages in hydrogen bonding with 2 different chloride anions. These interactions give rise to an extended linear hydrogen-bonded network whereby the various 1D chains are packed together in a complex 3D network *via* weaker interactions (Fig. 1a, inset). An even more complex arrangement is observed for 2c·HCl: each chloride anion is coordinated by 4 or 5 hydrogen bonds originating from 3 different guanidinium molecules and 1 bridging water molecule, whereas each guanidinium molecule interacts with 2 or 4 chloride anions, within 2D sheets held together *via* weaker interactions (Fig. 1b, inset). These solid-state structures illustrate the strong anion-binding potential of guanidinium moieties in their protonated form, albeit in highly cooperative, multi-component hydrogen-bonding networks.

### Anion transport

#### Estimate of chloride transport ability

**Fig. 2:**
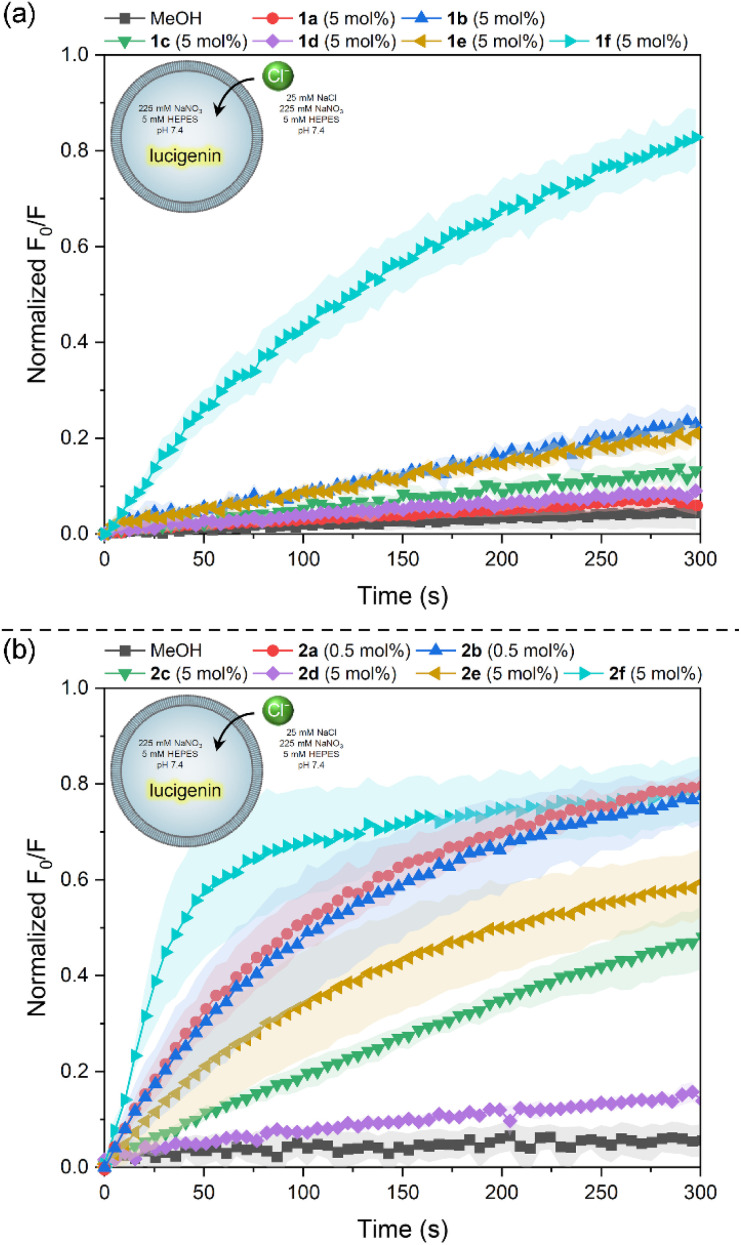
Chloride transport facilitated by the transporters. 100 nm POPC LUVs containing 1 mM lucigenin and 225 mM buffered NaNO_3_ (5 mM HEPES, pH 7.4) were suspended in 225 mM buffered NaNO_3_ (5 mM HEPES, pH 7.4). Transport was initiated by the addition of 25 mM NaCl and the compounds at 5 mol% with respect to lipid (0.5 mol% was used for compounds 2a and 2b due to their high activity). After 300 s, Triton X-100 was added to lyse the LUVs and normalize the data. Graphs represent the average of a minimum of three independent repeats. Symbols show 1 in every 50 data points, with the shaded area representing the standard deviation. MeOH was used as a negative control. (a) Unfluorinated compounds 1a–1f. (b) Fluorinated compounds 2a–2f.

Anion transport by the compounds was evaluated in 100 nm 1-palmitoyl-2-oleoyl-*glycero*-3-phosphocholine (POPC) large unilamellar vesicles (LUVs) using the standard lucigenin assay.^59^ Briefly, vesicles encapsulating lucigenin in HEPES buffer (1 mM lucigenin, 225 mM NaNO_3_, 5 mM HEPES, pH 7.4) were suspended in a lucigenin-free external buffer solution (225 mM NaNO_3_, 5 mM HEPES, pH 7.4). Chloride transport was initiated by the addition of NaCl (25 mM) and transporter (5 mol% with respect to lipid), and chloride influx was quantified by monitoring the decrease in lucigenin fluorescence over time. Initial pre-incorporation studies revealed that compound 2f suffers from reduced membrane deliverability when added externally, resulting in significantly diminished transport activity. Fortunately, this limitation could be overcome by increasing the amount of organic solvent used during external addition to 5% v/v relative to the total assay volume (see SI, Fig. S53). All subsequent transport experiments involving 2f were therefore conducted under these optimized conditions. The results of the lucigenin assay for all compounds are shown in Fig. 2.

Fig. 2 reveals that the fluorinated guanidines consistently outperform their unfluorinated analogues, indicating that lowering the p*K*_a_ of the compounds and thereby increasing the fraction of neutral guanidine is beneficial for transport—presumably because the neutral guanidines are more lipophilic. Within the unfluorinated series, transport activity increases systematically with alkyl chain length, following the order 1f > 1e > 1d ≈ 1c (Fig. 2a), consistent with the notion that increasing lipophilicity increases transport activity. In contrast, transport efficiency within the fluorinated guanidine series does not correlate directly with lipophilicity: 2f > 2e > 2c > 2d (Fig. 2b). Notably, compound 2c exhibits higher activity than the more lipophilic 2d, despite its higher polarity. We hypothesize that the significantly stronger chloride binding ability of 2c (Table 1)—arising from its higher number of hydrogen bond donors—partially compensates for its reduced lipophilicity.

These qualitative trends were further confirmed by dose-response measurements, which provide EC_50_ values (defined as the transporter concentration required to achieve 50% chloride efflux within 300 s) and Hill coefficients *n* (Table 1).^60^ The EC_50_ values largely mirror the trends observed in Fig. 2, but facilitate direct comparison with the (thio)urea isosteres. Although unfluorinated guanidine 1f is a better transporter than isosteric urea 1a and thiourea 1b, none of the fluorinated guanidines surpass the transport activity of fluorinated urea 2a and thiourea 2b. This suggests that the current set of guanidines are not highly active transporters yet, but there is potential to optimize them further to outcompete potent (thio)urea-based transporters. Interestingly, all guanidines display Hill coefficients significantly greater than 1, indicating cooperativity or higher stoichiometry. Consistent with this interpretation, the initial rate of transport scales linearly with the square of the transporter concentration (see SI, Fig. S55–S58), supporting a 2 : 1 transporter : chloride stoichiometry for the transporting species. The only exception is 2c, which shows a linear relationship of the initial transport rate with the square root of the transporter concentration, suggesting a 1 : 2 transporter : chloride stoichiometry (see SI, Fig. S56).^61^ This observation is unexpected, as one might anticipate a neutral 1 : 1 guanidinium : chloride complex to be responsible for transport. Instead, this data hints that chloride transport might be predominantly mediated by the neutral guanidine form rather than the cationic guanidinium.

#### Mechanism of chloride transport

An alternative explanation for the large Hill coefficients observed in the lucigenin assays is that the guanidines self-assemble into ion channels rather than act as mobile carriers. To probe this possibility, chloride transport was examined in dipalmitoylphosphatidylcholine (DPPC) vesicles above and below the lipid phase transition temperature (*T*_m_ = 41 °C). Below *T*_m_ (25 °C), the guanidines induce only minimal chloride transport, whereas a pronounced increase in transport activity is observed above *T*_m_ (45 °C) (see SI, Fig. S71–S82). This strong dependence on membrane fluidity is characteristic of a mobile carrier mechanism and thus rules out ion channel formation.

The chloride transport observed in the lucigenin assays thus far could, in principle, be the result of either M^+^/Cl^−^ symport or Cl^−^/NO_3_^−^ antiport, and either process could be electrogenic or electroneutral. Variants of the lucigenin assay in which the NaCl pulse was replaced with KCl, RbCl or CsCl, did not show any change in Cl^−^ transport, ruling out M^+^/Cl^−^ symport (see SI, Fig. S59–S70). In contrast, replacing NaCl with NaBr or NaI, resulted in a marked acceleration of anion transport, consistent with a halide/nitrate exchange mechanism (see SI, Fig. S59–S70). However, this apparent Cl^−^/NO_3_^−^ antiport could arise from several mechanistic scenarios: (i) true electroneutral Cl^−^/NO_3_^−^ antiport (where Cl^−^ and NO_3_^−^ transport cannot be separated), (ii) electrogenic Cl^−^ transport in one direction combined with electrogenic NO_3_^−^ transport in the other direction, or (iii) electroneutral Cl^−^/H^+^ transport in one direction combined with electroneutral NO_3_^−^/H^+^ in the other direction.

**Fig. 3:**
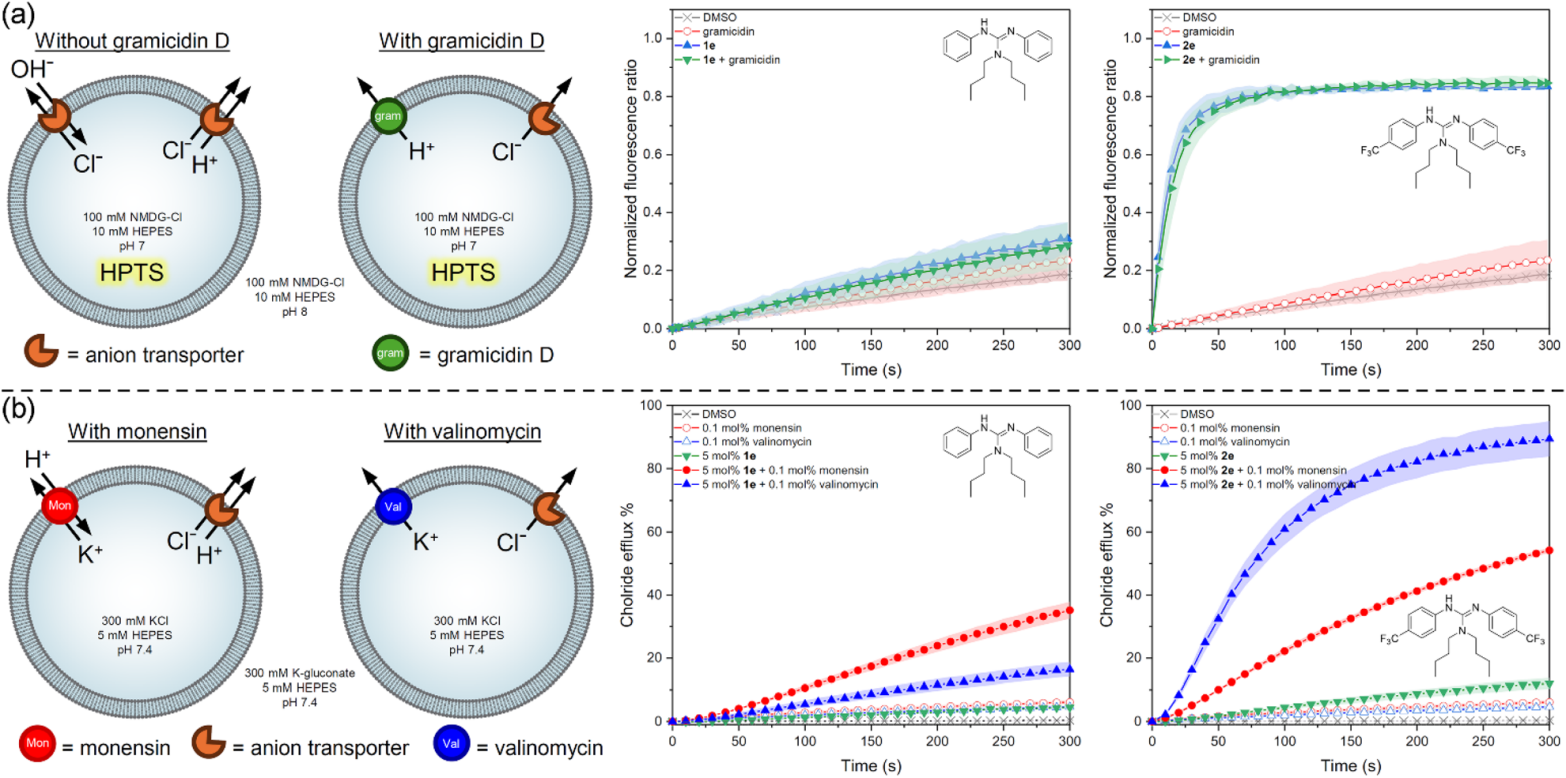
(a) Schematic representation of the HPTS assay illustrating proton transport in the presence and absence of gramicidin D, alongside the results of compound 1e (representing the behavior of the non-fluorinated compounds) and compound 2e (representing the behavior of the fluorinated compounds). (b) Schematic representation of electrogenic and electroneutral transport processes in POPC liposomes in the presence of the cationophores valinomycin and monensin, alongside the results for 1e and 2e. Results were obtained as described in the SI and are the average of minimum 3 trials, with shaded areas representing standard deviations.

Given that guanidiniums have previously been suggested as Cl^−^/OH^−^ antiporters (or equivalent Cl^−^/H^+^ symporters),^44,45^ we first investigated the ability of the guanidines to transport H^+^ (or OH^−^) using the 8-hydroxypyrene-1,3,6-trisulfonic acid (HPTS) assay under conditions that suppress fatty-acid-mediated proton transport.^62^ In this assay, LUVs encapsulating HPTS report on internal pH changes following the application of a base pulse to create a transmembrane pH gradient. Dissipation of this gradient upon addition of transporter indicates H^+^ (or OH^−^) transport. Representative results for 1e and 2e are shown in Fig. 3a, while data for all other compounds are provided in the SI (Fig. S83–S94). Among the unfluorinated guanidines, only 1f exhibited significant activity in the HPTS assay, whereas all fluorinated guanidines (except 2d) were active. This trend closely mirrors the results for the lucigenin assay and confirms that many of the guanidines can mediate both Cl^−^ and H^+^ (or OH^−^) transport. Furthermore, the rate of pH gradient dissipation induced by the guanidines is unaffected by the presence of Gramicidin D, a highly efficient proton channel, indicating that H^+^ transport is not the rate limiting step in the HPTS assay (see Fig. 3a).^63^

**Fig. 4:**
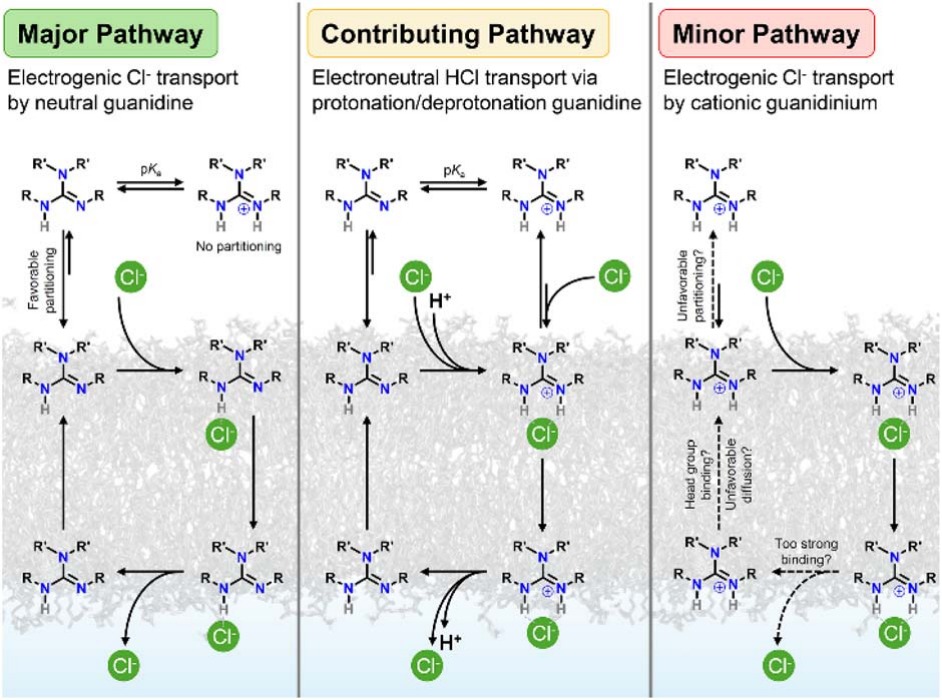
Proposed mechanism for chloride transport by guanidine-based anionophores. Two major pathways are proposed: electrogenic Cl^−^ transport mediated by the neutral guanidine (left) or electroneutral HCl cotransport (middle). The relative contribution of these two pathways depends on the p*K*_a_ of the transporter, with the fluorinated guanidiniums (low p*K*_a_) preferring electrogenic transport and the unfluorinated guanidiniums (high p*K*_a_) preferring electroneutral transport. A third, likely minor, pathway involves electrogenic Cl^−^ transport facilitated by cationic guanidiniums (right). Its limited contribution is presumably due to unfavorable partitioning of the polar guanidinium species, overly strong chloride binding, or hindered diffusion within the membrane due to strong interactions with the lipid headgroups. These unfavorable steps for the cationic guanidiniums are indicated by dashed arrows.

Question remains whether the apparent Cl^−^/NO_3_^−^ antiport observed in the lucigenin assay and the apparent Cl^−^/H^+^ symport observed in the HPTS assay are due to electroneutral or electrogenic transport events. To clarify this point, we employed the cationophore-coupled transport assay first developed by Wu *et al.*^63^ In this assay, LUVs containing buffered KCl are suspended in external potassium gluconate buffer, with gluconate serving as a highly polar, non-transportable anion to exclude Cl^−^/gluconate antiport. Under these conditions, chloride efflux can only occur *via* K^+^/Cl^−^ symport, and thus requires the combined action of an anion transporter and a cationophore. Two different cationophores were used: valinomycin, an electrogenic K^+^ carrier, and monensin, an electroneutral K^+^/H^+^ antiporter. Observations of chloride efflux (measured using an ion selective electrode) in the presence of valinomycin indicate electrogenic Cl^−^ transport, whereas chloride efflux in the presence of monensin indicates electroneutral Cl^−^/H^+^ symport. Representative results for 1e and 2e are shown in Fig. 3b, while the results for the other compounds are shown in the SI (Fig. S95–S106). All active guanidines facilitated chloride efflux in the presence of both valinomycin and monensin, demonstrating that they can mediate both electrogenic and electroneutral transport processes. Notably, distinct trends emerged between the two compound series. For the unfluorinated compounds, chloride efflux was more efficient in the presence of monensin than valinomycin, indicating a preference for electroneutral Cl^−^/H^+^ transport. In contrast, the fluorinated compounds showed enhanced chloride transport in the presence of valinomycin, consistent with a preference for electrogenic Cl^−^ transport. This notable difference can be rationalized based on the difference in p*K*_a_ between the two series (Table 1). The unfluorinated guanidines have a p*K*_a_ ∼10 in water, but this p*K*_a_ is expected to be lower in the membrane. These compounds are therefore expected to undergo protonation/deprotonation events as they partition into the membrane, accounting for the observed Cl^−^/H^+^ transport. On the other hand, the p*K*_a_ of the fluorinated compounds is substantially lower (p*K*_a_ ∼8), resulting in a larger fraction of neutral guanidine present at physiological pH and eliminating the need for deprotonation to access the membrane interior. Furthermore, given that electrogenic Cl^−^ transport is favored by the compounds with the lowest p*K*_a_, provides evidence that neutral guanidines, rather than cationic guanidiniums, are the primary transporting species. An overview of the proposed transport mechanism is given in Fig. 4.

#### pH-dependent chloride transport

**Fig. 5:**
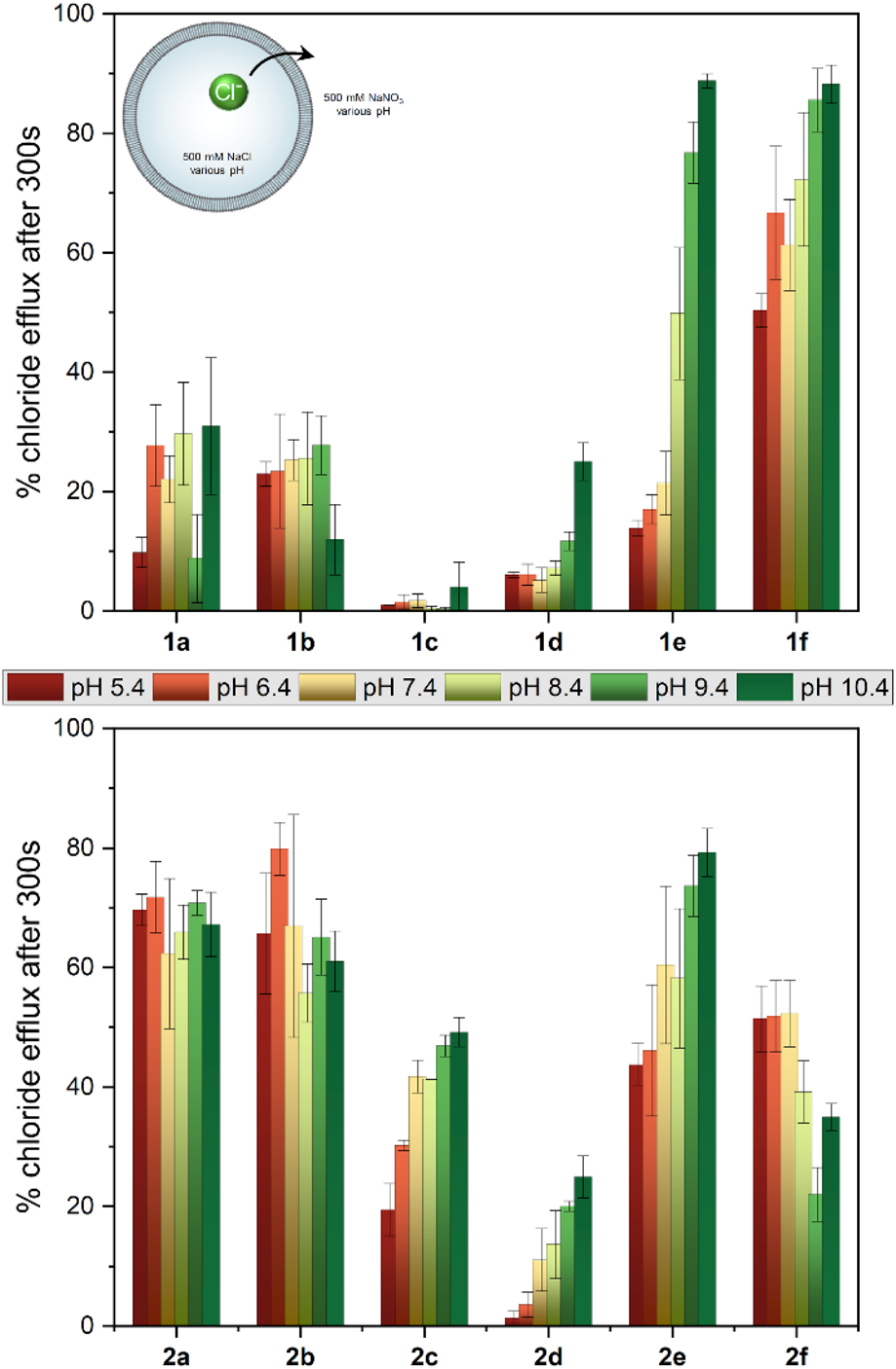
pH-dependent chloride efflux measured 300 s after the addition of transporter using an ion selective electrode. Experiments were performed as described in the SI and are the average of minimum 3 independent repeats with error bars representing standard deviations. All compounds were added at a concentration of 5 mol% with respect to lipid, except 2a and 2b, which were added at 0.5 mol%, and 2f, which was added at 1 mol%.

The large Hill coefficients observed for the guanidine series, together with the preference for electrogenic transport by the derivatives with the lowest p*K*_a_, suggest that anion transport is mediated predominantly by the neutral guanidine rather than the cationic guanidinium. If this hypothesis is correct, transport efficiency should increase as the fraction of neutral guanidine increases. We therefore examined pH-dependent chloride transport using the standard Cl^−^/NO_3_^−^ assay (where chloride efflux is measured using an ion selective electrode)^64^ across a pH range of 5.4 to 10.4 (Fig. 5). As expected, Fig. 5 shows that ureas 1a and 2a, as well as thioureas 2a and 2b, do not exhibit a clear pH dependence within experimental error. In contrast, most guanidines show a marked pH dependence where chloride transport increases with increasing pH, providing strong evidence that the neutral guanidine is the more effective transporting form. This effect is most pronounced for compounds 1d, 1e and 2d, which show almost no measurable chloride transport activity at pH 5.4, but become efficient chloride transporters at pH 10.4. For 1f, 2c and 2e, appreciable transport is already evident at pH 5.4, yet transport rates still increase substantially at higher pH. The residual transport activity observed at low pH could arise from (i) a small but non-zero amount of neutral guanidine remaining even under acidic conditions—especially in the membrane where p*K*_a_ values may be lower, and/or (ii) some transport competence of the cationic guanidinium, albeit less efficiently than the neutral form. Regardless of the origin of the transport activity at low pH, the overall increase in transport activity with increasing pH strongly supports the conclusion that the neutral guanidine form dominates anion transport. This assignment also helps rationalize the otherwise counterintuitive observation that 2c outperforms 2d despite being more polar. Most guanidines in this manuscript (1d–1f, and 2d–2f) possess only one hydrogen bond donor in their neutral form and accordingly bind chloride only weakly. In contrast, 2c retains 3 hydrogen bond donors in its neutral form and exhibits much stronger chloride affinity, which appears to be sufficient to partially offset its reduced lipophilicity and still enable efficient transport. Collectively, these data imply that potent guanidine-based anion transporters will require a carefully balanced combination of (i) sufficient lipophilicity for membrane partitioning, (ii) a low enough p*K*_a_ to ensure a significant neutral fraction at physiological pH, and (iii) multiple hydrogen bond donors in the neutral form (or otherwise enhanced anion binding).

Only one compound deviates from the general pH trend: 2f shows a slight decrease in chloride transport with increasing pH (Fig. 5). Notably, 2f is also the compound that exhibited deliverability limitations in the initial lucigenin experiments due to its high lipophilicity. When the pH-dependent transport experiments were conducted under standard conditions (without the increased fraction of organic solvent to mitigate precipitation/aggregation), the inverse pH dependence became even more pronounced, with high transport activity observed at pH 5.4 and essentially no chloride transport observed at pH 10.4 (see SI, Fig. S119). This suggests that for 2f the neutral guanidine form may be too lipophilic and causes deliverability problems (*e.g.*, precipitating in solution), whereas the cationic guanidinium form is more soluble/deliverable yet sufficiently lipophilic to partition into the membrane and mediate transport.

Several factors could contribute to the superior performance of the neutral guanidines relative to the guanidinium cations, as indicated in Fig. 4. One possibility is that the cationic guanidiniums bind Cl^−^ too strongly and thereby cannot release the anion on the other side of the membrane, thus impeding transport. While protonation clearly increases chloride affinity (as shown by the ^1^H NMR titrations discussed above), the measured chloride binding constant for protonated 2d (*K*_a_ = 702 M^−1^) is comparable to that of neutral 2c (*K*_a_ = 1005 M^−1^), making it less likely that strong anion binding can account for the reduced transport ability of cationic guanidinium species. A more compelling explanation is that the cationic guanidiniums are too polar and have insufficient membrane partitioning ability. This hypothesis is corroborated by the fact that the more lipophilic fluorinated guanidines retain chloride transport ability at pH 5.4, and that the most lipophilic guanidine 2f displays an inverse pH dependence where more efficient transport is observed at lower pH. This suggests that cationic guanidiniums can become potent anion transporters provided that they are made lipophilic enough to mask the cationic charge (although this creates deliverability problems). Finally, an additional possibility is that the cationic guanidiniums bind strongly to the lipid phosphate headgroups, effectively trapping them at the membrane interface and limiting diffusion across the bilayer. Such interfacial sequestration would be expected to disfavor electrogenic transport in particular, akin to other systems where strong headgroup interactions immobilize the anion transporter (*e.g.*, prodigiosin or a tetra-urea previously reported by Gale and co-workers).^63,65^

#### Lipid binding and lipid-dependent transport

**Fig. 6:**
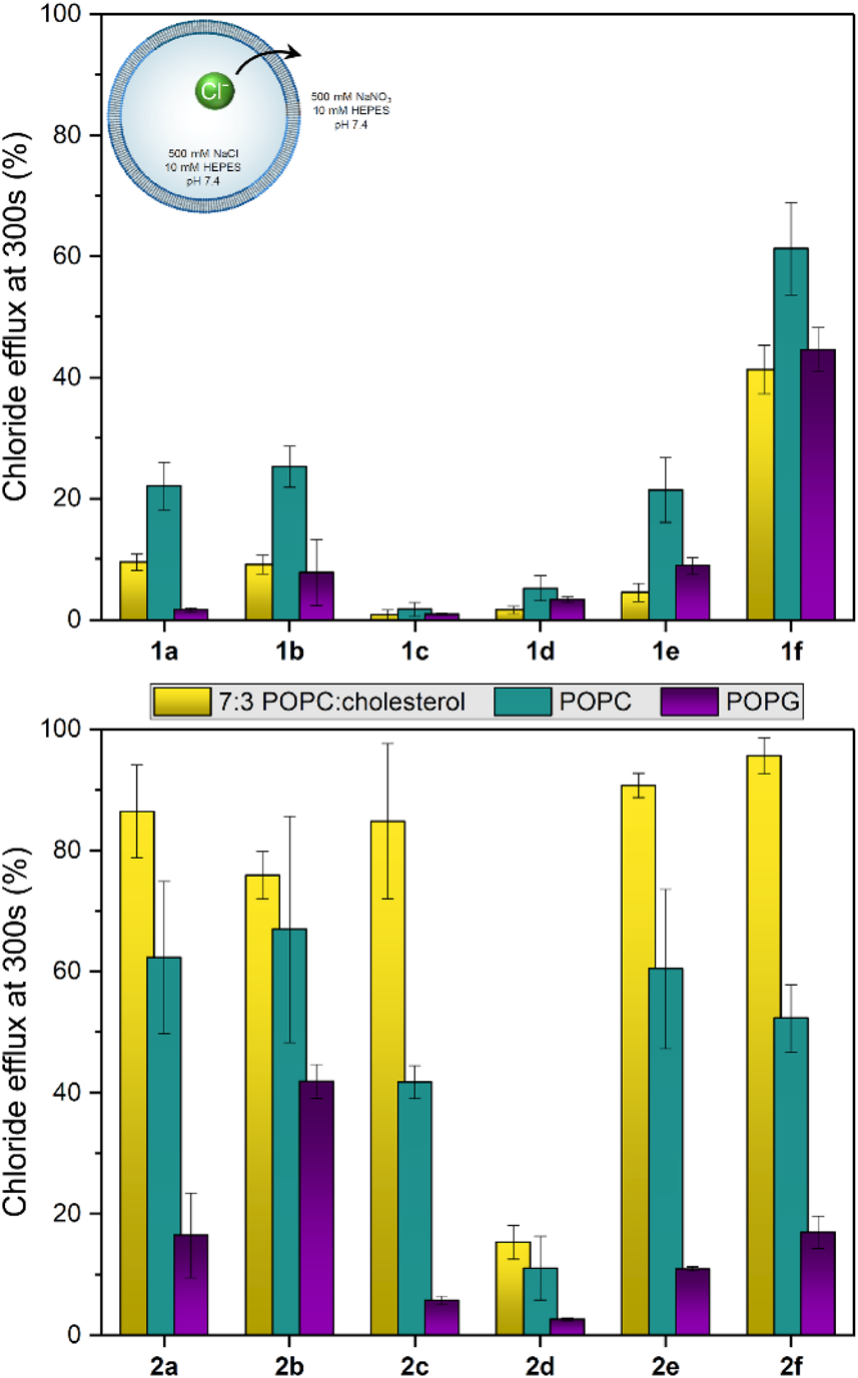
Chloride efflux from 200 nm LUVs with different lipid compositions (7 : 3 POPC : cholesterol, 100% POPC, or 100% POPC) measured 300 s after the addition of transporter using an ion selective electrode. Experiments were performed as described in the SI and are the average of minimum 3 independent repeats with error bars representing standard deviations. All compounds were added at a concentration of 5 mol% with respect to lipid, except 2a and 2b, which were added at 0.5 mol%, and 2f, which was added at 1 mol%.

To assess whether phospholipid headgroup interactions contribute to the unusual anion transport behavior of the guanidine derivatives, we examined Cl^−^/NO_3_^−^ antiport using vesicles of varied lipid composition, including zwitterionic POPC, POPC : cholesterol (7 : 3), and the anionic lipid POPG (1-palmitoyl-2-oleoyl-*sn-glycero*-3-phosphoglycerol). If headgroup binding were a primary determinant of transport ability, a pronounced dependence of lipid composition would be expected. Fig. 6 summarizes the lipid-dependent transport experiments at pH 7.4, while corresponding datasets at pH 5.4 and pH 10.4 are given in the SI (Fig. S124–S126).

Across all compound classes, chloride transport in POPG vesicles is less efficient than in POPC vesicles (Fig. 6). This decrease may reflect stronger interactions between the transporters and the anionic POPG headgroup, but may also arise from the intrinsic energetic penalty associated with moving anionic species across an anionic membrane. More diagnostic information emerges from the experiments in POPC : cholesterol (7 : 3). For the unfluorinated series, transport is generally more efficient in pure POPC than in POPC : cholesterol vesicles (Fig. 6), consistent with the expected reduction of mobile carrier transport upon membrane rigidification by cholesterol.^66^ In contrast, the fluorinated series exhibits the opposite trend: chloride transport is enhanced in 7 : 3 POPC : cholesterol relative to pure POPC (Fig. 6). This behavior is unusual for mobile carriers, particularly given that DPPC phase-transition experiments indicated carrier-type transport for these compounds (see above). A plausible explanation is competitive binding to POPC headgroups. If the fluorinated compounds associate strongly with the phosphocholine phosphate groups, the fraction of transporter available for productive chloride binding and transport would be reduced. Diluting POPC with cholesterol would then reduce headgroup competition and increase the effective population of transport-effective species, leading to enhanced transport in POPC : cholesterol membranes.

**Fig. 7:**
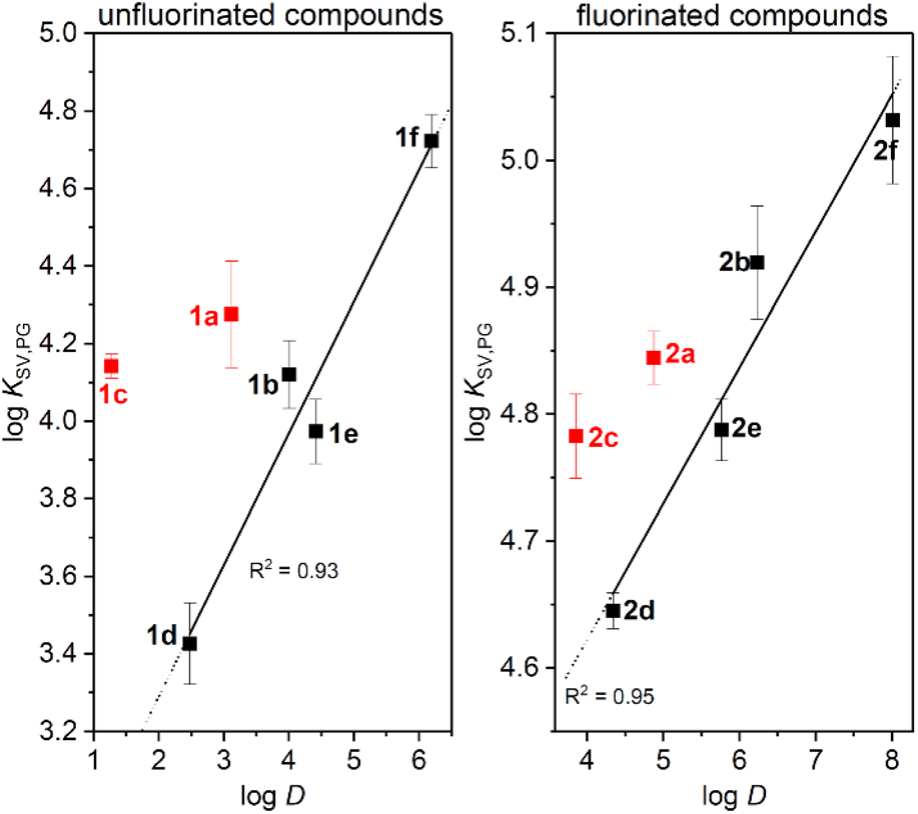
Logarithm of the Stern–Volmer constant for POPG binding (log *K*_SV,PG_) plotted against the calculated log *D* (pH 7.4) of the unfluorinated (left panel) and fluorinated (right panel) compounds. Stern–Volmer constants were determined as described in the SI and are the average of minimum 3 independent repeats with error bars representing standard deviations. Black lines represent linear fits, whereby the red data points were omitted from the fit.

To further probe membrane association and potential headgroup interactions, we employed our recently developed NBD (7-nitrobenz-2-oxa-1,3-diazole) quenching assay.^67^ In this assay, POPC vesicles containing 1 mol% NBD-labelled lipid (18 : 1–06 : 0 NBD-PC), or POPG vesicles containing 18 : 1–06 : 0 NBD-PG, are titrated with aliquots of the transporters and the quenching of the NBD fluorescence is monitored. The quenching follows a Stern–Volmer relationship and can be quantified by Stern–Volmer constants: *K*_SV,PC_ (to estimate binding to POPC) and *K*_SV,PG_ (to estimate binding to POPG) (Table 1). Provided that direct quenching of NBD by the transporters in bulk solution is minimal (see SI, Fig. S139), differences in *K*_SV_ primarily reflect differences in local transporter concentrations in the membrane, and therefore report in relative membrane partitioning or interfacial enrichment. For all compounds, *K*_SV,PG_ > *K*_SV,PC_, indicating greater partitioning in anionic POPG membranes than in zwitterionic POPC membranes. This trend supports the presence of favorable interactions with anionic headgroups and is consistent with some degree of headgroup association across the series. However, this behavior is not unique to the guanidines and is also observed for the (thio)urea derivatives. Thus, while headgroup binding is clearly present, there is no evidence from these data that the distinctive pH-dependence and reduced activity of the guanidines arises from uniquely strong lipid headgroup binding relative to (thio)ureas. Consistent with this interpretation, plotting the logarithm of *K*_SV,PG_ against calculated log *D* yields an approximate linear relationship (Fig. 7), suggesting that membrane partitioning is largely dominated by lipophilicity rather than specific headgroup recognition. The only exceptions are guanidines 1c and 2c (which have more hydrogen bond donors than the alkylated guanidines) and ureas 1a and 2a, all of which display larger *K*_SV,PG_ values than predicted by log *D* alone. This behavior is indicative of stronger interactions with the lipid headgroups. Notably, the same compounds also show more efficient chloride transport than expected from lipophilicity alone, suggesting that strong lipid headgroup binding is not necessarily detrimental for anion transport. Instead, in some cases lipid headgroup binding may increase the effective local concentration of the transporters in the membrane and thereby enhance transport.

In summary, the transporters investigated here show clear evidence for phospholipid headgroup interactions, particularly with anionic membranes. However, this behavior is general across the series and is not specific to the guanidine derivatives. Consequently, the lower activity of the fluorinated guanidines relative to the fluorinated (thio)ureas, as well as the unusual pH-dependence that implicates the neutral guanidine as the dominant transporting form, is unlikely to originate from unusually strong headgroup binding by the cationic guanidiniums. Instead, the pH-dependent transport of the guanidines is most likely the result of suboptimal partitioning of the cationic guanidinium species compared to neutral guanidines (or neutral (thio)ureas). This is further corroborated by the fact that the pH dependence (enhanced transport at high pH) is observed for the guanidines across all the lipid compositions tested and does not seem to be lipid specific (see SI, Fig. S127–S138).

#### Antibacterial activity

The lipid-binding and lipid-dependent transport experiments revealed that all compounds studied here preferentially partition into POPG membranes, and that several derivatives retain measurable Cl^−^/NO_3_^−^ transport in POPG vesicles—with EC_50_ values of 5.5 mol% for 1f, 0.63 mol% for 2a, 0.35 mol% for 2b, and 4.5 mol% for 2f (see SI, Fig. S164–S167). Phosphatidylglycerol (PG) lipids are commonly found in bacteria (up to 70% in Gram-positive bacteria, and ∼20% in Gram-negative bacteria), but is present only at trace levels in mammalian membranes (<1%).^68–70^ We therefore sought to investigate if the (thio)urea and guanidine derivatives investigated in this work also exhibit functional antibacterial activity.

Minimum inhibitory concentrations (MICs) were determined against three Gram-positive strains (*B. subtilis* – ATCC 6051, *E. faecalis* – ATCC 29212, and *S. aureus* – ATCC 25923) and two Gram-negative strains (*P. aeruginosa* – ATCC 27853, and *E. coli* – ATCC 25922) using standard broth microdilution methods.^71^ None of the compounds displayed activity against Gram-negative bacteria, which is unsurprising given the low PG content and the additional outer membrane barrier in these organisms. In contrast, several compounds inhibited growth in Gram-positive bacteria (MICs against *S. aureus* are summarized in Table 1; remaining MIC values are given in the SI). A clear qualitative relationship emerges between vesicle transport and antibacterial activity. Compounds that show negligible anion transport ability in vesicles (1a–1e, and 2d) also show no measurable antibacterial activity (MIC > 128 µM). Conversely, the more active anion transporters generally display antibacterial activity. The only exceptions are 2e and 2f, which are active in vesicle assays but precipitated under MIC assay conditions, preventing reliable potency determination (consistent with the deliverability problems observed for 2f in the transport experiments). Among the soluble compounds, the most efficient chloride transporters, 2a and 2b, also exhibit the strongest antibacterial potency (MIC = 0.25–1 µM), whereas 1f and 2c show only moderate activity (MIC = 16–128 µM). This nanomolar MIC of 2a makes it one of the most potent synthetic anion transporters with antibacterial activity reported to date. Importantly, none of the compounds displayed significant hemolytic activity at concentrations up to their maximum solubility (see SI, Fig. S173–S184). Furthermore, compound 2a has previously been reported to have low cytotoxicity towards human cell lines, with CC_50_ values of 178 µM against VeroE6 cells and 1 mM against A549 cells.^72^ These values are much higher than the antibacterial activity of 2a (Table 1), suggesting a promising therapeutic index of >100.

**Fig. 8:**
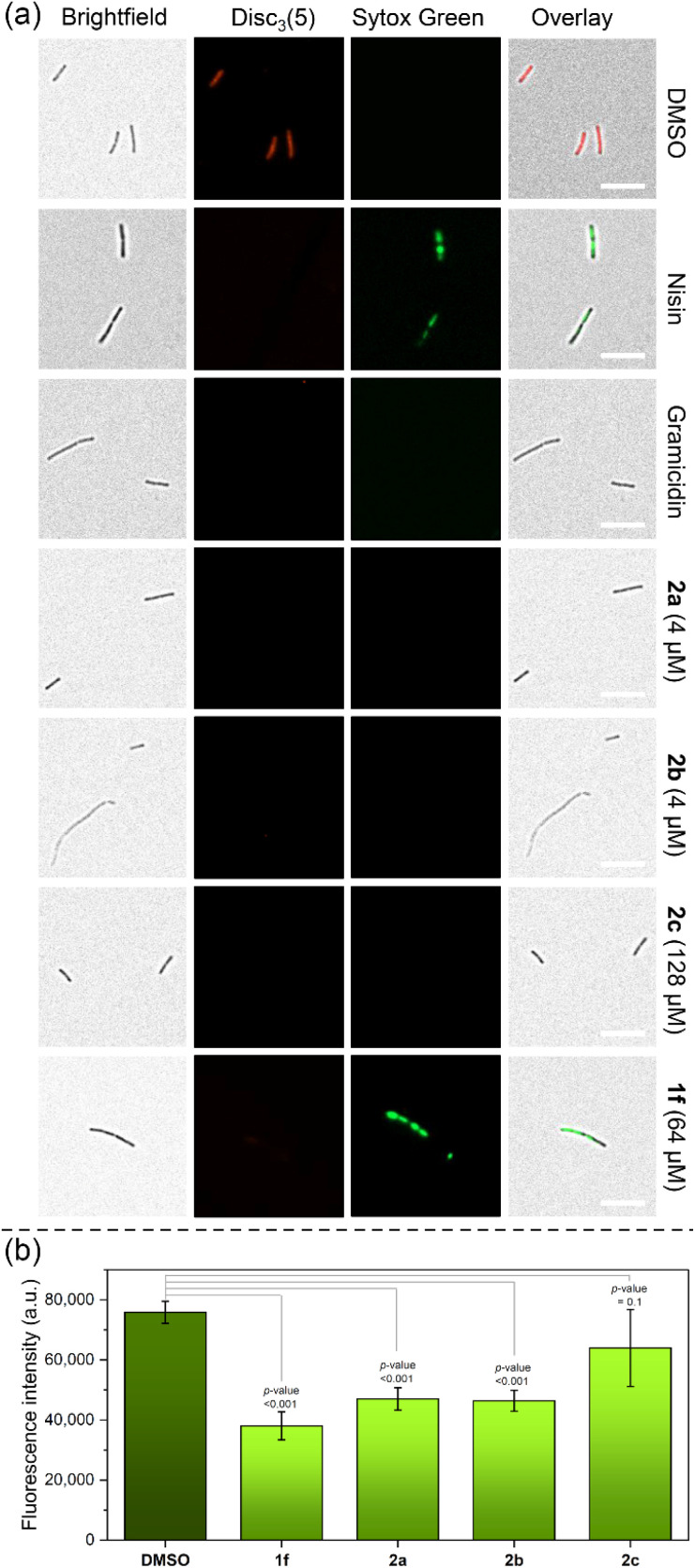
(a) Microscopy images of *B. subtilis* treated with Disc_3_(5) (red channel), Sytox Green (green channel) and the indicated antibiotics at double their MIC. Experiments were performed as described in the SI. Nisin (5 µM) served as positive control for membrane disruption, and gramicidin (2 µM) served as positive control for membrane depolarization without lysis. DMSO was used as negative control. Scale bars represent 10 µm. (b) MQAE fluorescence intensity in *B. subtilis* upon treatment with the indicated antibacterial agents at their respective MIC concentration for 10 minutes (2a (2 µM), 2b (2 µM), 2c (64 µM), and 1f (32 µM)). Experiments were conducted as described in the SI and are the average of minimum 3 independent repeats with error bars representing standard deviations.

The pronounced correlation between transport efficiency and antibacterial activity suggests that chloride transport may contribute to antibacterial activity. To test this hypothesis, mechanistic studies of antibacterial activity were performed using *B. subtilis*. Membrane depolarization was evaluated using the voltage-sensitive dye DiSC_3_(5), which accumulates and self-quenches in polarized cells and exhibits increased fluorescence upon depolarization due to dye release.^73^ At 2× MIC values, compounds 1f, 2a, 2b, and 2c induced strong depolarization responses comparable to the known depolarizing agent gramicidin (see SI, Fig. S168).^74^ Such depolarization may arise from ion gradient collapse driven by selective ion transport, but it can also be a secondary consequence of membrane permeabilization or lysis. We therefore evaluated membrane integrity using Sytox Green, a membrane-impermeable DNA-binding dye that fluoresces only upon membrane disruption (see SI, Fig. S169).^75^ To visualize depolarization and permeabilization simultaneously at the single-cell level, DiSC_3_(5) and Sytox Green were combined and analyzed by fluorescence microscopy (Fig. 8a). Gramicidin and nisin were used as positive controls for depolarization and permeabilization, respectively, while DMSO served as a negative control. Compounds 2a, 2b, and 2c produced robust membrane depolarization without Sytox Green uptake, indicating that membrane lysis does not contribute to their antibacterial activity. In contrast, 1f caused substantial Sytox Green fluorescence, consistent with membrane damage and suggesting that its antibacterial activity occurs only at concentrations sufficient to disrupt membrane integrity. This was surprising, as liposome-based calcein-leakage assays did not show significant membrane disruption at concentrations used for anion transport experiments (5 mol% transporter with respect to lipid) (see SI, Fig. S170). However, 1f is a weak anion transporter and showed relatively high MIC values. Therefore, the calcein leakage assay was repeated at much higher concentrations (100 mol% relative to lipid), and in this case membrane leakage was observed (see SI, Fig. S171–S172). These findings suggest that the potent fluorinated anion transporters 2a, 2b, and 2c act primarily through membrane depolarization without lysis, whereas the antibacterial effect of 1f become evident only under conditions that induce membrane disruption.

While 2a, 2b, and 2c clearly induce membrane depolarization, the membrane potential reflects multiple ionic species and depolarization alone does not uniquely implicate chloride transport.^20^ To provide a more direct link between chloride transport and antibacterial activity, we employed the recently reported MQAE chloride influx assay.^76^ MQAE is a halide-sensitive fluorophore whose fluorescence is quenched by chloride anions. Thus, reduced MQAE fluorescence relative to DMSO-treated cells indicates elevated intracellular chloride concentrations consistent with chloride influx. As shown in Fig. 8b, 2a and 2b induce significant MQAE quenching in *B. subtilis*, consistent with efficient chloride transport into the cells. Compound 2c produced weaker MQAE quenching, matching its lower anion transport activity observed in vesicles. Compound 1f also caused significant quenching of MQAE, but this is more likely due to its general effect on membrane leakage rather than chloride transport. Together with the Disc_3_(5)/Sytox Green microscopy results, these findings support anion transport-mediated membrane depolarization as the primary antibacterial mechanism for the potent fluorinated transporters in this series, and further underscore the potential of anionophores as antibacterial agents.

## Conclusions

In conclusion, we present a systematic benchmark of mono-guanidines *versus* mono-(thio)ureas that integrates anion binding, membrane transport, lipid association and antibacterial function within a unified structure–function framework. While several guanidines display measurable chloride transport and antibacterial activity, the corresponding (thio)ureas remain the most efficient transporters and the most potent antibacterials in this series (with urea 1a representing the anionophore with the most potent antibacterial activity reported to date). This finding is explained by a variety of mechanistic assays that converged on an unexpected central finding: anion transport is predominantly mediated by the neutral guanidine rather than the protonated guanidinium, as evidenced by the increase in transport activity at high pH. Although lipid-binding studies confirmed that the guanidines interact with the phospholipid headgroups, these interactions are comparable in magnitude to those observed for the urea and thiourea analogues, arguing against uniquely strong headgroup sequestration of the guanidinium cations as the primary cause of diminished transport. Instead, the data are most consistent with insufficient membrane partitioning and/or bilayer mobility of the more polar guanidinium form. This finding also explains the lower transport and antibacterial activity of the guanidines compared to the (thio)ureas: the current set of guanidine derivatives only have a single hydrogen bond donor in their neutral form and therefore show weak chloride binding and chloride transport. Sufficiently lipophilic guanidine derivatives with a low enough p*K*_a_ and with multiple hydrogen bond donors in their neutral form are therefore required to increase potency—a research avenue that we are currently exploring.

## Author contributions

R. D. D. S. W. A. performed most synthesis, transport studies, binding studies and bacterial studies, and co-wrote the manuscript. S. R. M. performed preliminary synthesis and anion transport studies. H. G. synthesized 1d and performed preliminary antibacterial studies. N. B. was responsible for conceptualization, oversight of the project and data analysis, and writing of the manuscript.

## Conflicts of interest

There are no conflicts to declare.

## Supplementary Material

RA-016-D6RA04370D-s001

RA-016-D6RA04370D-s002

## Data Availability

CCDC 2542395 and 2542396 contain the supplementary crystallographic data for this paper.^77*a*,*b*^ The data supporting this article have been included as part of the supplementary information (SI). Supplementary information: detailed synthesis and characterization, experimental procedures and additional figures for the p*K*_a_ determinations, anion binding studies, anion transport studies and antibacterial studies. See DOI: https://doi.org/10.1039/d6ra04370d.

## References

[cit1] Gale P. A., Davis J. T., Quesada R. (2017). Chem. Soc. Rev..

[cit2] Davis J. T., Gale P. A., Quesada R. (2020). Chem. Soc. Rev..

[cit3] Rathnaweera U. M. C., Chowdhury S. M., Salam R., Busschaert N. (2025). Chem. Rev..

[cit4] Feo E., Gale P. A. (2024). Curr. Opin. Chem. Biol..

[cit5] Jiang C., Lee E. R., Lane M. B., Xiao Y.-F., Harris D. J., Cheng S. H. (2001). Am. J. Physiol. Lung Cell. Mol. Physiol..

[cit6] Tomich J. M. , Bukovnik U., Layman J. and Schultz B. D., Cystic Fibrosis—Renewed Hopes through Research, 2012

[cit7] Fiore M., Cossu C., Capurro V., Picco C., Ludovico A., Mielczarek M., Carreira-Barral I., Caci E., Baroni D., Quesada R., Moran O. (2019). Br. J. Pharmacol..

[cit8] Li H., Valkenier H., Thorne A. G., Dias C. M., Cooper J. A., Kieffer M., Busschaert N., Gale P. A., Sheppard D. N., Davis A. P. (2019). Chem. Sci..

[cit9] Sessler J. L., Eller L. R., Cho W.-S., Nicolaou S., Aguilar A., Lee J. T., Lynch V. M., Magda D. J. (2005). Angew. Chem., Int. Ed..

[cit10] Park S.-H., Park S.-H., Howe E. N. W., Hyun J. Y., Chen L.-J., Hwang I., Vargas-Zuñiga G., Busschaert N., Gale P. A., Sessler J. L., Shin I. (2019). Chem.

[cit11] Yan T., Zheng X., Liu S., Zou Y., Liu J. (2022). Sci. China: Chem..

[cit12] Fares M., Wu X., McNaughton D. A., Gilchrist A. M., Lewis W., Keller P. A., Arias-Betancur A., Fontova P., Pérez-Tomás R., Gale P. A. (2023). Org. Biomol. Chem..

[cit13] Salunke S. B., Save S. N., Roy N. J., Naorem R., Sharma S., Talukdar P. (2024). Org. Biomol. Chem..

[cit14] Brennan L. E., Elmes R. B. P. (2025). Trends Chem..

[cit15] Yang B., Tong Z., Shi J., Wang Z., Liu Y. (2023). Med. Res. Rev..

[cit16] Sohlenkamp C., Geiger O. (2015). FEMS Microbiol. Rev..

[cit17] Hilton K. L. F., Manwani C., Boles J. E., White L. J., Ozturk S., Garrett M. D., Hiscock J. R. (2021). Chem. Sci..

[cit18] Yang M., Brackenbury W. J. (2013). Front. Psychol..

[cit19] Tran Q. H., Unden G. (1998). Eur. J. Biochem..

[cit20] Benarroch J. M., Asally M. (2020). Trends Microbiol..

[cit21] Centers for Disease Control and Prevention , Antimicrobial resistance threats in the United States, 2021–2022, Centers for Disease Control and Prevention, 2024, https://www.cdc.gov/antimicrobial-resistance/data-research/threats/update-2022.html

[cit22] Gravel J., Schmitzer A. R. (2017). Org. Biomol. Chem..

[cit23] Elie C. R., David G., Schmitzer A. R. (2015). J. Med. Chem..

[cit24] La Cognata S., Armentano D., Marchesi N., Grisoli P., Pascale A., Kieffer M., Taglietti A., Davis A. P., Amendola V. (2022). Chemistry.

[cit25] Kempf J., Schmitzer A. (2016). RSC Adv..

[cit26] Kikuchi K., Bernard E. M., Sadownik A., Regen S. L., Armstrong D. (1997). Antimicrob. Agents Chemother..

[cit27] Chen W.-H., Wennersten C., Moellering Jr R. C., Regen S. L. (2013). Chem. Biodivers..

[cit28] Maslowska-Jarzyna K., Cataldo A., Marszalik A., Ignatikova I., Butler S. J., Stachowiak R., Chmielewski M. J., Valkenier H. (2022). Org. Biomol. Chem..

[cit29] Carreira-Barral I., Rumbo C., Mielczarek M., Alonso-Carrillo D., Herran E., Pastor M., Del Pozo A., García-Valverde M., Quesada R. (2019). Commun. Chem..

[cit30] Share A. I., Patel K., Nativi C., Cho E. J., Francesconi O., Busschaert N., Gale P. A., Roelens S., Sessler J. L. (2016). Commun. Chem..

[cit31] Shinde S. V., Talukdar P. (2017). Angew. Chem., Int. Ed..

[cit32] Mukherjee S., Shinde S. V., Talukdar P., Haldar J. (2024). RSC Med. Chem..

[cit33] Das S., Karn R., Kumar M., Srimayee S., Manna D. (2024). Org. Biomol. Chem..

[cit34] Herschede S. R., Salam R., Gneid H., Busschaert N. (2023). Supramol. Chem..

[cit35] Akhtar N., Conthagamage U. N. K., Bucher S. P., Abdulsalam Z. A., Davis M. L., Beavers W. N., García-López V. (2024). Mater. Adv..

[cit36] Brennan L. E., Kumawat L. K., Piatek M. E., Kinross A. J., McNaughton D. A., Marchetti L., Geraghty C., Wynne C., Tong H., Kavanagh O. N., O'Sullivan F., Hawes C. S., Gale P. A., Kavanagh K., Elmes R. B. P. (2023). Chem.

[cit37] Brennan L. E., Luo X., Mohammed F. A., Kavanagh K., Elmes R. B. P. (2025). Chem. Sci..

[cit38] Allingham M. T., Bennett E. L., Davies D. H., Harper P. M., Howard-Jones A., Mehdar Y. T., Murphy P. J., Thomas D. A., Caulkett P. W., Potter D. (2016). Tetrahedron.

[cit39] Caine B. A., Dardonville C., Popelier P. L. (2018). ACS Omega.

[cit40] Verdolino V., Cammi R., Munk B. H., Schlegel H. B. (2008). J. Phys. Chem. BC.

[cit41] Chan D. I., Prenner E. J., Vogel H. J. (2006). Biochim. Biophys. Acta, Biomembr..

[cit42] Straus S. K. (2024). Biochim. Biophys. Acta, Biomembr..

[cit43] Maslowska-Jarzyna K., Ojah E. O., Korczak M. L., Chmielewski M. J., Busschaert N. (2025). ACS Omega.

[cit44] Danby P. M., Lombardi C., Meanwell M., Fyles T. (2017). Supramol. Chem..

[cit45] Licen S., Bagnacani V., Baldini L., Casnati A., Sansone F., Giannetto M., Pengo P., Tecilla P. (2013). Supramol. Chem..

[cit46] Khurana R., Yang F., Khurana R., Liu J., Keinan E., Reany O. (2022). Commun. Chem..

[cit47] Busschaert N., Kirby I. L., Young S., Coles S. J., Horton P. N., Light M. E., Gale P. A. (2012). Angew. Chem., Int. Ed..

[cit48] Marshall S. R., Singh A., Wagner J. N., Busschaert N. (2020). Commun. Chem..

[cit49] Salam R., Chowdhury S. M., Marshall S. R., Gneid H., Busschaert N. (2021). Commun. Chem..

[cit50] Nelson J. W., Plummer M. S., Blount K. F., Ames T. D., Breaker R. R. (2015). Chem. Biol..

[cit51] Liu J., Weng Q., Da D., Yao S., Zhang Y., Wu Y. (2024). Antibiotics.

[cit52] Dangate P. S., Akamanchi K. G. (2012). Tetrahedron Lett..

[cit53] Busschaert N., Wenzel M., Light M. E., Iglesias-Hernández P., Pérez-Tomás R., Gale P. A. (2011). J. Am. Chem. Soc..

[cit54] Yoo J., Cui Q. (2008). Biophys. J..

[cit55] Li L., Vorobyov I., MacKerell A. D., Allen T. W. (2008). Biophys. J..

[cit56] Paixão J. A., Pereira Silva P. S., Matos Beja A., Ramos Silva M., De Matos Gomes E., Belsley M. (1999). Acta Crystallogr., Sect. C: Cryst. Struct. Commun..

[cit57] Silva M. R., Paixão J. A., Beja A. M., da Veiga L. A. (2001). J. Fluorine Chem..

[cit58] Binoy J., James C., Hubert Joe I., Jayakumar V. S. (2006). J. Mol. Struct..

[cit59] McNally B. A., Koulov A. V., Smith B. D., Joos J.-B., Davis A. P. (2005). Chem. Commun..

[cit60] Hill A. V. (1913). Biochem. J..

[cit61] McNally B. A., O'Neil E. J., Nguyen A., Smith B. D. (2008). J. Am. Chem. Soc..

[cit62] Jowett L. A., Howe E. N., Wu X., Busschaert N., Gale P. A. (2018). Chem.--Eur. J..

[cit63] Wu X., Judd L. W., Howe E. N., Withecombe A. M., Soto-Cerrato V., Li H., Busschaert N., Valkenier H., Perez-Tomas R., Sheppard D. N. (2016). Chem.

[cit64] Jowett L. A., Gale P. A. (2019). Supramol. Chem..

[cit65] Wu X., Small J. R., Cataldo A., Withecombe A. M., Turner P., Gale P. A. (2019). Angew. Chem., Int. Ed..

[cit66] Torrisi J., Chvojka M., Jurček P., Zhang X., Zeng H., Šindelář V., Valkenier H. (2025). Angew. Chem., Int. Ed..

[cit67] Herschede S. R., Gneid H., Dent T., Jaeger E. B., Lawson L. B., Busschaert N. (2021). Org. Biomol. Chem..

[cit68] Malanovic N., Lohner K. (2016). Biochim. Biophys. Acta, Biomembr..

[cit69] Epand R. F., Savage P. B., Epand R. M. (2007). Biochim. Biophys. Acta, Biomembr..

[cit70] Vance J. E. (2015). Traffic.

[cit71] Balouiri M., Sadiki M., Ibnsouda S. K. (2016). J. Pharm. Anal..

[cit72] Kumar N., Taily I. M., Singh C., Kumar S., Rajmani R. S., Chakraborty D., Sharma A., Singh P., Thakur K. G., Varadarajan R. (2023). PLoS Pathog..

[cit73] Te Winkel J. D., Gray D. A., Seistrup K. H., Hamoen L. W., Strahl H. (2016). Front. Cell Dev. Biol..

[cit74] Kelkar D. A., Chattopadhyay A. (2007). Biochim. Biophys. Acta.

[cit75] Roth B. L., Poot M., Yue S. T., Millard P. J. (1997). Appl. Environ. Microbiol..

[cit76] Brennan L. E., Kumawat L. K., Piatek M. E., Kinross A. J., McNaughton D. A., Marchetti L., Geraghty C., Wynne C., Tong H., Kavanagh O. N. (2023). Chem.

[cit77] (a) CCDC 2542395: Experimental Crystal Structure Determination, 2026, 10.5517/ccdc.csd.cc2rbkrf

