# Supplementary material for: Lipophilic mono-guanidines versus mono-(thio)ureas: anion transport, lipid binding, and antibacterial activity
Source: RSC Adv. 2026 Jul 6. Online ahead of print. doi: 10.1039/d6ra04370d (PMC13334524; doi:10.1039/d6ra04370d)
Supplement: RA-OLF-D6RA04370D-s001 [file RA-OLF-D6RA04370D-s001.pdf]

Supplementary Information (SI) for RSC Advances.
This journal is © The Royal Society of Chemistry 2026

Supporting Information:

Lipophilic mono-guanidines versus mono-(thio)ureas: anion transport, lipid binding, and antibacterial activity

Randima D. De Silva Weerakonda Arachchige,[a] Sarah R. Marshall,[a] Hassan Gneid,[a] and Nathalie Busschaert[a]

[a] Department of Chemistry, Tulane University, New Orleans, LA 70118, United States. Email: nbuss-chaert@tulane.edu

Table of Contents

S1 General information S3
S2 Synthesis and Characterization S3
S3 pK_a Determination S19
S4 ^{1}H NMR Titrations S34
S5 Single Crystal X-ray Diffraction S47
S5.1 Crystal Data for **2c·HCl** S47
S5.2 Crystal Data for **2d·HCl** S52
S6 Chloride Transport Assays S56
S6.1 Lucigenin Assay (external addition or pre-incorporation) S56
S6.2 Effect of Organic Solvent Amount on Transport S71
S6.3 Hill Plots (EC_{50} values) S72
S6.4 Transport Mechanism S76
S6.4.1 Anion/Cation Selectivity S76
S6.4.2 Carrier vs Ion Channel S83
S6.4.3 HPTS Assay S89
S6.4.4 Cationophore Coupled Assay S96
S6.5 pH Dependent Transport S103
S6.6 Lipid Dependent Transport S113
S7 Lipid Binding Assay S121
S7.1.1 Fluorescence Titrations with N-Propyl-7-nitro-2,1,3-benoxadiazol-4-amine ... S121
S7.1.2 Fluorescence Titrations with POPG and POPC S122
S8 Hill plots (EC_{50} POPG) S136
S9 Antibacterial Activity S138
S9.1 Minimum Inhibitory Concentrations S138
S9.2 Membrane Depolarization S139
S9.3 Sytox Green Influx S140
S9.4 $Disc_3(5)$ and Sytox Green Microscopy S142
S9.5 MQAE Chloride Influx Assay S142
S10 Calcein Leakage Assay S143
S11 Hemolytic activity S145
S12 References S152

S1 General information

Solvents, reagents, and inorganic salts were obtained from Millipore Sigma or Fisher Scientific and were used without further purification. 2-Iodoxybenzoic acid (IBX) was purchased as a stabilized product (45 wt%) and was used as provided with stabilizer—taking into account the weight of the stabilizer when calculating equivalents. Compound names are those generated by ChemDraw 21.0.0.28, following IUPAC nomenclature. Column chromatography was performed using aluminum oxide (basic, Brockmann I, 40-300 µm, 60A). Thin-layer chromatography (TLC) was executed using silica gel or alumina TLC plates with a fluorescent indicator, and the plates were visualized under ultraviolet (UV) light (254 nm) or by staining with ninhydrin solutions. ^{1}H NMR and ^{13}C NMR spectra were collected on a Bruker 400 MHz NMR spectrometer and ^{13}C NMR spectra were obtained proton decoupled. NMR samples were dissolved in DMSO-d_6 with a few drops of trifluoroacetic acid (TFA) to ensure full protonation of the guanidinium compounds, which gives better peak resolution. NMR processing was conducted using MestReNova 14.3.1-31739. Chemical shifts (δ) are reported in parts per million (ppm) and calibrated to the residual solvent peak in DMSO-d_6 (δ = 2.50 ppm (^{1}H) and 39.5 ppm (^{13}C)). Coupling constants (J) are expressed in Hertz (Hz). The following abbreviations are used for spin multiplicity: s = singlet, d = doublet, t = triplet, q = quartet, p = pentet, sx = sextet, m = multiplet, br. = broad. Infrared (IR) spectra were recorded on a Thermo Scientific Nicolet iS10 FTIR spectrometer; only selected maximum absorbances (ν_{max}) of the most intense peaks are reported (cm^{-1}). Electrospray Ionization (ESI) mass spectra were obtained on a Bruker microTOF. The lipids POPC (1 palmitoyl-2-oleoyl-glycero-3-phosphocholine), POPG (1-palmitoyl-2-oleoyl-*sn*-glycero-3-phospho-(1'-rac-glycerol) (sodium salt)), DPPC (1,2-dipalmitoyl-*sn*-glycero-3-phosphocholine), 18:1-6:0 NBD-PC (1-oleoyl-2-(6-((7-nitro-2-1,3-benzoxadiazol-4-yl)amino)hexanoyl)-*sn*-glycero-3-phosphocholine) and 18:1-6:0 NBD-PG (1-oleoyl-2-(6-[(7-nitro-2-1,3-benzoxadiazol-4-yl)amino]hexanoyl)-*sn*-glycero-3-[phospho-rac-(1-glycerol)] (ammonium salt)) were purchased from Avanti Polar Lipids, Inc. Fluorescence spectra and kinetic studies were conducted on an Agilent Cary Eclipse fluorescence spectrophotometer equipped with a stirring function and Peltier temperature controller. 3 mL macrocuvettes were used, and all solutions were stirred with a cuvette stir bar (Sigma- Aldrich # Z 363545). Bacterial strains were acquired from the American Type Culture Collection (ATCC). Hemolysis assays were performed using a BioTek Cytation 5 Cell Imaging Multi-Mode Reader using washed single-donor human red blood cells were sourced from Innovative Research, Inc. (IWB 3 ALS). Phosphate buffered saline (PBS) was purchased from Gibco (#10- 010- 031).

S2 Synthesis and Characterization

Compounds **1a** (1,3-diphenylurea, CAS: 142-15-8), **1b** (1,3-diphenylthiourea, CAS: 102-08-9), and **1c** (1,3-diphenylguanidine, CAS: 102-06-7) were acquired from Sigma-Aldrich and utilized without further purification. Compounds **2a** and **2b** were synthesized according to methods previously detailed in the literature.[1] Compound **1d** was synthesized according a previously reported method

using IBX as an oxidizing agent (see *Scheme 1*).[2] All other guanidine compounds were synthesized using the same IBX method.

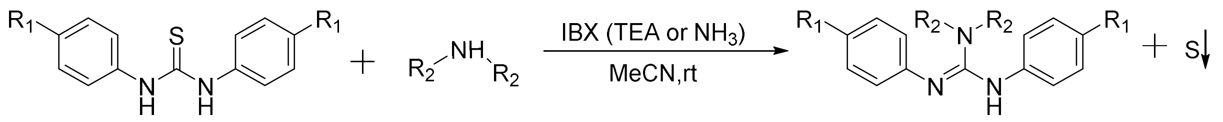

Scheme S1. Synthetic scheme for the preparation of compounds **1d-1f** and **2c-2e**.

General procedure for compounds 1e and 1f

In an oven-dried 100 mL round-bottom flask equipped with a stir bar, 1,3-diphenylthiourea (1 eq) was dissolved in acetonitrile, followed by the addition of dibutylamine (for **1e**) or dihexylamine (for **1f**) (1.5 eq). The mixture was stirred until complete dissolution. Meanwhile, in a separate vial, 2-iodoxybenzoic acid (IBX, 45 wt%, 2 eq) was suspended in acetonitrile and excess triethylamine was added until all IBX was dissolved. This IBX solution was added dropwise to the thiourea mixture and the reaction was stirred at room temperature for at least 48 hours (until TLC indicated full conversion of the thiourea starting material). After completion, the reaction mixture was concentrated using a rotary evaporator. The crude product was then treated with ethyl acetate, sonicated, and filtered to remove precipitated white solids (mostly unreacted thiourea). The filtrate was transferred to a separatory funnel and extracted at least three times with an aqueous saturated sodium bicarbonate solution. The organic layer was dried over anhydrous magnesium sulfate, filtered, and concentrated under reduced pressure. The crude residue was purified by column chromatography over basic alumina using hexanes and a slow gradient of ethyl acetate (starting with 100% hexanes).

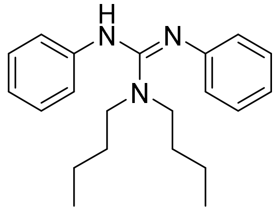

1,1-dibutyl-2,3-diphenylguanidine (1e). Brown oil (575 mg, 1.8 mmol). Yield: 81%. ^{1}H NMR (400 MHz, DMSO-d_6 with a few drops of TFA, ppm) δ 10.18 (s, 2H), 7.22 (m, 4H), 7.01 (m, 6H), 3.51 (t, *J* = 7.3 Hz, 4H), 1.61 (p, *J* = 7.3 Hz, 4H), 1.30 (sx, *J* = 7.3 Hz, 4H), 0.88 (t, *J* = 7.3 Hz, 6H). ^{13}C NMR (101 MHz, DMSO-d_6 with a few drops of TFA, ppm) δ 153.3, 137.3, 129.2, 124.9, 121.3, 49.2, 29.2, 19.2, 13.5. IR (neat): ν (cm^{-1}) = 3385, 2956, 2928, 2870, 1614, 1576, 746, 689. HRMS (ESI/Q-TOF) for $C_{21}H_{30}N_3$ [M+H]$^+$ m/z= 324.2440 (calculated), 324.2441 (observed).

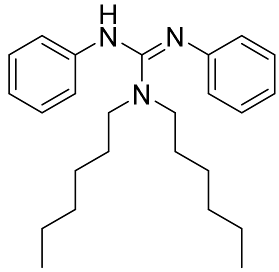

1,1-dihexyl-2,3-diphenylguanidine (1f). White solid (254 mg, 0.67 mmol). Yield: 13%. ^{1}H NMR (400 MHz, DMSO-d_6 with a few drops of TFA, ppm) δ 10.12 (s, 2H), 7.19 (m, 4H), 6.99 (m, 6H), 3.49 (t, *J* = 7.3 Hz, 4H), 1.60 (p, *J* = 7.3 Hz, 4H), 1.24 (m, 12H), 0.81 (m, 6H). ^{13}C NMR (101 MHz, DMSO-d_6 with a few drops of TFA, ppm) δ 153.2, 137.3, 129.2, 125.0, 121.2, 49.4, 30.8, 27.0, 25.5, 22.0, 13.9. IR (neat): ν (cm^{-1}) = 3153, 2955, 2925, 2856, 1567, 1314, 1110, 1066, 745, 692. HRMS (ESI/Q-TOF) for $C_{25}H_{38}N_3$ [M+H]$^+$ *m/z* =380.3066 (calculated), 380.3079 (observed).

Procedure for 1,3-bis(4-(trifluoromethyl)phenyl)guanidine (2c).

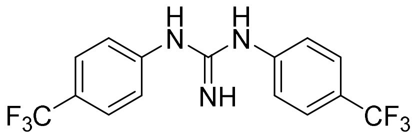

In a 100 mL oven-dried round bottom flask, 1,3-bis(4-(trifluoromethyl)phenyl)thiourea **2b** (1.01 g, 2.8 mmol, 1 eq) was dissolved fully in 10 mL of acetonitrile. In a separate vial, 2-iodoxybenzoic acid (IBX) (3.06 g of 45 wt%, 4.9 mmol, 1.75 eq) was added to 15 mL of aqueous ammonium hydroxide solution (28%) and allowed to stir until the IBX was fully dissolved. This IBX solution was then added dropwise to the thiourea solution over 10 minutes. The reaction was allowed to stir for at least 48 hours at room temperature, until TLC indicated full conversion of the thiourea starting material. The resulting thick syrup was treated with about 30 mL ethyl acetate and extracted at least three times with an aqueous saturated sodium bicarbonate solution. The organic layer was dried over anhydrous magnesium sulfate, filtered, and concentrated under reduced pressure. The crude product was purified by column chromatography (basic alumina), using a slow gradient of ethyl acetate in hexanes (starting with 100% hexanes). The purified fractions were combined and evaporated to dryness, to give compound **2c** as a glassy solid (580 mg, 1.7 mmol). Yield: 61%. ^{1}H NMR (400 MHz, DMSO-d_6 with a few drops of TFA, ppm) δ 10.70 (s, 2H), 8.67 (s, 2H), 7.79 (m, 4H), 7.52 (m, 4H). ^{13}C NMR (101 MHz, DMSO-d_6 with a few drops of TFA, ppm) δ 153.7, 140.2, 126.8 (q,$^3J_{C\text{-}F}$= 3.6 Hz), 126.0 (q,$^2J_{C\text{-}F}$= 32.3 Hz), 124.2 (q, $^1J_{C\text{-}F}$ = 272.2 Hz), 123.6. IR (neat): ν (cm^{-1}) = 1564, 1314, 1104, 1065, 836. HRMS (ESI/Q-TOF) for $C_{15}H_{12}F_6N_3$ $[M+H]^+$ m/z =348.0935 (calculated); found 348.0935 (observed).

General procedure for compounds 2d-2f

In a 100 mL oven-dried round bottom flask, 1,3-bis(4-(trifluoromethyl)phenyl)thiourea **2b** (1 eq) and the appropriate dialkylamine (1.5 eq) were added to acetonitrile and stirred until all compounds were fully dissolved. Meanwhile, in a separate vial, 2-iodoxybenzoic acid (IBX, 45 wt%, 2 eq) was added to acetonitrile and a large excess of triethylamine was added until the IBX was fully dissolved. At that point, the IBX solution was added dropwise to the thiourea solution and the reaction was allowed to stir for at least 48 hours at room temperature, until TLC indicated full conversion of the thiourea starting material. After completion, the reaction mixture was concentrated using a rotary evaporator. This crude product was treated with ethyl acetate, sonicated, and filtered to remove precipitated white solids (mostly unreacted thiourea). The filtrate was transferred to a separatory funnel and extracted at least three times with an aqueous saturated sodium bicarbonate solution. The organic layer was dried over anhydrous magnesium sulfate, filtered, and concentrated under reduced pressure. The crude residue was purified by column chromatography over basic alumina using hexanes and a slow gradient of ethyl acetate (starting with 100% hexanes).

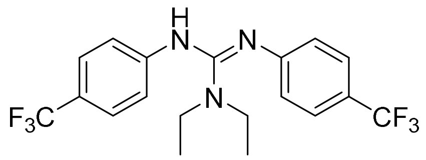

1,1-diethyl-2,3-bis(4-(trifluoromethyl)phenyl)guanidine (2d). Fluffy white solid (1.09 g, 2.7 mmol). Yield: 89%, ^{1}H NMR (400 MHz, DMSO-d_6 with a few drops of TFA, ppm) δ 10.45 (s, 2H), 7.53 (m, 4H), 7.22 (m, 4H), 3.62 (q, J = 7.1 Hz, 4H), 1.26 (t, J = 7.1 Hz, 6H). ^{13}C NMR (101 MHz, DMSO-d_6 with a few drops of TFA) δ 152.8, 141.0, 126.2 (q,$^3J_{C-F}$= 3.6 Hz), 125.1 (q,$^2J_{C-F}$ = 32.3 Hz), 124.0 (q,$^1J_{C-F}$= 272.7 Hz), 122.0, 44.6, 12.8. IR (neat): v (cm^{-1}) = 2977, 1564, 1314, 1104, 1065, 836. HRMS (ESI/Q-TOF) for $C_{19}H_{20}F_6N_3$ $[M+H]^+$ m/z = 404.1561(calculated); 404.1555 (observed).

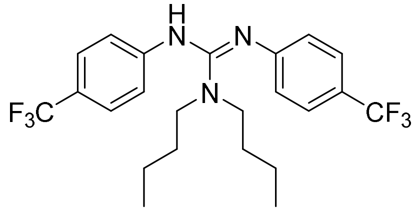

1,1-dibutyl-2,3-bis(4-(trifluoromethyl)phenyl)guanidine (2e). White solid (0.605 mg, 1.3 mmol). Yield: 96%, ^{1}H NMR (400 MHz, DMSO-d_6 with a few drops of TFA, ppm) δ 10.63 (s, 2H), 7.53 (m, 4H), 7.17 (m, 4H), 3.60 (t, J = 7.3 Hz, 4H), 1.64 (p, J = 7.3 Hz, 4H), 1.31 (sx, J = 7.3 Hz, 4H), 0.88 (t, J = 7.3 Hz, 6H). ^{13}C NMR (101 MHz, DMSO-d_6 with a few drops of TFA, ppm) δ 153.0, 140.8, 126.3 (q, $^3J_{C-F}$ = 3.6 Hz), 124.9 (q, ,$^2J_{C-F}$ = 32.3 Hz), 124.0 (q, $^1J_{C-F}$ = 272.7 Hz), 121.6, 49.5, 29.1, 19.1, 13.5. IR (neat): v (cm^{-1}) = 2954, 2867, 1557, 1330, 1154, 1104, 1067, 839. HRMS (ESI/Q-TOF) for $C_{23}H_{28}F_6N_3$ $[M+H]^+$ m/z = 460.2187 (calculated); 460.2173 (observed).

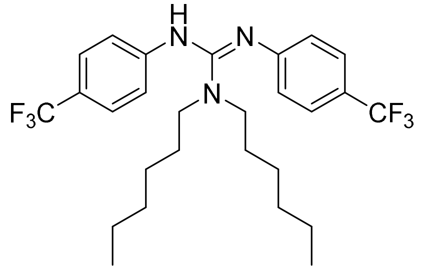

1,1-dihexyl-2,3-bis(4-(trifluoromethyl)phenyl)guanidine (2f). White solid (0.675 mg, 1.3 mmol). Yield: 68%. ^{1}H NMR (400 MHz, DMSO-d_6 with a few drops of TFA, ppm) δ 10.63 (s, 2H), 7.53 (m, 4H), 7.17 (m, 4H), 3.59 (t, J = 7.3 Hz, 4H), 1.65 (p, J = 7.3 Hz, 4H), 1.26 (m, 12H), 0.82 (t, J = 7.3 Hz, 6H). ^{13}C NMR (101 MHz, DMSO-d_6 with a few drops of TFA, ppm) δ 153.0, 140.8, 126.3 (q, $^3J_{C-F}$ = 3.6 Hz), 125.0 (q, $^2J_{C-F}$ = 32.3 Hz), 124.0 (q, $^1J_{C-F}$ = 272.7 Hz), 121.6, 49.8, 30.8, 27.0, 25.5, 22.0, 13.8. IR (neat): v (cm^{-1}) = 2929, 2858, 1563, 1515, 1316, 1276, 1155, 1106, 1066, 836. HRMS (ESI/Q-TOF) for $C_{27}H_{36}F_6N_3$ $[M+H]^+$ m/z = 516.2813 (calculated); 516.2817 (observed).

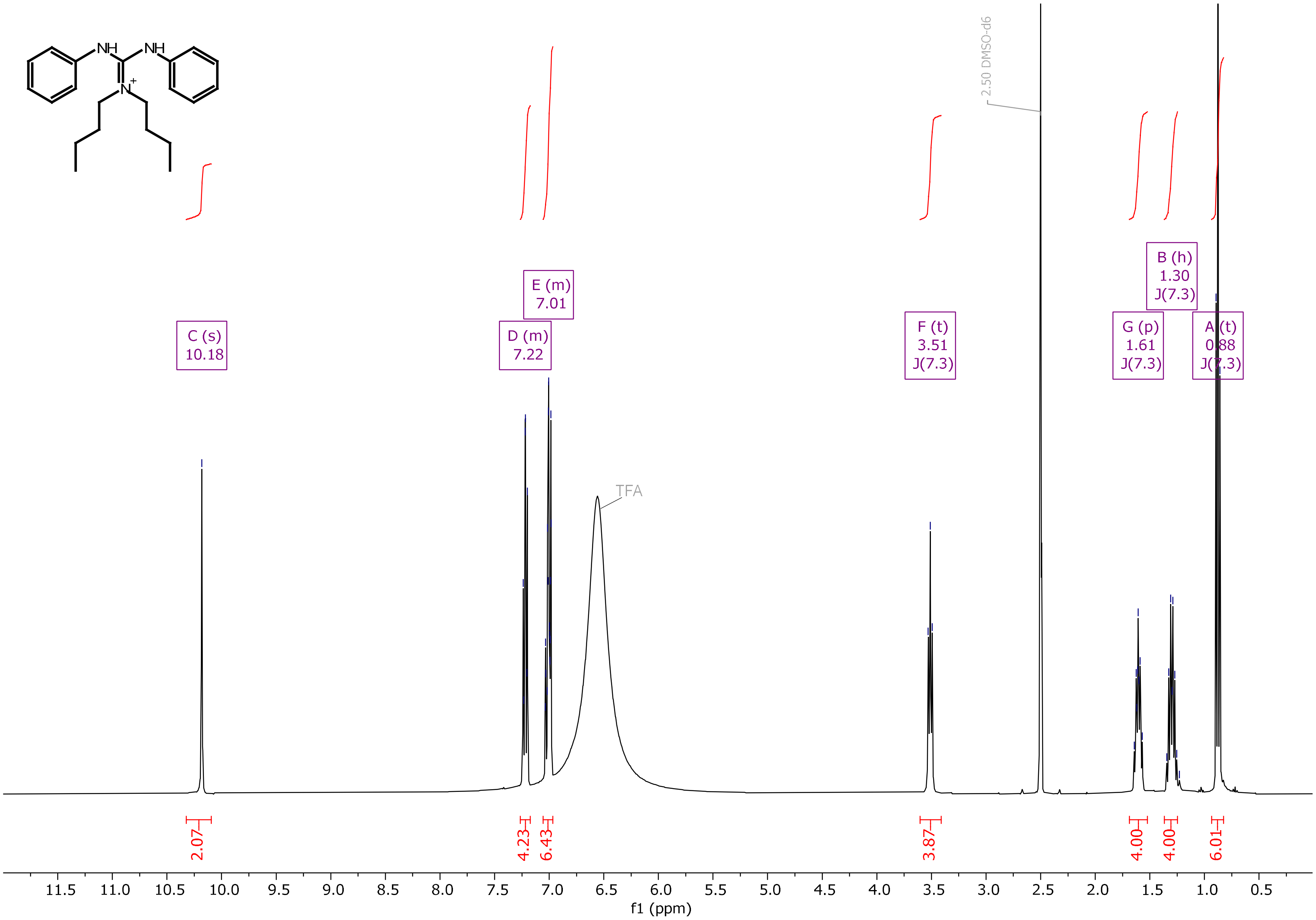

Figure S1. ^{1}H NMR (400 Hz) spectrum of **1e** DMSO-d_6 (with a few drops of TFA) at 298 K.

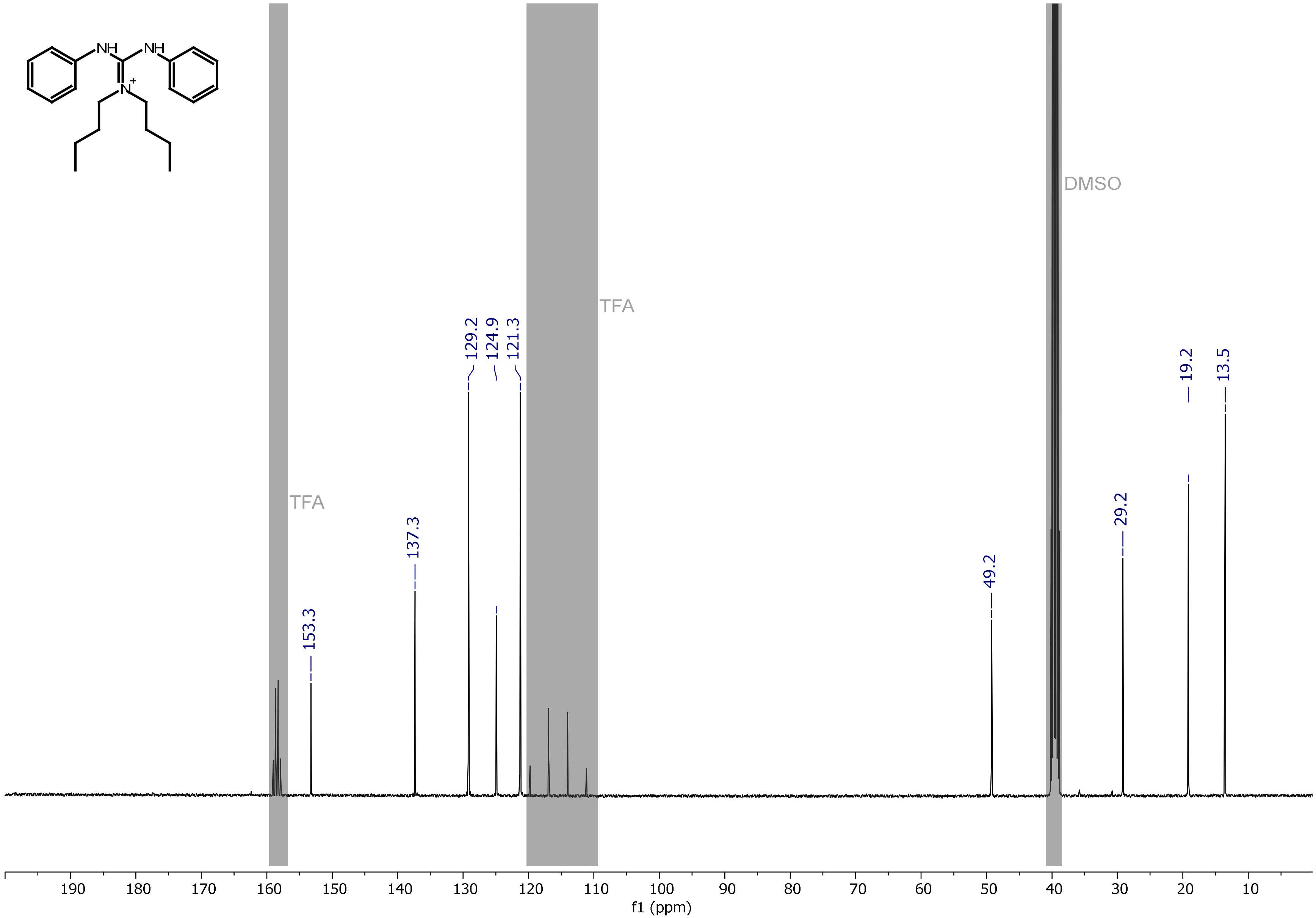

Figure S2. ^{13}C NMR (400 MHz) spectrum of **1e** DMSO-d_6 (with a few drops of TFA) at 298 K.

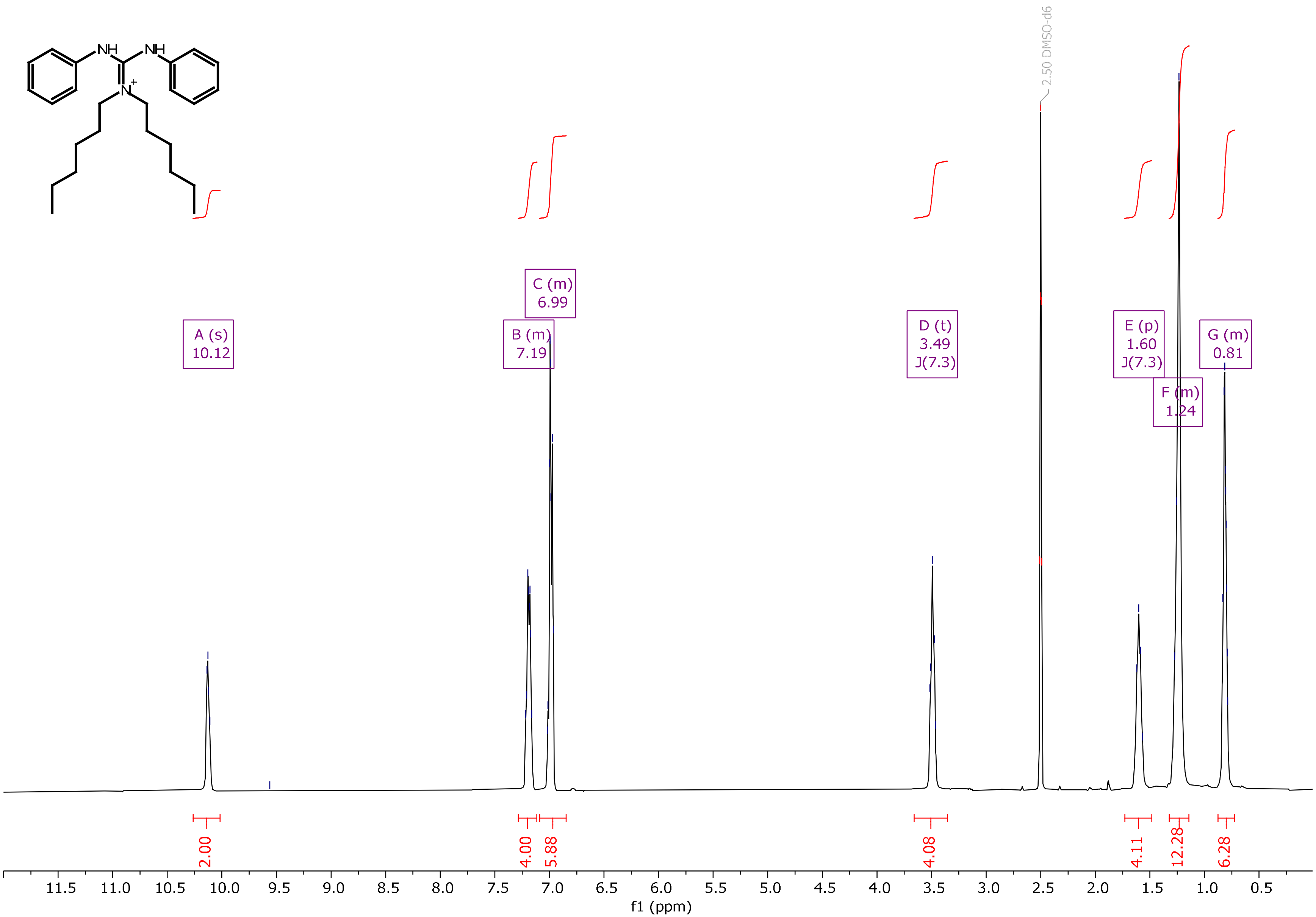

Figure S3. ^{1}H NMR (400 MHz) spectrum of **1f** DMSO-d_6 (with a few drops of TFA) at 298 K.

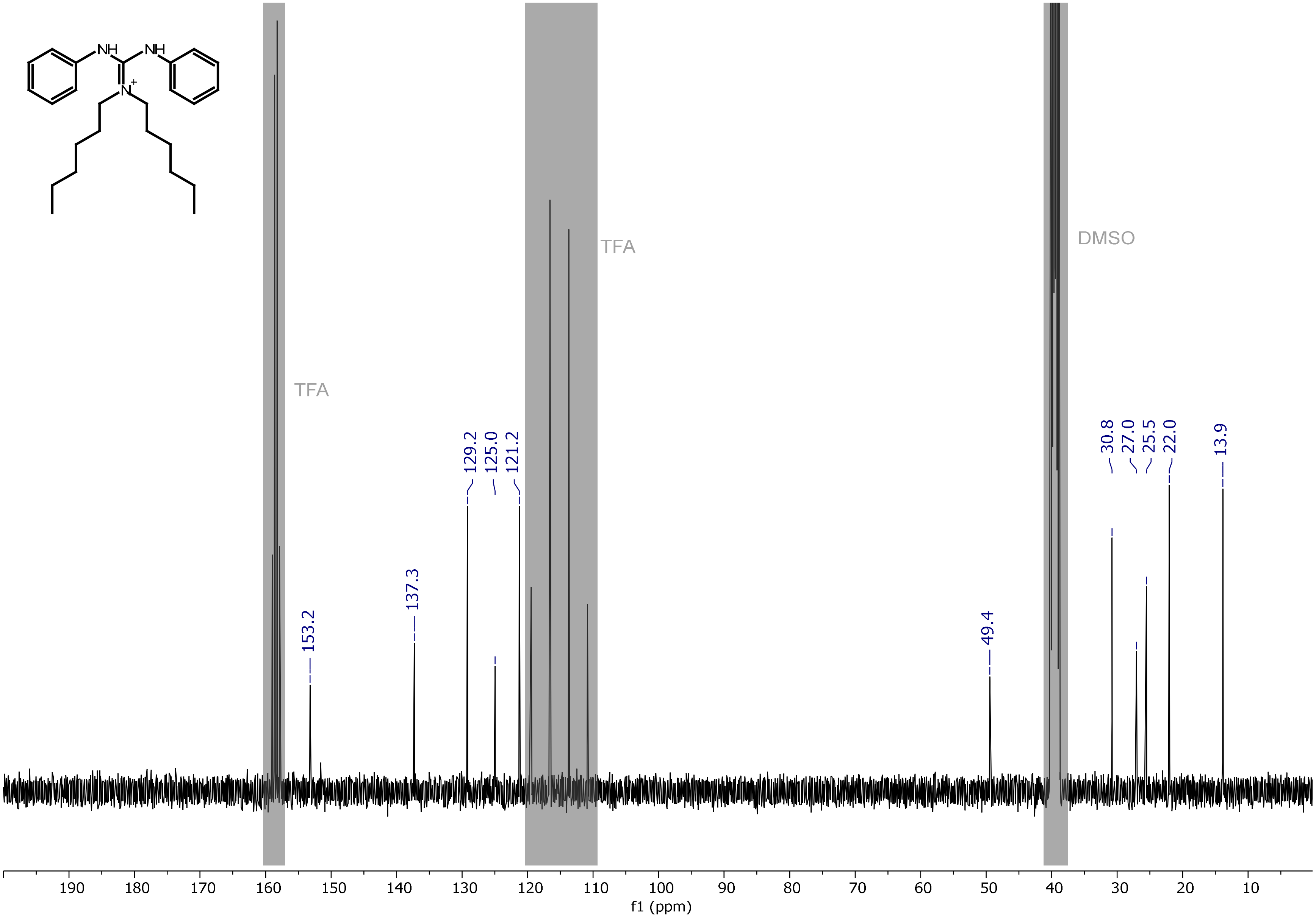

Figure S4. ^{13}C NMR (400 MHz) spectrum of **1f** DMSO-d_6 (with a few drops of TFA) at 298 K.

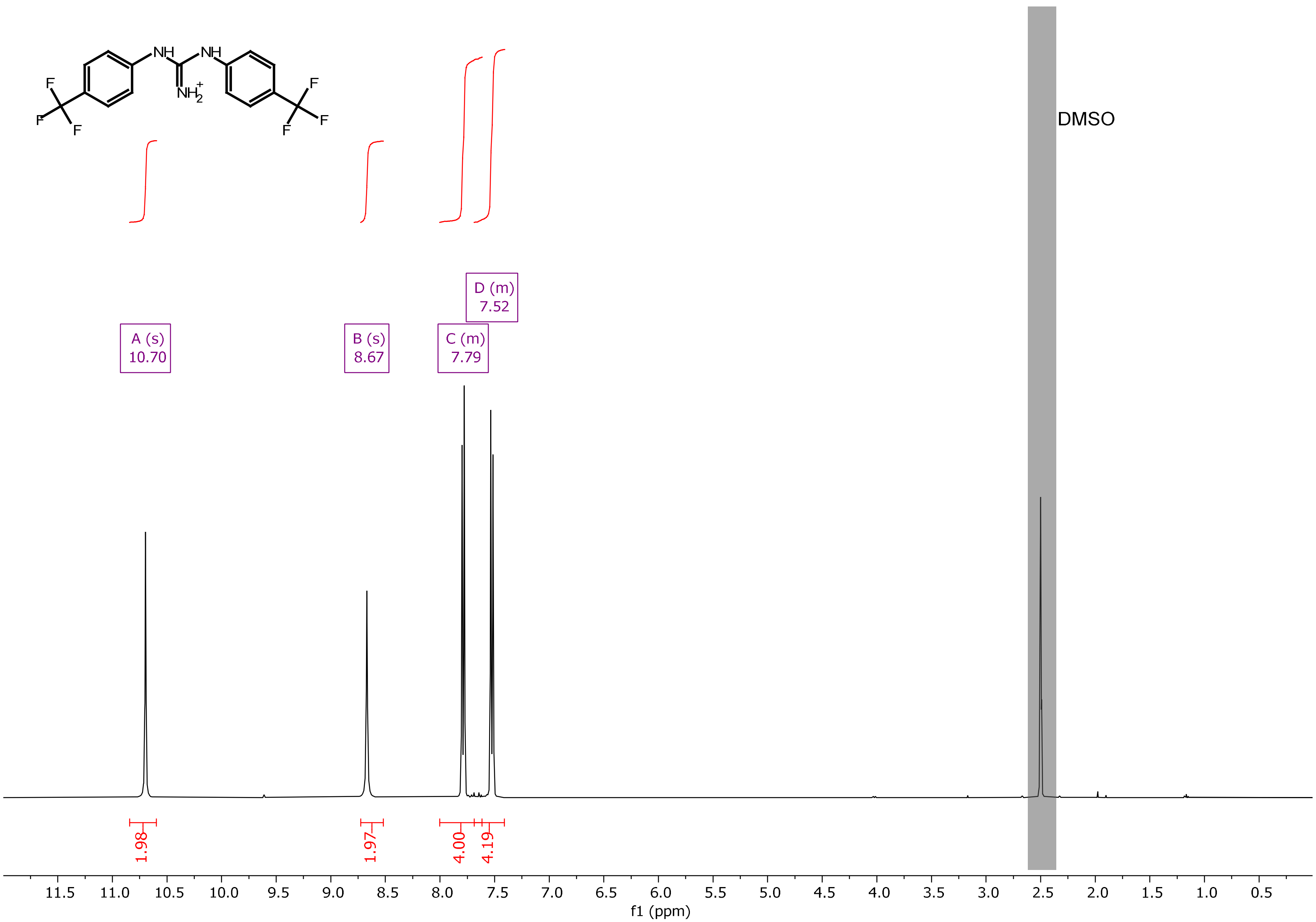

Figure S5. ^{1}H NMR (400 MHz) spectrum of **2c** DMSO-d_6 (with a few drops of TFA) at 298 K.

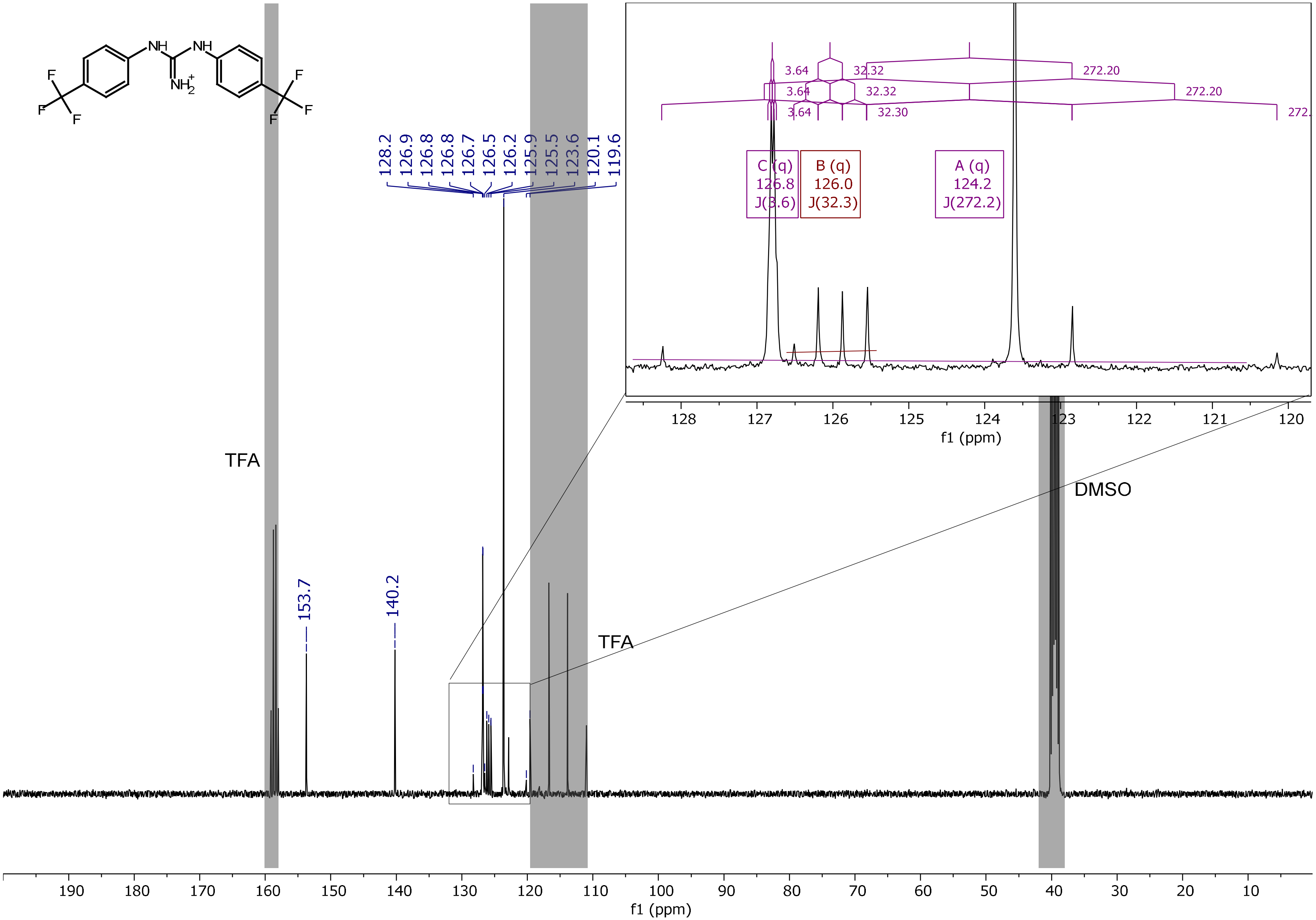

Figure S6. ^{13}C NMR (400 MHz) spectrum of **2c** DMSO-d_6 (with a few drops of TFA) at 298 K.

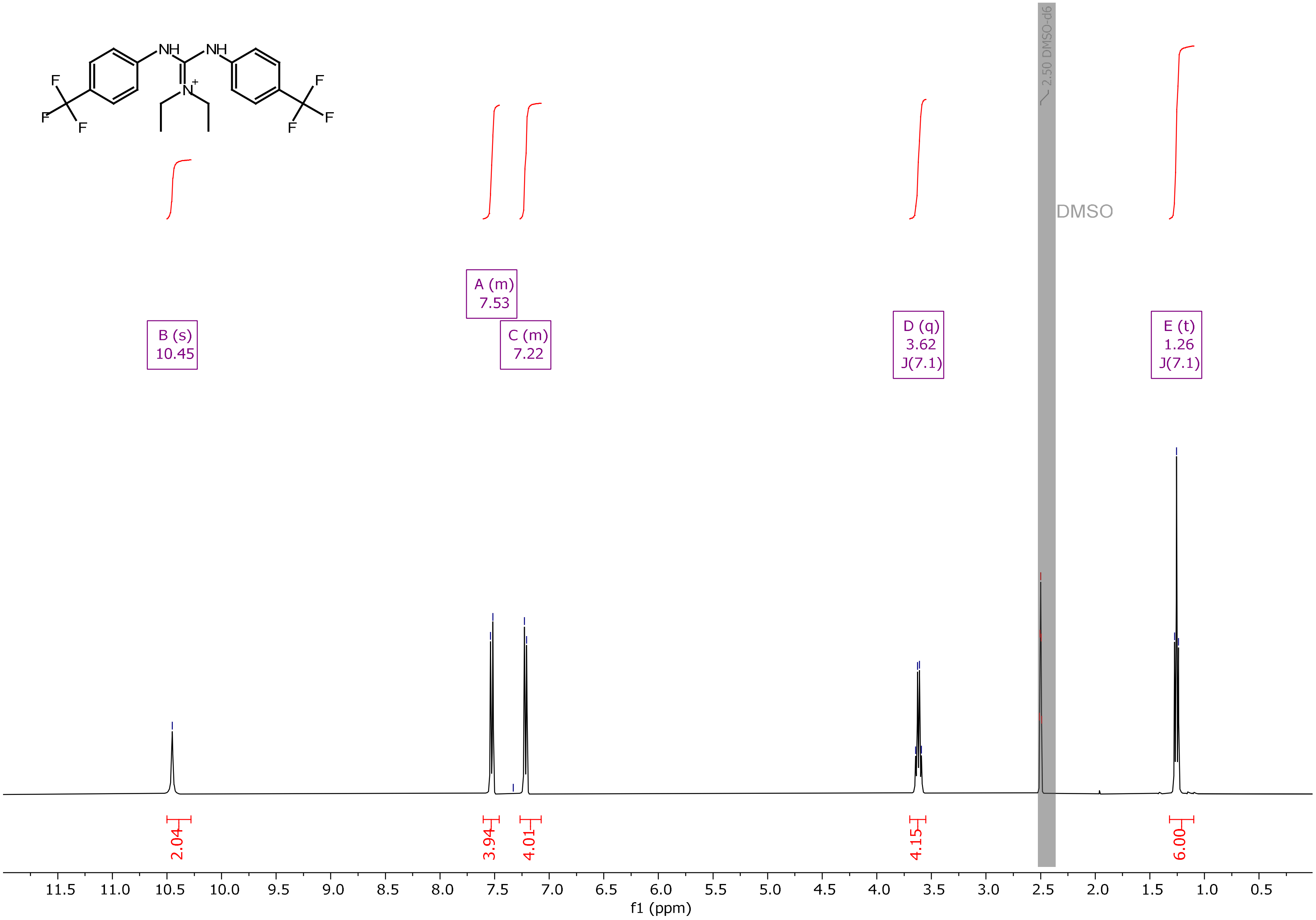

Figure S7. ^{1}H NMR (400 MHz) spectrum of **2d** DMSO-d$_6$ (with a few drops of TFA) at 298 K.

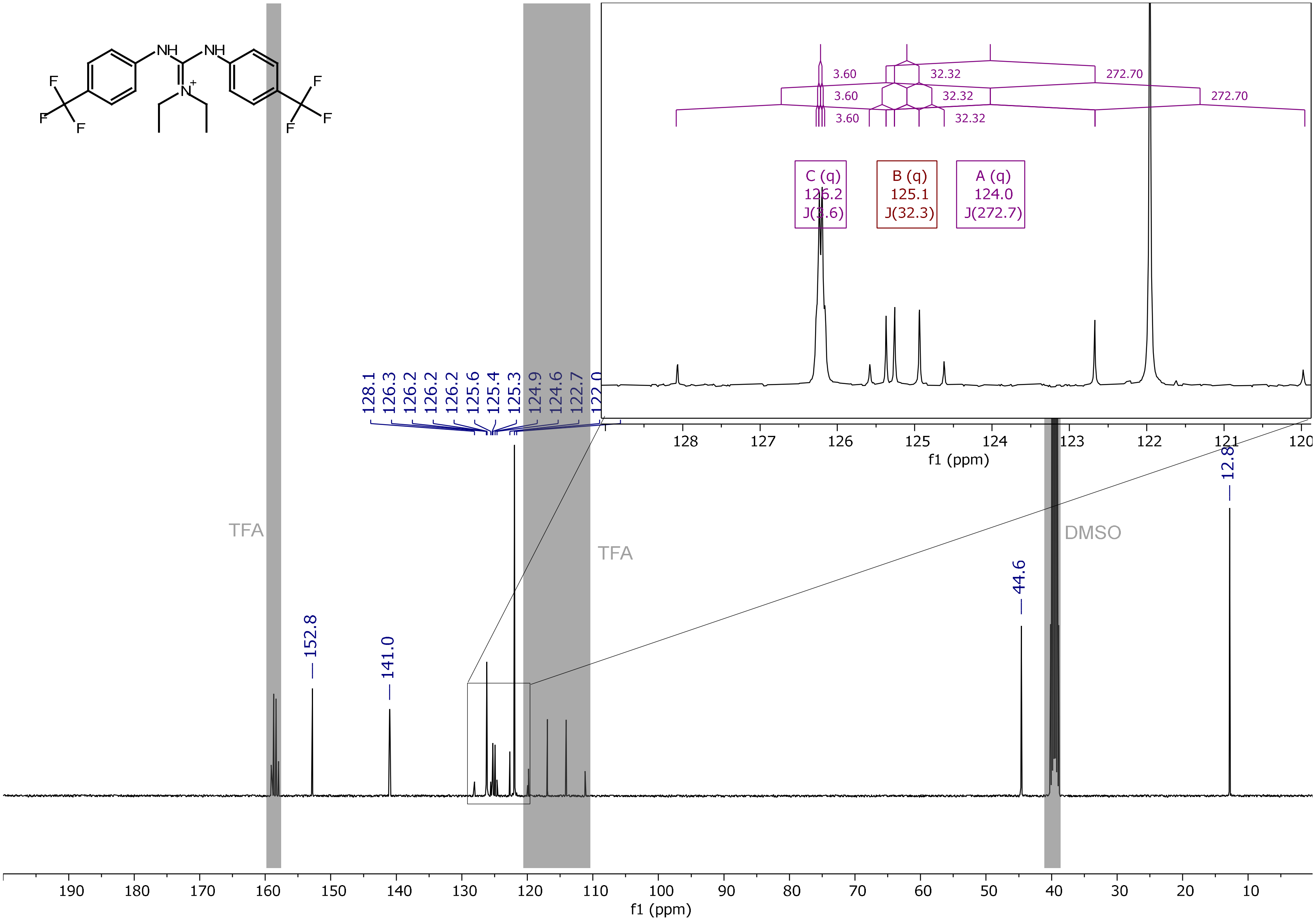

Figure S8. ^{13}C NMR (400 MHz) spectrum of **2d** DMSO-d_6 (with a few drops of TFA) at 298 K.

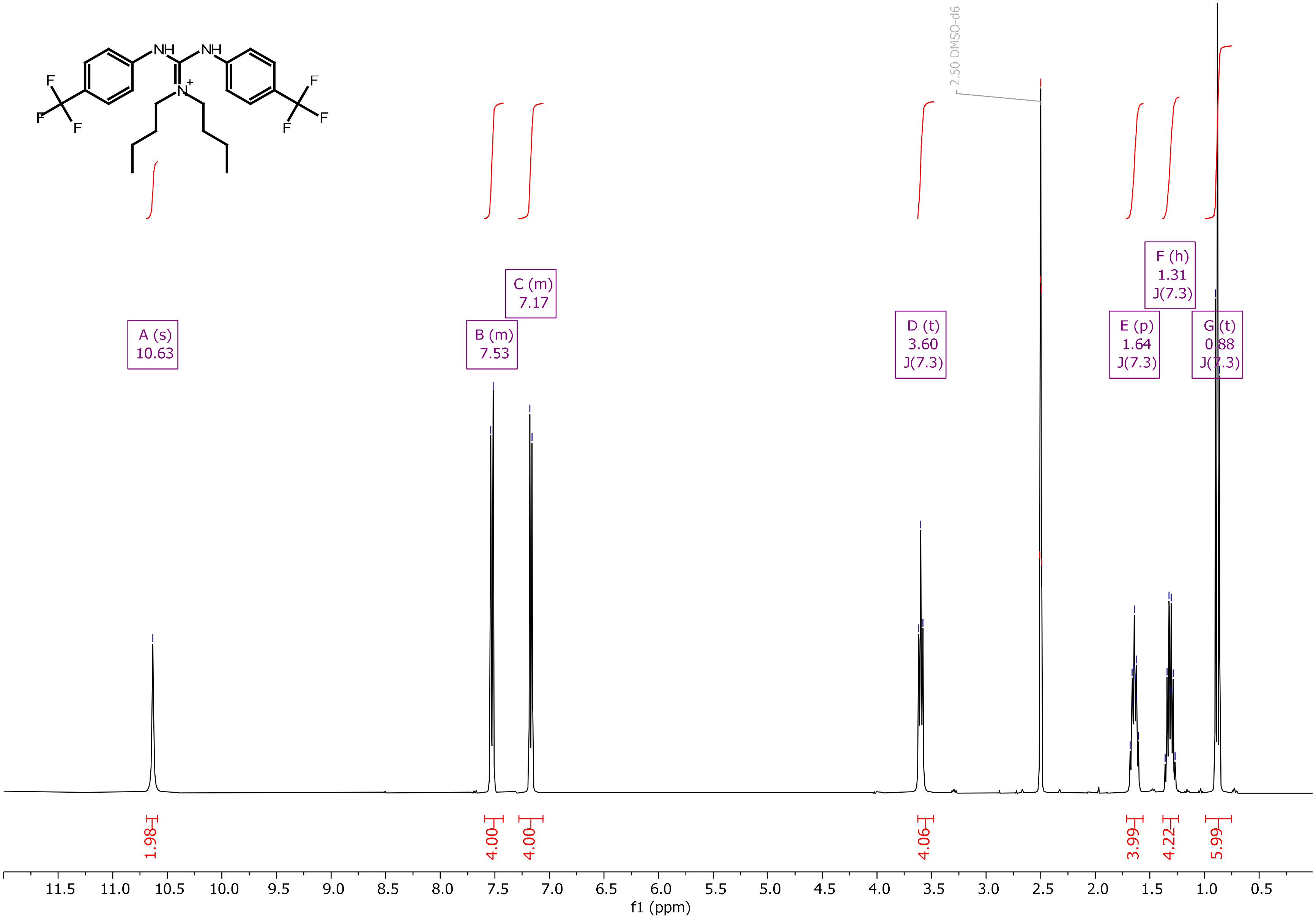

Figure S9. ^{1}H NMR (400 MHz) spectrum of **2e** DMSO-d_6 (with a few drops of TFA) at 298 K.

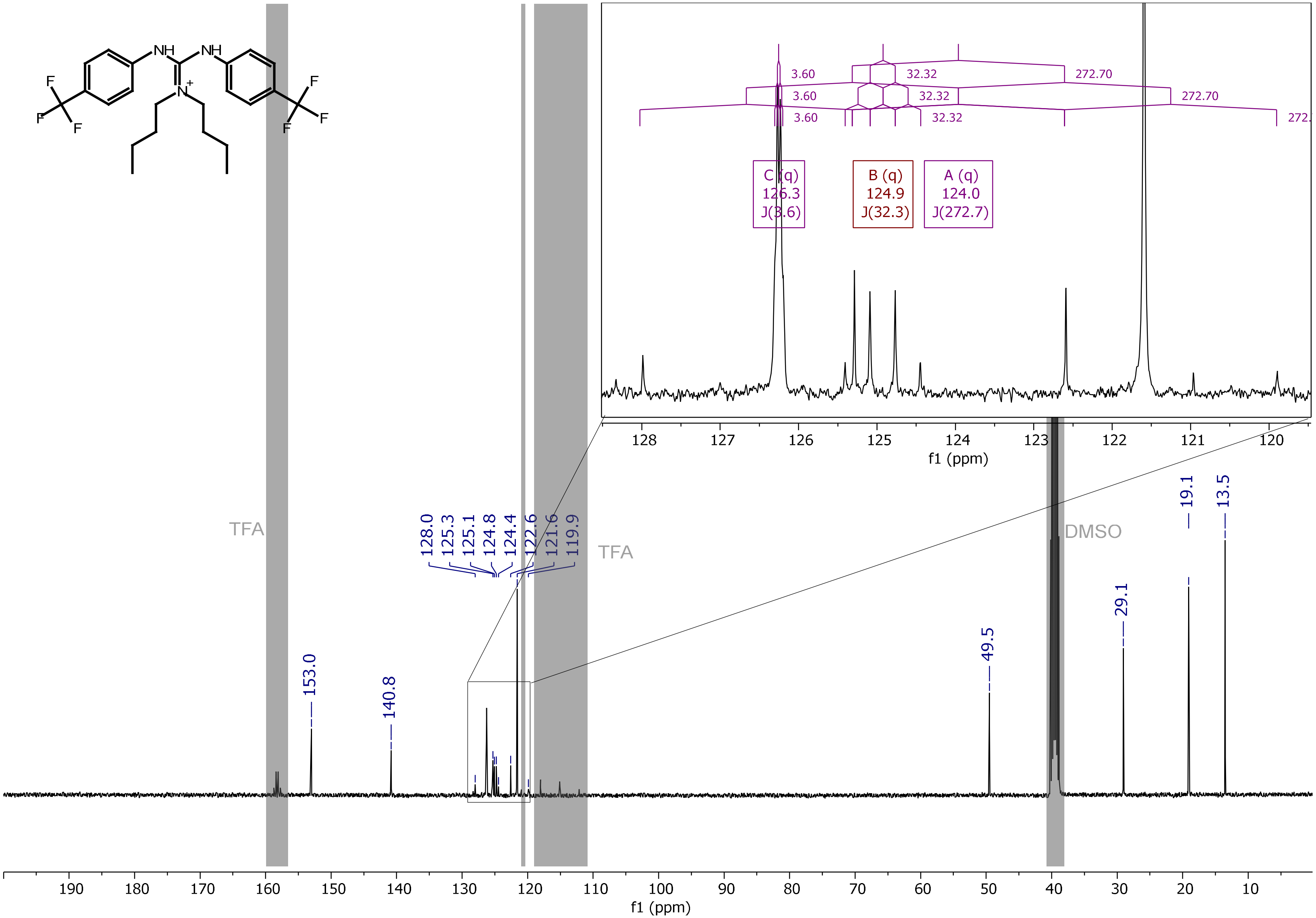

Figure S10. ^{13}C NMR (400 MHz) spectrum of **2e** DMSO-d_6 (with a few drops of TFA) at 298 K.

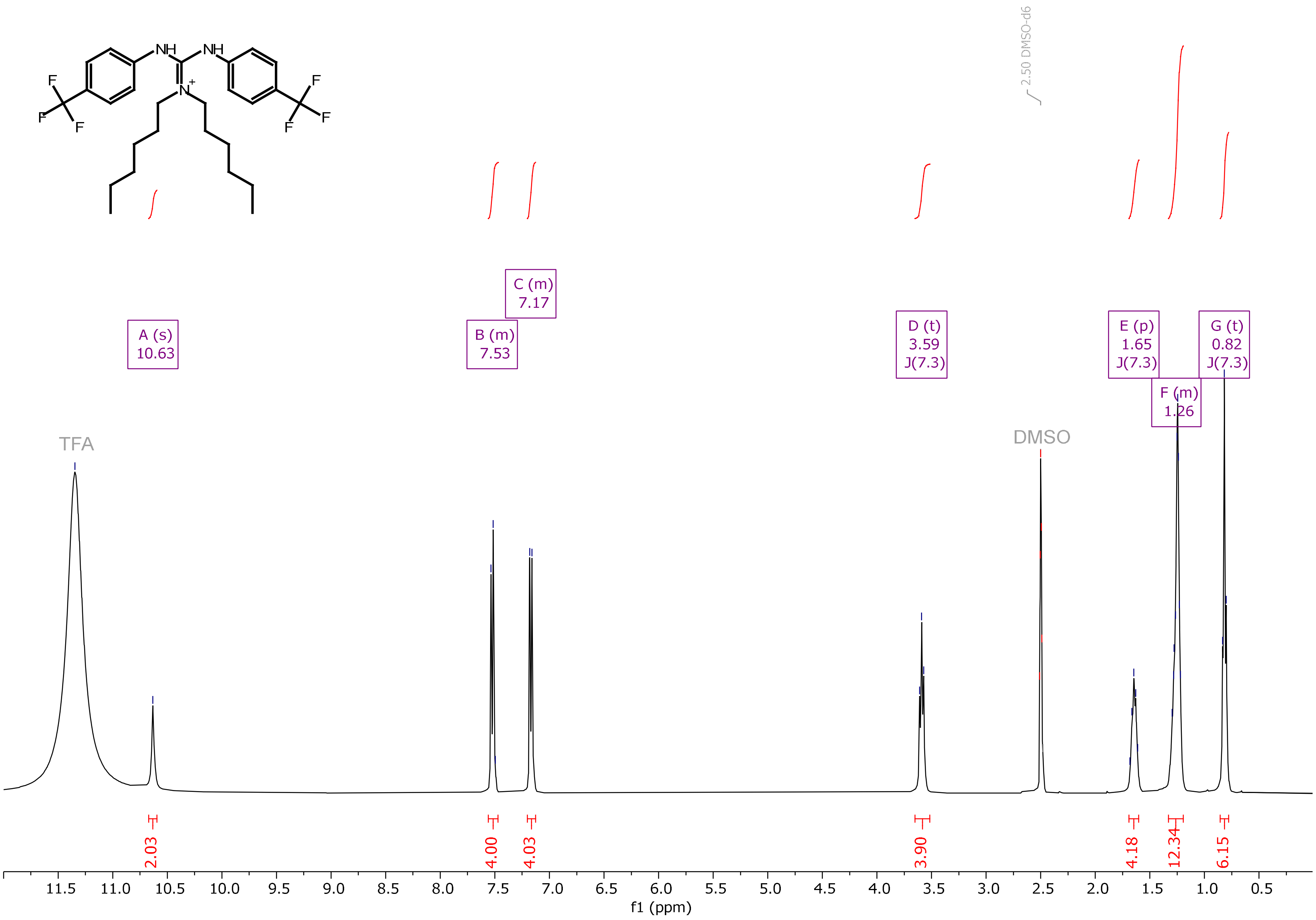

Figure S11. ^{1}H NMR (400 MHz) spectrum of **2f** DMSO-d_6 (with a few drops of TFA) at 298 K.

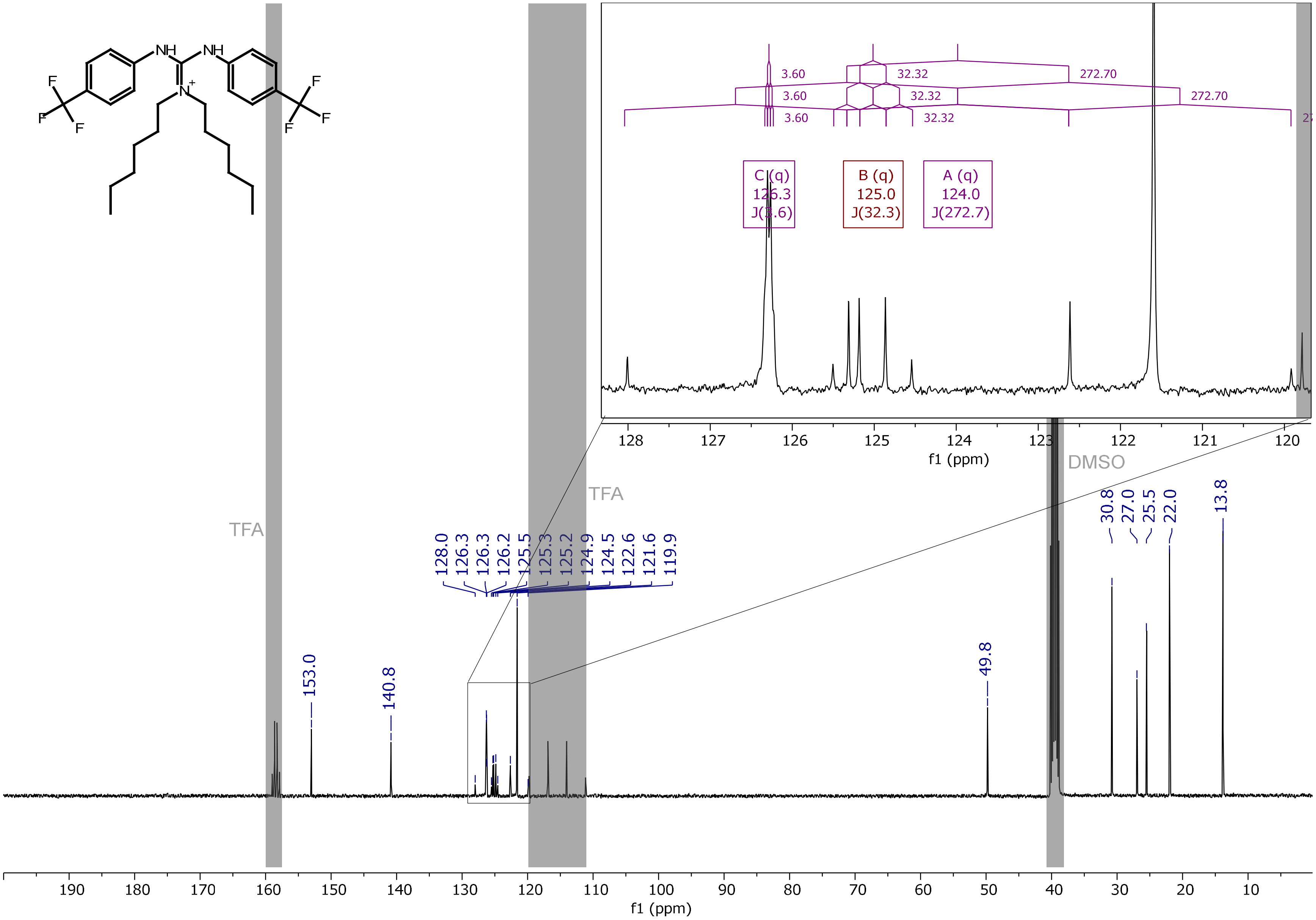

Figure S12. ^{13}C NMR (400 MHz) spectrum of **2f** DMSO-d_6 (with a few drops of TFA) at 298 K.

S3 pK_a Determination

To determine the pK_a, 100 µM of each transporter was dissolved in 1:1 acetonitrile:buffer (buffer = 100 mM NaH_2PO_4 as starting point). The initial pH was recorded, and 500 µL of the solution was transferred to a 1 cm pathlength microcuvette for UV–Vis absorbance measurements using an Agilent Cary 100 spectrophotometer. The pH was gradually increased by approximately 0.1 units via addition of NaOH, and absorbance spectra were recorded after each addition until the pH reached 11. The ratio of absorbance at two selected wavelengths was plotted against pH in OriginPro 2023b and fitted with a sigmoidal curve. The inflection point of the curve ($LOGx_0$) was taken as the pK_a value. All measurements were performed in triplicate to estimate experimental error. The results are shown in **Figure S13-Figure S20**. For compounds **1a, 1b,** and **2a**, pK_a values could not be determined because the compounds precipitated at high pHs.

To account for solvent effects, two reference compounds (**1c** and **1d**) were analyzed in solutions containing 10–50% acetonitrile (**Figure S21-Figure S25**). The extrapolated pK_a values in 0% acetonitrile (pure water) were 9.91 ± 0.02 and 10.23 ± 0.04 (**Figure S26**), while those measured in 50% acetonitrile were 9.25 ± 0.02 and 9.46 ± 0.02, respectively. This indicates that the pK_a of guanidines in water is 0.71 ± 0.08 units higher compared to the values measured in 50% acetonitrile. For all compounds, a value of 0.71 was therefore added to the measured pK_a values to estimate the pK_a in water. **Table S1** shows all experimental pK_a values in 50% acetonitrile, as well as the adjusted value for 100% water.

Table S1. Experimental pK_a values in 50% acetonitrile and the adjusted pK_a values for 100% water.

Compound	pK_a (50% MeCN)	pK_a (100% water)
**1a**	n.d.[a]	n.d.[a]
**1b**	n.d.[a]	n.d.[a]
**1c**	9.25 ± 0.02	9.96 ± 0.08
**1d**	9.46 ± 0.02	10.17 ± 0.08
**1e**	9.48 ± 0.02	10.19 ± 0.08
**1f**	8.78 ± 0.05	9.49 ± 0.09
**2a**	n.d.[a]	n.d.[a]
**2b**	n.d.[a]	n.d.[a]
**2c**	7.17 ± 0.04	7.88 ± 0.09
**2d**	7.29 ± 0.13	8.00 ± 0.15
**2e**	7.13 ± 0.13	7.84 ± 0.15
**2f**	7.13 ± 0.06	7.84 ± 0.10

[a] pK_a could not be determined (n.d.) due to the low solubility of the compound.

50% Acetonitrile

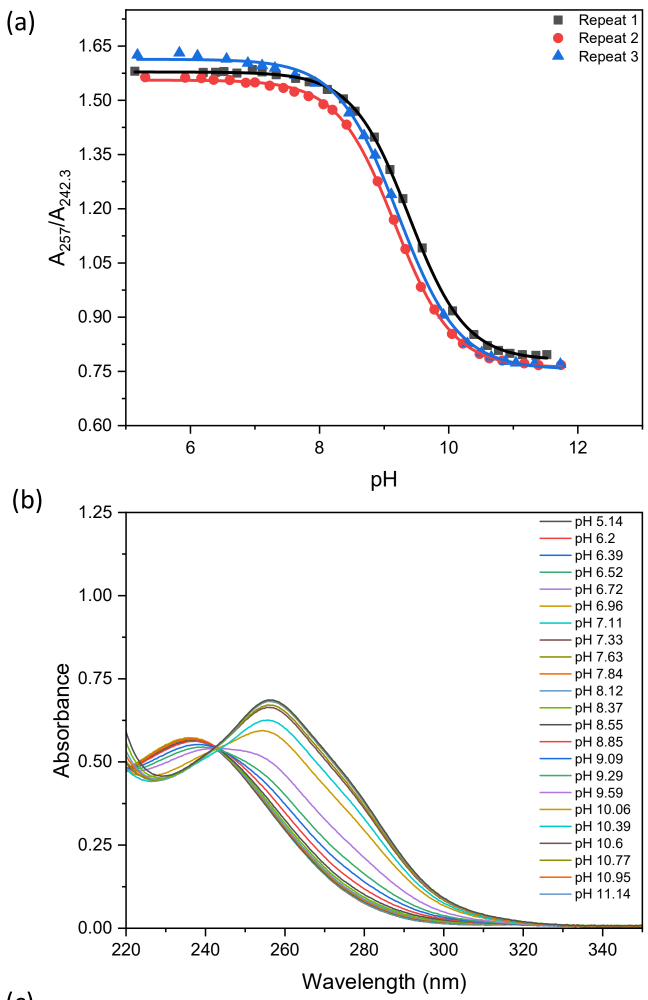

(c)

Model	DoseResp		
Equation	y = A1 + (A2-A1)/(1 + 10^((LOGx0-x)*p))		
Plot	Repeat 1	Repeat 2	Repeat 3
A1	0.781 ± 0.00263	0.761 ± 0.00234	0.756 ± 0.00577
A2	1.58 ± 0.00163	1.56 ± 0.00162	1.61 ± 0.00382
LOGx0	9.38 ± 0.00725	9.16 ± 0.00647	9.21 ± 0.0177
p	-0.989 ± 0.0147	-0.973 ± 0.0130	-0.924 ± 0.0297
Reduced Chi-Sqr	2.30E-5	2.21E-5	9.73E-5
R-Square (COD)	1.000	1.000	0.999
Adj. R-Square	1.000	1.000	0.999

Figure S13. pK_a determination of **1c** in 1:1 MeCN:buffer (100 mM phosphate buffer). The pH was adjusted with dilute NaOH. (a) Graph of pH vs $A_{257}/A_{242.3}$ for **1c**. The solid lines represent the fit of each graph using the DoseResp Model in OriginPro 2023b. Three independent repeats were conducted. (b) Representative example of the absorbance spectra of 100 µM **1c** in 1:1 MeCN:buffer (100 mM phosphate buffer) at various pH. (c) Overview of the parameters of the DoseResp fitting of each repeat. LOGx0 represents the pK_a in 1:1 MeCN:buffer.

(a)

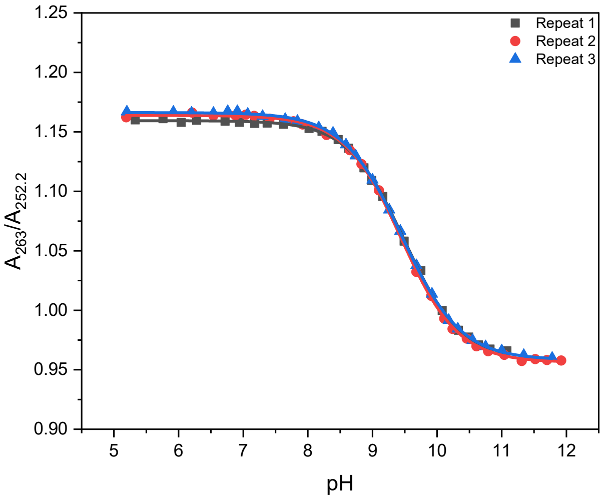

(b)

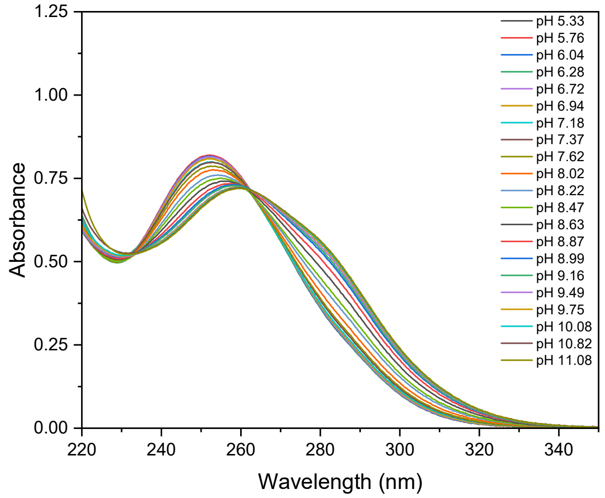

(c)

Model	DoseResp		
Equation	y = A1 + (A2-A1)/(1 + 10^((LOGx0-x)*p))		
Plot	Repeat 1	Repeat 2	Repeat 3
A1	0.959 ± 0.00116	0.956 ± 6.52E-4	0.958 ± 6.57E-4
A2	1.16 ± 4.63E-4	1.16 ± 4.95E-4	1.17 ± 3.58E-4
LOGx0	9.49 ± 0.00961	9.45 ± 0.00805	9.46 ± 0.00596
p	-0.998 ± 0.0173	-0.985 ± 0.0138	-0.955 ± 0.0108
Reduced Chi-Sqr	1.89E-6	1.86E-6	1.13E-6
R-Square (COD)	1.000	1.000	1.000
Adj. R-Square	1.000	1.000	1.000

Figure S14. pK_a determination of **1d** in 1:1 MeCN:buffer (100 mM phosphate buffer). The pH was adjusted with dilute NaOH. (a) Graph of pH vs $A_{263}/A_{252.2}$ for **1d**. The solid lines represent the fit of each graph using the DoseResp Model in OriginPro 2023b. Three independent repeats were conducted. (b) Representative example of the absorbance spectra of 100 µM **1d** in 1:1 MeCN:buffer (100 mM phosphate buffer) at various pH. (c) Overview of the parameters of the DoseResp fitting of each repeat. LOGx0 represents the pK_a in 1:1 MeCN:buffer.

(a)

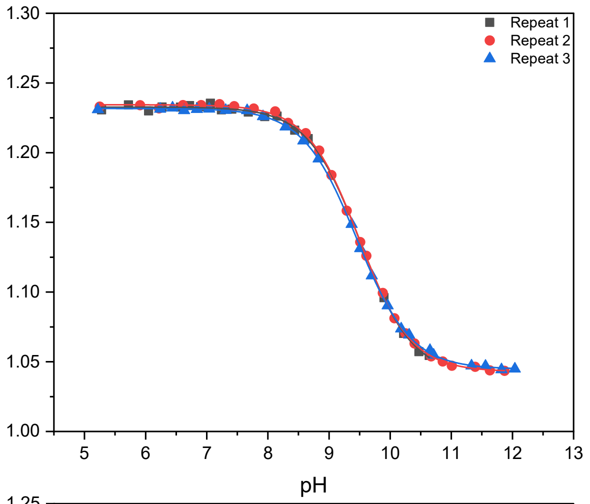

(b)

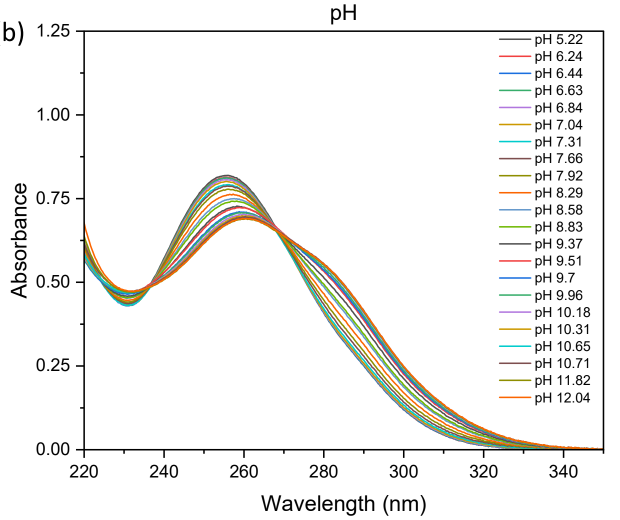

(c)

Model	DoseResp		
Equation	y = A1 + (A2-A1)/(1 + 10^((LOGx0-x)*p))		
Plot	Repeat 1	Repeat 2	Repeat 3
A1	1.04 ± 0.00273	1.04 ± 6.57E-4	1.04 ± 5.01E-4
A2	1.23 ± 5.49E-4	1.23 ± 4.59E-4	1.23 ± 3.56E-4
LOGx0	9.51 ± 0.0258	9.49 ± 0.00675	9.45 ± 0.00587
p	-1.01 ± 0.0331	-1.02 ± 0.0147	-0.984 ± 0.0120
Reduced Chi-Sqr	2.92E-6	1.68E-6	9.95E-7
R-Square (COD)	0.999	1.000	1.000
Adj. R-Square	0.999	1.000	1.000

Figure S15. pK_a determination of **1e** in 1:1 MeCN:buffer (100 mM phosphate buffer). The pH was adjusted with dilute NaOH. (a) Graph of pH vs A_{257}/A_{268} for **1e**. The solid lines represent the fit of each graph using the DoseResp Model in OriginPro 2023b. Three independent repeats were conducted. (b) Representative example of the absorbance spectra of 100 µM **1e** in 1:1 MeCN:buffer (100 mM phosphate buffer) at various pH. (c) Overview of the parameters of the DoseResp fitting of each repeat. LOGx0 represents the pK_a in 1:1 MeCN:buffer.

(a)

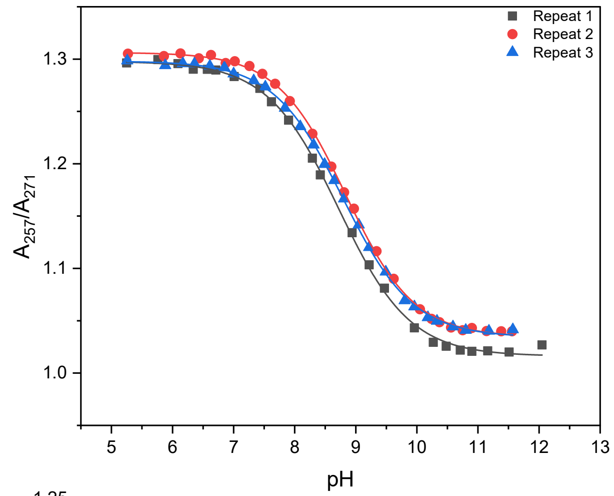

(b)

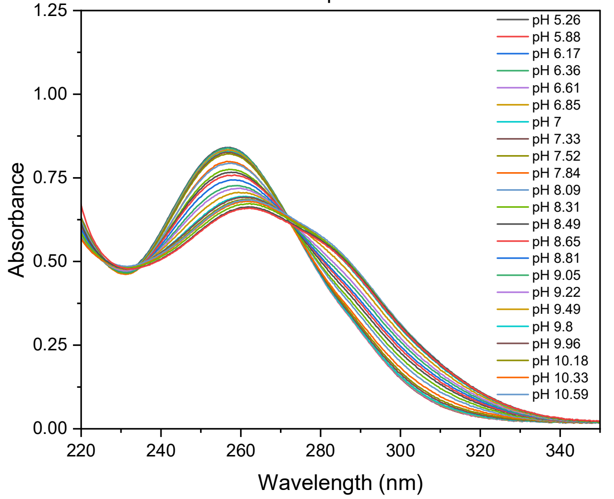

(c)

Model	DoseResp		
Equation	y = A1 + (A2-A1)/(1 + 10^((LOGx0-x)*p))		
Plot	Repeat 1	Repeat 2	Repeat 3
A1	1.02 ± 0.00179	1.03 ± 0.00123	1.03 ± 0.00142
A2	1.30 ± 0.00168	1.31 ± 0.00108	1.30 ± 0.00108
LOGx0	8.72 ± 0.0190	8.84 ± 0.0120	8.80 ± 0.0120
p	-0.743 ± 0.0222	-0.774 ± 0.0165	-0.757 ± 0.0157
Reduced Chi-Sqr	1.26E-5	5.78E-6	5.68E-6
R-Square (COD)	0.999	1.000	1.000
Adj. R-Square	0.999	1.000	0.999

Figure S16. pK_a determination of **1f** in 1:1 MeCN:buffer (100 mM phosphate buffer). The pH was adjusted with dilute NaOH. (a) Graph of pH vs A_{257}/A_{271} for **1f**. The solid lines represent the fit of each graph using the DoseResp Model in OriginPro 2023b. Three independent repeats were conducted. (b) Representative example of the absorbance spectra of 100 µM **1f** in 1:1 MeCN:buffer (100 mM phosphate buffer) at various pH. (c) Overview of the parameters of the DoseResp fitting of each repeat. LOGx0 represents the pK_a in 1:1 MeCN:buffer.

(a) (b)

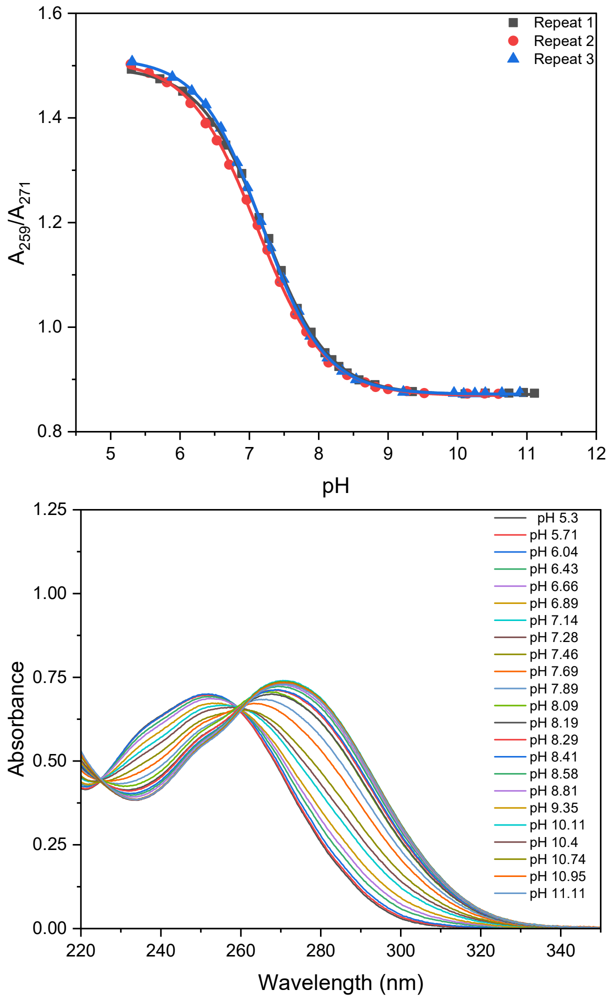

(c)

Model	DoseResp		
Equation	y = A1 + (A2-A1)/(1 + 10^((LOGx0-x)*p))		
Plot	Repeat 1	Repeat 2	Repeat 3
A1	0.871 ± 0.00147	0.869 ± 0.00169	0.872 ± 9.07E-4
A2	1.50 ± 0.00301	1.51 ± 0.00339	1.51 ± 0.00208
LOGx0	7.22 ± 0.00778	7.11 ± 0.00849	7.19 ± 0.00480
p	-0.937 ± 0.0130	-0.888 ± 0.0136	-0.959 ± 0.00862
Reduced Chi-Sqr	1.43E-5	1.70E-5	5.84E-6
R-Square (COD)	1.000	1.000	1.000
Adj. R-Square	1.000	1.000	1.000

Figure S17. pK_a determination of **2c** in 1:1 MeCN:buffer (100 mM phosphate buffer). The pH was adjusted with dilute NaOH. (a) Graph of pH vs A_{259}/A_{271} for **2c**. The solid lines represent the fit of each graph using the DoseResp Model in OriginPro 2023b. Three independent repeats were conducted. (b) Representative example of the absorbance spectra of 100 µM **2c** in 1:1 MeCN:buffer (100 mM phosphate buffer) at various pH. (c) Overview of the parameters of the DoseResp fitting of each repeat. LOGx0 represents the pK_a in 1:1 MeCN:buffer.

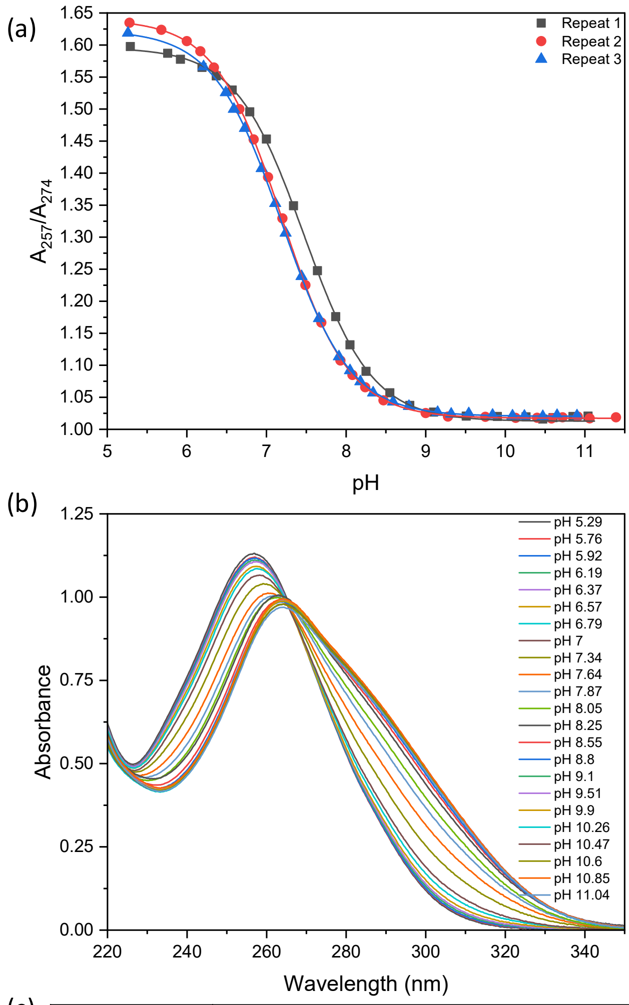

(c)

Model	DoseResp		
Equation	y = A1 + (A2-A1)/(1 + 10^((LOGx0-x)*p))		
Plot	Repeat 1	Repeat 2	Repeat 3
A1	1.01 ± 0.00299	1.02 ± 5.27E-4	1.02 ± 5.32E-4
A2	1.60 ± 0.00542	1.64 ± 0.00117	1.62 ± 0.00153
LOGx0	7.47 ± 0.0180	7.20 ± 0.00305	7.19 ± 0.00344
p	-1.01 ± 0.0350	-1.03 ± 0.00613	-1.01 ± 0.00620
Reduced Chi-Sqr	7.77E-5	2.74E-6	2.66E-6
R-Square (COD)	0.999	1.000	1.000
Adj. R-Square	0.999	1.000	1.000

Figure S18. pK_a determination of **2d** in 1:1 MeCN:buffer (100 mM phosphate buffer). The pH was adjusted with dilute NaOH. (a) Graph of pH vs A_{257}/A_{274} for **2d**. The solid lines represent the fit of each graph using the DoseResp Model in OriginPro 2023b. Three independent repeats were conducted. (b) Representative example of the absorbance spectra of 100 µM **2d** in 1:1 MeCN:buffer (100 mM phosphate buffer) at various pH. (c) Overview of the parameters of the DoseResp fitting of each repeat. LOGx0 represents the pK_a in 1:1 MeCN:buffer.

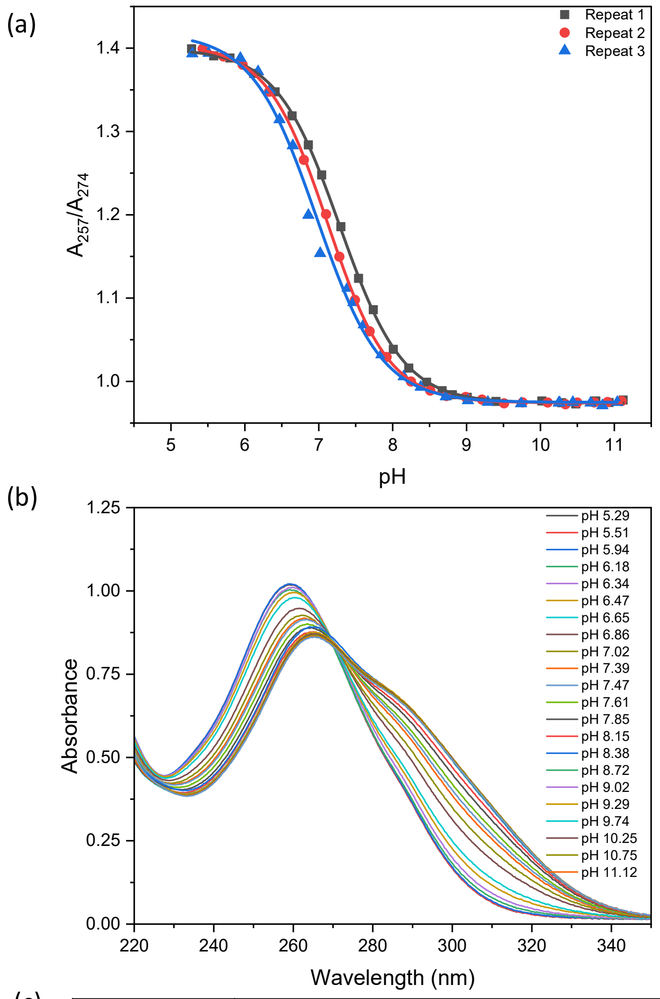

(c)

Model	DoseResp		
Equation	y = A1 + (A2-A1)/(1 + 10^((LOGx0-x)*p))		
Plot	Repeat 1	Repeat 2	Repeat 3
A1	0.974 ± 7.85E-4	0.974 ± 6.10E-4	0.975 ± 0.00387
A2	1.40 ± 0.00147	1.40 ± 0.00156	1.42 ± 0.0100
LOGx0	7.28 ± 0.00608	7.12 ± 0.00578	6.97 ± 0.0343
p	-1.01 ± 0.0123	-1.05 ± 0.0130	-0.987 ± 0.0610
Reduced Chi-Sqr	5.19E-6	3.87E-6	1.46E-4
R-Square (COD)	1.000	1.000	0.995
Adj. R-Square	1.000	1.000	0.995

Figure S19. pK_a determination of **2e** in 1:1 MeCN:buffer (100 mM phosphate buffer). The pH was adjusted with dilute NaOH. (a) Graph of pH vs A_{257}/A_{274} for **2e**. The solid lines represent the fit of each graph using the DoseResp Model in OriginPro 2023b. Three independent repeats were conducted. (b) Representative example of the absorbance spectra of 100 µM **2e** in 1:1 MeCN:buffer (100 mM phosphate buffer) at various pH. (c) Overview of the parameters of the DoseResp fitting of each repeat. LOGx0 represents the pK_a in 1:1 MeCN:buffer.

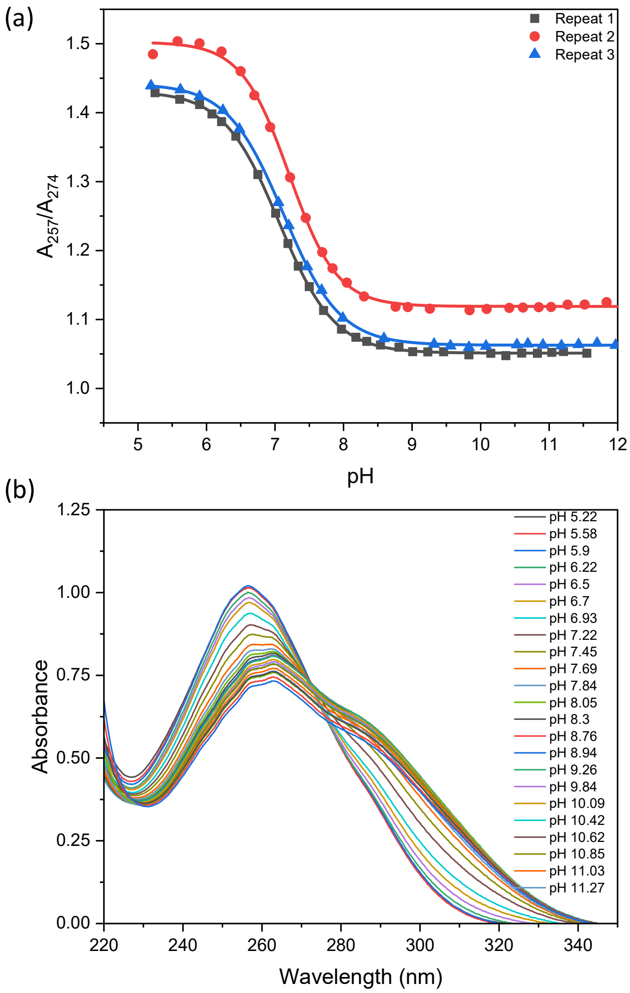

(c)

Model	DoseResp		
Equation	y = A1 + (A2-A1)/(1 + 10^((LOGx0-x)*p))		
Plot	Repeat 1	Repeat 2	Repeat 3
A1	1.05 ± 5.07E-4	1.12 ± 0.00167	1.06 ± 4.76E-4
A2	1.43 ± 0.00128	1.50 ± 0.00321	1.44 ± 0.00124
LOGx0	7.06 ± 0.00529	7.21 ± 0.0135	7.13 ± 0.00561
p	-1.07 ± 0.0119	-1.22 ± 0.0380	-1.06 ± 0.0131
Reduced Chi-Sqr	3.15E-6	2.77E-5	2.79E-6
R-Square (COD)	1.000	0.999	1.000
Adj. R-Square	1.000	0.999	1.000

Figure S20. pK_a determination of **2f** in 1:1 MeCN:buffer (100 mM phosphate buffer). The pH was adjusted with dilute NaOH. (a) Graph of pH vs A_{257}/A_{274} for **2f**. The solid lines represent the fit of each graph using the DoseResp Model in OriginPro 2023b. Three independent repeats were conducted. (b) Representative example of the absorbance spectra of 100 µM **2f** in 1:1 MeCN:buffer (100 mM phosphate buffer) at various pH. (c) Overview of the parameters of the DoseResp fitting of each repeat. LOGx0 represents the pK_a in 1:1 MeCN:buffer.

Various Acetonitrile Concentrations

(a)

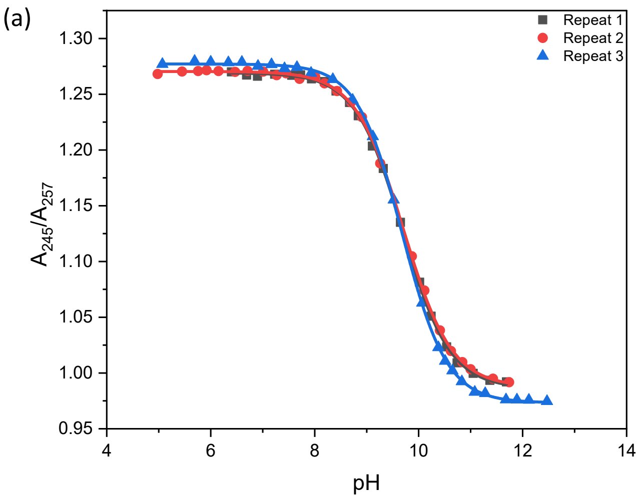

(b)

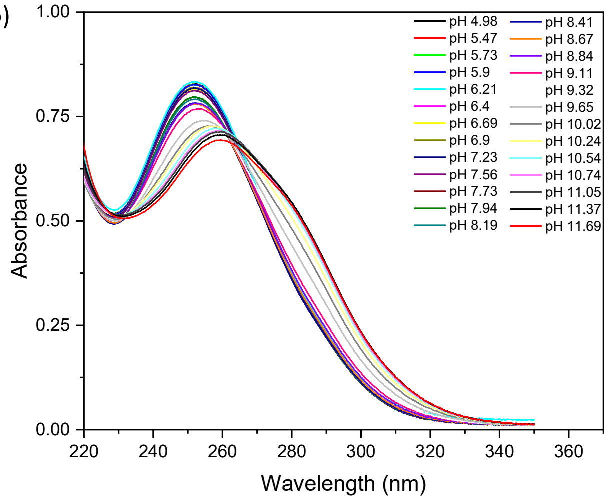

(c)

Model	DoseResp		
Equation	y = A1 + (A2-A1)/(1 + 10^((LOGx0-x)*p))		
Plot	Repeat 1	Repeat 2	Repeat 3
A1	0.986 ± 0.00137	0.987 ± 0.00130	0.973 ± 7.62E-4
A2	1.27 ± 7.44E-4	1.27 ± 5.40E-4	1.28 ± 5.27E-4
LOGx0	9.69 ± 0.00903	9.71 ± 0.00934	9.68 ± 0.00687
p	-0.953 ± 0.0157	-0.935 ± 0.0142	-1.01 ± 0.0123
Reduced Chi-Sqr	3.51E-6	3.20E-6	2.67E-6
R-Square (COD)	1.000	1.000	1.000
Adj. R-Square	1.000	1.000	1.000

Figure S21. pK_a determination of **1d** in 35% MeCN in buffer (100 mM phosphate buffer). The pH was adjusted with dilute NaOH. (a) Graph of pH vs A_{245}/A_{257} for **1d**. The solid lines represent the fit of each graph using the DoseResp Model in OriginPro 2023b. Three independent repeats were conducted. (b) Representative example of the absorbance spectra of 100 µM **1d** in 30% MeCN in buffer (100 mM phosphate buffer) at various pH. (c) Overview of the parameters of the DoseResp fitting of each repeat. LOGx0 represents the pK_a in 1:1 MeCN:buffer.

(a)

(b)

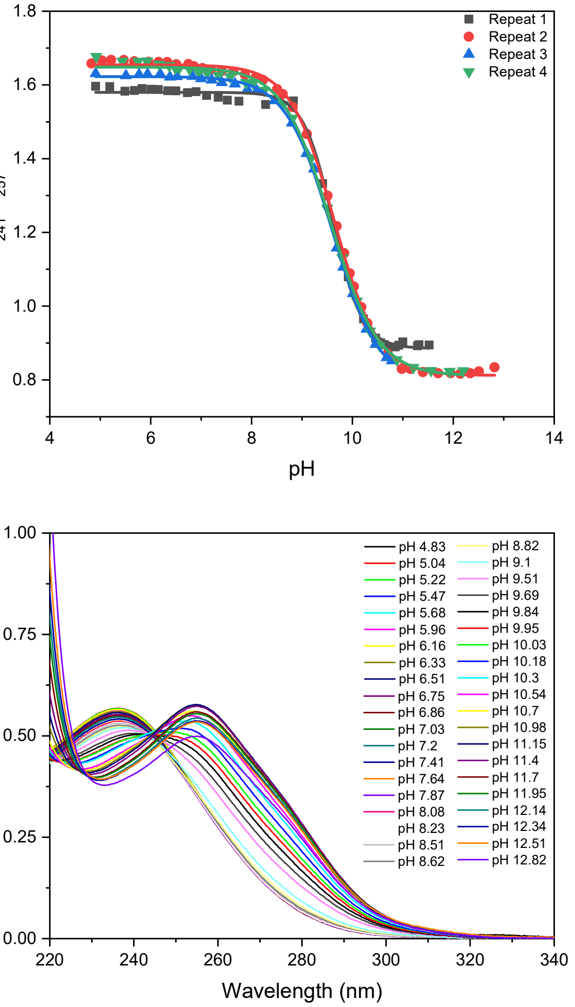

(c)

Model	DoseResp			
Equation	y = A1 + (A2-A1)/(1 + 10^((LOGx0-x)*p))			
Plot	Repeat 1	Repeat 2	Repeat 3	Repeat 4
A1	0.885 ± 0.00534	0.812 ± 0.00338	0.785 ± 0.00849	0.811 ± 0.00662
A2	1.58 ± 0.00324	1.65 ± 0.00231	1.62 ± 0.00189	1.65 ± 0.00341
LOGx0	9.61 ± 0.0192	9.64 ± 0.00964	9.60 ± 0.0140	9.56 ± 0.0178
p	-1.48 ± 0.0803	-1.00 ± 0.0203	-0.937 ± 0.0213	-0.922 ± 0.0295
Reduced Chi-Sqr	1.67E-4	8.57E-5	4.07E-5	1.50E-4
R-Square (COD)	0.999	0.999	1.000	0.999
Adj. R-Square	0.998	0.999	1.000	0.999

Figure S22. pK_a determination of **1c** in 25% MeCN in buffer (100 mM phosphate buffer). The pH was adjusted with dilute NaOH. (a) Graph pH vs A_{241}/A_{257} for **1c**. The solid lines represent the fit of each graph using the DoseResp Model in OriginPro 2023b. Three independent repeats were conducted. (b) Representative example of the absorbance spectra of 100 µM **1c** in 25% MeCN in buffer (100 mM phosphate buffer) at various pH. (c) Overview of the parameters of the DoseResp fitting of each repeat. LOGx0 represents the pK_a in 1:1 MeCN:buffer.

(a)

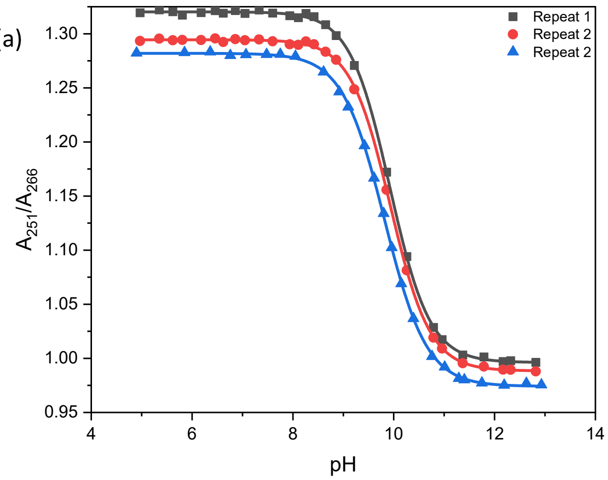

(b)

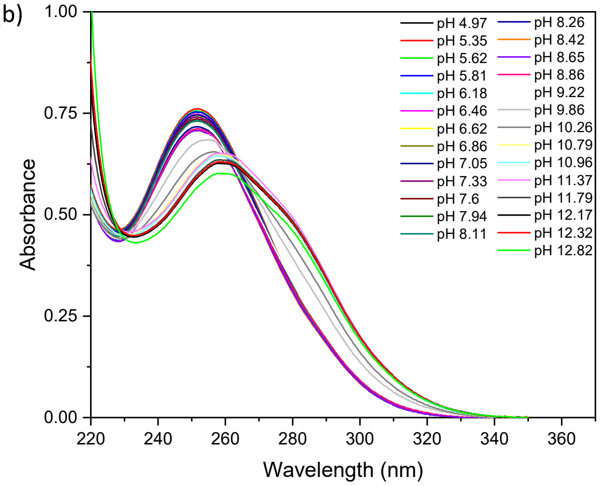

(c)

Model	DoseResp		
Equation	y = A1 + (A2-A1)/(1 + 10^((LOGx0-x)*p))		
Plot	Repeat 1	Repeat 2	Repeat 3
A1	0.996 ± 8.15E-4	0.988 ± 6.30E-4	0.974 ± 6.91E-4
A2	1.32 ± 4.64E-4	1.29 ± 3.58E-4	1.28 ± 5.63E-4
LOGx0	9.92 ± 0.00665	9.93 ± 0.00541	9.82 ± 0.00488
p	-1.09 ± 0.0154	-1.10 ± 0.0127	-1.03 ± 0.0115
Reduced Chi-Sqr	2.96E-6	1.77E-6	2.48E-6
R-Square (COD)	1.000	1.000	1.000
Adj. R-Square	1.000	1.000	1.000

Figure S23. pK_a determination of **1d** in 25% MeCN in buffer (100 mM phosphate buffer). The pH was adjusted with dilute NaOH. (a) Graph pH vs A_{251}/A_{266} for **1d**. The solid lines represent the fit of each graph using the DoseResp Model in OriginPro 2023b. Three independent repeats were conducted. (b) Representative example of the absorbance spectra of 100 µM **1d** in 25% MeCN in buffer (100 mM phosphate buffer) at various pH. (c) Overview of the parameters of the DoseResp fitting of each repeat. LOGx0 represents the pK_a in 1:1 MeCN:buffer.

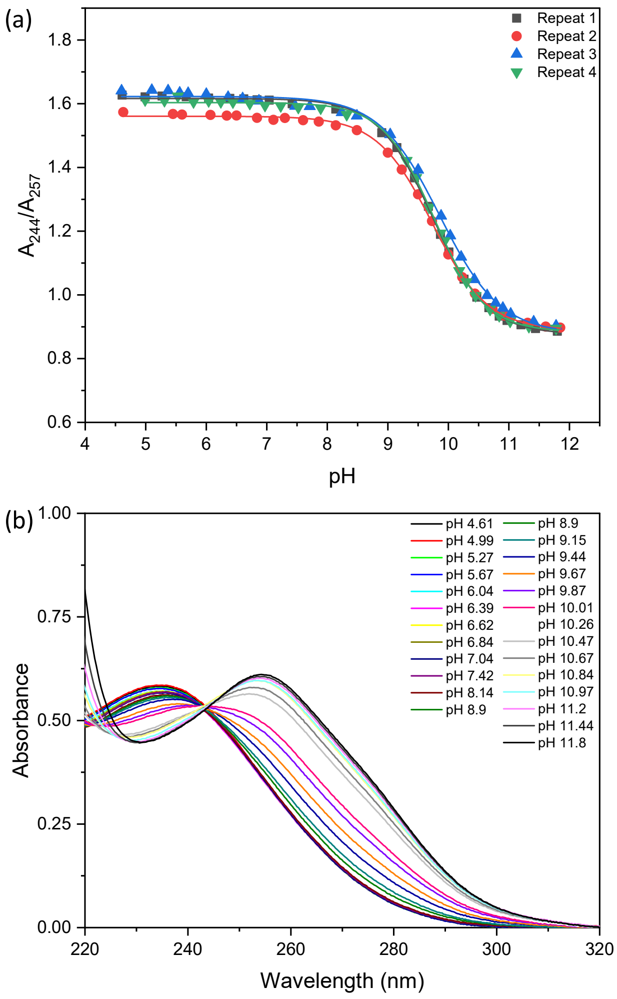

(c)

Model	DoseResp			
Equation	y = A1 + (A2-A1)/(1 + 10^((LOGx0-x)*p))			
Plot	Repeat 1	Repeat 2	Repeat 3	Repeat 4
A1	0.877 ± 0.00381	0.889 ± 0.00367	0.878 ± 0.0115	0.884 ± 0.00528
A2	1.62 ± 0.00180	1.56 ± 0.00171	1.62 ± 0.00409	1.60 ± 0.00216
LOGx0	9.73 ± 0.00897	9.74 ± 0.0101	9.85 ± 0.0261	9.76 ± 0.0108
p	-0.966 ± 0.0183	-0.950 ± 0.0189	-0.868 ± 0.0418	-1.03 ± 0.0282
Reduced Chi-Sqr	3.36E-5	3.14E-5	1.81E-4	5.61E-5
R-Square (COD)	1.000	1.000	0.998	0.999
Adj. R-Square	1.000	1.000	0.998	0.999

Figure S24. pK_a determination of **1c** in 10% MeCN in buffer (100 mM phosphate buffer). The pH was adjusted with dilute NaOH. (a) Graph pH vs A_{244}/A_{257} for **1c**. The solid lines represent the fit of each graph using the DoseResp Model in OriginPro 2023b. Three independent repeats were conducted. (b) Representative example of the absorbance spectra of 100 µM **1c** in 10% MeCN in buffer (100 mM phosphate buffer) at various pH. (c) Overview of the parameters of the DoseResp fitting of each repeat. LOGx0 represents the pK_a in 1:1 MeCN:buffer.

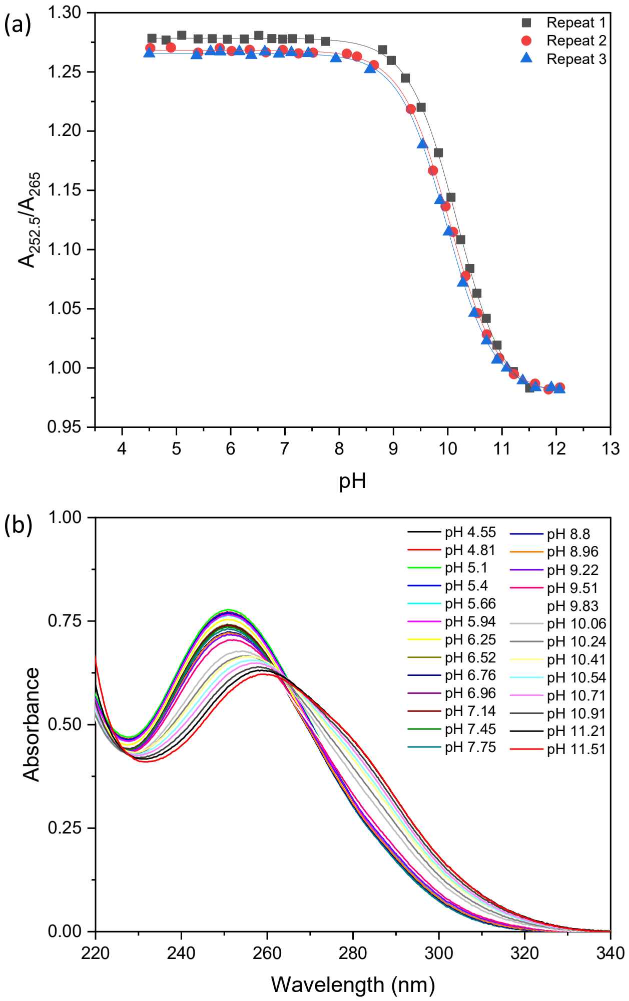

(c)

Model	DoseResp		
Equation	y = A1 + (A2-A1)/(1 + 10^((LOGx0-x)*p))		
Plot	Repeat 1	Repeat 2	Repeat 3
A1	0.970 ± 0.00186	0.978 ± 0.00123	0.979 ± 8.76E-4
A2	1.28 ± 4.39E-4	1.27 ± 5.50E-4	1.27 ± 4.28E-4
LOGx0	10.2 ± 0.00784	10.0 ± 0.00723	9.97 ± 0.00553
p	-0.977 ± 0.0136	-0.973 ± 0.0159	-0.999 ± 0.0138
Reduced Chi-Sqr	2.55E-6	3.46E-6	2.04E-6
R-Square (COD)	1.000	1.000	1.000
Adj. R-Square	1.000	1.000	1.000

Figure S25. pK_a determination of **1d** in 10% MeCN in buffer (100 mM phosphate buffer). The pH was adjusted with dilute NaOH. (a) Graph pH vs $A_{252.5}/A_{266}$ for **1d**. The solid lines represent the fit of each graph using the DoseResp Model in OriginPro 2023b. Three independent repeats were conducted. (b) Representative example of the absorbance spectra of 100 µM **1d** in 10% MeCN in buffer (100 mM phosphate buffer) at various pH. (c) Overview of the parameters of the DoseResp fitting of each repeat. LOGx0 represents the pK_a in 1:1 MeCN:buffer.

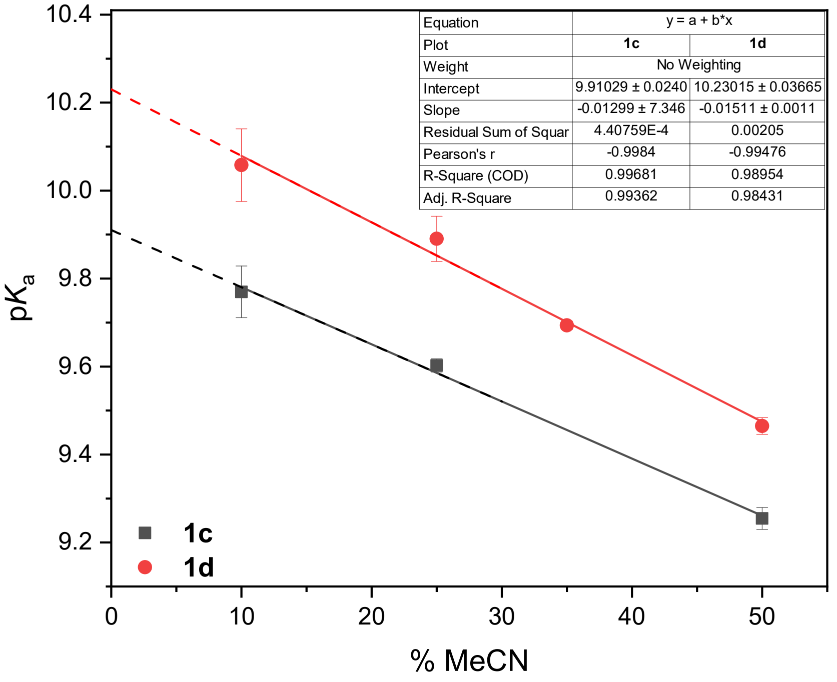

Figure S26. Linear fit of the pK_a values versus acetonitrile content (% MeCN) for compounds **1c** and **1d**. The pK_a values were measured in the previous figures and are the result of 3 repeats. The intercept of the linear fit corresponds to the pK_a value predicted for pure water.

S4 ^{1}H NMR Titrations

^{1}H NMR titrations were performed using a Bruker 400 or 600 MHz instrument, using acetonitrile-d_3 as the solvent. For all titrations, the transporter was the host and tetrabutylammonium chloride (TBACl) was the guest. Titrations were performed using a host concentration of 2.5 mM for compounds **1c** and **2c** and 5 mM for compounds **1d**, **1e**, **1f**, **2d**, **2e**, and **2f** as the starting point. To this host solution, aliquots of a solution containing TBACl (concentration varied in each experiment) and host were added using a Hamilton gas-tight syringe (this procedure ensures that the host concentration remains constant throughout the titration) through a rubber septum. The ^{1}H NMR spectrum was obtained upon each addition. The instrument was locked to acetonitrile-d_3 and the solvent peak referenced to δ = 1.94 ppm. The downfield shift of the various aromatic CH and NH peaks were determined using MestreNova, and these values were used to calculate association constants (K_a) using the online tool BindFit using a 1:1 model. The results are shown in **Figure S27-Figure S34**. While some residual plots showed some systematic error, other models such as 1:2 and 2:1 host:guest binding gave worse errors or unrealistic values. For compounds **1d-1f**, the NH peaks were not resolved, and only the aromatic peaks were used for the fitting. For the same 3 compounds the obtained binding constants were very low.

To evaluate the chloride binding affinity of the guanidine receptors in their protonated form, the host was first protonated by the addition of 2 equivalents HPF_6 (55 wt% in H_2O). HPF_6 is a strong, non-coordinating acid that can protonate the host molecules without introducing strongly competing anions. Two equivalents were added because the addition of only 1 equivalent did not seem to fully protonate the guanidines (potentially due to degradation and evaporation of HPF_6 from the commercial aqueous stock). The resulting solutions were lyophilized (>10 h) to remove residual water and obtain the protonated host prior to the titrations. Subsequently, ^{1}H NMR titrations were carried out as described above, using a 2.5 mM stock of protonated host as a starting point. Only the data for compound **2d** could be fitted satisfactory (**Figure S38**). The data for compound **1c** (**Figure S35**), **1d** (**Figure S36**) and **2c** (**Figure S37**) clearly show stronger chloride binding by the protonated guanidiniums compared to the neutral guanidines, but no good fit was obtained (presumably because the excess HPF_6 or the HPF_6 degradation products interfered with the titrations).

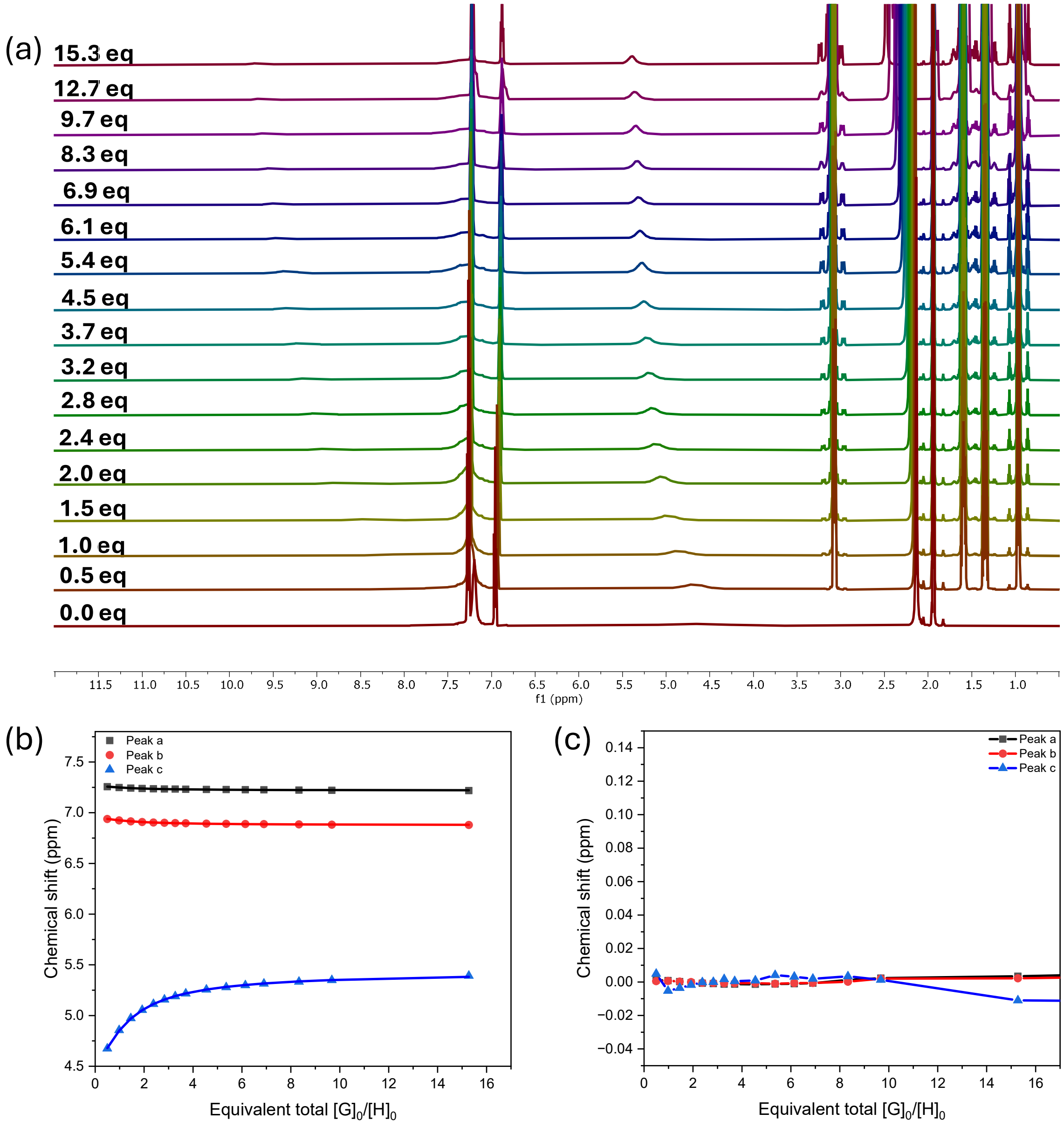

Figure S27. ^{1}H NMR titration of transporter **1c** (host, 2.5 mM) with TBACl (guest) in CD_3CN. (a) Stack plot of a representative titration. (b) Fitplot for the guanidine NH and phenyl CH peaks using global analysis and 1:1 binding stoichiometry. K_a = 245.11 M^{-1} (error = 1.0 %). (c) Plot of the residuals for the non-linear fit using global analysis and 1:1 binding stoichiometry.

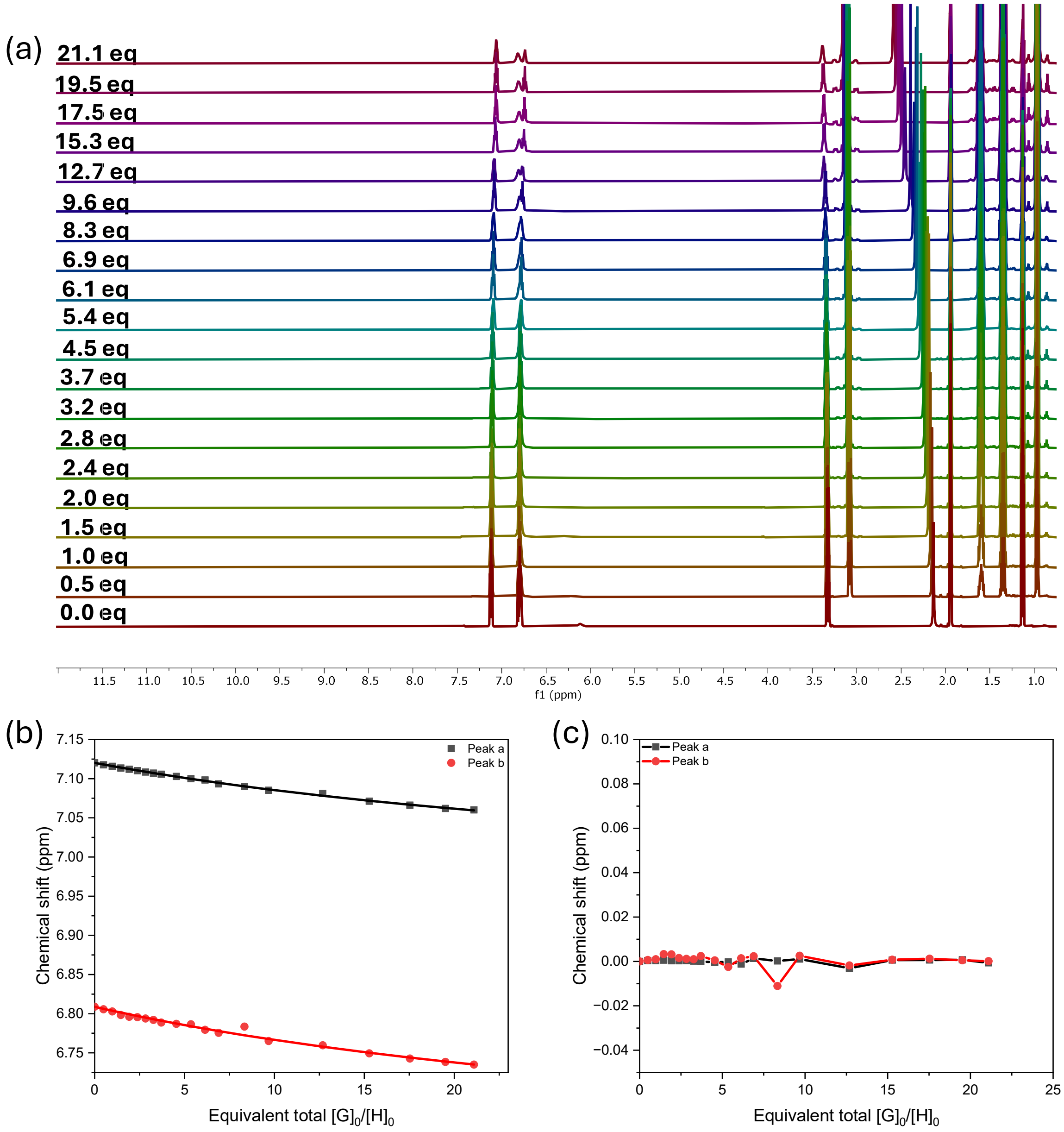

Figure S28. ^{1}H NMR titration of transporter **1d** (host, 5 mM) with TBACl (guest) in CD_3CN. (a) Stack plot of a representative titration. (b) Fit plot for the guanidine phenyl CH peaks using global analysis and 1:1 binding stoichiometry. K_a = 4.8 M^{-1} (error = 3.0 %). The guanidinium NH peaks were excluded from the fitting due to significant broadening during the titration (c) Plot of the residuals for the non-linear fit using global analysis and 1:1 binding stoichiometry.

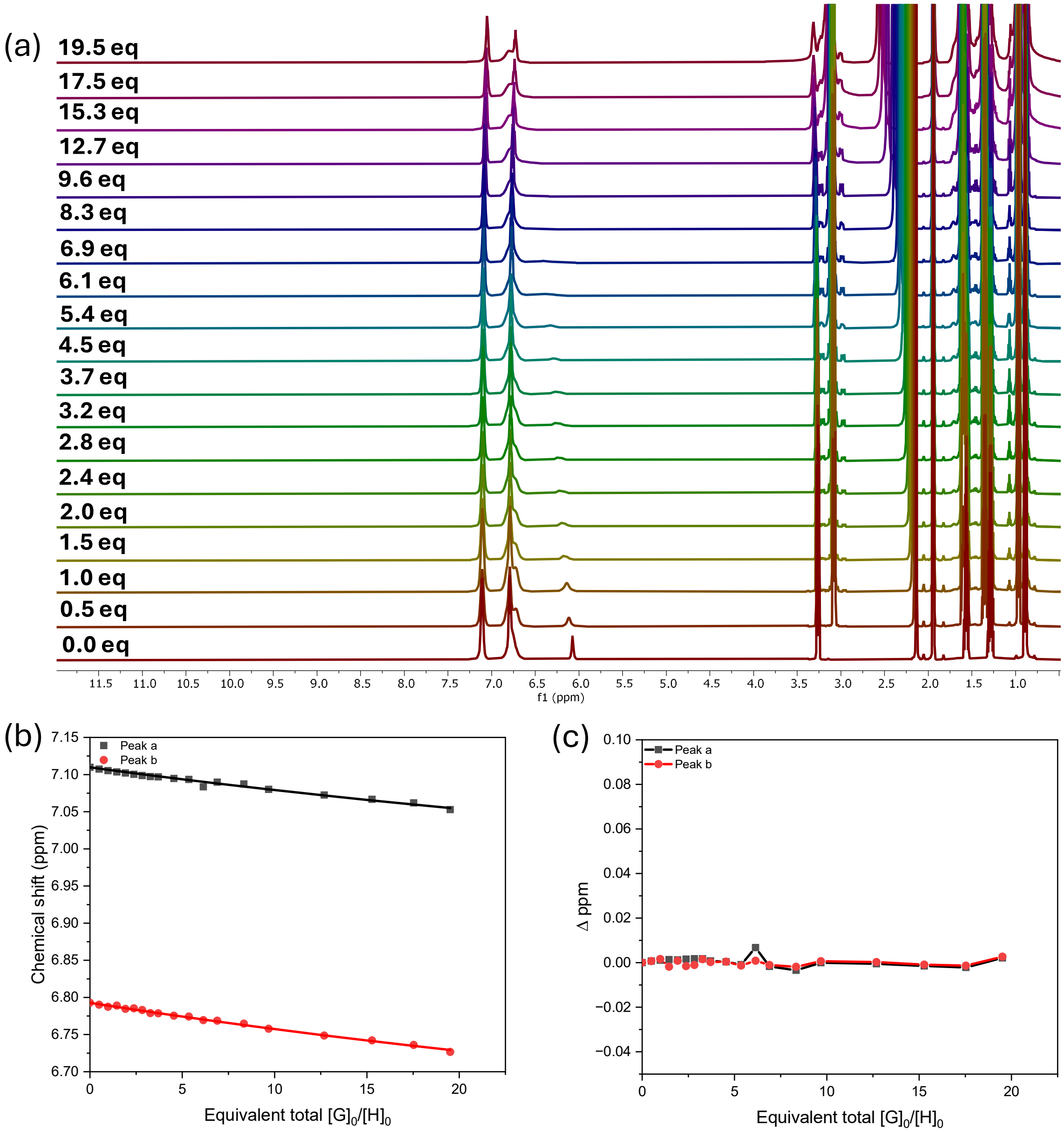

Figure S29. ^{1}H NMR titration of transporter **1e** (host, 5 mM) with TBACl (guest) in CD_3CN. (a) Stack plot of a representative titration. (b) Fit plot for the guanidine phenyl CH peaks using global analysis and 1:1 binding stoichiometry. K_a = 2.1 M^{-1} (error = 2.6%). (c) Plot of the residuals for the non-linear fit using global analysis and 1:1 binding stoichiometry.

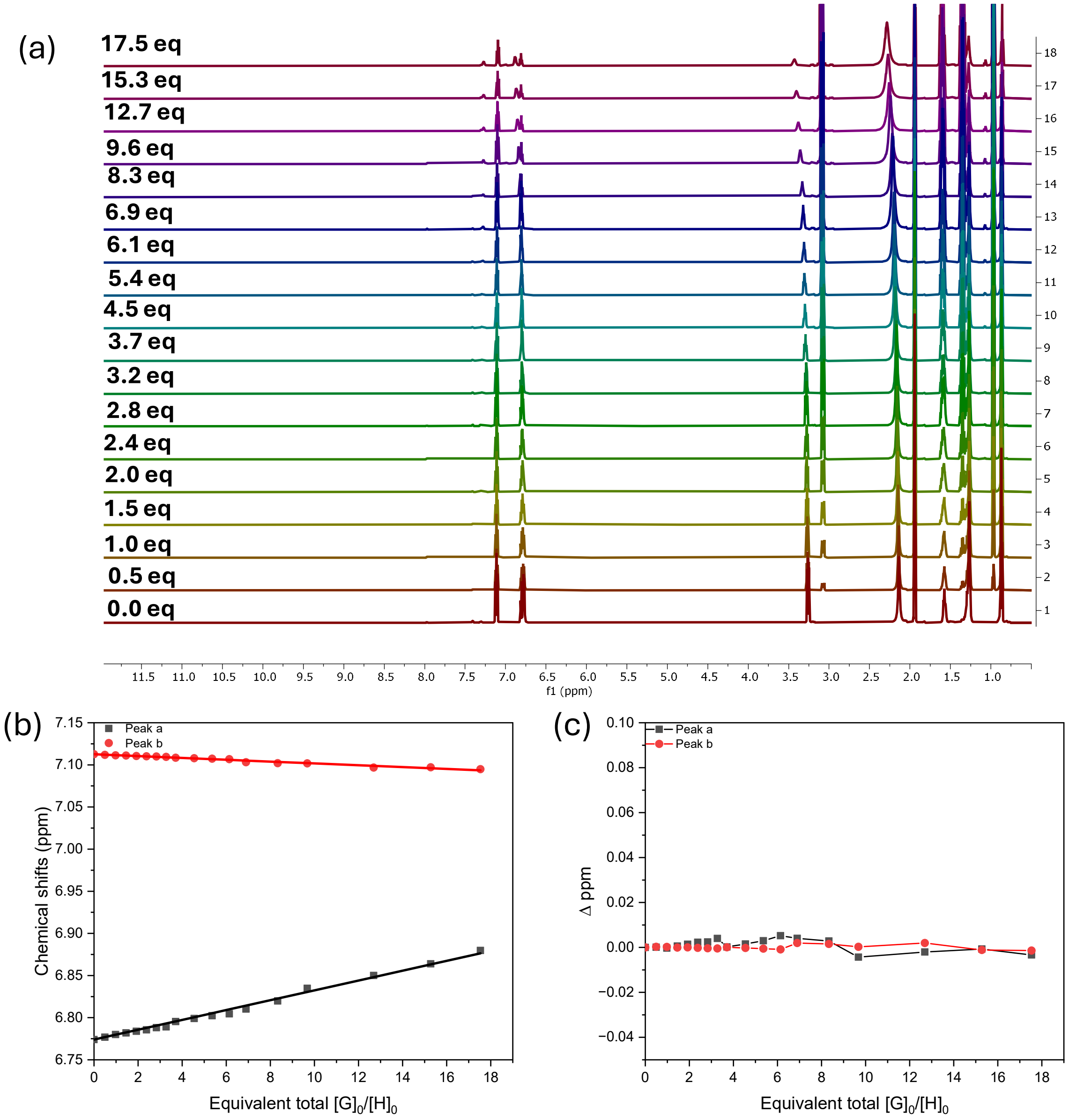

Figure S30. ^{1}H NMR titration of transporter **1f** (host, 5 mM) with TBACl (guest) in CD_3CN. (a) Stack plot of a representative titration. (b) Fit plot for the guanidine NH and phenyl CH peaks using global analysis and 1:1 binding stoichiometry. $K_a < 1\ M^{-1}$ (very low binding was seen). (c) Plot of the residuals for the non-linear fit using global analysis and 1:1 binding stoichiometry.

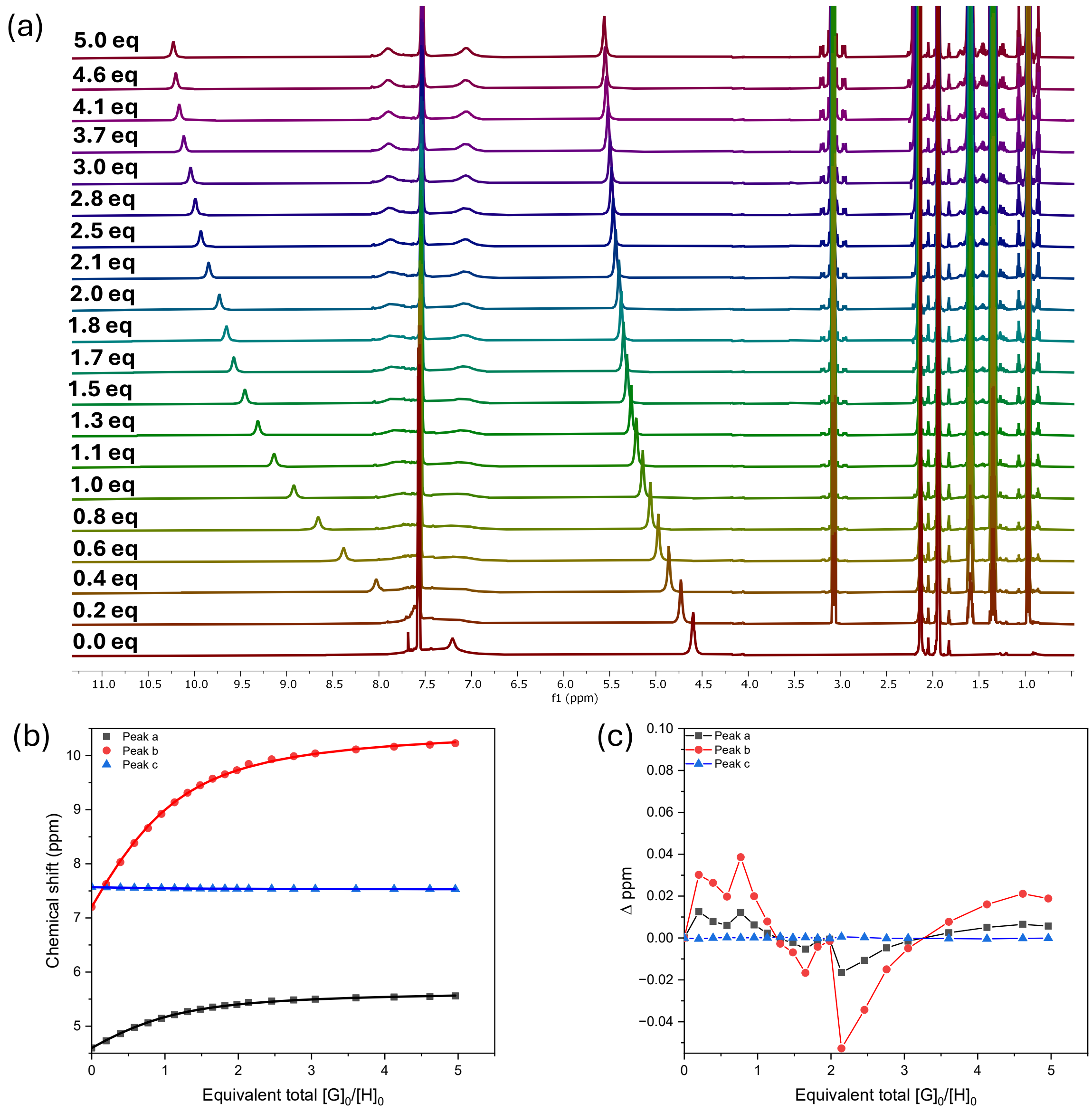

Figure S31. ^{1}H NMR titration of transporter **2c** (host, 2.5 mM) with TBACl (guest) in CD_3CN. (a) Stack plot of a representative titration. (b) Fit plot for the guanidine NH and phenyl CH peaks using global analysis and 1:1 binding stoichiometry. K_a = 1004.9 M^{-1} (error = 1.4%). (c) Plot of the residuals for the non-linear fit using global analysis and 1:1 binding stoichiometry.

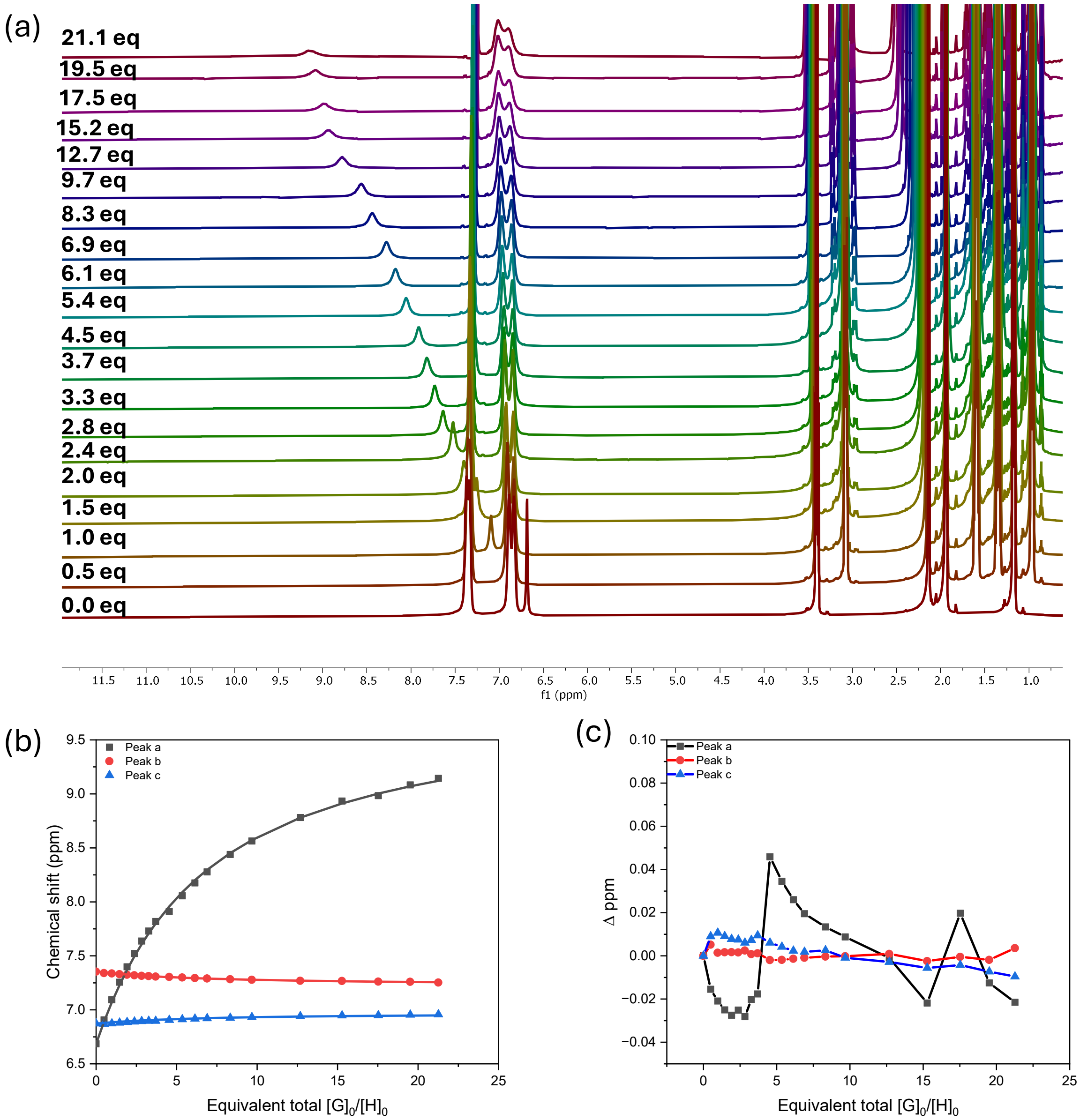

Figure S32. ^{1}H NMR titration of transporter **2d** (host, 5 mM) with TBACl (guest) in CD_3CN. (a) Stack plot of a representative titration. (b) Fitplot for the guanidine NH and phenyl CH peaks using global analysis and 1:1 binding stoichiometry. K_a = 32.7 M^{-1} (error = 1.0%). (c) Plot of the residuals for the non-linear fit using global analysis and 1:1 binding stoichiometry.

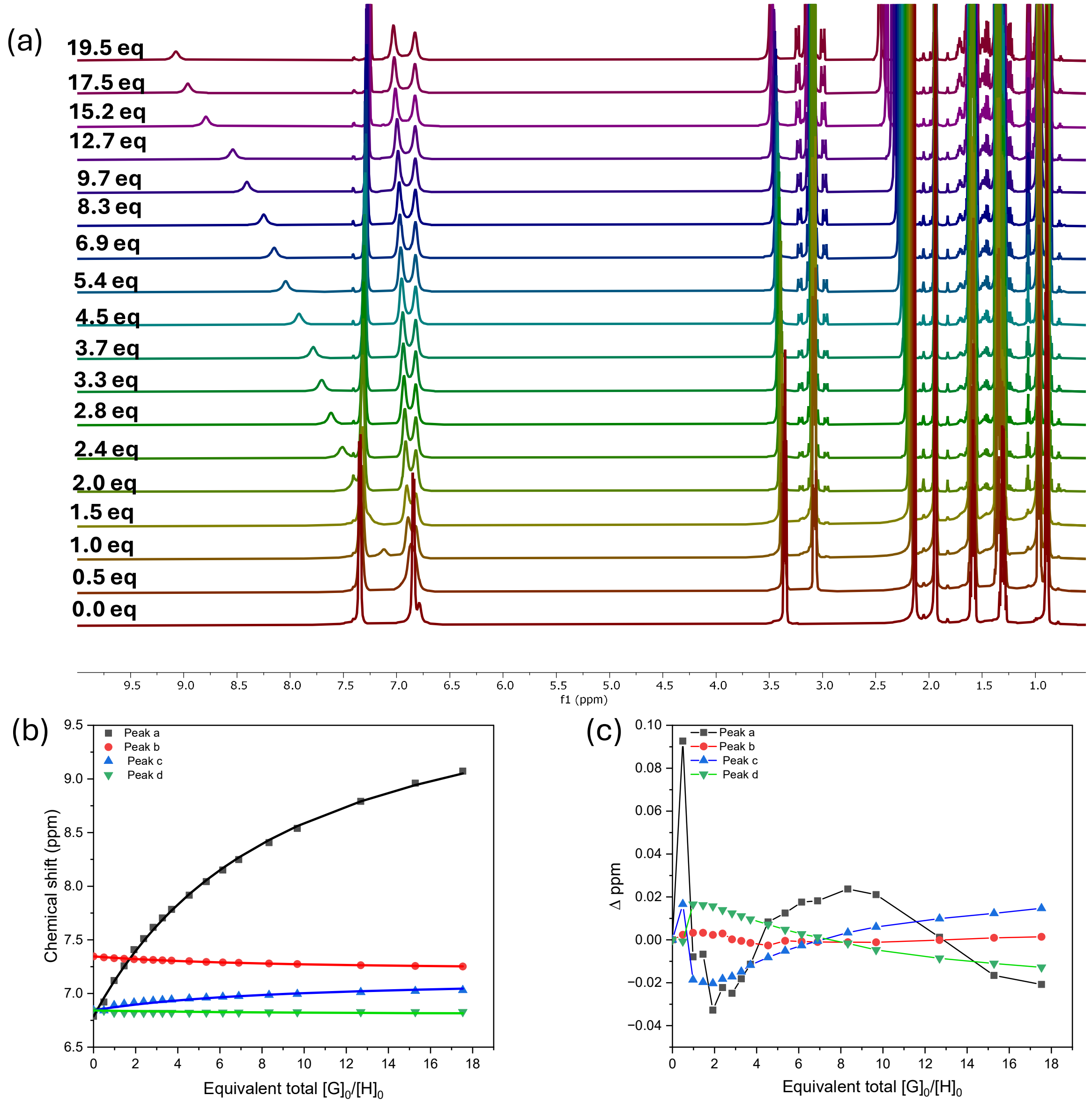

Figure S33. ^{1}H NMR titration of transporter **2e** (host, 5 mM) with TBACl (guest) in CD_3CN. (a) Stack plot of a representative titration. (b) Fitplot for the guanidine NH and phenyl CH peaks using global analysis and 1:1 binding stoichiometry. K_a = 24.3 M^{-1} (error = 1.0%). (c) Plot of the residuals for the non-linear fit using global analysis and 1:1 binding stoichiometry.

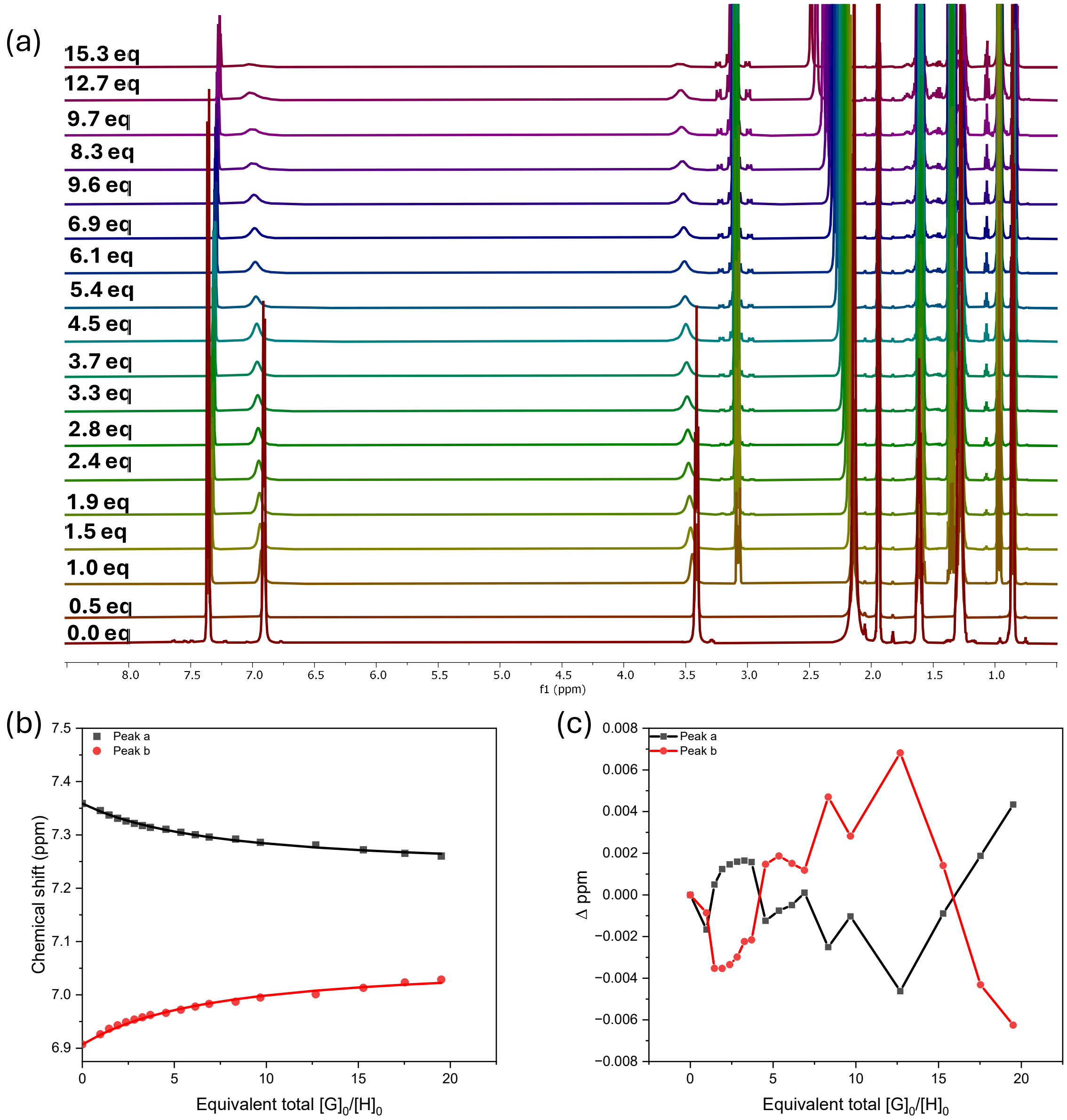

Figure S34. ^{1}H NMR titration of transporter **2f** (host, 5 mM) with TBACl (guest) in CD_3CN. (a) Stack plot of a representative titration. (b) Fitplot for the phenyl CH peaks using global analysis and 1:1 binding stoichiometry. K_a = 31 M^{-1} (error = 3.5 %). (c) Plot of the residuals for the non-linear fit using global analysis and 1:1 binding stoichiometry.

(a)

(b)

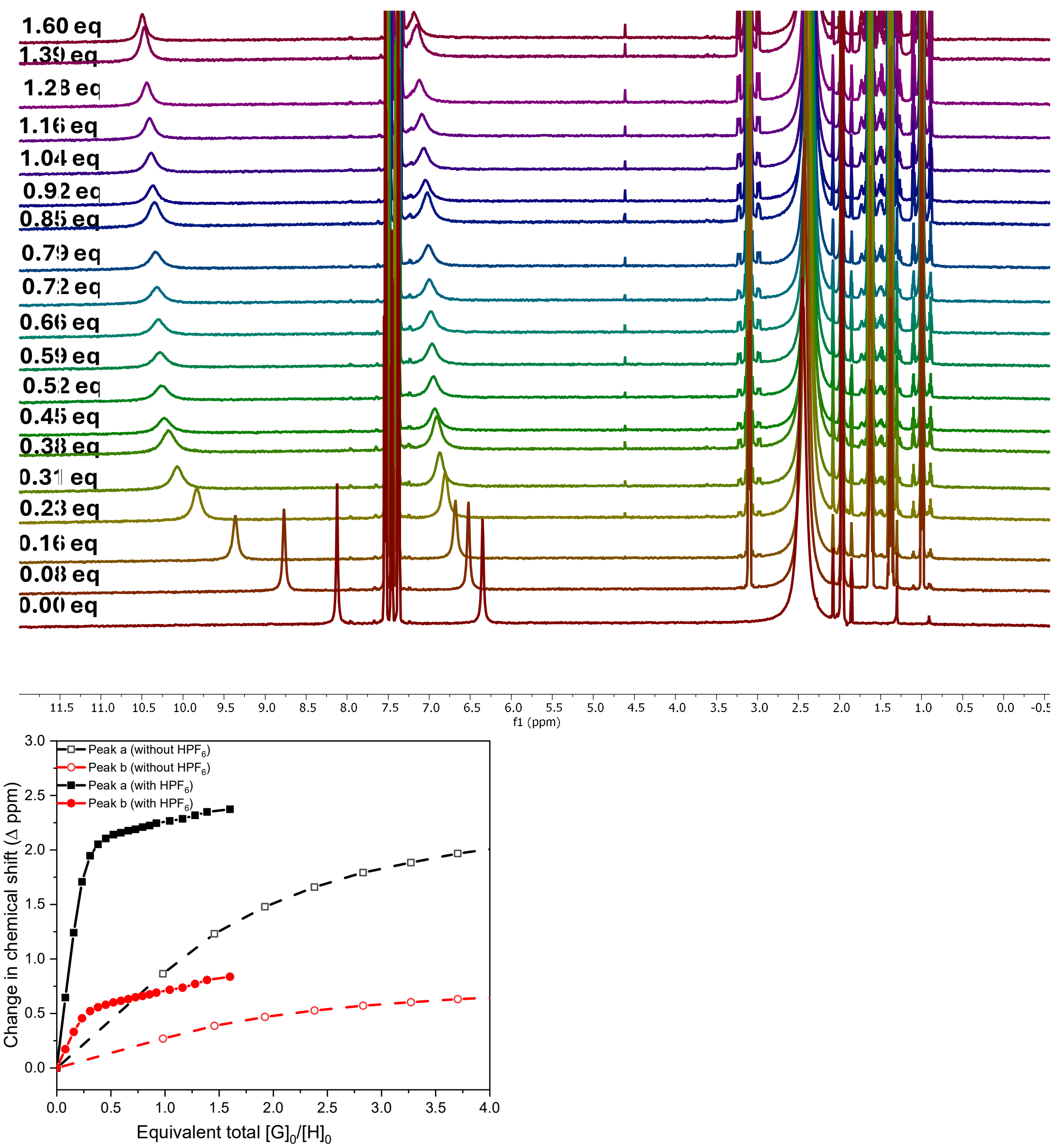

Figure S35. ^{1}H NMR titration of transporter **1c·HPF$_6$** (host, 2.5 mM) with TBACl (guest) in CD_3CN. (a) Stack plot of a representative titration. (b) Comparison of the change in chemical shifts of the guanidinium NH peaks with and without HPF_6 addition, illustrating the effect of protonation on chloride binding. Dashed lines represent the changes in chemical shifts upon addition of TBACl in the absence of HPF_6 (the corresponding stacked plot is shown in Figure S27), while solid lines represent the changes in chemical shifts observed in the presence of HPF_6. Data in the presence of HPF_6 could not be satisfactory fitted to 1:1, 1:2 or 2:1 model.

(a)

(b)

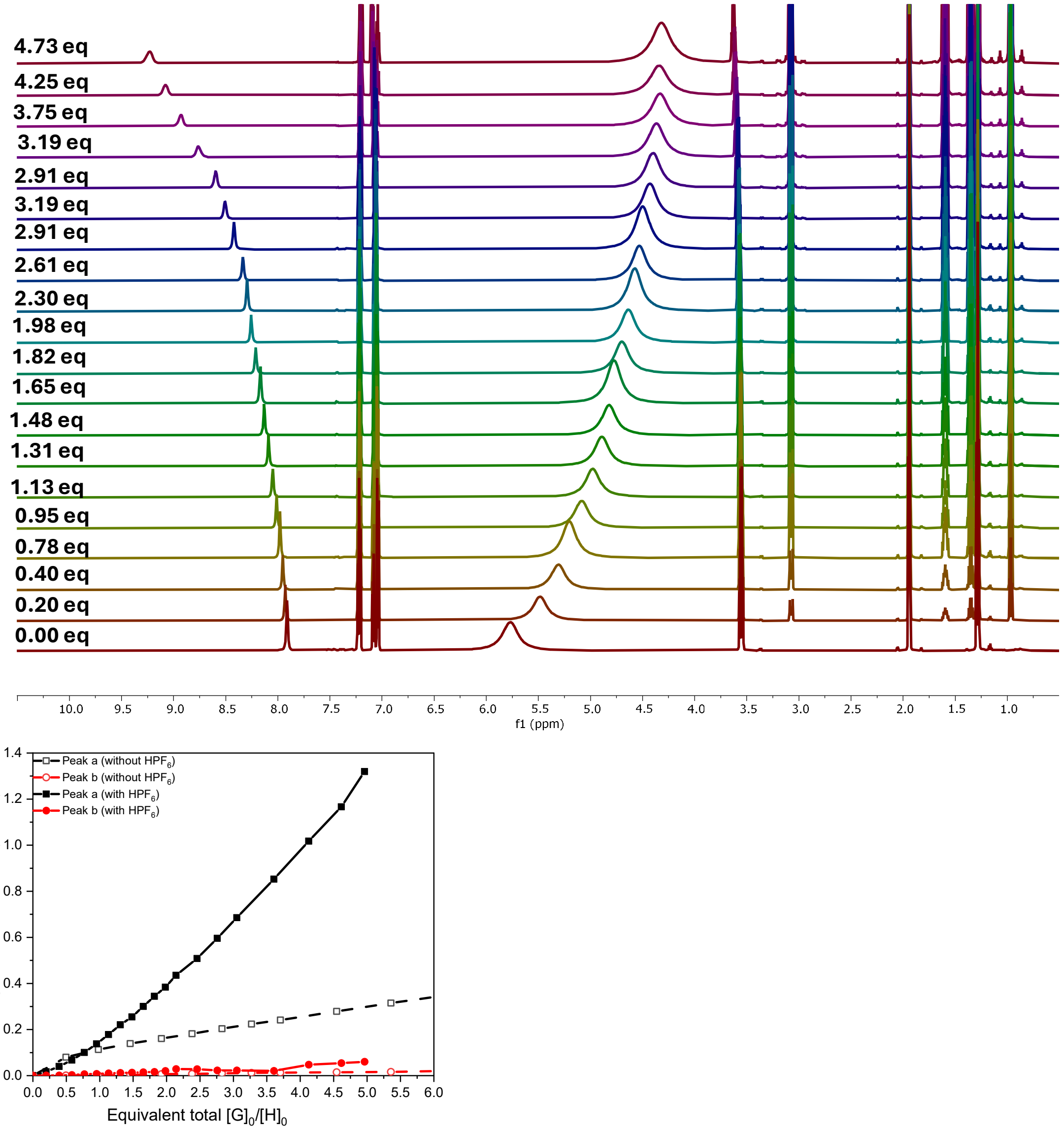

Figure S36. ^{1}H NMR titration of transporter **1d·HPF₆** (host, 2.5 mM) with TBACl (guest) in CD_3CN. (a) Stack plot of a representative titration. (b) Comparison of change in chemical shift of the guanidinium NHs and phenyl CH in the presence and absence of HPF_6. Dashed lines indicate the chemical shift trends in the absence of HPF_6 (already indicated in Figure S28) and solid lines indicate the chemical shift trends in the presence of HPF_6. Data in the presence of HPF_6 could not be satisfactory fitted to 1:1, 1:2 or 2:1 model.

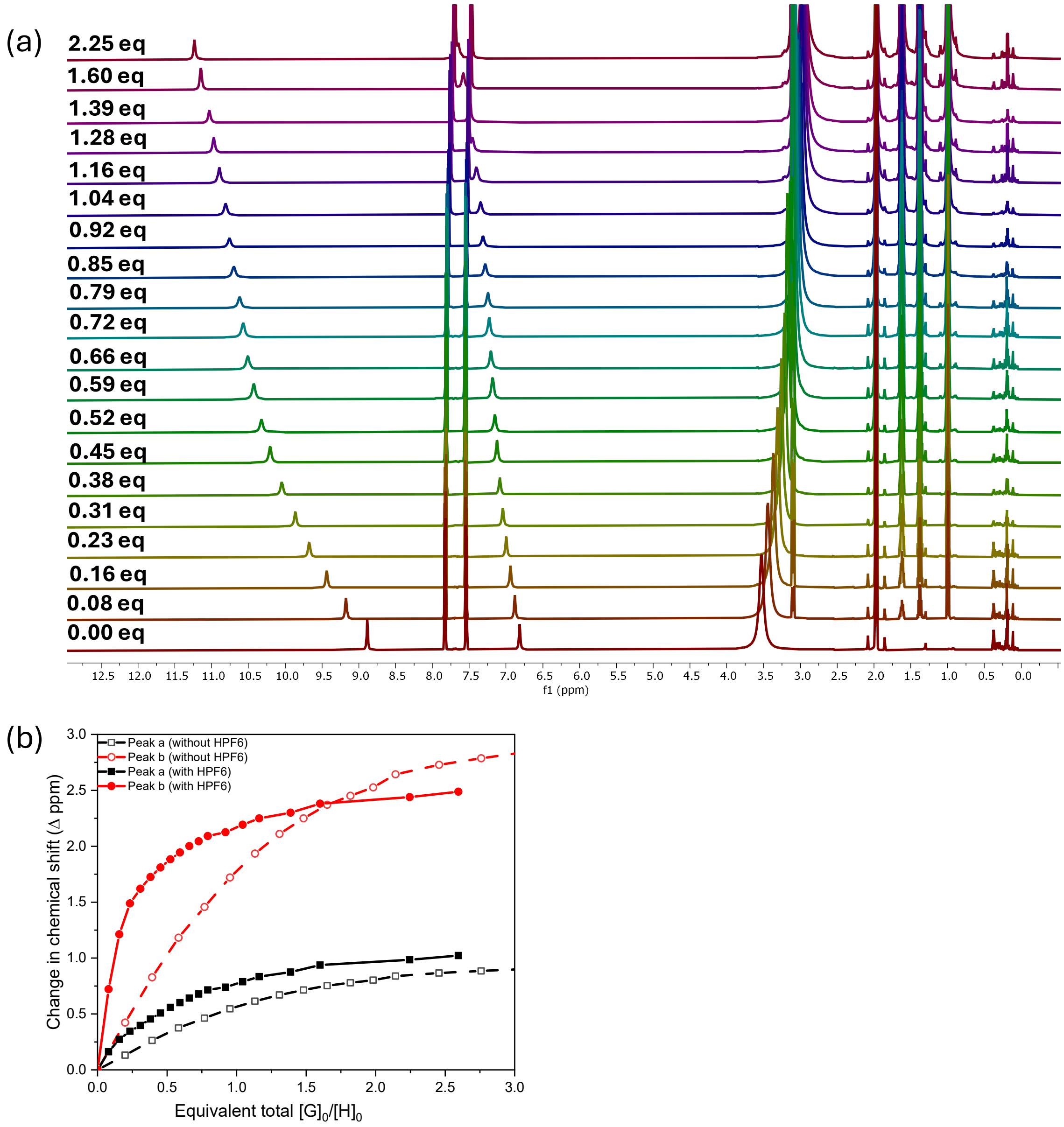

Figure S37. ^{1}H NMR titration of transporter **2c·HPF₆** (host, 2.5 mM) with TBACl (guest) in CD_3CN. (a) Stack plot of a representative titration. (b) Comparison of the change in chemical shift of the guanidinium NHs in the presence and absence of HPF_6. Dashed lines indicate the chemical shift trends in the absence of HPF_6 (already indicated in Figure S31) and solid lines indicate the chemical shift trends in the presence of HPF_6. Data in the presence of HPF_6 could not be satisfactory fitted to 1:1, 1:2 or 2:1 model.

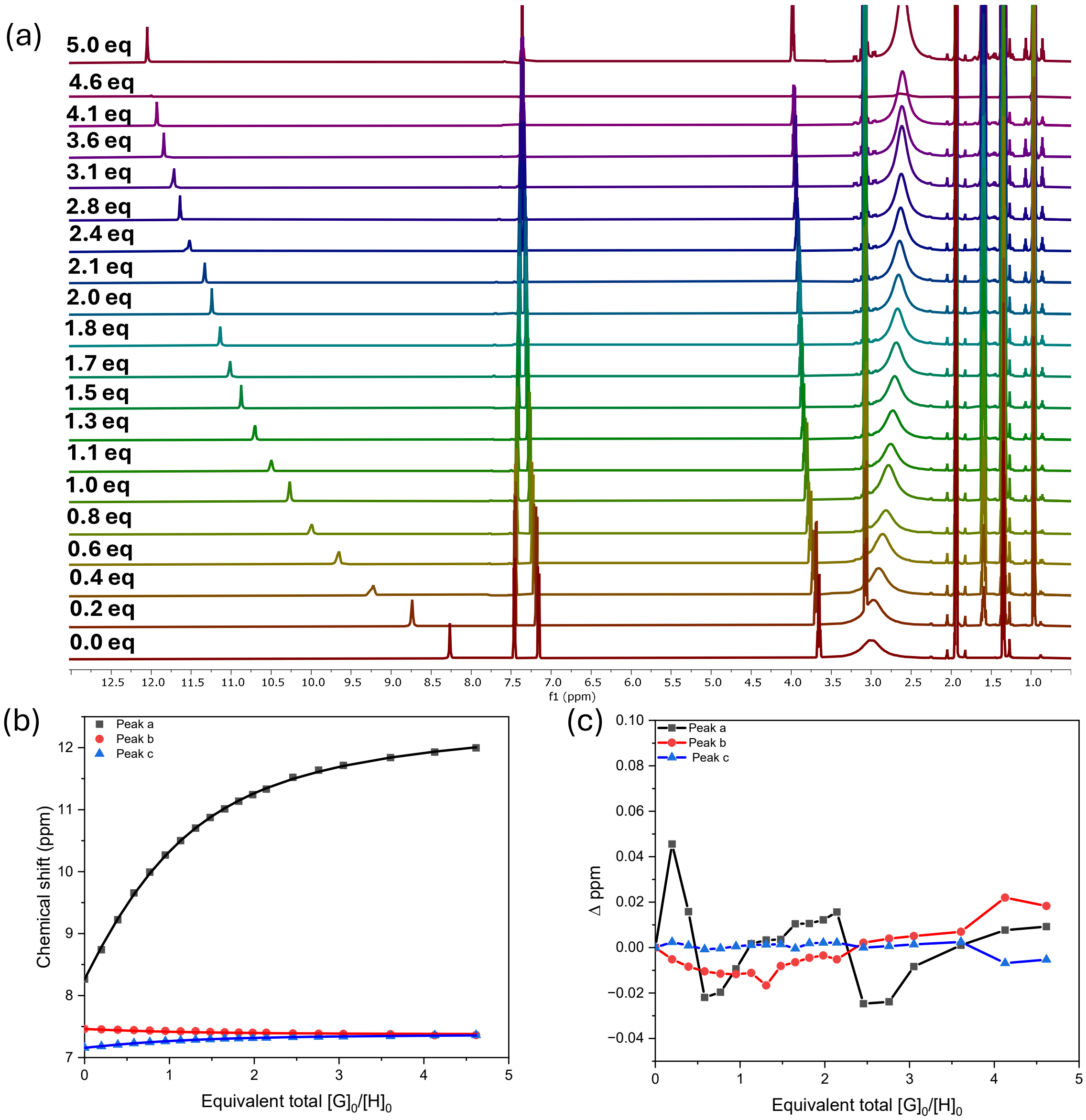

Figure S38. ^{1}H NMR titration of transporter **2d·HPF$_6$** (host, 2.5 mM) with TBACl (guest) in CD_3CN. (a) Stack plot of a representative titration. (b) Fitplot for the guanidinium NH and phenyl CH peaks using global analysis and 1:1 binding stoichiometry. K_a = 702.3 M^{-1} (error = 0.9%). (c) Plot of the residuals for the non-linear fit using global analysis and 1:1 binding stoichiometry.

S5 Single Crystal X-ray Diffraction

Single crystals suitable for X-ray diffraction were obtained by slow evaporation of a methanol solution of compound **2c** and **2d**, with a drop of 10 M HCl.

S5.1 Crystal Data for 2c·HCl

A clear colorless, block-shaped crystal was mounted on the goniometer. Data were collected from a single crystal in 24.67 hours at 150(2) K on a Bruker D8 FIXEDCHI diffractometer with a sealed tube using a flat graphite as monochromator and a PhotonIII_C7 CPAD detector. The diffractometer was equipped with a CryostreamPlus low temperature device and used MoK_α radiation (λ = 0.71073 Å). All data were integrated with SAINT V8.41, yielding 47426 reflections of which 5380 where independent (average redundancy 8.82) and 64.4% were greater than $2\sigma(F^2)$.[3] A Multi-Scan absorption correction using SADABS 2016/2 was applied.[4] The structure was solved by Intrinsic Phasing methods with SHELXT 2018/2 and refined by full-matrix least-squares methods against F^2 using SHELXL-2019/2.[5, 6] All non-hydrogen atoms were refined with anisotropic displacement parameters. All hydrogen atoms were refined with isotropic displacement parameters. Some of their coordinates were refined freely and some on calculated positions using a riding model with their U_{iso} values constrained to 1.5 times the U_{eq} of their pivot atoms for terminal sp^3 carbon atoms and 1.2 times for all other carbon atoms. Crystallographic data for the structures reported in this paper have been deposited with the Cambridge Crystallographic Data Centre,[7] CCDC 2542395 contain the supplementary crystallographic data for this paper. These data can be obtained free of charge from The Cambridge Crystallographic Data Centre via www.ccdc.cam.ac.uk/structures . The CIF file and the tables below were generated using FinalCif. The unit cell is shown in **Figure S39.**

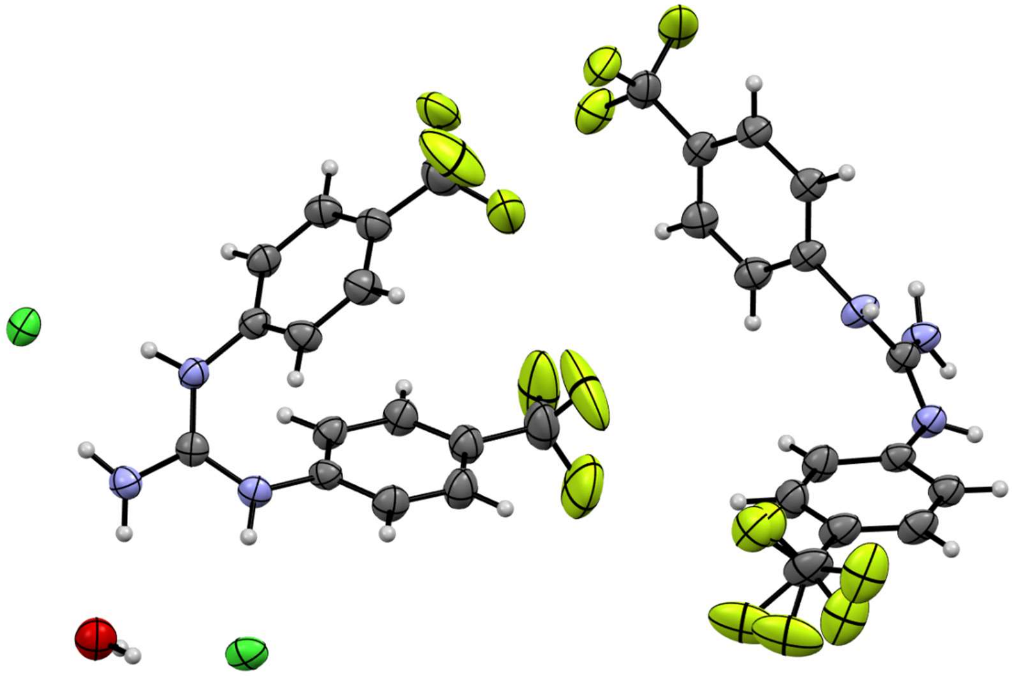

Figure S39. Unit cell of single crystals of **2c·HCl**, with atoms shown as ellipsoids representing 50% probability. Dark grey = carbon, white = hydrogen, blue = nitrogen, red = oxygen, light yellowish green = fluorine, green = chlorine.

Table S2. Crystal data and structure refinement for **2c·HCl**

CCDC number	2542395
Empirical formula	$C_{15}H_{13}ClF_6N_3O_{0.50}$
Formula weight	392.73
Temperature [K]	150(2)
Crystal system	triclinic
Space group (number)	$P\bar{1}$ (2)
a [Å]	10.5804(4)
b [Å]	11.5938(5)
c [Å]	15.2938(6)
α [c]	82.798(2)
β [°]	73.577(2)
γ [°]	74.203(2)
Volume [Å^3]	1729.16(12)
Z	4
ρ_{calc} [gcm^{-3}]	1.509
μ [mm^{-1}]	0.287
F(000)	796
Crystal size [mm^3]	0.022×0.032×0.132
Crystal colour	clear colourless
Crystal shape	block
Radiation	MoK_α (λ=0.71073 Å)
2θ range [°]	3.66 to 47.94 (0.87 Å)

Index ranges	−12 ≤ h ≤ 12<br>−13 ≤ k ≤ 13<br>−17 ≤ l ≤ 17
Reflections collected	47426
Independent reflections	5380<br>R_{int} = 0.1006<br>R_{sigma} = 0.0699
Completeness to θ = 23.968°	99.4
Data / Restraints / Parameters	5380 / 488 / 500
Goodness-of-fit on F^2	1.020
Final *R* indexes<br>[I≥2σ(I)]	R_1 = 0.0586<br>wR_2 = 0.1419
Final *R* indexes<br>[all data]	R_1 = 0.1036<br>wR_2 = 0.1660
Largest peak/hole [eÅ^{-3}]	0.84/−0.36

Table S3. Atomic coordinates and U_{eq} [Å^2] for **2c·HCl**

**Atom**	***x***	***y***	***z***	U_{eq}
Cl1	−0.00034(10)	0.75549(9)	0.98560(7)	0.0396(3)
Cl2	0.47425(11)	0.19716(10)	0.95992(8)	0.0452(3)
F1	0.3597(4)	0.6979(4)	0.4322(2)	0.1141(14)
F2	0.3118(4)	0.5359(3)	0.4233(2)	0.0993(12)
F3	0.1577(3)	0.7021(3)	0.43102(19)	0.0762(9)
F4	0.3266(4)	0.0309(4)	0.5405(3)	0.1109(13)
F5	0.1522(4)	0.1599(4)	0.5289(3)	0.1284(17)
F6	0.3501(6)	0.1875(5)	0.4691(3)	0.1415(19)
F7A	1.3577(5)	0.0093(6)	0.2426(5)	0.0772(18)
F8A	1.2419(9)	−0.0514(7)	0.3688(6)	0.091(2)
F9A	1.199(3)	0.1340(11)	0.3310(15)	0.061(3)
F7B	1.3251(11)	−0.0482(13)	0.2933(12)	0.0772(18)
F8B	1.1610(15)	−0.0173(15)	0.4081(10)	0.091(2)
F9B	1.205(6)	0.131(2)	0.316(3)	0.061(3)
F10	0.1653(2)	0.5354(2)	0.2824(2)	0.0575(7)
F11	0.2987(3)	0.6514(2)	0.23216(18)	0.0560(7)
F12	0.2107(3)	0.5944(3)	0.1412(2)	0.0779(10)
O1	0.2099(3)	0.2770(3)	1.1157(2)	0.0567(9)
H2	0.178(5)	0.223(4)	1.102(4)	0.085
H2B	0.295(3)	0.245(5)	1.085(4)	0.085
N1	0.1246(3)	0.5485(3)	0.8466(2)	0.0349(8)
H5	0.065(4)	0.609(3)	0.878(3)	0.052
N2	0.2698(3)	0.3568(3)	0.8581(2)	0.0344(8)
N3	0.1714(4)	0.4719(3)	0.9825(2)	0.0362(8)
H1	0.126(4)	0.547(2)	1.001(3)	0.054
H1B	0.195(4)	0.404(3)	1.017(3)	0.054
N4	0.8455(3)	−0.0346(3)	0.1218(2)	0.0362(8)
H1E	0.869(4)	−0.095(3)	0.086(3)	0.054
N5	0.7264(3)	0.1635(3)	0.1212(2)	0.0367(8)
H1G	0.805(3)	0.184(4)	0.104(3)	0.055
N6	0.6355(3)	0.0183(3)	0.0902(3)	0.0391(9)

H1C	0.578(4)	0.076(3)	0.066(3)	0.059
H1D	0.641(5)	−0.060(2)	0.087(3)	0.059
C1	0.1888(4)	0.4584(4)	0.8948(3)	0.0336(9)
C2	0.1626(4)	0.5753(3)	0.7510(3)	0.0335(9)
C3	0.0597(4)	0.6208(4)	0.7081(3)	0.0379(10)
H3	−0.032655	0.635302	0.742631	0.045
C4	0.0909(4)	0.6454(4)	0.6148(3)	0.0422(10)
H4	0.020183	0.676976	0.585067	0.051
C5	0.2253(4)	0.6238(4)	0.5648(3)	0.0428(10)
C6	0.3284(4)	0.5813(4)	0.6090(3)	0.0444(11)
H6	0.420850	0.569050	0.574981	0.053
C7	0.2970(4)	0.5569(4)	0.7025(3)	0.0391(10)
H7	0.367376	0.527733	0.732802	0.047
C8	0.2615(5)	0.6409(5)	0.4635(4)	0.0619(14)
C9	0.2668(4)	0.3047(3)	0.7798(3)	0.0335(9)
C10	0.1453(4)	0.3155(4)	0.7575(3)	0.0367(10)
H10	0.061504	0.357592	0.795246	0.044
C11	0.1477(4)	0.2643(4)	0.6800(3)	0.0432(11)
H11	0.065329	0.272953	0.663618	0.052
C12	0.2697(5)	0.2004(4)	0.6259(3)	0.0471(11)
C13	0.3887(4)	0.1859(4)	0.6512(3)	0.0480(11)
H13	0.471798	0.139606	0.615696	0.058
C14	0.3879(4)	0.2383(4)	0.7277(3)	0.0432(11)
H14	0.470238	0.228686	0.744489	0.052
C15	0.2730(6)	0.1485(6)	0.5412(4)	0.0681(15)
C16	0.7344(4)	0.0485(4)	0.1114(3)	0.0338(9)
C17	0.9379(4)	−0.0189(3)	0.1684(3)	0.0341(10)
C18	0.8930(4)	0.0380(4)	0.2498(3)	0.0431(11)
H18	0.798276	0.067727	0.275898	0.052
C19	0.9854(5)	0.0517(4)	0.2931(3)	0.0484(12)
H19	0.954217	0.092650	0.348165	0.058
C20	1.1231(5)	0.0063(4)	0.2568(3)	0.0480(11)
C21	1.1687(4)	−0.0545(4)	0.1771(3)	0.0432(11)
H21	1.263265	−0.087121	0.152590	0.052
C22	1.0765(4)	−0.0674(4)	0.1333(3)	0.0390(10)
H22	1.107767	−0.109735	0.078837	0.047
C23	1.2219(5)	0.0234(5)	0.3038(4)	0.0643(14)
C24	0.6068(4)	0.2581(3)	0.1425(3)	0.0322(9)
C25	0.4986(4)	0.2456(4)	0.2160(3)	0.0399(10)
H25	0.501337	0.171244	0.250417	0.048
C26	0.3861(4)	0.3419(4)	0.2393(3)	0.0402(10)
H26	0.310579	0.333225	0.288646	0.048
C27	0.3846(4)	0.4507(4)	0.1900(3)	0.0372(10)
C28	0.4938(4)	0.4634(4)	0.1171(3)	0.0372(10)
H28	0.493016	0.538443	0.083977	0.045
C29	0.6036(4)	0.3661(3)	0.0931(3)	0.0338(9)
H29	0.677423	0.373695	0.042027	0.041
H1F	0.330(4)	0.318(3)	0.890(3)	0.051
C30	0.2653(4)	0.5562(4)	0.2123(3)	0.0455(11)

U_{eq} is defined as 1/3 of the trace of the orthogonalized U_{ij} tensor.

Table S4. Anisotropic displacement parameters (Å^2) for **2c·HCl**. The anisotropic displacement factor exponent takes the form: $-2\pi^2[\, h^2(a^*)^2U_{11} + k^2(b^*)^2U_{22} + \ldots + 2hka^*b^*U_{12}\,]$.

Atom	U_{11}	U_{22}	U_{33}	U_{23}	U_{13}	U_{12}
Cl1	0.0327(6)	0.0398(6)	0.0489(7)	−0.0115(5)	−0.0115(5)	−0.0081(5)
Cl2	0.0403(6)	0.0450(7)	0.0585(7)	−0.0016(5)	−0.0297(6)	−0.0073(5)
F1	0.087(3)	0.200(4)	0.059(2)	0.045(2)	−0.0175(19)	−0.065(3)
F2	0.116(3)	0.103(3)	0.0497(19)	−0.0194(18)	−0.0270(19)	0.034(2)
F3	0.0639(19)	0.103(2)	0.0448(17)	0.0057(16)	−0.0226(15)	0.0106(17)
F4	0.122(3)	0.106(3)	0.110(3)	−0.066(2)	−0.044(3)	0.004(2)
F5	0.077(2)	0.209(5)	0.111(3)	−0.099(3)	−0.052(2)	0.015(3)
F6	0.212(5)	0.205(5)	0.041(2)	−0.025(2)	−0.011(3)	−0.122(4)
F7A	0.032(2)	0.099(4)	0.116(5)	−0.047(4)	−0.030(3)	−0.012(2)
F8A	0.088(5)	0.110(5)	0.115(5)	0.041(4)	−0.082(4)	−0.052(5)
F9A	0.060(3)	0.0643(19)	0.076(7)	−0.021(2)	−0.040(4)	−0.0118(15)
F7B	0.032(2)	0.099(4)	0.116(5)	−0.047(4)	−0.030(3)	−0.012(2)
F8B	0.088(5)	0.110(5)	0.115(5)	0.041(4)	−0.082(4)	−0.052(5)
F9B	0.060(3)	0.0643(19)	0.076(7)	−0.021(2)	−0.040(4)	−0.0118(15)
F10	0.0313(14)	0.0607(17)	0.0739(19)	−0.0174(14)	−0.0068(13)	−0.0017(12)
F11	0.0573(17)	0.0423(15)	0.0634(18)	−0.0146(13)	−0.0125(14)	−0.0022(12)
F12	0.0611(18)	0.096(2)	0.0662(19)	−0.0304(17)	−0.0412(16)	0.0353(16)
O1	0.048(2)	0.064(2)	0.059(2)	−0.0077(18)	−0.0182(18)	−0.0094(18)
N1	0.0313(19)	0.035(2)	0.036(2)	−0.0075(16)	−0.0114(16)	−0.0001(15)
N2	0.0283(19)	0.041(2)	0.038(2)	−0.0046(16)	−0.0165(16)	−0.0058(16)
N3	0.040(2)	0.037(2)	0.036(2)	−0.0032(16)	−0.0152(17)	−0.0098(17)
N4	0.0313(19)	0.036(2)	0.046(2)	−0.0093(16)	−0.0195(17)	−0.0031(16)
N5	0.0247(18)	0.034(2)	0.055(2)	−0.0062(17)	−0.0166(17)	−0.0053(15)
N6	0.037(2)	0.034(2)	0.053(2)	−0.0033(18)	−0.0237(18)	−0.0063(17)
C1	0.027(2)	0.040(2)	0.037(2)	−0.0055(19)	−0.0105(19)	−0.0103(19)
C2	0.031(2)	0.032(2)	0.037(2)	−0.0032(18)	−0.0110(18)	−0.0050(18)
C3	0.028(2)	0.042(2)	0.041(2)	−0.005(2)	−0.0096(19)	−0.0022(19)
C4	0.036(2)	0.047(3)	0.043(3)	−0.001(2)	−0.017(2)	−0.003(2)
C5	0.040(3)	0.047(3)	0.038(2)	0.000(2)	−0.013(2)	−0.005(2)
C6	0.032(2)	0.055(3)	0.043(3)	0.004(2)	−0.008(2)	−0.010(2)
C7	0.032(2)	0.046(3)	0.040(2)	0.002(2)	−0.0145(19)	−0.008(2)
C8	0.053(3)	0.079(4)	0.045(3)	0.001(3)	−0.014(2)	−0.002(3)
C9	0.034(2)	0.034(2)	0.036(2)	−0.0007(18)	−0.0169(19)	−0.0079(18)
C10	0.030(2)	0.036(2)	0.044(3)	−0.0056(19)	−0.0123(19)	−0.0047(18)
C11	0.037(2)	0.049(3)	0.048(3)	−0.012(2)	−0.020(2)	−0.005(2)
C12	0.046(3)	0.052(3)	0.044(3)	−0.011(2)	−0.016(2)	−0.006(2)
C13	0.036(2)	0.056(3)	0.049(3)	−0.016(2)	−0.011(2)	−0.001(2)
C14	0.031(2)	0.050(3)	0.049(3)	−0.008(2)	−0.016(2)	−0.003(2)
C15	0.062(3)	0.090(4)	0.052(3)	−0.029(3)	−0.023(3)	0.002(3)
C16	0.027(2)	0.036(2)	0.041(2)	−0.0064(19)	−0.0160(19)	−0.0038(18)
C17	0.030(2)	0.033(2)	0.043(2)	−0.0018(19)	−0.0191(19)	−0.0031(18)
C18	0.031(2)	0.047(3)	0.052(3)	−0.012(2)	−0.017(2)	−0.002(2)
C19	0.048(3)	0.049(3)	0.052(3)	−0.017(2)	−0.024(2)	0.000(2)
C20	0.047(3)	0.039(3)	0.068(3)	−0.009(2)	−0.035(2)	−0.002(2)
C21	0.029(2)	0.042(3)	0.062(3)	−0.007(2)	−0.021(2)	−0.0015(19)
C22	0.037(2)	0.037(2)	0.046(3)	−0.0050(19)	−0.020(2)	−0.0039(19)
C23	0.060(3)	0.055(3)	0.091(4)	−0.012(3)	−0.052(3)	0.002(3)
C24	0.026(2)	0.032(2)	0.046(3)	−0.0053(19)	−0.0185(19)	−0.0070(17)

C25	0.035(2)	0.039(2)	0.047(3)	−0.001(2)	−0.015(2)	−0.0071(19)
C26	0.027(2)	0.050(3)	0.045(3)	−0.005(2)	−0.0105(19)	−0.0087(19)
C27	0.030(2)	0.038(2)	0.049(3)	−0.012(2)	−0.020(2)	−0.0043(18)
C28	0.037(2)	0.034(2)	0.047(3)	−0.0053(19)	−0.020(2)	−0.0071(19)
C29	0.024(2)	0.038(2)	0.045(3)	−0.0043(19)	−0.0150(18)	−0.0094(18)
C30	0.036(2)	0.053(3)	0.047(3)	−0.015(2)	−0.018(2)	0.003(2)

S5.2 Crystal Data for 2d·HCl

A clear colorless, plate-shaped crystal was mounted on the goniometer. Data were collected from a single crystal in 9.30 hours at 150(2) K on a Bruker D8 FIXEDCHI diffractometer with a sealed tube using a flat graphite as monochromator and a PhotonIII_C7 CPAD detector. The diffractometer was equipped with a CryostreamPlus low temperature device and used MoK_α radiation (λ = 0.71073 Å). All data were integrated with SAINT V8.41, yielding 27280 reflections of which 3633 where independent (average redundancy 7.51) and 76.4% were greater than 2$\sigma(F^2)$.[3] A Multi-Scan absorption correction using SADABS 2016/2 was applied.[4] The structure was solved by Intrinsic Phasing methods with SHELXT 2018/2 and refined by full-matrix least-squares methods against F^2 using SHELXL-2019/2.[5, 6] All non-hydrogen atoms were refined with anisotropic displacement parameters. All hydrogen atoms were refined with isotropic displacement parameters. Some of their coordinates were refined freely and some on calculated positions using a riding model with their U_{iso} values constrained to 1.5 times the U_{eq} of their pivot atoms for terminal sp^3 carbon atoms and 1.2 times for all other carbon atoms. Crystallographic data for the structures reported in this paper have been deposited with the Cambridge Crystallographic Data Centre,[7] CCDC 2542396 contain the supplementary crystallographic data for this paper. These data can be obtained free of charge from The Cambridge Crystallographic Data Centre via www.ccdc.cam.ac.uk/structures . The CIF file and the tables below were generated using FinalCif. The unit cell is shown in **Figure S40**.

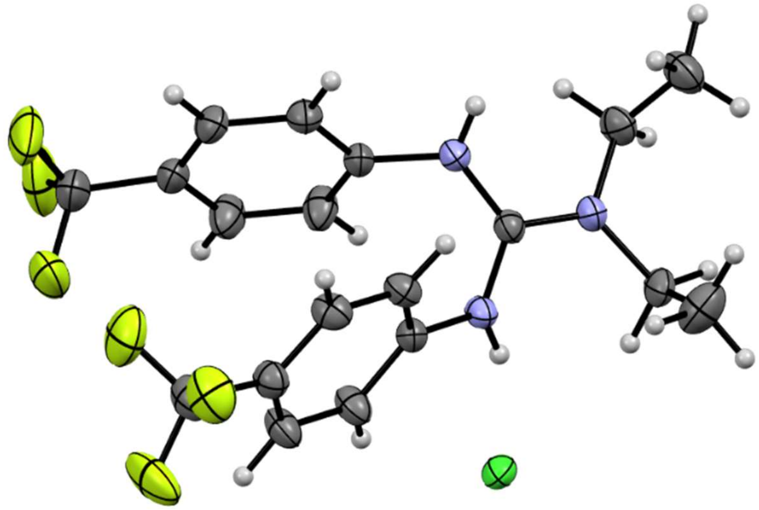

Figure S40. Unit cell of single crystals of **2d·HCl**, with atoms shown as ellipsoids representing 50% probability. Dark grey = carbon, white = hydrogen, blue = nitrogen, light yellowish green = fluorine, green = chlorine.

Table S5. Crystal data and structure refinement for **2d·HCl**.

CCDC number	2542396
Empirical formula	$C_{19}H_{20}ClF_6N_3$
Formula weight	439.83
Temperature [K]	150(2)
Crystal system	monoclinic
Space group (number)	$P2_1/c$ (14)
a [Å]	12.8658(3)
b [Å]	15.1322(4)
c [Å]	11.0358(3)
α [°]	90
β [°]	106.7770(10)
γ [°]	90
Volume [Å^3]	2057.08(9)
Z	4
ρ_{calc} [gcm^{-3}]	1.42
μ [mm^{-1}]	0.248
F(000)	904
Crystal size [mm^3]	0.019×0.099×0.215
Crystal colour	clear colourless
Crystal shape	plate
Radiation	MoK_α (λ=0.71073 Å)
2θ range [°]	5.07 to 50.15 (0.84 Å)
Index ranges	−15 ≤ h ≤ 14<br>−18 ≤ k ≤ 18<br>−12 ≤ l ≤ 13

Reflections collected	27280
Independent reflections	3633<br>R_{int} = 0.0385<br>R_{sigma} = 0.0275
Completeness to θ = 25.073°	99.5
Data / Restraints / Parameters	3633 / 245 / 270
Goodness-of-fit on F^2	1.032
Final R indexes [I≥2σ(I)]	R_1 = 0.0407<br>wR_2 = 0.0939
Final R indexes [all data]	R_1 = 0.0594<br>wR_2 = 0.1032
Largest peak/hole [eÅ^{-3}]	0.31/−0.24

Table S6. Atomic coordinates and Ueq [Å^2] for **2d·HCl**.

**Atom**	***x***	***y***	***z***	U_{eq}
Cl1	0.51239(4)	0.89442(3)	0.11546(5)	0.03287(16)
F1	1.04677(11)	0.71731(11)	0.65320(14)	0.0568(4)
F2	1.07061(11)	0.78715(12)	0.49508(17)	0.0700(5)
F3	1.05449(12)	0.64624(11)	0.48968(16)	0.0639(5)
F4	0.97229(14)	0.44607(12)	0.1857(2)	0.0790(6)
F5	0.97535(12)	0.58383(11)	0.15252(17)	0.0628(5)
F6	0.89081(13)	0.49965(14)	0.00369(17)	0.0764(6)
N1	0.56074(14)	0.73182(12)	0.29594(18)	0.0276(4)
H2	0.534(2)	0.7782(17)	0.251(2)	0.041
N2	0.53629(14)	0.57828(12)	0.28472(17)	0.0276(4)
H1	0.510(2)	0.5365(17)	0.314(2)	0.041
N3	0.38856(14)	0.67197(11)	0.25863(17)	0.0284(4)
C1	0.49471(16)	0.66021(13)	0.28039(19)	0.0260(5)
C2	0.34266(18)	0.75893(15)	0.2764(2)	0.0334(5)
H2A	0.263706	0.758425	0.233129	0.040
H2B	0.375913	0.804844	0.235602	0.040
C3	0.3602(2)	0.78350(18)	0.4128(3)	0.0530(7)
H3A	0.329732	0.842272	0.417699	0.080
H3B	0.438220	0.784115	0.456580	0.080
H3C	0.324241	0.740132	0.452946	0.080
C4	0.31033(18)	0.59956(15)	0.2121(2)	0.0345(5)
H4A	0.347136	0.551161	0.180298	0.041
H4B	0.250453	0.621487	0.140257	0.041
C5	0.2630(2)	0.56308(18)	0.3125(3)	0.0452(6)
H5A	0.213366	0.514416	0.276847	0.068
H5B	0.223313	0.609898	0.341547	0.068
H5C	0.321828	0.541299	0.384151	0.068
C6	0.67364(16)	0.73102(13)	0.3587(2)	0.0258(5)
C7	0.71656(18)	0.68119(14)	0.4672(2)	0.0302(5)
H7	0.669845	0.648725	0.503538	0.036
C8	0.82729(18)	0.67906(15)	0.5220(2)	0.0330(5)
H8	0.856907	0.643562	0.594770	0.040
C9	0.89597(17)	0.72838(14)	0.4716(2)	0.0320(5)
C10	0.85232(18)	0.78154(16)	0.3676(2)	0.0369(6)

H10	0.898637	0.817357	0.334984	0.044
C11	0.74144(17)	0.78282(15)	0.3108(2)	0.0336(5)
H11	0.711749	0.819212	0.239007	0.040
C12	1.01552(19)	0.72059(17)	0.5262(2)	0.0431(6)
C13	0.62760(16)	0.55833(13)	0.2421(2)	0.0275(5)
C14	0.70501(18)	0.49923(14)	0.3101(2)	0.0314(5)
H14	0.695297	0.470227	0.382361	0.038
C15	0.79633(18)	0.48287(14)	0.2718(2)	0.0342(5)
H15	0.849679	0.442455	0.317892	0.041
C16	0.81040(18)	0.52513(14)	0.1664(2)	0.0328(5)
C17	0.7316(2)	0.58144(16)	0.0970(2)	0.0424(6)
H17	0.740544	0.609226	0.023463	0.051
C18	0.6395(2)	0.59769(16)	0.1339(2)	0.0386(6)
H18	0.584576	0.635803	0.085124	0.046
C19	0.9114(2)	0.51299(16)	0.1283(3)	0.0423(6)

U_{eq} is defined as 1/3 of the trace of the orthogonalized U_{ij} tensor.

Table S7. Anisotropic displacement parameters (Å^2) for **2d·HCl**. The anisotropic displacement factor exponent takes the form: $-2\pi^2[\, h^2(a^*)^2U_{11} + k^2(b^*)^2U_{22} + \ldots + 2hka^*b^*U_{12}\,]$.

Atom	U_{11}	U_{22}	U_{33}	U_{23}	U_{13}	U_{12}
Cl1	0.0339(3)	0.0251(3)	0.0431(3)	0.0042(2)	0.0167(3)	0.0060(2)
F1	0.0353(8)	0.0771(11)	0.0470(9)	0.0059(8)	−0.0055(7)	0.0014(7)
F2	0.0263(8)	0.0814(12)	0.0933(13)	0.0365(10)	0.0030(8)	−0.0108(8)
F3	0.0405(9)	0.0752(11)	0.0716(11)	−0.0027(9)	0.0091(8)	0.0217(8)
F4	0.0636(11)	0.0678(11)	0.1278(16)	0.0315(11)	0.0626(11)	0.0346(9)
F5	0.0416(9)	0.0603(10)	0.0960(13)	−0.0160(9)	0.0350(9)	−0.0128(7)
F6	0.0481(10)	0.1246(16)	0.0675(12)	−0.0333(11)	0.0339(9)	−0.0037(10)
N1	0.0211(9)	0.0247(9)	0.0369(11)	0.0061(8)	0.0085(8)	0.0012(7)
N2	0.0244(10)	0.0246(9)	0.0368(11)	0.0027(8)	0.0135(8)	−0.0001(7)
N3	0.0213(10)	0.0280(9)	0.0370(11)	0.0000(8)	0.0099(8)	0.0000(7)
C1	0.0243(11)	0.0281(11)	0.0275(12)	0.0029(9)	0.0105(9)	0.0002(9)
C2	0.0229(11)	0.0323(12)	0.0455(14)	0.0051(10)	0.0108(10)	0.0070(9)
C3	0.0580(18)	0.0479(16)	0.0503(16)	−0.0071(13)	0.0110(14)	0.0165(13)
C4	0.0219(11)	0.0366(12)	0.0415(14)	−0.0011(10)	0.0037(10)	−0.0036(10)
C5	0.0318(13)	0.0496(15)	0.0553(16)	0.0024(13)	0.0142(12)	−0.0095(11)
C6	0.0229(11)	0.0255(11)	0.0304(12)	−0.0004(9)	0.0096(9)	0.0007(9)
C7	0.0309(12)	0.0314(12)	0.0298(12)	0.0012(9)	0.0109(10)	−0.0049(9)
C8	0.0324(13)	0.0347(12)	0.0298(12)	0.0051(10)	0.0055(10)	−0.0014(10)
C9	0.0251(11)	0.0343(12)	0.0351(13)	0.0023(10)	0.0065(10)	0.0006(9)
C10	0.0270(12)	0.0441(14)	0.0409(14)	0.0112(11)	0.0120(11)	−0.0042(10)
C11	0.0258(12)	0.0391(13)	0.0354(13)	0.0119(10)	0.0081(10)	−0.0012(10)
C12	0.0311(13)	0.0489(15)	0.0471(16)	0.0101(12)	0.0077(12)	0.0015(11)
C13	0.0228(11)	0.0247(11)	0.0371(13)	−0.0023(9)	0.0121(10)	0.0004(9)
C14	0.0315(12)	0.0272(11)	0.0374(13)	0.0047(9)	0.0131(10)	0.0017(9)
C15	0.0278(12)	0.0293(12)	0.0452(14)	0.0019(10)	0.0099(11)	0.0067(9)
C16	0.0296(12)	0.0269(11)	0.0456(14)	−0.0056(10)	0.0168(11)	0.0005(9)
C17	0.0494(15)	0.0425(14)	0.0447(15)	0.0107(11)	0.0284(13)	0.0104(12)
C18	0.0384(14)	0.0431(14)	0.0387(14)	0.0130(11)	0.0179(11)	0.0144(11)
C19	0.0376(14)	0.0389(14)	0.0570(17)	−0.0059(12)	0.0240(12)	0.0021(11)

S6 Chloride Transport Assays

S6.1 Lucigenin Assay (external addition or pre-incorporation)

The lucigenin assay was conducted following established procedures.[8] Palmitoyloleoylphosphatidylcholine (POPC) was stored as a chloroform solution at -20°C until use. Buffer solutions were always freshly prepared using UltraPure water, following an online buffer calculator to achieve a 5 mM HEPES buffer at pH 7.4 with an ionic strength of 225 mM at 25°C.

***For external addition*:** A measured volume of the phosphatidylcholine (POPC) stock solution in chloroform was transferred into a round-bottom flask and evaporated using a rotary evaporator to form a thin lipid film. To ensure complete removal of residual solvent, the film was subjected to high vacuum drying for a minimum of five hours. Hydration of the lipid film was carried out with the internal buffer solution containing 1 mM lucigenin, 225 mM $NaNO_3$, and 5 mM HEPES (pH 7.4), followed by vortexing for approximately five minutes. The resulting lipid suspension underwent nine cycles of freeze-thawing, alternating between rapid freezing in liquid nitrogen and thawing in water maintained at approximately 30°C. After equilibration at room temperature for 30 minutes, the suspension was extruded 25 times through a 100 nm polycarbonate membrane (Nucleopore) using an Avanti mini extruder set (Avanti Polar Lipids, Inc.), producing large unilamellar vesicles (LUVs). To remove any unencapsulated lucigenin, the vesicle solution was purified via size-exclusion chromatography on a Sephadex G-25 (medium) column. The final stock of concentrated liposomes was diluted with an external buffer (225 mM $NaNO_3$, 5 mM HEPES, pH 7.4) to obtain a total lipid concentration of 0.5 mM. 3 mL of lucigenin-loaded liposomes (0.5 mM total lipid concentration) were transferred into a glass fluorescence cuvette containing a small magnetic stir bar. The cuvette was placed inside the fluorometer, and stirring was initiated at maximum speed, continuing throughout the experiment. Data acquisition began with excitation and emission wavelengths set at 430 nm and 505 nm, respectively. At t = 10 s, 75 µL of a 1 M NaCl stock solution was introduced into the cuvette to achieve a final concentration of 25 mM NaCl. At t = 40 s, 7.5 µL of either methanol (MeOH) or the test compound (prepared as a 10 mM solution in MeOH) was added. At t = 350 s, 75 µL of 10% Triton X-100 solution was introduced to fully disrupt the membrane, allowing for the measurement of maximum fluorescence quenching.

For pre-incorporation: A POPC lipid film was first prepared from a chloroform stock using a rotary evaporator and subsequently dried under vacuum for at least 4 hours. The dried lipid mass was then weighed to determine the amount of transporters required. Each transporter was dissolved in methanol, and the appropriate volume was added to the lipid film to achieve a final ratio of 5 mol% relative to the lipid content. The mixture was vortexed for 1 minute and subjected to rotary evaporation to reform the lipid film, followed by an additional vacuum-drying step of at least 4 hours. To rehydrate the lipid–transporter film, the sample was vortexed with lucigenin

buffer (1 mM lucigenin, 5 mM HEPES, pH 7.4, 225 mM $NaNO_3$). The resulting suspension underwent nine freeze–thaw cycles, alternating between liquid nitrogen and lukewarm water. After resting at room temperature for 30 minutes, the sample was extruded 25 times through a 100 nm polycarbonate membrane (Nucleopore) using an Avanti mini extruder (Avanti Polar Lipids, Inc.). Unencapsulated lucigenin was removed by size-exclusion chromatography using a Sephadex G-25 (medium) column. The purified liposome stock was diluted in external buffer (225 mM $NaNO_3$, 5 mM HEPES, pH 7.4) to a final lipid concentration of 0.5 mM. Approximately 3 mL of lucigenin-loaded liposomes (0.5 mM total lipid concentration) were transferred into a glass fluorescence cuvette containing a small magnetic stir bar. The cuvette was placed inside the fluorometer, and stirring was initiated at maximum speed, continuing throughout the experiment. Data acquisition began with excitation and emission wavelengths set at 430 nm and 505 nm, respectively. At t = 10 s, 75 µL of a 1 M NaCl stock solution was added to the cuvette to achieve a final concentration of 25 mM NaCl. At t = 350 s, 75 µL of 10% Triton X-100 was introduced to achieve maximal fluorescence quenching.

Data processing: The time scale of the raw kinetic trace was adjusted to ensure chloride transport initiation was set at time 0. This reference point was defined as 1 second after the addition of NaCl. The corrected kinetic trace was then transformed into a normalized fluorescence ratio (F_o/F) using the equation:

$$Normalized\frac{F_0}{F} = \frac{\frac{F_0}{F} - \frac{F_0}{F_0}}{\frac{F_0}{F_{final}} - \frac{F_0}{F_0}} = \frac{\frac{F_0}{F} - 1}{\frac{F_0}{F_{final}} - 1}$$

where F represents the recorded fluorescence intensity at any given time point, F_o is the fluorescence intensity at time 0 (1 second post-NaCl addition), and F_{final} corresponds to the intensity after Triton X-100 addition.

Deliverability calculation: To assess deliverability, initial rates from all individual repeats in both pre-incorporation and external addition assays were obtained by fitting the transport curves in OriginPro using the function that best matched each compound's profile (linear fit for weakly active compounds; exponential decay or asymptotic functions for most other compounds). Deliverability was quantified as the ratio of the initial transport rate in the external addition assay to the rate obtained in the pre-incorporation assay and the calculated values are mentioned in **Table S8**. A deliverability value close to 1 indicates that the compound has good deliverability and does not exhibit solubility-related limitations, while values close to 0 indicate problems with solubility. Only compound **2f** showed deliverability problems. The normalized fluorescence data, comparisons between pre-incorporated and externally added conditions, and fitted initial-rate plots from

all individual repeats (from both external addition and pre-incorporation experiments) are shown in **Figure S41-Figure S52**.

Table S8. Initial rate of chloride transport measured using the lucigenin assay for both external addition and pre-incorporation of the transporters, as well as the resulting deliverability factor.

Compound	Initial rate (external rate), s^{-1}	Initial rate (pre-incorporated), s^{-1}	Deliverability[a]
**1a**	0.00022 ± 0.00008	0.00029 ± 0.00017	-[b]
**1b**	0.00073 ± 0.00017	0.00054 ± 0.00024	-[b]
**1c**	0.00042 ± 0.00008	0.00013 ± 0.00007	-[b]
**1d**	0.00027 ± 0.00001	0.00020 ± 0.00012	-[b]
**1e**	0.00065 ± 0.00006	0.00023 ± 0.00009	-[b]
**1f**	0.0069 ± 0.0014	0.0058 ± 0.0014	1.18
**2a**	0.0074 ± 0.0013	0.0055 ± 0.0014	1.35
**2b**	0.0073 ± 0.0028	0.0084 ± 0.0012	0.88
**2c**	0.0022 ± 0.0003	0.0024 ± 0.0005	0.90
**2d**	0.00040 ± 0.00003	0.00038 ± 0.00015	-[b]
**2e**	0.0043 ± 0.0012	0.0044 ± 0.0002	0.97
**2f**	0.00050 ± 0.00015	0.00561 ± 0.00051	0.09

[a] Calculated as the ratio of the initial rate of external addition to the initial rate of pre-incorporation. [b] Due to the very low rates of transport (< 0.001 s^{-1}), the deliverability cannot be calculated accurately.

(a)

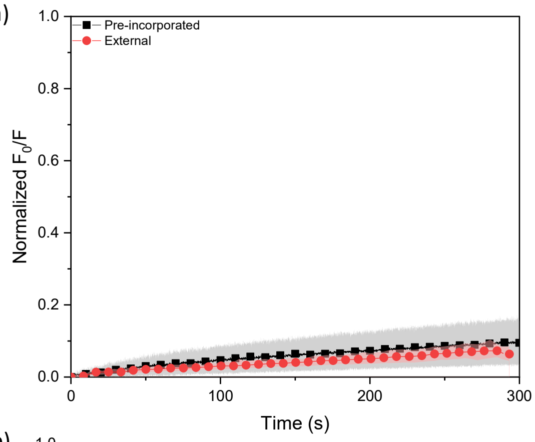

(b)

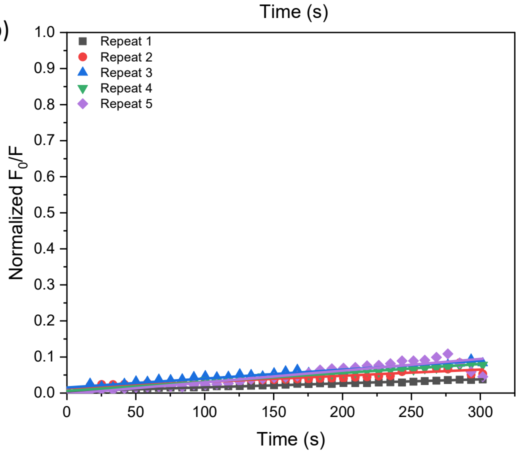

(c)

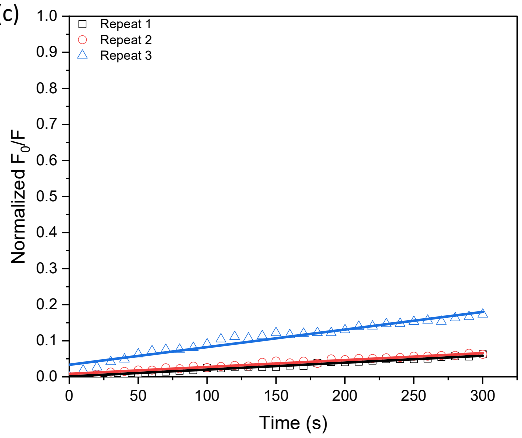

(d)

Equation	y = a + b*x				
Plot	Repeat 1	Repeat 2	Repeat 3	Repeat 4	Repeat 5
Weight	No Weighting				
Intercept	0.00492 ± 3.44631E-4	0.01376 ± 0.00169	0.01541 ± 0.00134	0.00633 ± 5.64409E-4	-0.00264 ± 0.00283
Slope	1.09879E-4 ± 1.97344E-6	1.71254E-4 ± 9.65081E-6	2.47224E-4 ± 7.67071E-6	2.47551E-4 ± 3.23194E-6	3.24292E-4 ± 1.62097E-5
Residual Sum of Squares	1.57437E-4	0.00377	0.00238	4.22267E-4	0.01062
Pearson's r	0.98874	0.90333	0.96748	0.994	0.92159
R-Square (COD)	0.97761	0.81601	0.93602	0.98804	0.84933
Adj. R-Square	0.9773	0.81342	0.93512	0.98787	0.84721

(e)

Equation	y = a + b*x		
Plot	Repeat 1	Repeat 2	Repeat 3
Weight	No Weighting		
Intercept	0.00102 ± 7.85E-5	0.00809 ± 1.23E-4	0.0334 ± 3.20E-4
Slope	1.91E-4 ± 4.53E-7	1.95E-4 ± 7.12E-7	4.88E-4 ± 1.85E-6
Residual Sum of Squares	0.0139	0.0342	0.230
Pearson's r	0.992	0.981	0.979
R-Square (COD)	0.983	0.961	0.959
Adj. R-Square	0.983	0.961	0.959

Figure S41. Chloride transport activity of compound **1a** (5 mol%) under different delivery conditions. (a) Normalized *F_0/F* traces showing chloride transport upon external addition of the transporter to pre-formed liposomes (red) and pre-incorporation within the lipid bilayer during liposome preparation (black). (b) Individual repeats of external addition; lines represent linear fits. (c) Individual repeats of pre-incorporation experiments, lines represent linear fits. (d, e) Summary tables of the fitting results for (d) external addition and (e) pre-incorporation. Initial rates correspond to the slopes.

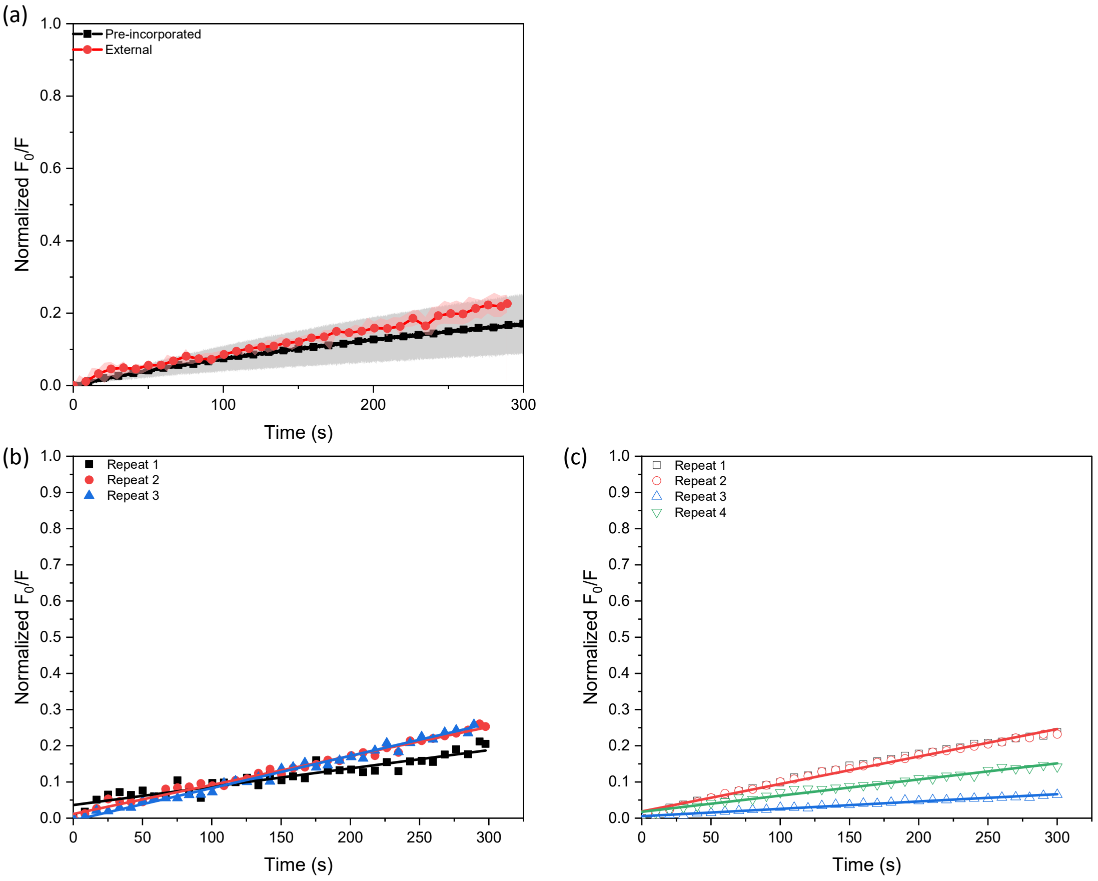

(d)

Equation	y = a + b*x		
Plot	Repeat 1	Repeat 2	Repeat 3
Weight	No Weighting		
Intercept	0.0363 ± 0.00366	0.0117 ± 0.00196	-0.00607 ± 0.00205
Slope	5.05E-4 ± 2.13E-5	8.01E-4 ± 1.14E-5	8.91E-4 ± 1.23E-5
Residual Sum of Squares	0.0173	0.00495	0.00514
Pearson's r	0.943	0.993	0.994
R-Square (COD)	0.890	0.986	0.987
Adj. R-Square	0.888	0.986	0.987

(e)

Equation	y = a + b*x			
Plot	Repeat 1	Repeat 2	Repeat 3	Repeat 4
Weight	No Weighting			
Intercept	0.01843 ± 2.59217E-4	0.01851 ± 2.67034E-4	0.00533 ± 9.8107E-5	0.01761 ± 2.37855E-4
Slope	7.68221E-4 ± 1.49609E-6	7.59341E-4 ± 1.54121E-6	2.03398E-4 ± 5.66232E-7	4.46203E-4 ± 1.3728E-6
Residual Sum of Squares	0.15119	0.16044	0.02166	0.12729
Pearson's r	0.99436	0.99388	0.98858	0.9861
R-Square (COD)	0.98875	0.9878	0.97729	0.9724
Adj. R-Square	0.98875	0.98779	0.97728	0.97239

Figure S42. Chloride transport activity of compound **1b** (5 mol%) under different delivery conditions. (a) Normalized F_0/F traces showing chloride transport upon external addition of the transporter to pre-formed liposomes (red) and pre-incorporation within the lipid bilayer during liposome preparation (black). (b) Individual repeats of external addition; lines represent linear fits. (c) Individual repeats of pre-incorporation experiments, lines represent linear fits. (d, e) Summary tables of the fitting results for (d) external addition and (e) pre-incorporation. Initial rates correspond to the slopes.

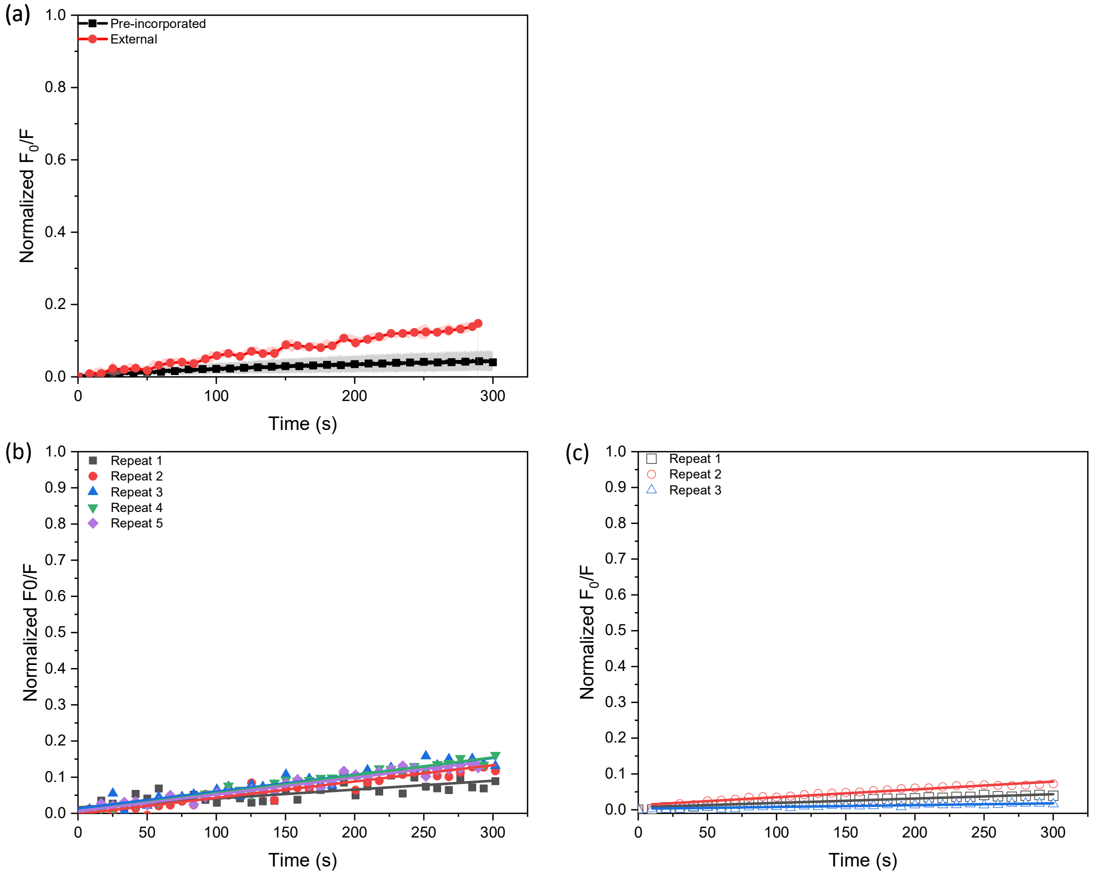

(d)

Equation	y = a + b*x				
Plot	Repeat 1	Repeat 2	Repeat 3	Repeat 4	Repeat 5
Weight	No Weighting				
Intercept	0.01639 ± 0.00352	-0.0021 ± 0.00266	0.01349 ± 0.00279	0.00601 ± 0.00183	0.00582 ± 0.00212
Slope	2.4616E-4 ± 2.01394E-5	4.50556E-4 ± 1.52254E-5	4.65341E-4 ± 1.59661E-5	4.96007E-4 ± 1.04783E-5	4.65204E-4 ± 1.26673E-5
Residual Sum of Squares	0.0164	0.00937	0.01031	0.00444	0.00548
Pearson's r	0.82332	0.96177	0.96066	0.98452	0.97571
R-Square (COD)	0.67785	0.925	0.92286	0.96929	0.952
Adj. R-Square	0.67332	0.92395	0.92178	0.96885	0.9513

(e)

Equation	y = a + b*x		
Plot	Repeat 1	Repeat 2	Repeat 3
Weight	No Weighting		
Intercept	0.00701 ± 9.64E-5	0.0131 ± 1.60E-4	0.00285 ± 8.24E-5
Slope	1.21E-4 ± 5.47E-7	2.19E-4 ± 9.06E-7	5.05E-5 ± 4.68E-7
Residual Sum of Squares	0.0176	0.0484	0.0129
Pearson's r	0.972	0.976	0.895
R-Square (COD)	0.944	0.953	0.801
Adj. R-Square	0.944	0.953	0.801

Figure S43. Chloride transport activity of compound **1c** (5 mol%) under different delivery conditions. (a) Normalized F_0/F traces showing chloride transport upon external addition of the transporter to pre-formed liposomes (red) and pre-incorporation within the lipid bilayer during liposome preparation (black). (b) Individual repeats of external addition; lines represent linear fits. (c) Individual repeats of pre-incorporation experiments, lines represent linear fits. (d, e) Summary tables of the fitting results for (d) external addition and (e) pre-incorporation. Initial rates correspond to the slopes.

(a)

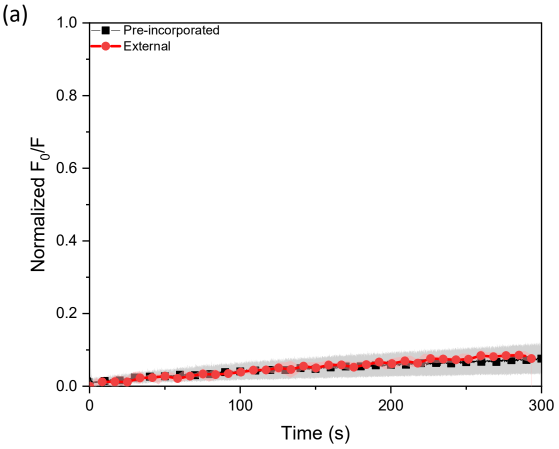

(b)

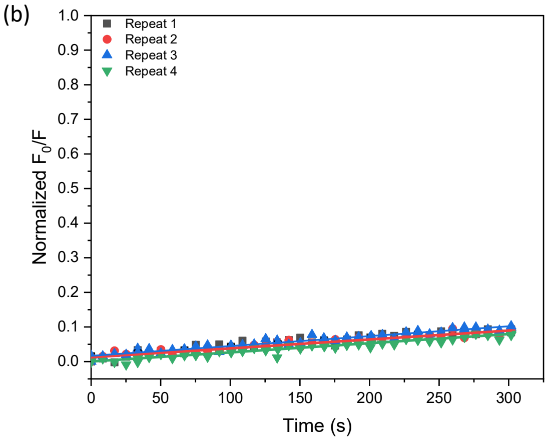

(c)

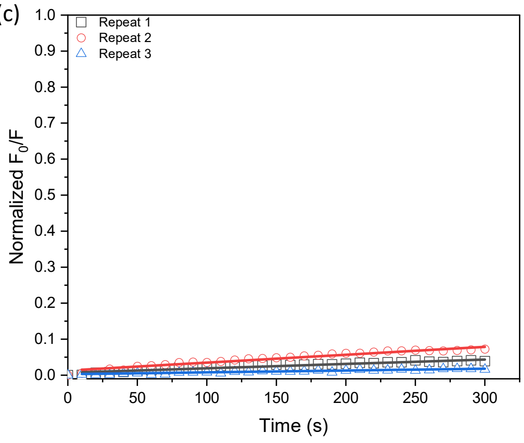

(c)

Equation	y = a + b*x			
Plot	Repeat 1	Repeat 2	Repeat 3	Repeat 4
Weight	No Weighting			
Intercept	0.01506 ± 0.00204	0.01237 ± 0.00169	0.01642 ± 0.00174	2.03904E-4 ± 0.00187
Slope	2.57565E-4 ± 1.1656E-5	2.55828E-4 ± 9.6763E-6	2.85165E-4 ± 9.96125E-6	2.64276E-4 ± 1.07277E-5
Residual Sum of Squares	0.00549	0.00379	0.00401	0.00465
Pearson's r	0.93437	0.95278	0.95931	0.94618
R-Square (COD)	0.87305	0.90779	0.92027	0.89526
Adj. R-Square	0.87126	0.90649	0.91915	0.89379

(d)

Equation	y = a + b*x			
Plot	Repeat 1	Repeat 2	Repeat 3	Repeat 4
Weight	No Weighting			
Intercept	0.00347 ± 8.54785E-5	0.03899 ± 2.33851E-4	0.01156 ± 1.15185E-4	0.01704 ± 2.05885E-4
Slope	3.69585E-5 ± 4.93346E-7	2.29168E-4 ± 1.34969E-6	1.74644E-4 ± 6.64798E-7	3.64209E-4 ± 1.18828E-6
Residual Sum of Squares	0.01644	0.12304	0.02985	0.09537
Pearson's r	0.8073	0.95172	0.97896	0.98441
R-Square (COD)	0.65173	0.90578	0.95835	0.96906
Adj. R-Square	0.65161	0.90575	0.95834	0.96905

Figure S44. Chloride transport activity of compound **1d** (5 mol%) under different delivery conditions. (a) Normalized F_0/F traces showing chloride transport upon external addition of the transporter to pre-formed liposomes (red) and pre-incorporation within the lipid bilayer during liposome preparation (black). (b) Individual repeats of external addition; lines represent linear fits. (c) Individual repeats of pre-incorporation experiments, lines represent linear fits. (d, e) Summary tables of the fitting results for (d) external addition and (e) pre-incorporation. Initial rates correspond to the slopes.

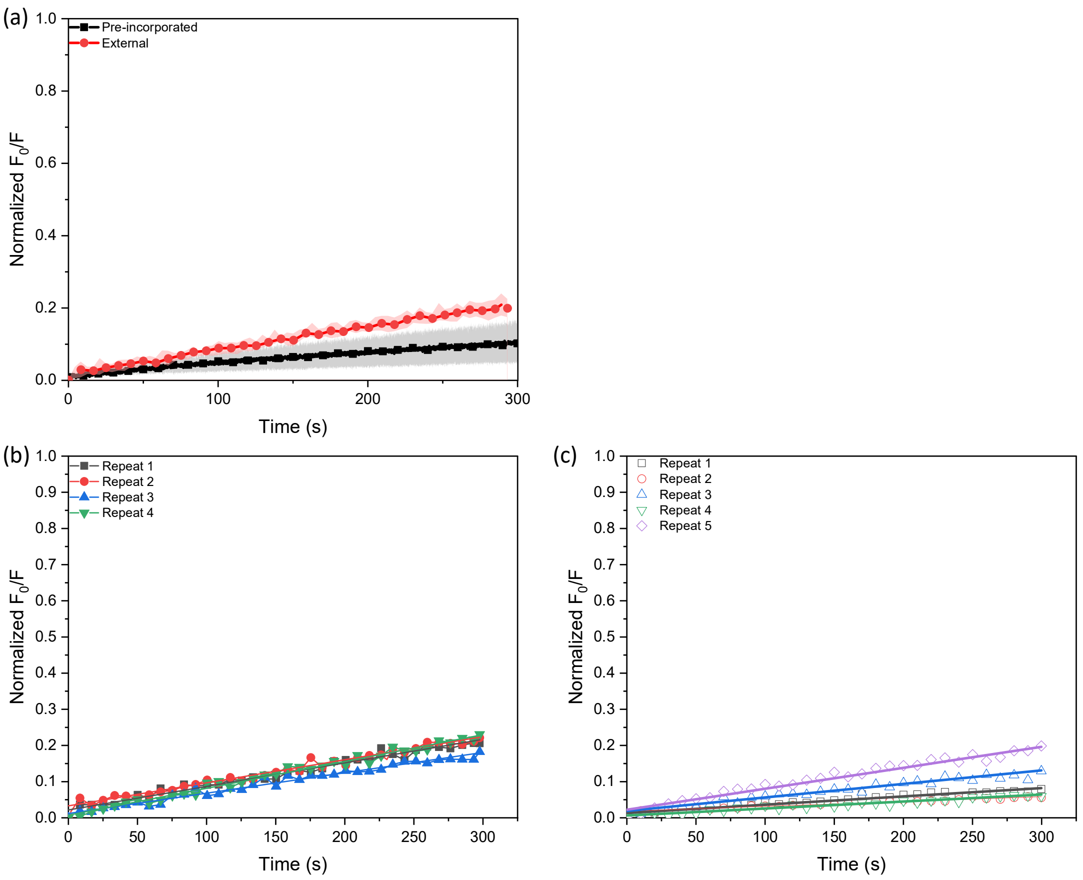

(d)

Equation	y = a + b*x			
Plot	Repeat 1	Repeat 2	Repeat 3	Repeat 4
Weight	No Weighting			
Intercept	0.0218 ± 0.00208	0.0330 ± 0.00240	0.0118 ± 0.00187	0.00736 ± 0.00194
Slope	6.50E-4 ± 1.21E-5	6.38E-4 ± 1.39E-5	5.64E-4 ± 1.08E-5	7.42E-4 ± 1.12E-5
Residual Sum of Squares	0.00556	0.00742	0.00449	0.00483
Pearson's r	0.988	0.984	0.987	0.992
R-Square (COD)	0.976	0.968	0.975	0.984
Adj. R-Square	0.976	0.967	0.974	0.984

(e)

Equation	y = a + b*x				
Plot	Repeat 1	Repeat 2	Repeat 3	Repeat 4	Repeat 5
Weight	No Weighting				
Intercept	0.0132 ± 1.54E-4	0.0184 ± 1.27E-4	0.0190 ± 2.64E-4	0.00641 ± 1.51E-4	0.0222 ± 3.39E-4
Slope	2.30E-4 ± 8.87E-7	1.33E-4 ± 7.36E-7	3.74E-4 ± 1.52E-6	1.93E-4 ± 8.73E-7	5.81E-4 ± 1.96E-6
Residual Sum of Squares	0.0529	0.0365	0.156	0.0512	0.259
Pearson's r	0.978	0.957	0.976	0.971	0.983
R-Square (COD)	0.957	0.916	0.953	0.942	0.967
Adj. R-Square	0.957	0.916	0.953	0.942	0.967

Figure S45. Chloride transport activity of compound **1e** (5 mol%) under different delivery conditions. (a) Normalized F_0/F traces showing chloride transport upon external addition of the transporter to pre-formed liposomes (red) and pre-incorporation within the lipid bilayer during liposome preparation (black). (b) Individual repeats of external addition; lines represent linear fits. (c) Individual repeats of pre-incorporation experiments, lines represent linear fits. (d, e) Summary tables of the fitting results for (d) external addition and (e) pre-incorporation. Initial rates correspond to the slopes.

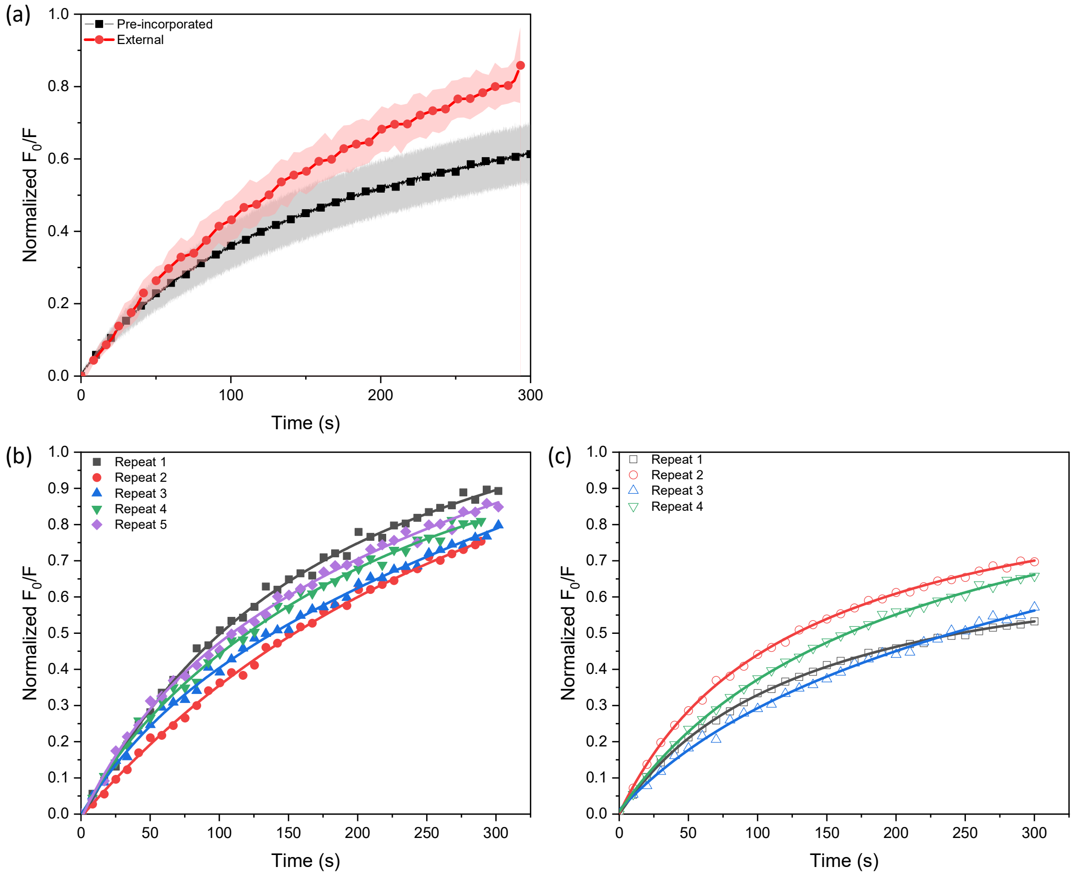

(d)

Model	ExpDec2				
Equation	y = A1*exp(-x/t1) + A2*exp(-x/t2) + y0				
Plot	Repeat 1	Repeat 2	Repeat 3	Rpeat 4	Repeat 5
y0	2.91E+3 ± 6.22E+4	2.41 ± 545	1.61 ± 1.05	1.06 ± 0.0492	1.29 ± 0.324
A1	-0.731 ± 0.0747	-0.877 ± 10.5	-0.217 ± 0.170	-0.0701 ± 0.0312	-0.239 ± 0.159
t1	109 ± 11.6	218 ± 1.04E+3	57.0 ± 31.0	22.2 ± 13.5	48.2 ± 23.2
A2	-2.91E+3 ± 6.22E+4	-1.54 ± 535	-1.40 ± 0.875	-0.999 ± 0.0228	-1.06 ± 0.168
t2	3.80E+6 ± 8.12E+7	3.64E+3 ± 1.52E+6	567 ± 681	207 ± 24.6	332 ± 215
Reduced Chi-Sqr	1.54E-4	1.09E-4	1.54E-4	1.62E-4	2.15E-4
R-Square (COD)	0.998	0.998	0.997	0.997	0.996
Adj. R-Square	0.998	0.998	0.997	0.997	0.996

(e)

Model	ExpDec2			
Equation	y = A1*exp(-x/t1) + A2*exp(-x/t2) + y0			
Plot	Repeat 1	Repeat 2	Repeat 3	Repeat 4
y0	0.624 ± 0.00333	0.836 ± 0.00759	1.09 ± 0.153	0.888 ± 0.0164
A1	-0.130 ± 0.00845	-0.218 ± 0.0146	-0.178 ± 0.0378	-0.170 ± 0.0217
t1	44.0 ± 1.80	45.5 ± 1.95	72.8 ± 8.73	59.0 ± 4.14
A2	-0.493 ± 0.00553	-0.616 ± 0.00786	-0.897 ± 0.116	-0.712 ± 0.00671
t2	177 ± 4.69	198 ± 8.42	551 ± 163	261 ± 17.5
Reduced Chi-Sqr	1.28E-5	3.95E-5	5.69E-5	2.68E-5
R-Square (COD)	0.999	0.999	0.998	0.999
Adj. R-Square	0.999	0.999	0.998	0.999

Figure S46. Chloride transport activity of compound **1f** (5 mol%) under different delivery conditions. (a) Normalized F_0/F traces showing chloride transport upon external addition of the transporter to pre-formed liposomes (red) and pre-incorporation within the lipid bilayer during liposome preparation (black). (b) Individual repeats of external addition; lines represent non-linear fits to an ExpDec2 function. (c) Individual repeats of pre-incorporation experiments, lines represent non-linear fits to an ExpDec2 function. (d, e) Summary tables of the fitting results for (d) external addition and (e) pre-incorporation. Initial rates correspond to $k_{ini} = -(A1/t1) - (A2/t2)$.

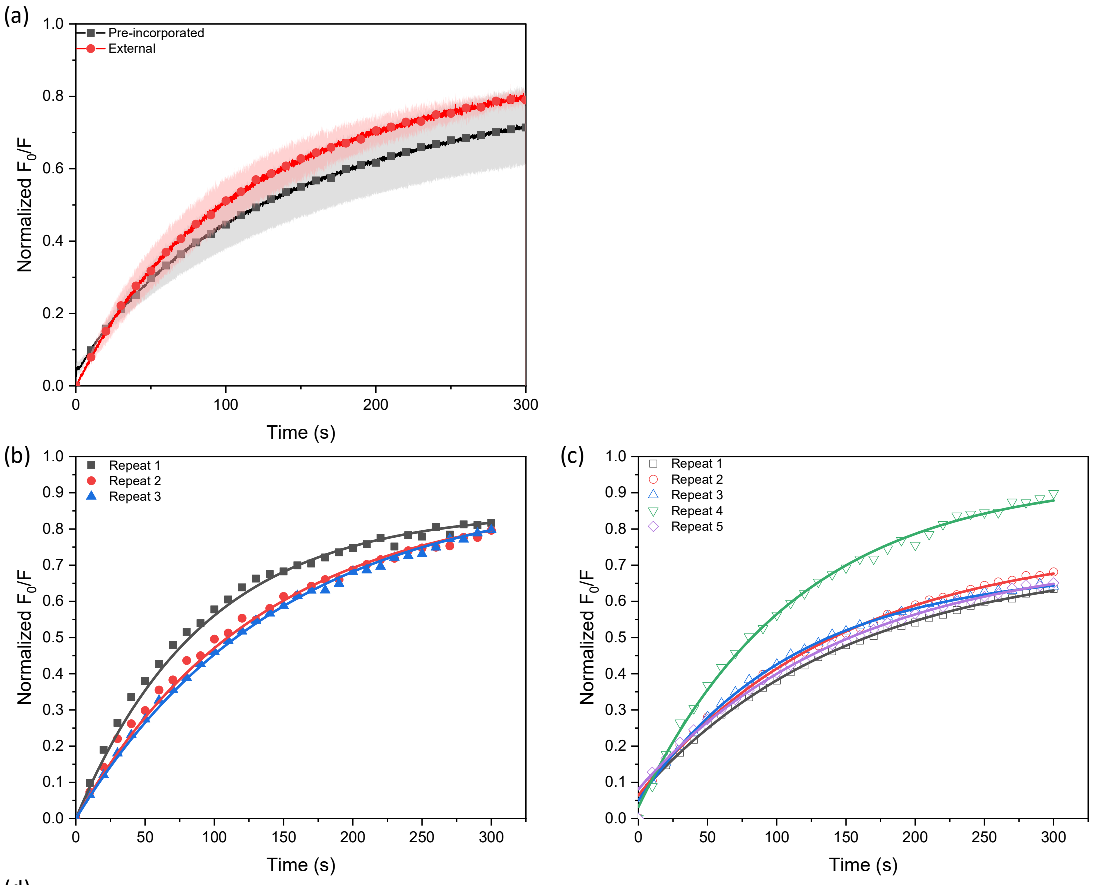

(d)

Model	Asymptotic1		
Equation	y = a-b*c^x		
Plot	Repeat 1	Repeat 2	Repeat 3
a	0.851 ± 9.40E-4	0.888 ± 0.00188	0.914 ± 0.00130
b	0.851 ± 0.00109	0.889 ± 0.00157	0.914 ± 0.00107
c	0.989 ± 4.05E-5	0.992 ± 4.11E-5	0.993 ± 2.34E-5
Reduced Chi-Sqr	2.36E-4	3.02E-4	1.02E-4
R-Square (COD)	0.995	0.993	0.998
Adj. R-Square	0.995	0.993	0.998

(e)

Model	Asymptotic1				
Equation	y = a-b*c^x				
Plot	Repeat 1	Repeat 2	Repeat 3	Repeat 4	Repeat 5
a	0.719 ± 6.55E-4	0.764 ± 7.61E-4	0.688 ± 3.75E-4	0.944 ± 0.00104	0.738 ± 0.00112
b	0.656 ± 5.36E-4	0.700 ± 6.25E-4	0.638 ± 3.45E-4	0.913 ± 9.50E-4	0.657 ± 9.20E-4
c	0.993 ± 1.56E-5	0.993 ± 1.82E-5	0.991 ± 1.52E-5	0.991 ± 2.89E-5	0.993 ± 2.67E-5
Reduced Chi-Sqr	2.34E-5	3.65E-5	2.07E-5	1.54E-4	6.85E-5
R-Square (COD)	0.999	0.999	0.999	0.997	0.997
Adj. R-Square	0.999	0.999	0.999	0.997	0.997

Figure S47. Chloride transport activity of compound **2a** (0.5 mol%) under different delivery conditions. (a) Normalized F_0/F traces showing chloride transport upon external addition of the transporter to pre-formed liposomes (red) and pre-incorporation within the lipid bilayer during liposome preparation (black). (b) Individual repeats of external addition; lines represent non-linear fits to an Asymptotic1 function. (c) Individual repeats of pre-incorporation experiments, lines represent non-linear fits to an Asymptotic1 function. (d, e) Summary tables of the fitting results for (d) external addition and (e) pre-incorporation. Initial rates correspond to $k_{ini} = -b*ln(c)$.

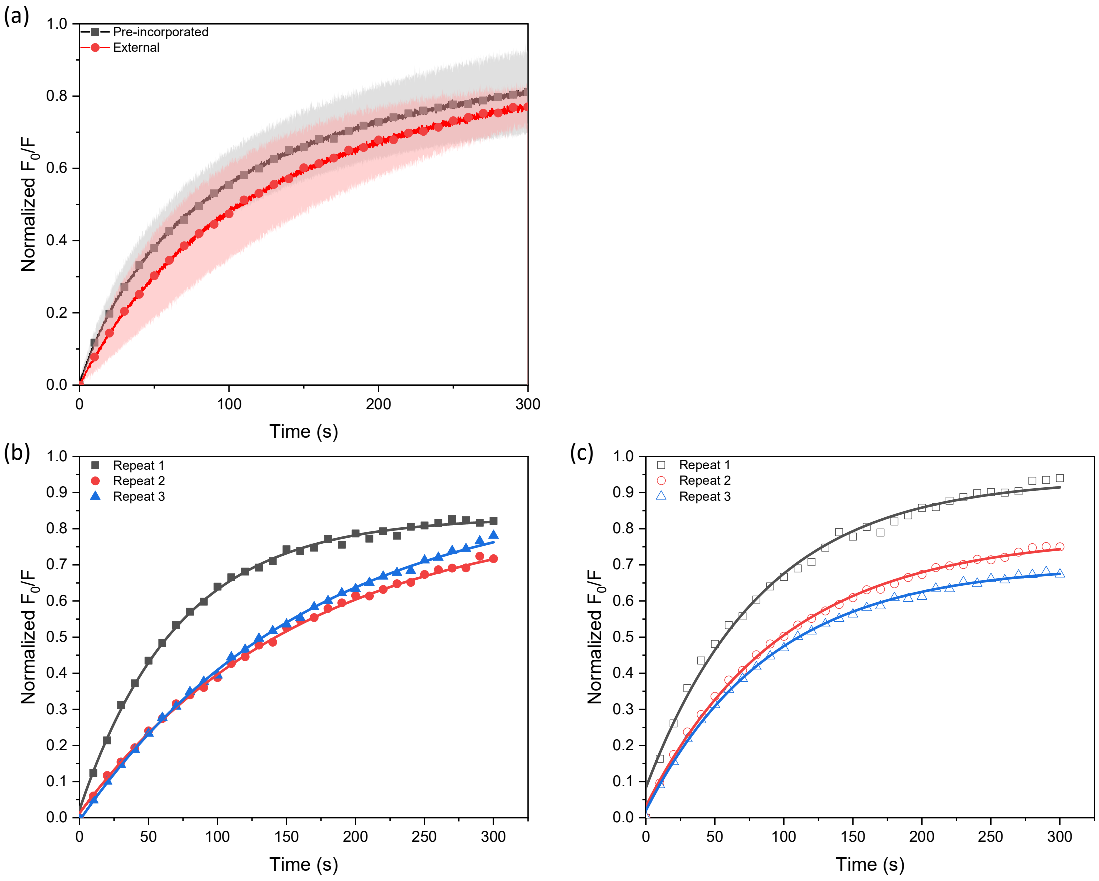

(d)

Model	Asymptotic1		
Equation	y = a-b*c^x		
Plot	Repeat 1	Repeat 2	Repeat 3
a	0.831 ± 3.96E-4	0.847 ± 0.00119	0.914 ± 9.54E-4
b	0.809 ± 6.94E-4	0.834 ± 9.80E-4	0.922 ± 7.87E-4
c	0.986 ± 2.89E-5	0.994 ± 1.96E-5	0.994 ± 1.36E-5
Reduced Chi-Sqr	8.41E-5	5.87E-5	3.44E-5
R-Square (COD)	0.998	0.998	0.999
Adj. R-Square	0.998	0.998	0.999

(e)

Model	Asymptotic1		
Equation	y = a-b*c^x		
Plot	Repeat 1	Repeat 2	Repeat 3
a	0.940 ± 9.82E-4	0.780 ± 5.54E-4	0.700 ± 3.74E-4
b	0.857 ± 0.00131	0.748 ± 5.83E-4	0.680 ± 4.52E-4
c	0.988 ± 4.96E-5	0.990 ± 2.38E-5	0.989 ± 2.12E-5
Reduced Chi-Sqr	3.34E-4	6.62E-5	4.05E-5
R-Square (COD)	0.993	0.998	0.999
Adj. R-Square	0.993	0.998	0.999

Figure S48. Chloride transport activity of compound **2b** (0.5 mol%) under different delivery conditions. (a) Normalized F_0/F traces showing chloride transport upon external addition of the transporter to pre-formed liposomes (red) and pre-incorporation within the lipid bilayer during liposome preparation (black). (b) Individual repeats of external addition; lines represent non-linear fits to an Asymptotic1 function. (c) Individual repeats of pre-incorporation experiments, lines represent non-linear fits to an Asymptotic1 function. (d, e) Summary tables of the fitting results for (d) external addition and (e) pre-incorporation. Initial rates correspond to $k_{ini} = -b*ln(c)$.

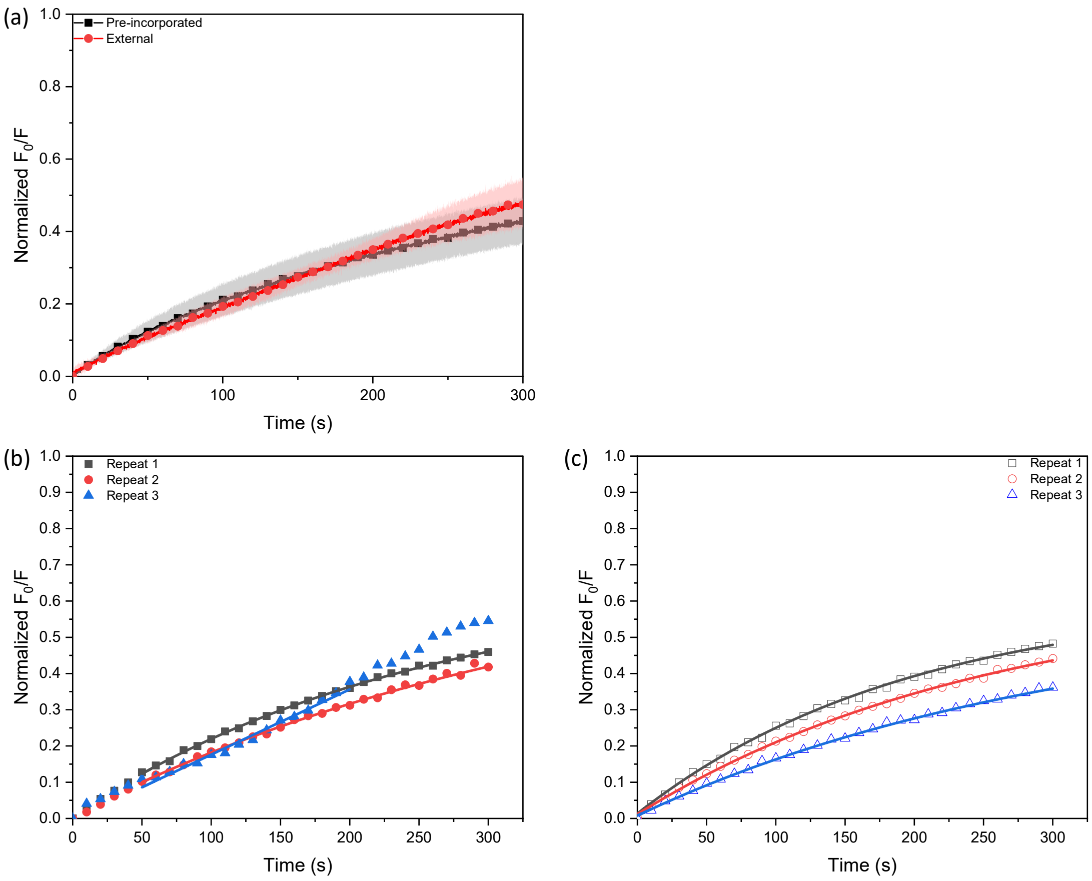

(d)

Model	Asymptotic1		
Equation	y = a-b*c^x		
Plot	Repeat 1	Repeat 2	Repeat 3
a	0.661 ± 0.00245	0.754 ± 0.00618	63.9 ± 338
b	0.655 ± 0.00186	0.748 ± 0.00552	63.9 ± 338
c	0.996 ± 2.83E-5	0.997 ± 3.47E-5	1.000 ± 1.50E-4
Reduced Chi-Sqr	1.86E-5	2.60E-5	7.68E-5
R-Square (COD)	0.998	0.997	0.988
Adj. R-Square	0.998	0.997	0.988

(e)

Model	Asymptotic1		
Equation	y = a-b*c^x		
Plot	Repeat 1	Repeat 2	Repeat 3
a	0.61049 ± 0.00114	0.62664 ± 0.0015	0.57807 ± 0.00182
b	0.59695 ± 9.71761E-4	0.61522 ± 0.00135	0.57032 ± 0.0017
c	0.99497 ± 1.88722E-5	0.9961 ± 1.63123E-5	0.99683 ± 1.58302E-5
Reduced Chi-Sqr	2.53717E-5	1.66363E-5	1.09714E-5
R-Square (COD)	0.99855	0.99887	0.99891
Adj. R-Square	0.99855	0.99887	0.99891

Figure S49. Chloride transport activity of compound **2c** (5 mol%) under different delivery conditions. (a) Normalized F_0/F traces showing chloride transport upon external addition of the transporter to pre-formed liposomes (red) and pre-incorporation within the lipid bilayer during liposome preparation (black). (b) Individual repeats of external addition; lines represent non-linear fits to an Asymptotic1 function. (c) Individual repeats of pre-incorporation experiments, lines represent non-linear fits to an Asymptotic1 function. (d, e) Summary tables of the fitting results for (d) external addition and (e) pre-incorporation. Initial rates correspond to $k_{ini} = -b*ln(c)$.

(a)

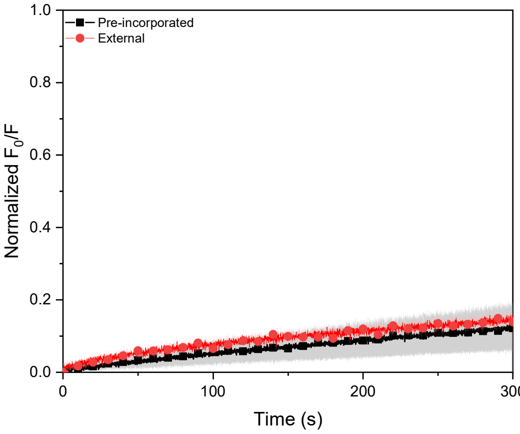

(b)

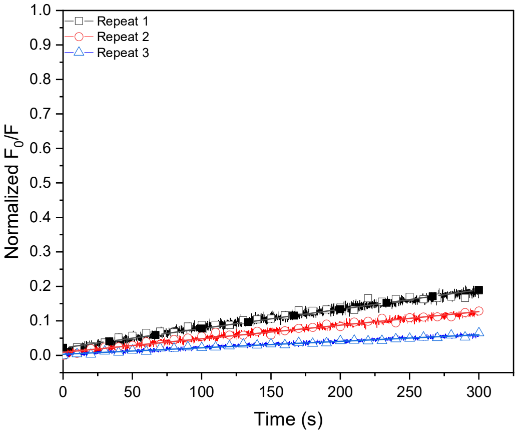

(c)

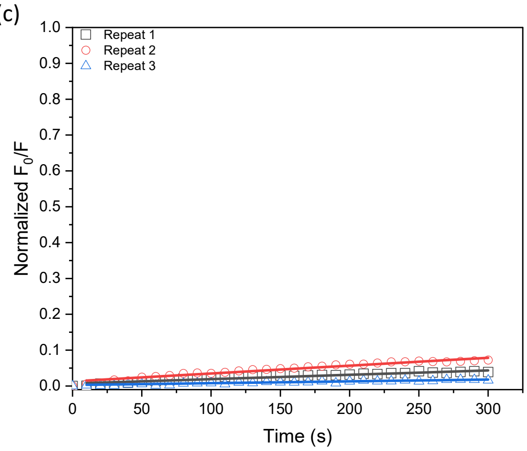

(d)

Equation	y = a + b*x		
Plot	Repeat 1	Repeat 2	Repeat 3
Weight	No Weighting		
Intercept	0.03567 ± 4.47613E-4	0.0303 ± 3.71087E-4	0.02984 ± 3.92245E-4
Slope	3.84747E-4 ± 2.58343E-6	3.72512E-4 ± 2.14176E-6	4.44596E-4 ± 2.26387E-6
Residual Sum of Squares	0.4508	0.30984	0.34618
Pearson's r	0.93856	0.95384	0.96325
R-Square (COD)	0.88089	0.9098	0.92785
Adj. R-Square	0.88085	0.90977	0.92783

(e)

Equation	y = a + b*x		
Plot	Repeat 1	Repeat 2	Repeat 3
Weight	No Weighting		
Intercept	0.02187 ± 3.06264E-4	0.00894 ± 2.00312E-4	0.00319 ± 1.1538E-4
Slope	5.59494E-4 ± 1.76762E-6	3.92473E-4 ± 1.15612E-6	1.87839E-4 ± 6.65927E-7
Residual Sum of Squares	0.21104	0.09028	0.02995
Pearson's r	0.98536	0.98724	0.98167
R-Square (COD)	0.97094	0.97464	0.96368
Adj. R-Square	0.97093	0.97463	0.96366

Figure S50. Chloride transport activity of compound **2d** (5 mol%) under different delivery conditions. (a) Normalized F_0/F traces showing chloride transport upon external addition of the transporter to pre-formed liposomes (red) and pre-incorporation within the lipid bilayer during liposome preparation (black). (b) Individual repeats of external addition; lines represent linear fits. (c) Individual repeats of pre-incorporation experiments, lines represent linear fits. (d, e) Summary tables of the fitting results for (d) external addition and (e) pre-incorporation. Initial rates correspond to the slopes.

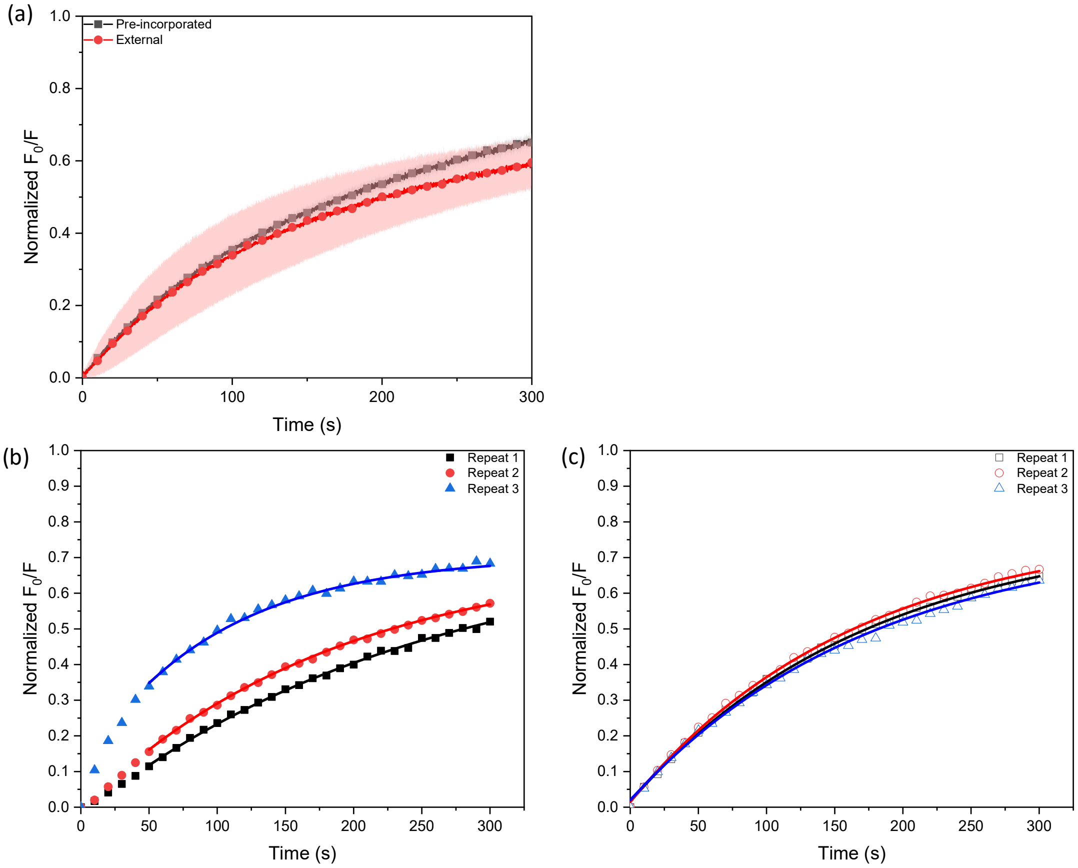

(d)

Model	ExpDec1		
Equation	y = A1*exp(-x/t1) + y0		
Plot	Repeat 1	Repeat 2	Repeat 3
y0	0.747 ± 0.00242	0.714 ± 0.00126	0.705 ± 6.55E-4
A1	-0.771 ± 0.00181	-0.722 ± 7.43E-4	-0.591 ± 0.00142
t1	247 ± 1.52	187 ± 0.784	99.3 ± 0.511
Reduced Chi-Sqr	2.05E-5	1.61E-5	4.35E-5
R-Square (COD)	0.998	0.999	0.995
Adj. R-Square	0.998	0.999	0.995

(e)

Model	ExpDec1		
Equation	y = A1*exp(-x/t1) + y0		
Plot	Repeat 1	Repeat 2	Repeat 3
y0	0.787 ± 0.00107	0.790 ± 0.00105	0.767 ± 0.00136
A1	-0.771 ± 8.90E-4	-0.775 ± 8.69E-4	-0.748 ± 0.00114
t1	175 ± 0.517	167 ± 0.497	177 ± 0.681
Reduced Chi-Sqr	3.54E-5	4.14E-5	5.52E-5
R-Square (COD)	0.999	0.999	0.998
Adj. R-Square	0.999	0.999	0.998

Figure S51. Chloride transport activity of compound **2e** (5 mol%) under different delivery conditions. (a) Normalized F_0/F traces showing chloride transport upon external addition of the transporter to pre-formed liposomes (red) and pre-incorporation within the lipid bilayer during liposome preparation (black). (b) Individual repeats of external addition; lines represent non-linear fits to an ExpDec1 function. (c) Individual repeats of pre-incorporation experiments, lines represent non-linear fits to an ExpDec1 function. (d, e) Summary tables of the fitting results for (d) external addition and (e) pre-incorporation. Initial rates correspond to $k_{ini} = -(A1/t1)$.

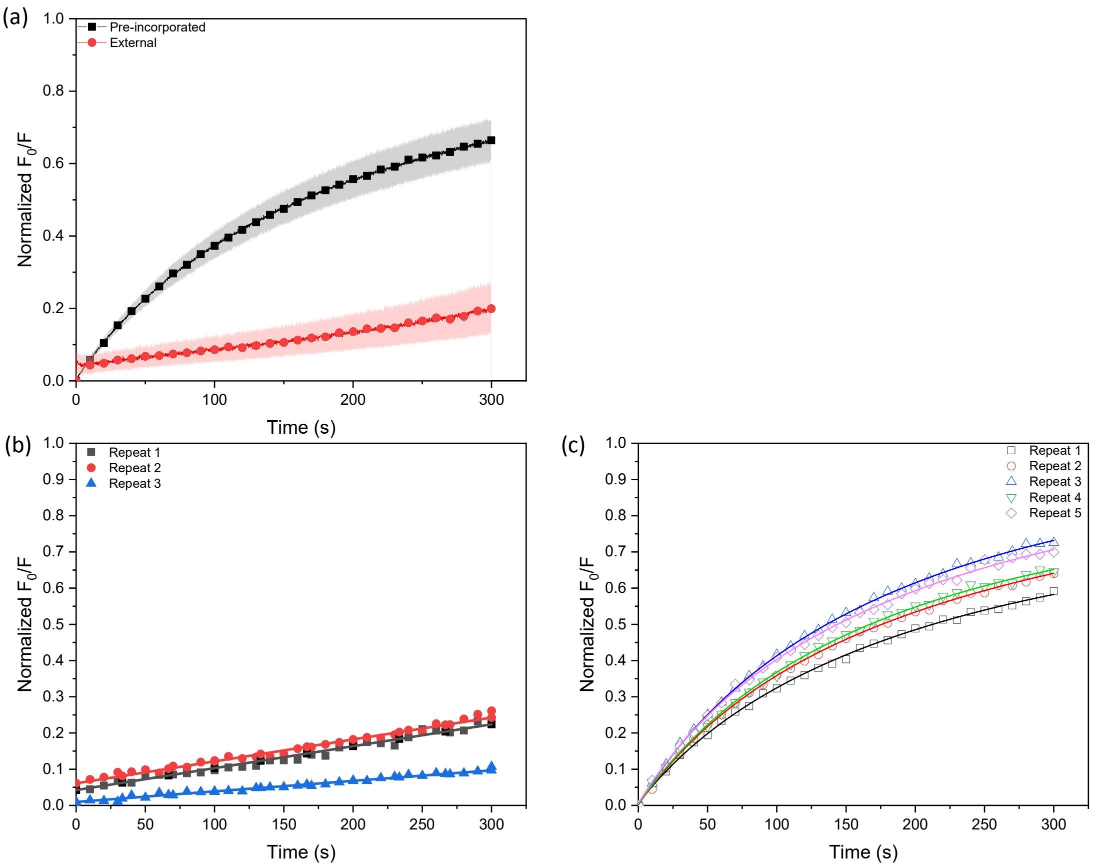

(d)

Equation	y = a + b*x		
Plot	Repeat 1	Repeat 2	Repeat 3
Weight	No Weighting		
Intercept	0.0423 ± 2.93E-4	0.0611 ± 2.91E-4	0.00963 ± 1.32E-4
Slope	6.06E-4 ± 1.69E-6	6.05E-4 ± 1.68E-6	2.91E-4 ± 7.63E-7
Residual Sum of Squares	0.193	0.190	0.0393
Pearson's r	0.989	0.989	0.990
R-Square (COD)	0.977	0.977	0.980
Adj. R-Square	0.977	0.977	0.980

(e)

Model	ExpDec2				
Equation	y = A1*exp(-x/t1) + A2*exp(-x/t2) + y0				
Plot	Repeat 1	Repeat 2	Repeat 3	Repeat 4	Repeat 5
y0	0.759 ± 0.011	0.872 ± 0.020	0.923 ± 0.010	0.810 ± 0.007	0.928 ± 0.019
A1	-0.106 ± 0.016	-0.187 ± 0.027	-0.144 ± 0.021	-0.108 ± 0.018	-0.192 ± 0.029
t1	51.759 ± 4.684	64.058 ± 4.721	55.447 ± 4.387	53.937 ± 4.762	59.408 ± 4.769
A2	-0.646 ± 0.006	-0.682 ± 0.008	-0.776 ± 0.012	-0.697 ± 0.011	-0.729 ± 0.011
t2	231.124 ± 11.860	275.208 ± 23.582	213.880 ± 9.637	202.636 ± 7.941	250.363 ± 19.963
Reduced Chi-Sqr	0.000	0.000	0.000	0.000	0.000
R-Square (COD)	0.999	0.999	0.999	0.999	0.999
Adj. R-Square	0.999	0.999	0.999	0.999	0.999

Figure S52. Chloride transport activity of compound **2f** (5 mol%) under different delivery conditions. (a) Normalized F_0/F traces showing chloride transport upon external addition of the transporter to pre-formed liposomes (red) and pre-incorporation within the lipid bilayer during liposome preparation (black). (b) Individual repeats of external addition; lines represent non-linear fits. (c) Individual repeats of pre-incorporation experiments, lines represent non-linear fits to an ExpDec2 function. (d, e) Summary tables of the fitting results for (d) external addition and (e) pre-incorporation. Initial rates correspond to the slope for external addition and $k_{ini} = -(A1/t1) - (A2/t2)$ for pre-incorporation.

S6.2 Effect of Organic Solvent Amount on Transport

Another approach to overcome the poor deliverability of compound **2f** was to increase the proportion of organic solvent in the sample medium. Liposome preparation and data-workup was carried out as described in *Section S6.1* for external addition**.** The only difference was that varying volumes of transporter stock solutions (dissolved in methanol) were added at *t* = 40 s to achieve a final concentration of 5 mol% in each case. To reach this concentration, stock solutions were diluted as the volume of added MeOH was increased. The final MeOH volumes ranged from 7.5 µL (0.25% MeOH) to 200 µL (6.67% MeOH) in a 3 mL cuvette. The experiments were performed with compound **2f**, which showed poor deliverability under aqueous conditions as well as compounds **1e** and **2e**, both of which did not show deliverability issues in the pre-incorporation study. The results are shown in **Figure S53-Figure S54**. For compound **2f**, a large effect of MeOH amount was observed, with the use of 150 µL MeOH even showing higher activity than pre-incorporation experiments. This suggests that even during pre-incorporation, not all of **2f** is pre-incorporated. Furthermore, the addition of 150 µL MeOH alone does not cause any influx of chloride, suggesting that the liposomes are stable under these conditions. In addition, increasing the amount of MeOH for compounds **1e** and **2e** had no effect on the transport of these compounds, suggesting that large amounts of MeOH do not artificially increase the transport ability of anionophores but only overcome deliverability problems. We therefore decided to use 5% MeOH for testing compound **2f** in all subsequent assays.

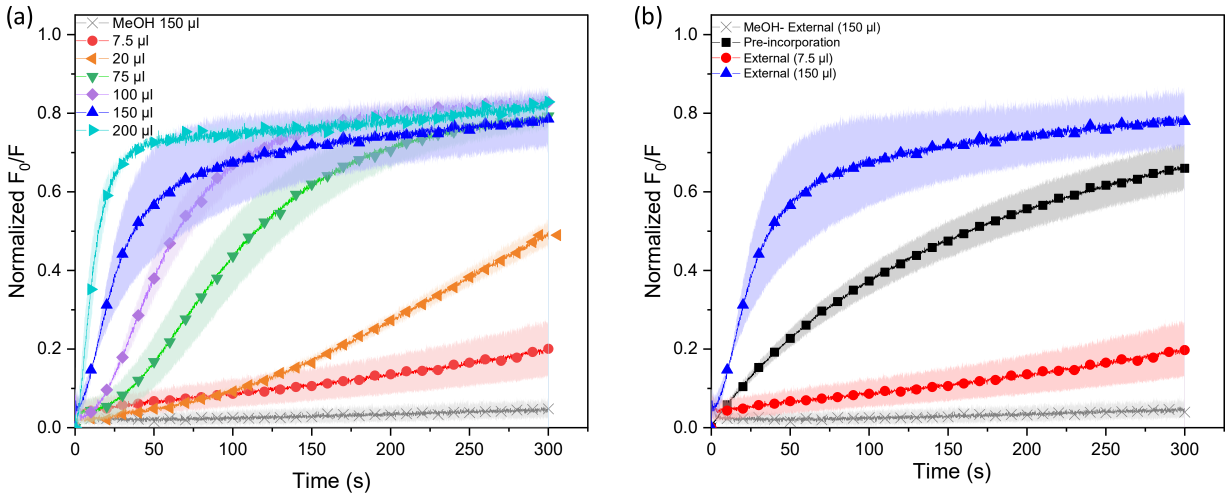

Figure S53. Increasing amount of MeOH to overcome the deliverability of compound **2f** in transport assays. (a) Normalized F_0/F of **2f** following the incremental addition of **2f** stock solution (dissolved in methanol) to achieve a final concentration of 5 mol% in each case. (b) Comparison of pre-incorporation versus external addition of **2f**, using 7.5 µL (the standard condition for fluorescence assays) and 150 µL.

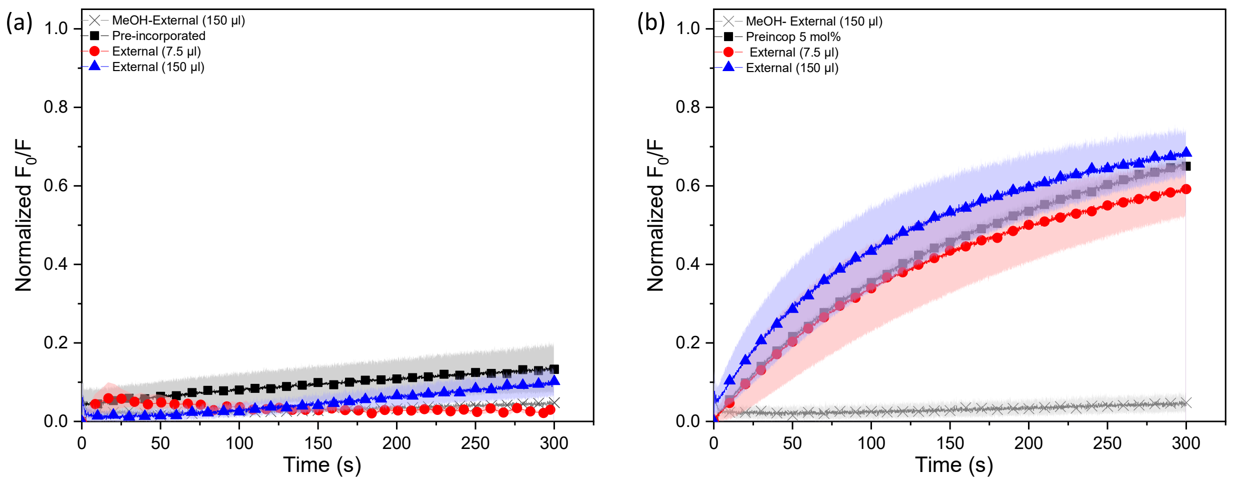

Figure S54. Normalized fluorescence intensity comparing pre-incorporation and external addition at 7.5 µL and 150 µL for compound **1e** (a) and **2e** (b).

S6.3 Hill Plots (*EC_{50}* values)

For the Hill plots, the lucigenin assay was performed as described in *Section S6.1* (external addition) for various concentrations of transporters **1f**, **2c**, **2e**, **2f** —which showed measurable transport at 5 mol%. Hill plots for compounds **2a** and **2b** have been previously reported.[9] For transporter **2f**, assays were carried out under both pre-incorporation conditions and with external addition at an increased solvent volume of 5% MeOH (150 µL). The normalized F_0/F values at 300 s after transporter addition were plotted against transporter concentration (mol%), and the resulting data were fitted to the Hill equation using OriginPro 2023.

$$y = \frac{V_{max} \cdot x^n}{k^n + x^n}$$

where *y* is the *normalized F_0/F* value 300 s after the addition of transporter and *x* is the transporter concentration (mol% with respect to lipid). V_{max}, *k* and *n* are the parameters to be fitted. V_{max} is the maximum value for *y* and is fixed to 0.9 (the maximum value we normally obtain in these experiments), *n* is the Hill coefficient, and *k* is the EC_{50} or inflection point of the graph. To further investigate the binding behavior of the compounds, the initial rates obtained from chloride transport traces at different transporter concentrations were plotted against transporter concentration (mol%), the square of the concentration, or the square root of the concentration (for compound **2c**). A linear relationship between the initial rate and the square root of the transporter concentration suggests a 1:2 binding stoichiometry, whereas a linear relationship between the

initial rate and transporter concentration indicates a 1:1 binding stoichiometry. The results are shown in **Figure S55-Figure S58.**

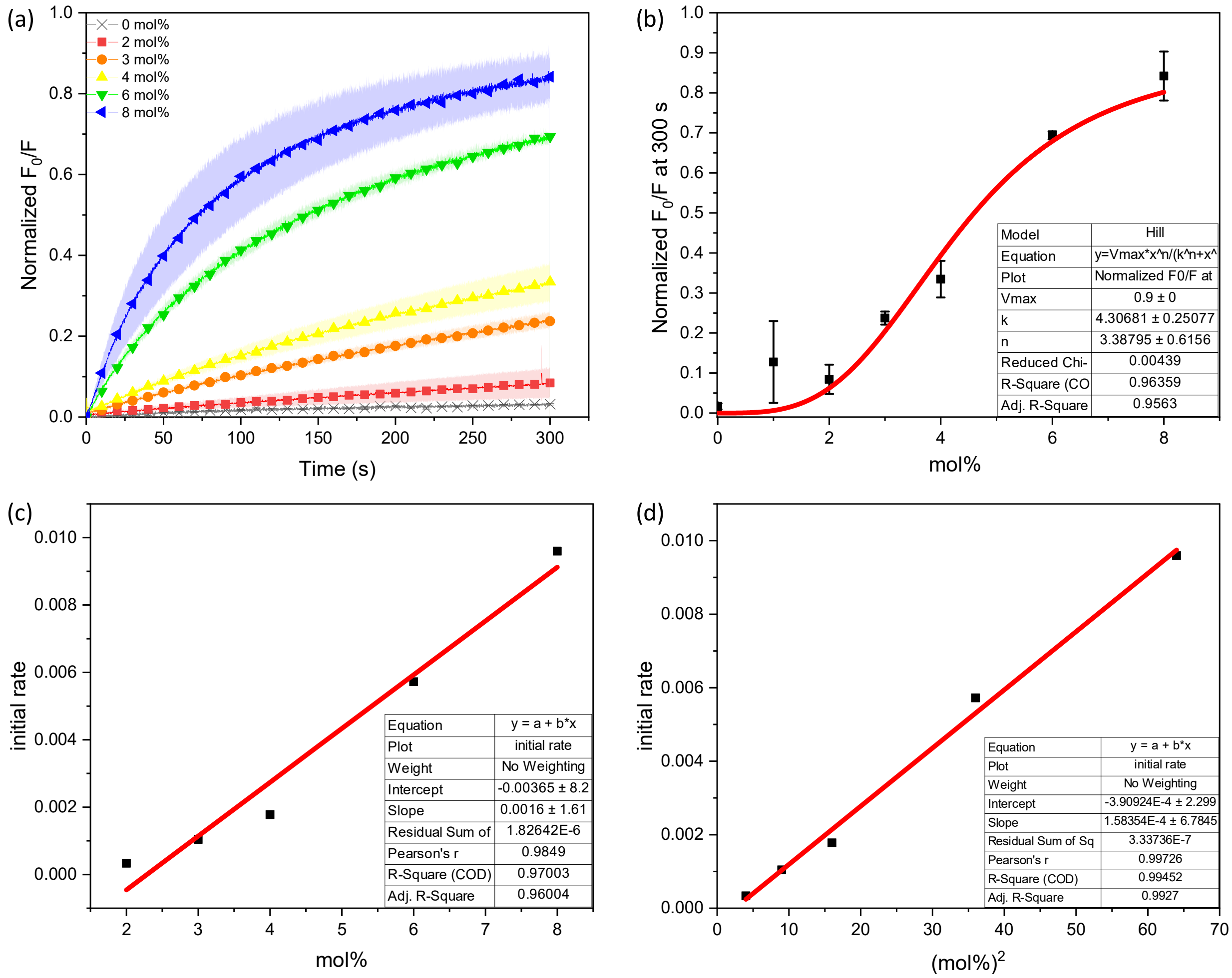

Figure S55. (a) Chloride influx (*Normalized F_0/F*) mediated by transporter **1f** (various mol% transporter with respect to lipid) from 100 nm unilamellar POPC vesicles loaded with 1 mM lucigenin, 225 mM $NaNO_3$ and 5 mM HEPES at pH 7.4. The experiments were performed as described in *Section S6.1.* and is the average of minimum of 3 repeats whereby the shaded areas represent standard deviations. (b) Hill plots from the chloride influx values at 300 s mediated by transporter **1f**. (c) Plot of transporter concentration (mol%) versus initial rate of chloride transport fitted with a linear function. (d) Plot of the square of transporter concentration ((mol%)^2) versus initial rate of chloride transport fitted with a linear function.

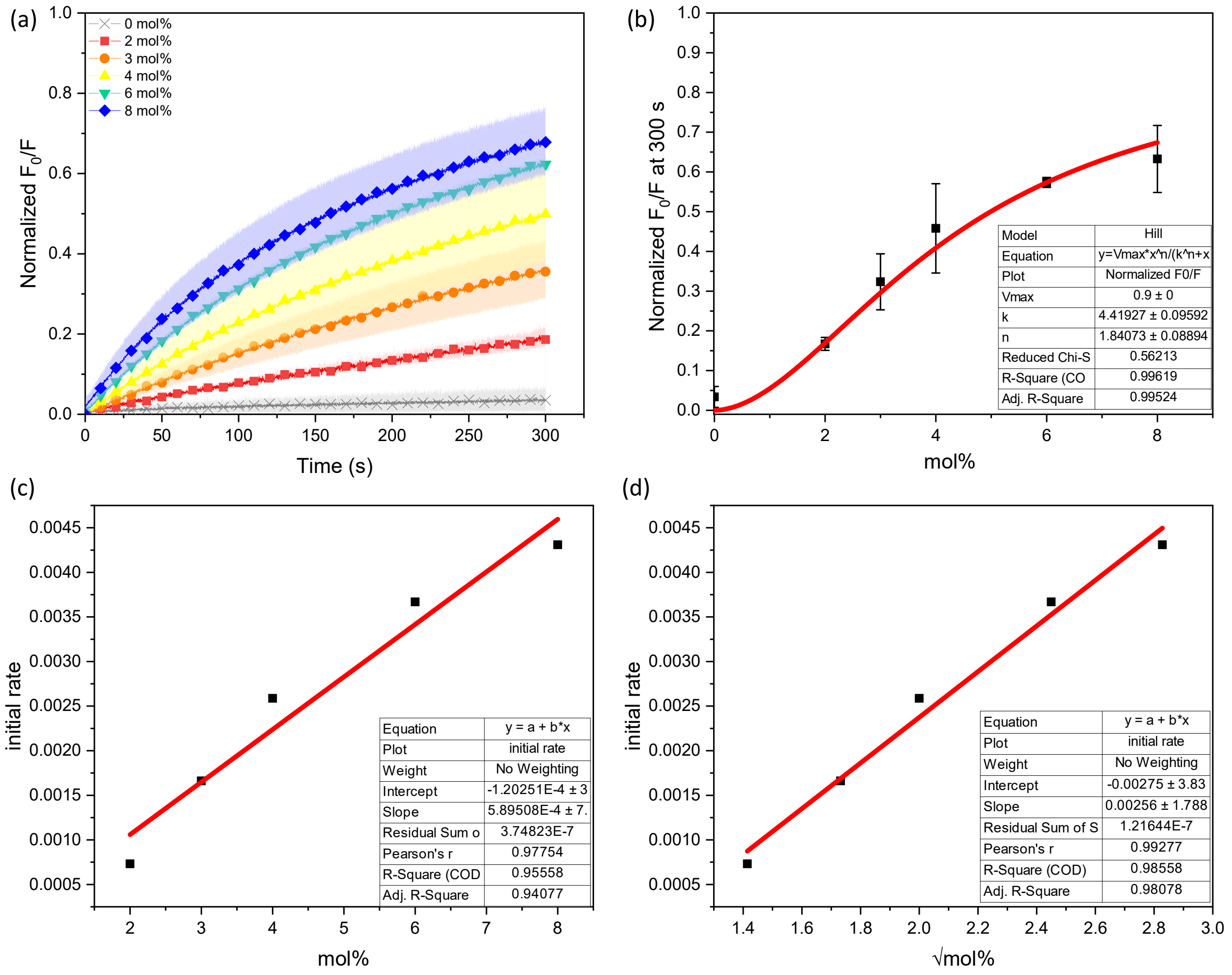

Figure S56. (a) Chloride influx (*Normalized F_0/F*) mediated by transporter **2c** (various mol% transporter with respect to lipid) from 100 nm unilamellar POPC vesicles loaded with 1 mM lucigenin, 225 mM $NaNO_3$ and 5 mM HEPES at pH 7.4. The experiments were performed as described in *Section S6.1.* and is the average of minimum of 3 repeats whereby the shaded areas represent standard deviations. (b) Hill plots from the chloride influx values at 300 s mediated by transporter **2c**. (c) Plot of transporter concentration (mol%) versus initial rate of chloride transport fitted with a linear function. (d) Plot of the square root of transporter concentration (√mol%) versus initial rate of chloride transport fitted with a linear function.

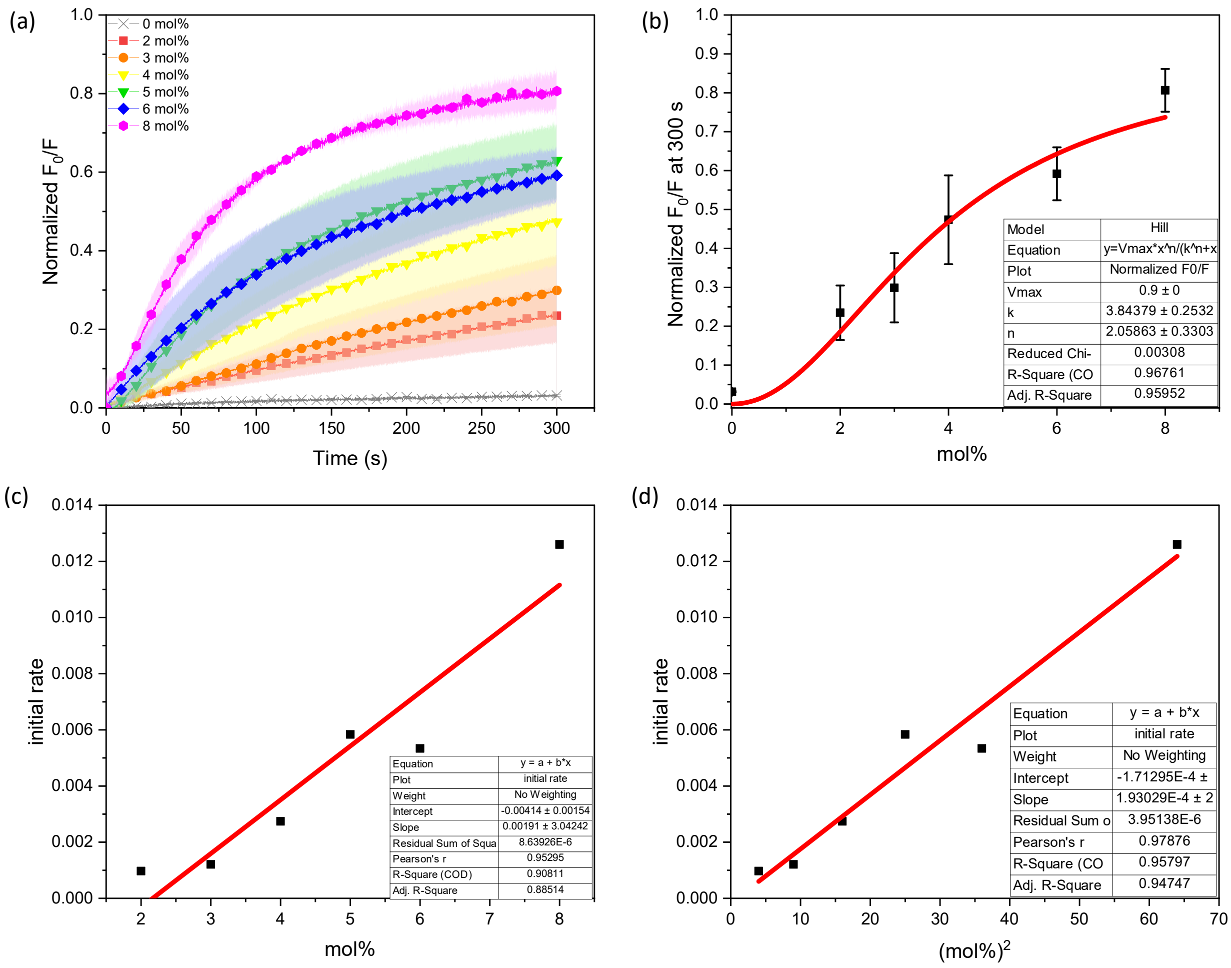

Figure S57. (a) Chloride influx (*Normalized F_0/F*) mediated by transporter **2e** (various mol% transporter with respect to lipid) from 100 nm unilamellar POPC vesicles loaded with 1 mM lucigenin, 225 mM $NaNO_3$ and 5 mM HEPES at pH 7.4. The experiments were performed as described in *Section S6.1.* and is the average of minimum of 3 repeats whereby the shaded areas represent standard deviations. (b) Hill plots from the chloride influx values at 300 s mediated by transporter **2e**. (c) Plot of transporter concentration (mol%) versus initial rate of chloride transport fitted with a linear function. (d) Plot of the square root of transporter concentration (√mol%) versus initial rate of chloride transport fitted with a linear function.

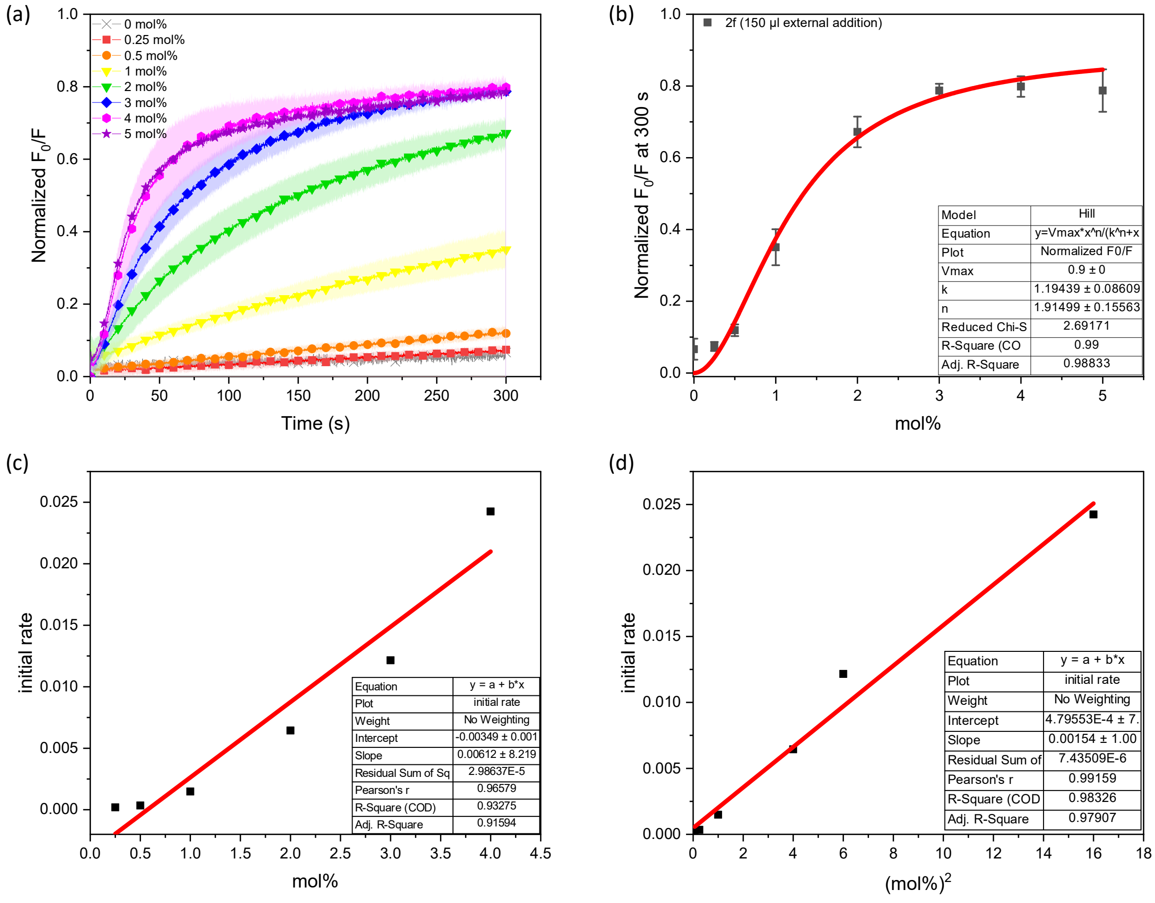

Figure S58. (a) Chloride influx (*Normalized F_0/F*) mediated by transporter **2f** (various mol% transporter with respect to lipid) into 100 nm unilamellar POPC vesicles loaded with 1 mM lucigenin, 225 mM $NaNO_3$ and 5 mM HEPES at pH 7.4. The experiments were performed as described in *Section S6.1.* using external addition of 150 µL MeOH stocks and is the average of minimum of 3 repeats whereby the shaded areas represent standard deviations (c) Hill plots from the chloride influx values at 300 s mediated by transporter **2f** using external addition (150 µL MeOH stocks). (d) Plot of transporter concentration (mol%) versus initial rate of chloride transport fitted with a linear function. (e) Plot of the square root of transporter concentration (mol%^{2}) versus initial rate of chloride transport fitted with a linear function.

S6.4 Transport Mechanism

S6.4.1 Anion/Cation Selectivity

Selectivity experiments followed the same liposome preparation procedure described in *Section S6.1*, using pre-incorporated compounds to accurately assess transport efficiency. The only difference is that the 25 mM NaCl pulse is replaced with either KCl, RbCl or CsCl (to determine possible cation transport) or NaBr or NaI (to determine anion transport). Compounds **1a-1f** and **2c-2f** were pre-incorporated at 5 mol% transporter-to-lipid, while compounds **2a** and **2b** were tested at 0.5

mol%. The results are presented in **Figure S59-Figure S70**. In general, there is no effect on the nature of the cation —suggesting that M^+/Cl^- symport is unlikely, but a strong influence on the nature of the anion is observed —confirming anion over cation selectivity for transmembrane transport.

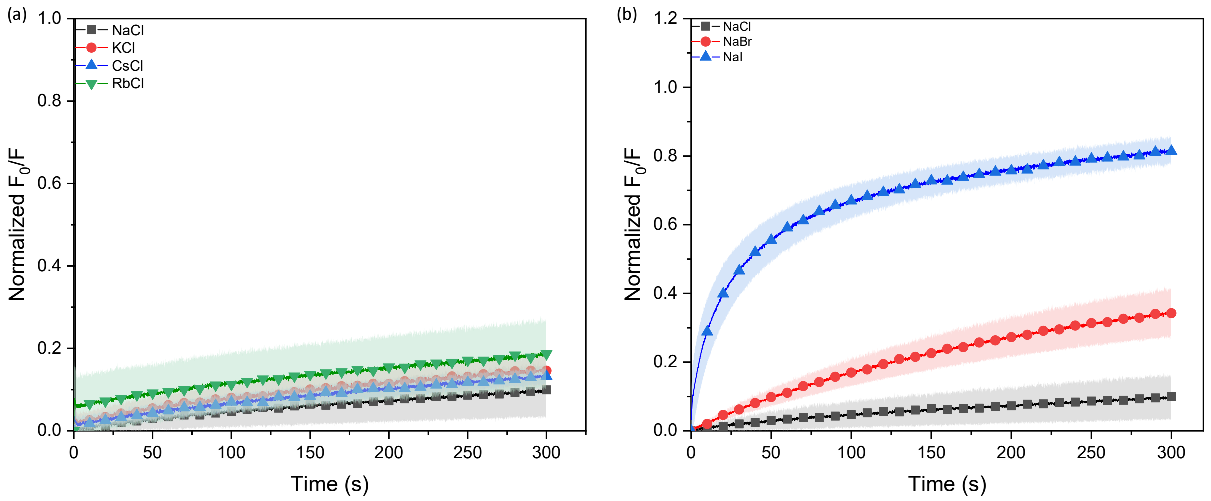

Figure S59. Anion transport (*Normalized F_0/F*) mediated by transporter **1a** (5 mol%) into 100 nm unilamellar POPC vesicles loaded with 1 mM lucigenin, 225 mM $NaNO_3$ and 5 mM HEPES buffer at pH 7.4 and suspended into a solution of 225 mM $NaNO_3$ and 5 mM HEPES buffer at pH 7.4. The experiments were performed as described in *Section S6.1.* using pre-incorporation to test either (a) cation transport (pulses of NaCl, KCl, RbCl or CsCl), or (b) anion transport (pulses of NaCl, NaBr or NaI).

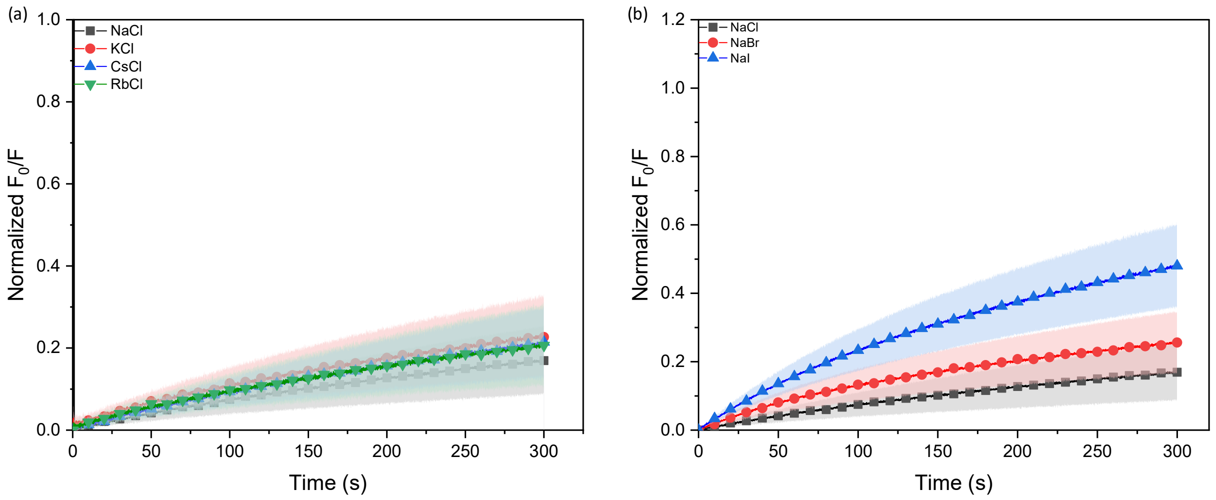

Figure S60. Anion transport (*Normalized F_0/F*) mediated by transporter **1b** (5 mol%) into 100 nm unilamellar POPC vesicles loaded with 1 mM lucigenin, 225 mM $NaNO_3$ and 5 mM HEPES buffer at pH 7.4 and suspended into a solution of 225 mM $NaNO_3$ and 5 mM HEPES buffer at pH 7.4. The experiments were performed as described in *Section S6.1.* using pre-incorporation to test either (a) cation transport (pulses of NaCl, KCl, RbCl or CsCl), or (b) anion transport (pulses of NaCl, NaBr or NaI).

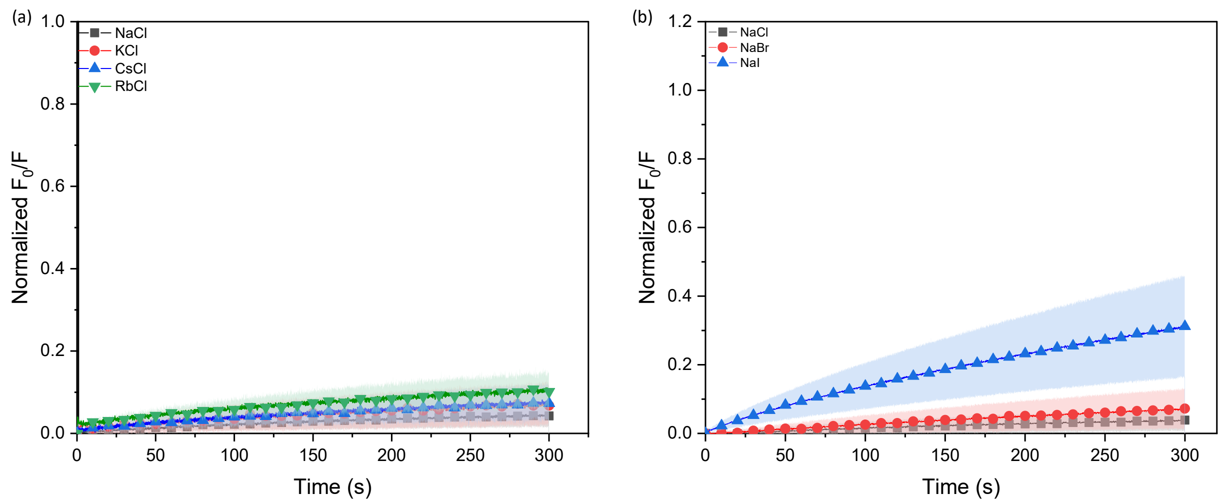

Figure S61. Anion transport (*Normalized F_0/F*) mediated by transporter **1c** (5 mol%) into 100 nm unilamellar POPC vesicles loaded with 1 mM lucigenin, 225 mM $NaNO_3$ and 5 mM HEPES buffer at pH 7.4 and suspended into a solution of 225 mM $NaNO_3$ and 5 mM HEPES buffer at pH 7.4. The experiments were performed as described in *Section S6.1.* using pre-incorporation to test either (a) cation transport (pulses of NaCl, KCl, RbCl or CsCl), or (b) anion transport (pulses of NaCl, NaBr or NaI).

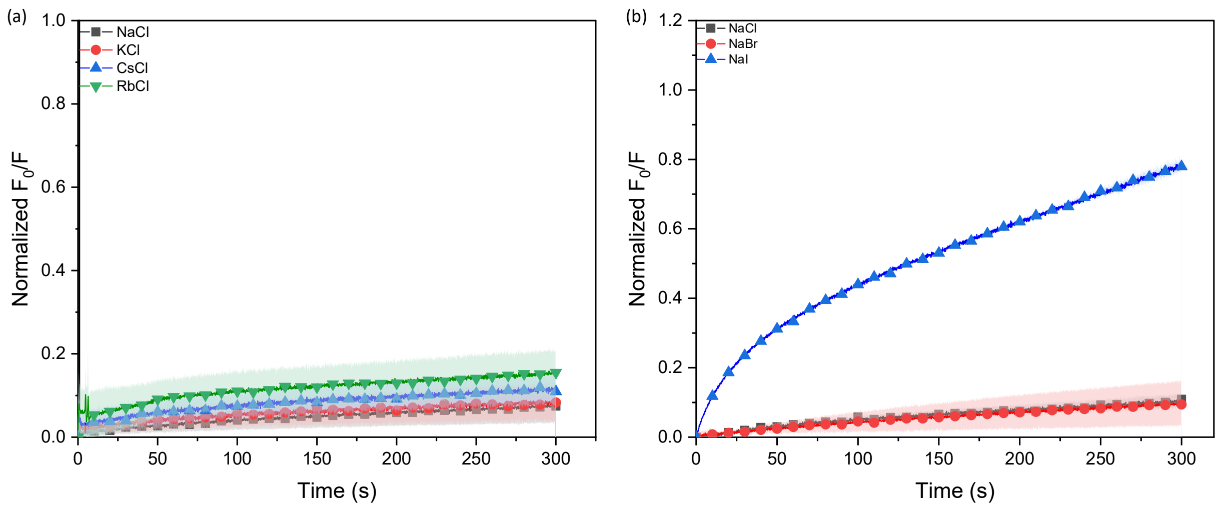

Figure S62. Anion transport (*Normalized F_0/F*) mediated by transporter **1d** (5 mol%) into 100 nm unilamellar POPC vesicles loaded with 1 mM lucigenin, 225 mM $NaNO_3$ and 5 mM HEPES buffer at pH 7.4 and suspended into a solution of 225 mM $NaNO_3$ and 5 mM HEPES buffer at pH 7.4. The experiments were performed as described in *Section S6.1.* using pre-incorporation to test either (a) cation transport (pulses of NaCl, KCl, RbCl or CsCl), or (b) anion transport (pulses of NaCl, NaBr or NaI).

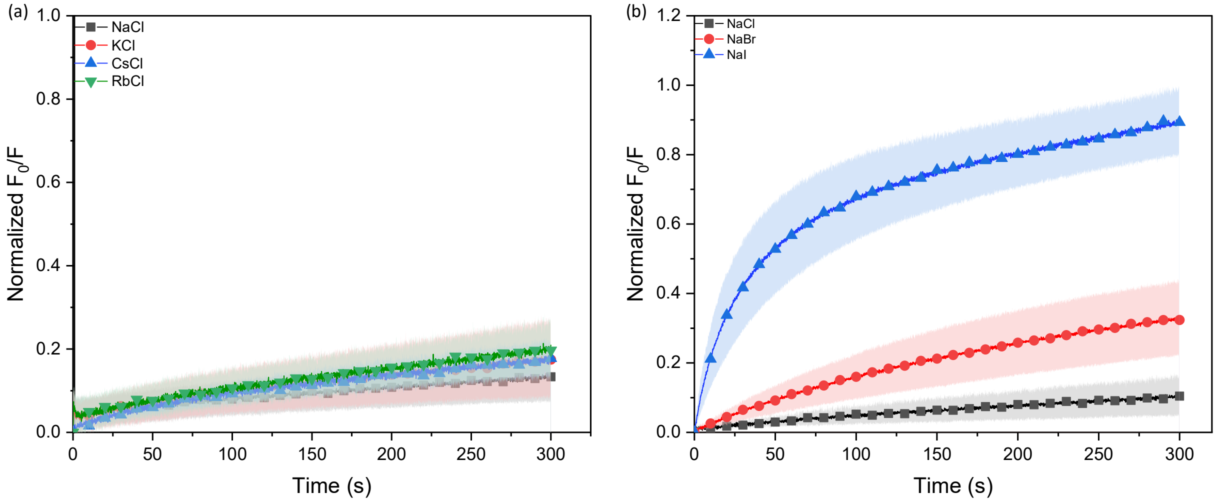

Figure S63. Anion transport (*Normalized F_0/F*) mediated by transporter **1e** (5 mol%) into 100 nm unilamellar POPC vesicles loaded with 1 mM lucigenin, 225 mM $NaNO_3$ and 5 mM HEPES buffer at pH 7.4 and suspended into a solution of 225 mM $NaNO_3$ and 5 mM HEPES buffer at pH 7.4. The experiments were performed as described in *Section S6.1.* using pre-incorporation to test either (a) cation transport (pulses of NaCl, KCl, RbCl or CsCl), or (b) anion transport (pulses of NaCl, NaBr or NaI).

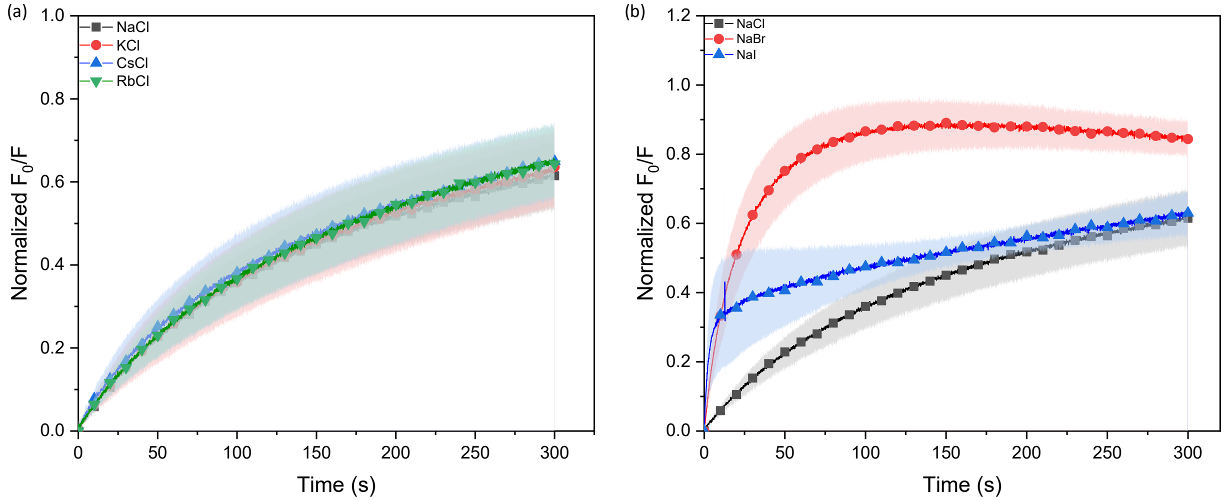

Figure S64. Anion transport (*Normalized F_0/F*) mediated by transporter **1f** (5 mol%) into 100 nm unilamellar POPC vesicles loaded with 1 mM lucigenin, 225 mM $NaNO_3$ and 5 mM HEPES buffer at pH 7.4 and suspended into a solution of 225 mM $NaNO_3$ and 5 mM HEPES buffer at pH 7.4. The experiments were performed as described in *Section S6.1.* using pre-incorporation to test either (a) cation transport (pulses of NaCl, KCl, RbCl or CsCl), or (b) anion transport (pulses of NaCl, NaBr or NaI).

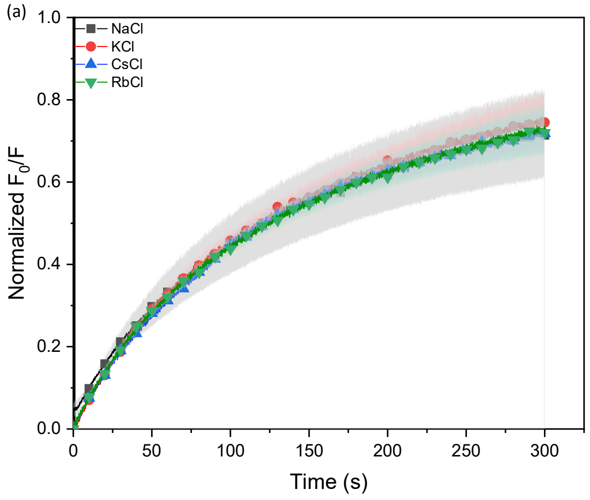

Figure S65. Anion transport (*Normalized F_0/F*) mediated by transporter **2a** (0.5 mol%) into 100 nm unilamellar POPC vesicles loaded with 1 mM lucigenin, 225 mM $NaNO_3$ and 5 mM HEPES buffer at pH 7.4 and suspended into a solution of 225 mM $NaNO_3$ and 5 mM HEPES buffer at pH 7.4. The experiments were performed as described in *Section S6.1.* using pre-incorporation to test either (a) cation transport (pulses of NaCl, KCl, RbCl or CsCl), or (b) anion transport (pulses of NaCl, NaBr or NaI).

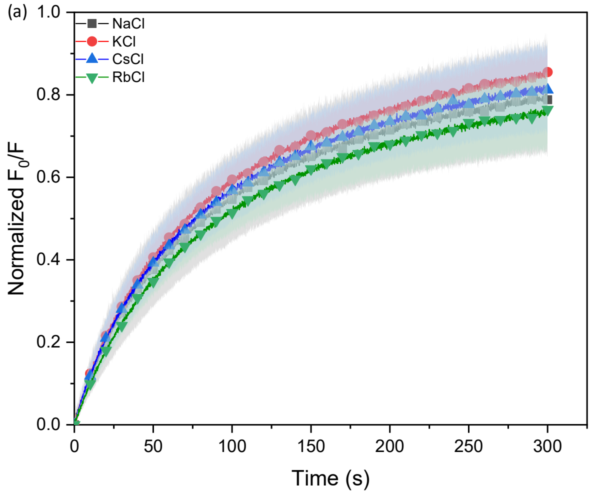

Figure S66. Anion transport (*Normalized F_0/F*) mediated by transporter **2b** (0.5 mol%) into 100 nm unilamellar POPC vesicles loaded with 1 mM lucigenin, 225 mM $NaNO_3$ and 5 mM HEPES buffer at pH 7.4 and suspended into a solution of 225 mM $NaNO_3$ and 5 mM HEPES buffer at pH 7.4. The experiments were performed as described in *Section S6.1.* using pre-incorporation to test either (a) cation transport (pulses of NaCl, KCl, RbCl or CsCl), or (b) anion transport (pulses of NaCl, NaBr or NaI).

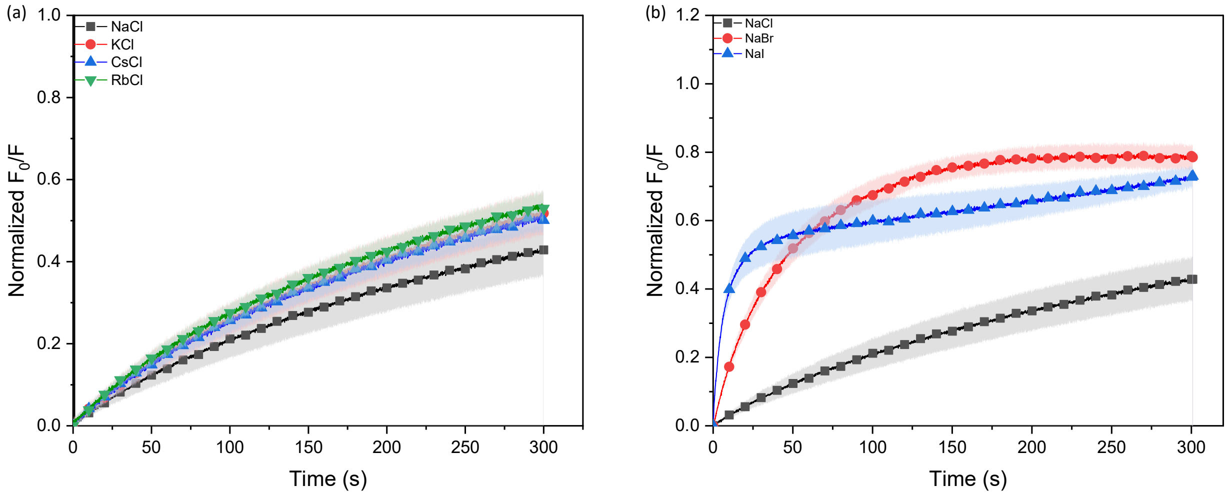

Figure S67. Anion transport (*Normalized F_0/F*) mediated by transporter **2c** (5 mol%) into 100 nm unilamellar POPC vesicles loaded with 1 mM lucigenin, 225 mM $NaNO_3$ and 5 mM HEPES buffer at pH 7.4 and suspended into a solution of 225 mM $NaNO_3$ and 5 mM HEPES buffer at pH 7.4. The experiments were performed as described in *Section S6.1.* using pre-incorporation to test either (a) cation transport (pulses of NaCl, KCl, RbCl or CsCl), or (b) anion transport (pulses of NaCl, NaBr or NaI).

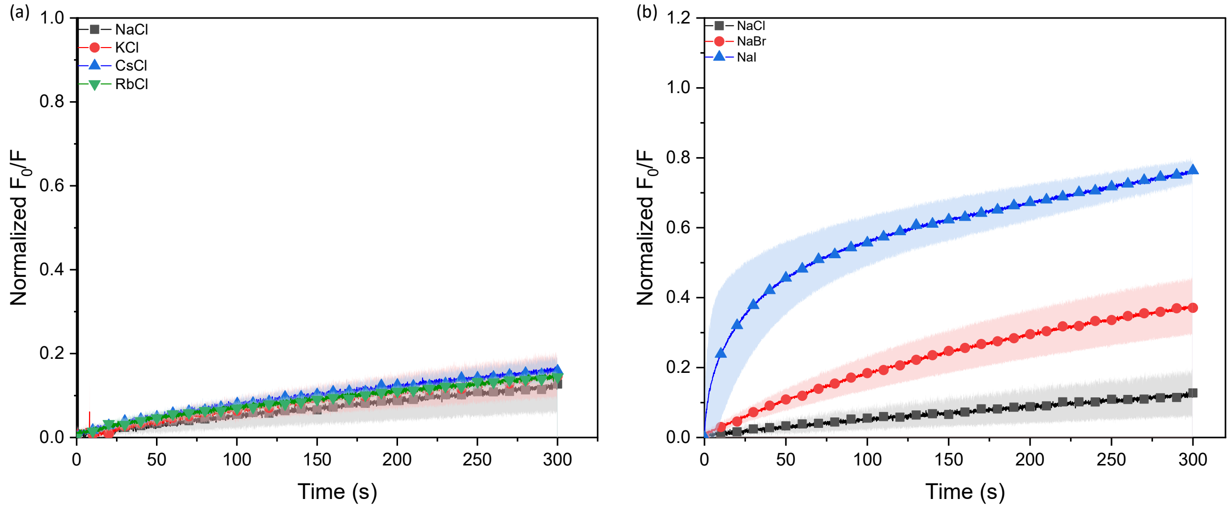

Figure S68. Anion transport (*Normalized F_0/F*) mediated by transporter **2d** (5 mol%) into 100 nm unilamellar POPC vesicles loaded with 1 mM lucigenin, 225 mM $NaNO_3$ and 5 mM HEPES buffer at pH 7.4 and suspended into a solution of 225 mM $NaNO_3$ and 5 mM HEPES buffer at pH 7.4. The experiments were performed as described in *Section S6.1.* using pre-incorporation to test either (a) cation transport (pulses of NaCl, KCl, RbCl or CsCl), or (b) anion transport (pulses of NaCl, NaBr or NaI).

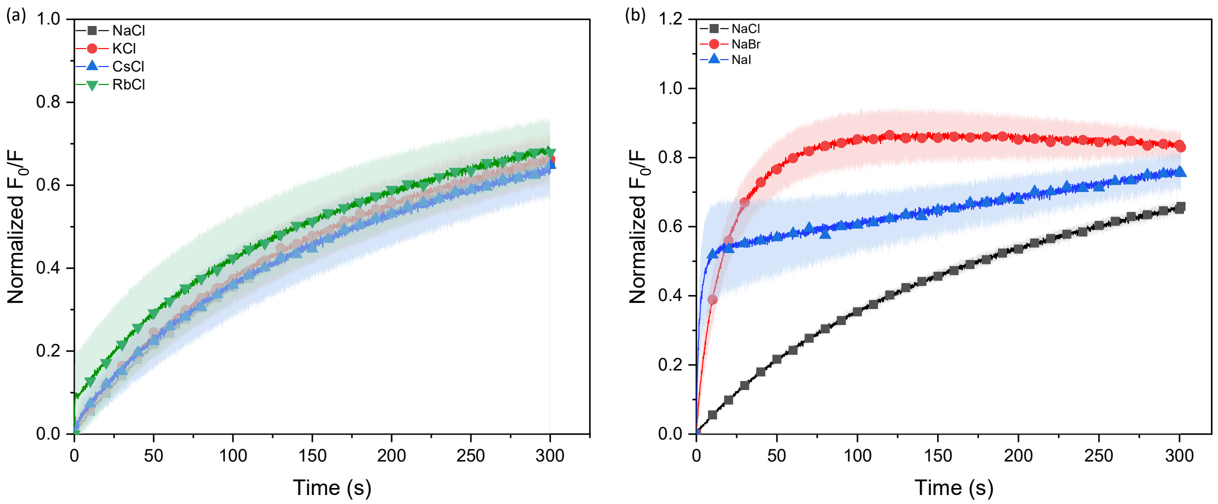

Figure S69. Anion transport (*Normalized F_0/F*) mediated by transporter **2e** (5 mol%) into 100 nm unilamellar POPC vesicles loaded with 1 mM lucigenin, 225 mM $NaNO_3$ and 5 mM HEPES buffer at pH 7.4 and suspended into a solution of 225 mM $NaNO_3$ and 5 mM HEPES buffer at pH 7.4. The experiments were performed as described in *Section S6.1.* using pre-incorporation to test either (a) cation transport (pulses of NaCl, KCl, RbCl or CsCl), or (b) anion transport (pulses of NaCl, NaBr or NaI).

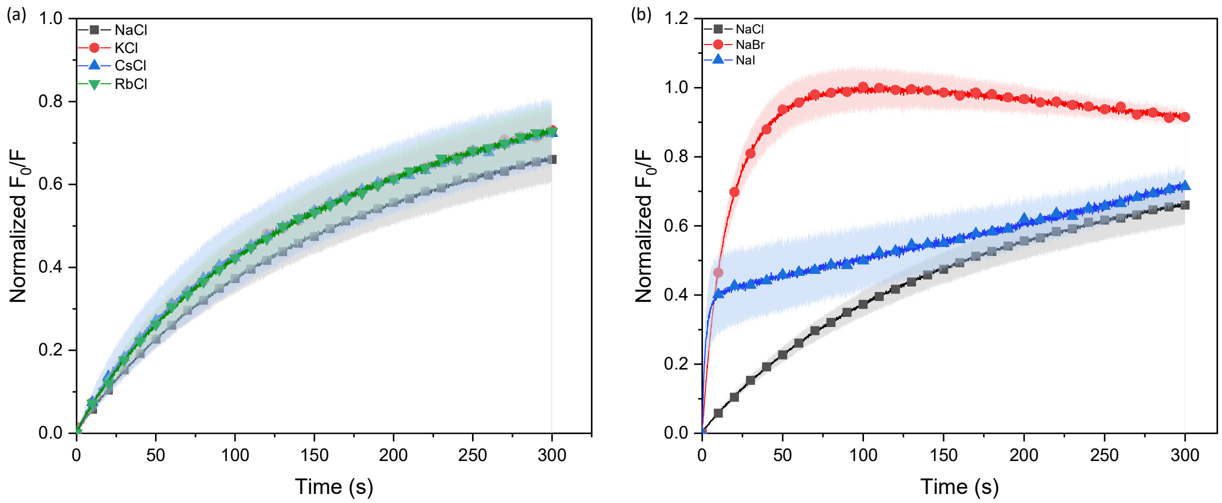

Figure S70. Anion transport (*Normalized F_0/F*) mediated by transporter **2f** (5 mol%) into 100 nm unilamellar POPC vesicles loaded with 1 mM lucigenin, 225 mM $NaNO_3$ and 5 mM HEPES buffer at pH 7.4 and suspended into a solution of 225 mM $NaNO_3$ and 5 mM HEPES buffer at pH 7.4. The experiments were performed as described in *Section S6.1.* using pre-incorporation to test either (a) cation transport (pulses of NaCl, KCl, RbCl or CsCl), or (b) anion transport (pulses of NaCl, NaBr or NaI).

S6.4.2 Carrier vs Ion Channel

To determine whether the transporters function as channels or a mobile carriers, the standard lucigenin assay was carried out using 1,2-dipalmitoyl-sn-glycero-3-phosphocholine (DPPC). The principle of this experiment is based on the gel-to-liquid crystalline phase transition of DPPC, which occurs at approximately 41°C. Below this temperature (e.g., at 25°C), the lipid bilayer remains in a rigid gel phase, restricting the mobility of molecules. A transporter that still facilitates chloride efflux in this phase is likely to operate via a channel-like mechanism, allowing ions to diffuse through a stable pore.[8] In contrast, a transporter that only exhibits activity above the phase transition temperature (e.g., at 45°C) indicates mobile-carrier behavior, requiring lipid fluidity for molecular diffusion across the bilayer.

Experimental procedures generally followed the standard lucigenin assay described in *Section S6.1* for external addition, with modifications to accommodate the use of DPPC and temperature-dependent measurements: hydration of the lipid film was performed at 50 °C, freeze-thaw cycles were performed so that thawing reached 55 °C, and extrusion was performed at 55 °C using a pre-heated block for the extrusion kit covered with aluminum foil. Samples placed in the fluorometer were allowed to equilibrate for 10 minutes to ensure the liposome reached the desired temperature (25°C or 45°C) maintained by the Peltier temperature controller of the instrument. Data collection and work-up were performed as described in *Section S6.1*. Experiments were performed in triplicate using independently prepared liposome batches and the results are shown in **Figure S71-Figure S82.**

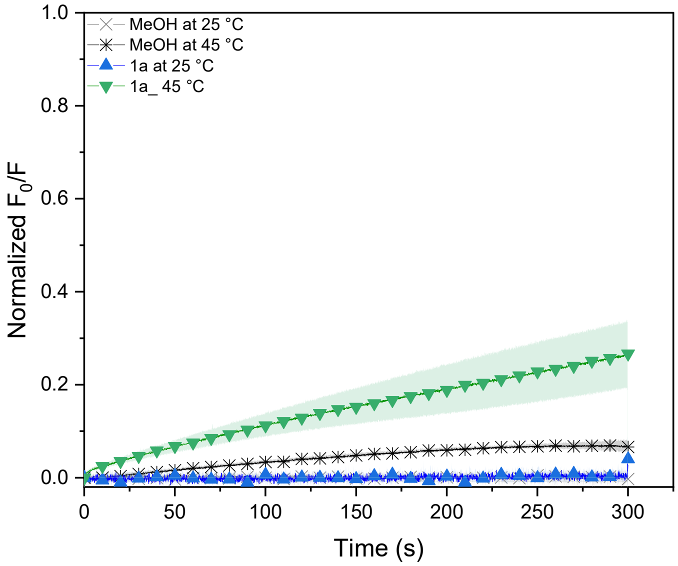

Figure S71. Chloride transport activity of compound **1a** (5 mol% relative to total lipid) in DPPC vesicles at 25°C and 45°C measured using the lucigenin assay, with corresponding MeOH blanks. Each trace represents the average of three independent experiments and shaded areas represent standard deviation.

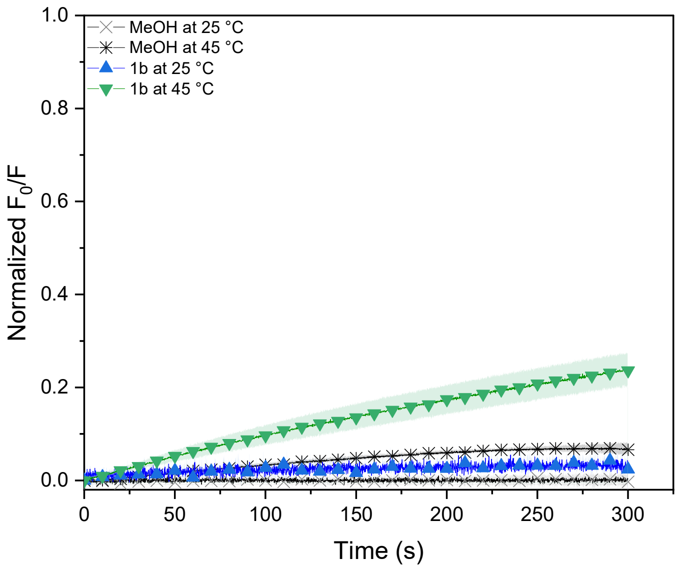

Figure S72. Chloride transport activity of compound **1b** (5 mol% relative to total lipid) in DPPC vesicles at 25°C and 45°C measured using the lucigenin assay, with corresponding MeOH blanks. Each trace represents the average of three independent experiments and shaded areas represent standard deviation.

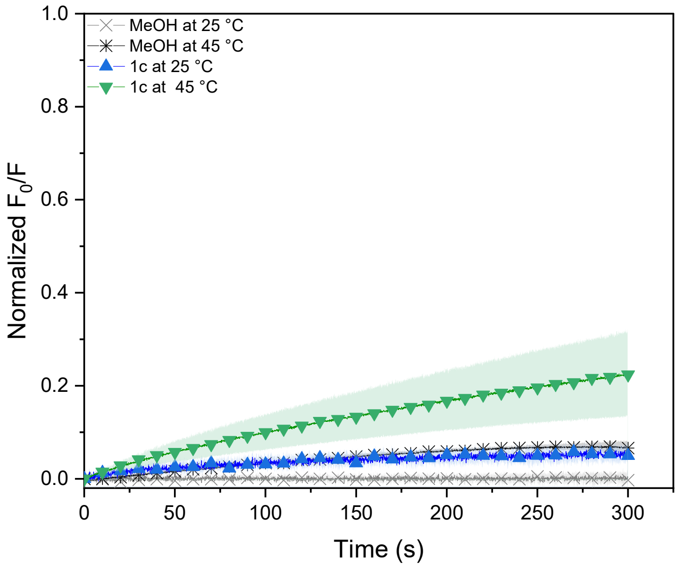

Figure S73. Chloride transport activity of compound **1c** (5 mol% relative to total lipid) in DPPC vesicles at 25°C and 45°C measured using the lucigenin assay, with corresponding MeOH blanks. Each trace represents the average of three independent experiments and shaded areas represent standard deviation.

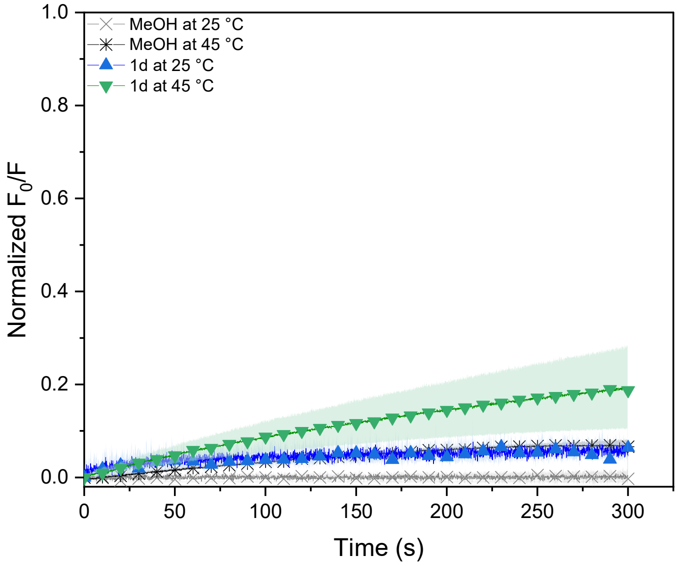

Figure S74. Chloride transport activity of compound **1d** (5 mol% relative to total lipid) in DPPC vesicles at 25°C and 45°C measured using the lucigenin assay, with corresponding MeOH blanks. Each trace represents the average of three independent experiments and shaded areas represent standard deviation.

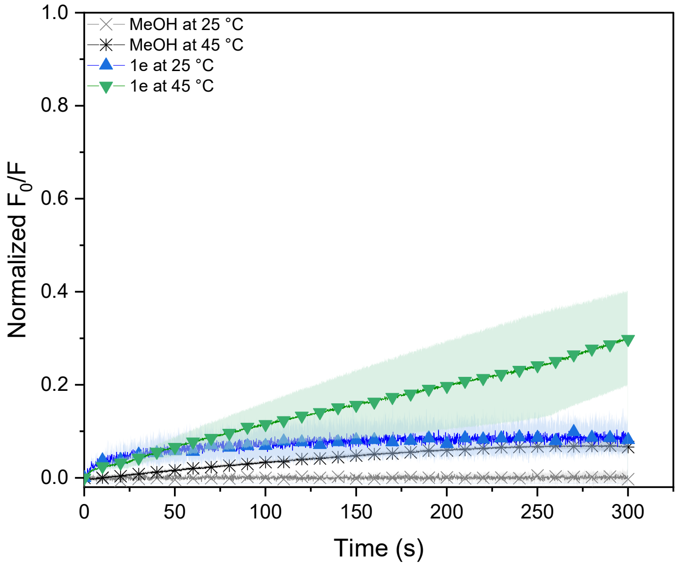

Figure S75. Chloride transport activity of compound **1e** (5 mol% relative to total lipid) in DPPC vesicles at 25°C and 45°C measured using the lucigenin assay, with corresponding MeOH blanks. Each trace represents the average of three independent experiments and shaded areas represent standard deviation.

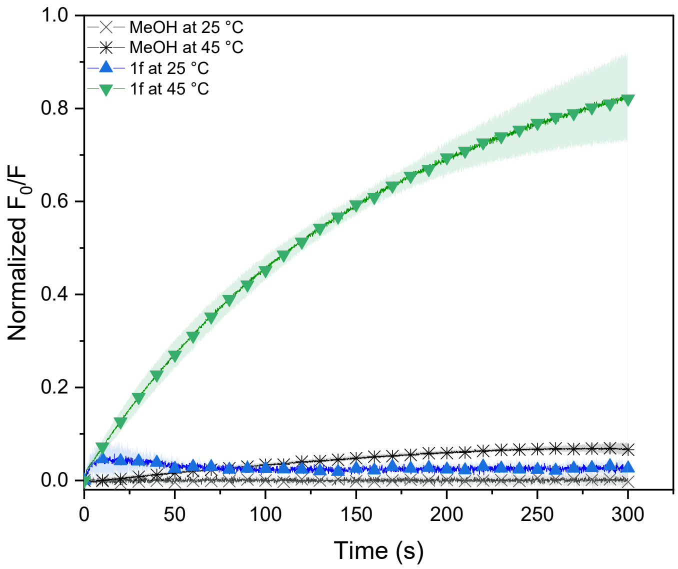

Figure S76. Chloride transport activity of compound **1f** (5 mol% relative to total lipid) in DPPC vesicles at 25°C and 45°C measured using the lucigenin assay, with corresponding MeOH blanks. Each trace represents the average of three independent experiments and shaded areas represent standard deviation.

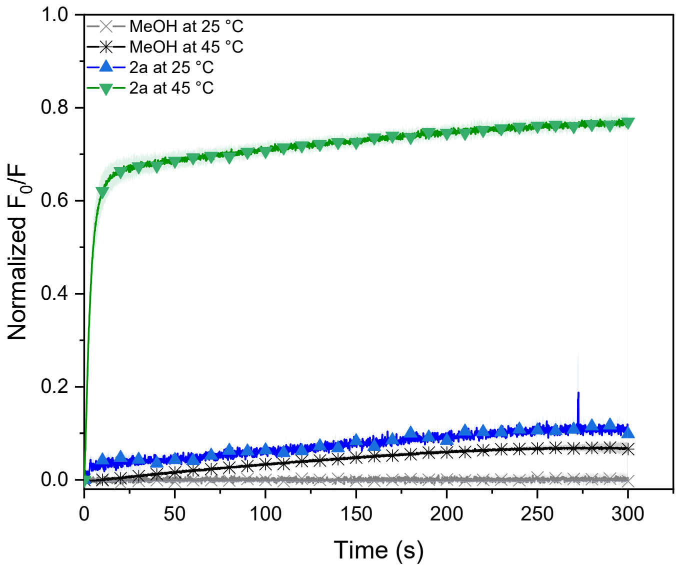

Figure S77. Chloride transport activity of compound **2a** (0.5 mol% relative to total lipid) in DPPC vesicles at 25°C and 45°C measured using the lucigenin assay, with corresponding MeOH blanks. Each trace represents the average of three independent experiments and shaded areas represent standard deviation.

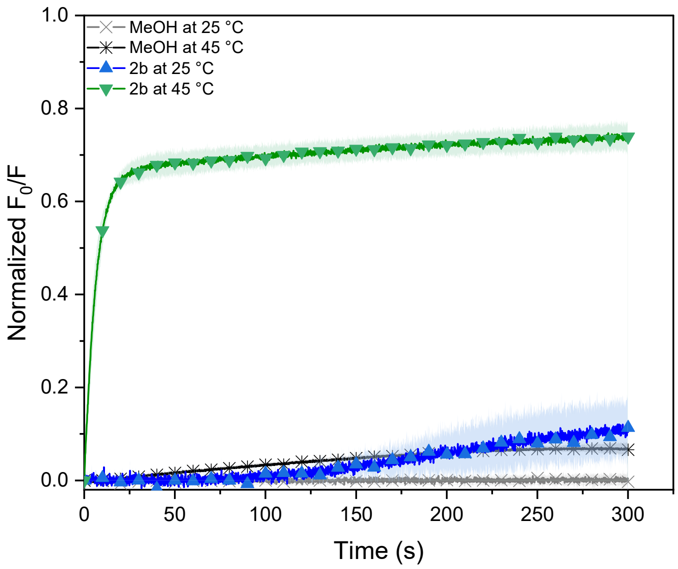

Figure S78. Chloride transport activity of compound **2b** (0.5 mol% relative to total lipid) in DPPC vesicles at 25°C and 45°C measured using the lucigenin assay, with corresponding MeOH blanks. Each trace represents the average of three independent experiments and shaded areas represent standard deviation.

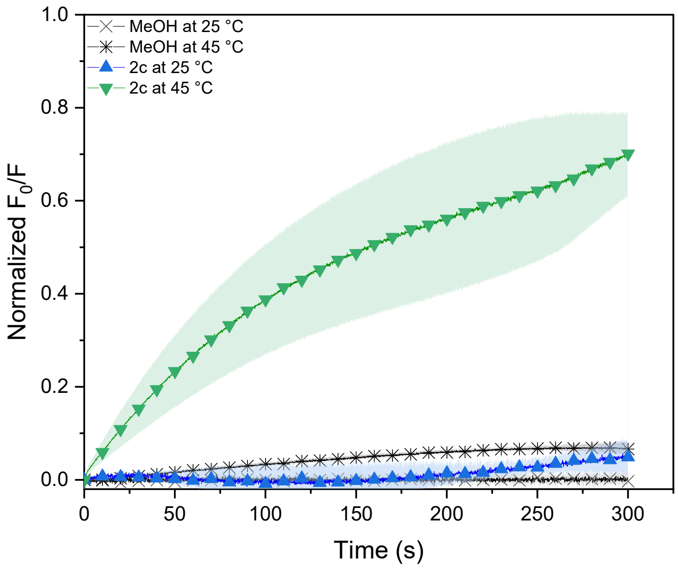

Figure S79. Chloride transport activity of compound **2c** (5 mol% relative to total lipid) in DPPC vesicles at 25°C and 45°C measured using the lucigenin assay, with corresponding MeOH blanks. Each trace represents the average of three independent experiments and shaded areas represent standard deviation.

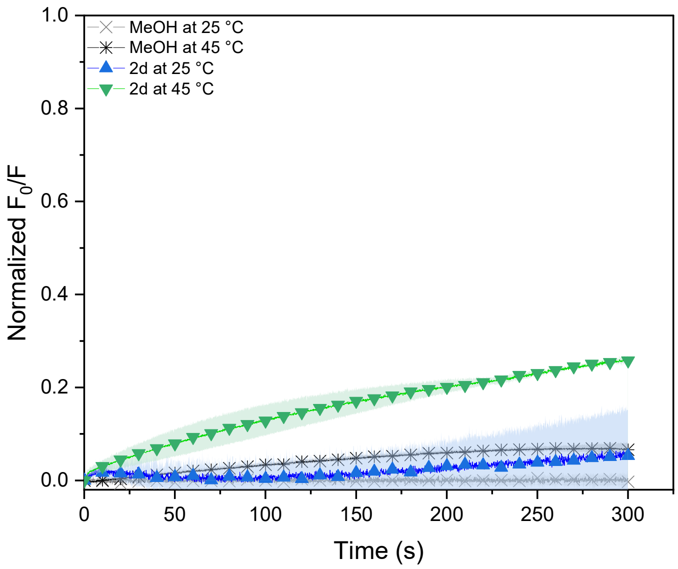

Figure S80. Chloride transport activity of compound **2d** (5 mol% relative to total lipid) in DPPC vesicles at 25°C and 45°C measured using the lucigenin assay, with corresponding MeOH blanks. Each trace represents the average of three independent experiments and shaded areas represent standard deviation.

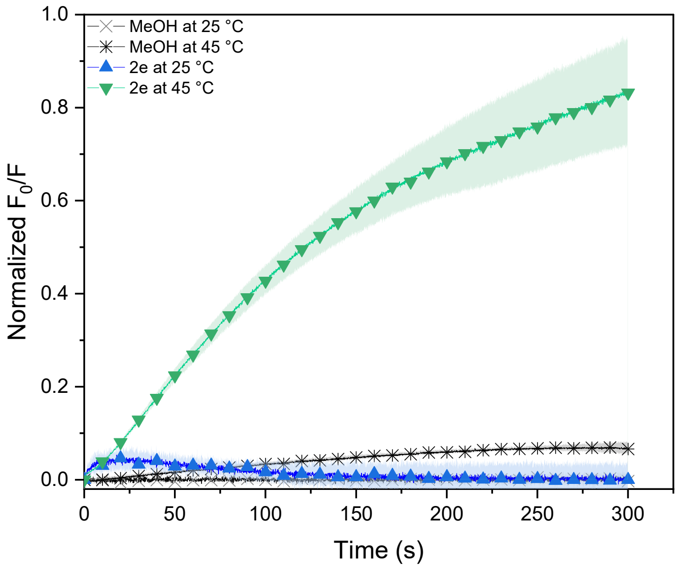

Figure S81. Chloride transport activity of compound **2e** (5 mol% relative to total lipid) in DPPC vesicles at 25°C and 45°C measured using the lucigenin assay, with corresponding MeOH blanks. Each trace represents the average of three independent experiments and shaded areas represent standard deviation.

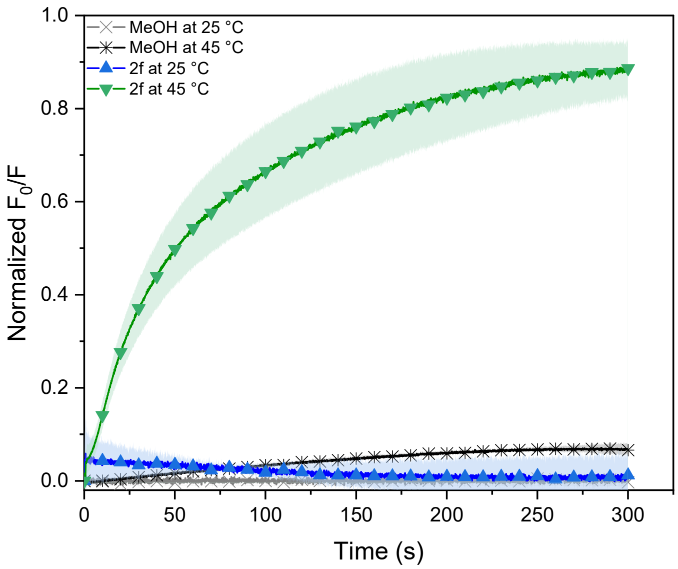

Figure S82. Chloride transport activity of compound **2f** (5 mol% relative to total lipid; compound added as a MeOH stock solution to achieve a final MeOH concentration of 5% v/v to ensure deliverability) in DPPC vesicles at 25 °C and 45 °C measured using the lucigenin assay, with corresponding MeOH blanks. Each trace represents the average of three independent experiments, and shaded areas represent the standard deviation.

S6.4.3 HPTS Assay

The 8-hydroxypyrene-1,3,6-trisulfonic acid (HPTS) assay was performed according to previously reported methods.[10] A lipid film of POPC was formed from a chloroform solution under reduced pressure and subsequently dried under vacuum for at least 4 hours. The dry film was rehydrated with 1 mL buffer (1 mM HPTS, 100 mM NMDG-Cl (*N*-methyl-D-glucamine, HCl salt), 10 mM HEPES, pH 7.0), followed by vortexing. The hydrated lipid suspension was subjected to nine freeze–thaw cycles (alternating between liquid nitrogen and warm water) and equilibrated at room temperature for 30 min. The vesicles were then extruded 25 times through a 200 nm polycarbonate membrane (Nucleopore™) using an Avanti mini-extruder. Unencapsulated HPTS was removed by size exclusion chromatography using a Sephadex G-25 column and an external buffer of 100 mM NMDG-Cl, 10 mM HEPES, pH 7.0 (without HPTS). The lipid-containing fractions were collected and diluted to give a final lipid concentration of 0.1 mM. Bovine serum albumin (BSA) was then added to the vesicle suspension at 1 mol% relative to lipid and gently stirred for 30 minutes. 3 mL of the HPTS-loaded liposomes (0.1 mM total lipid concentration) were transferred into a glass fluorescence cuvette containing a small magnetic stir bar. The cuvette was placed inside the fluorometer, and stirring was initiated at maximum speed, continuing throughout the experiment. Data

acquisition began with excitation wavelengths set to 405 nm (protonated form) and 460 nm (deprotonated form) and the emission wavelength set to 510 nm in each case. The fluorescence signal was collected as the ratio (*R*) of the emission intensity measured at 510 nm after excitation at 405 nm and 460 nm, $R = I_{460}/I_{405}$. At t = 10 s, a base pulse (15 µL of 1 M NMDG) was added to establish a pH gradient (pH ≈ 7 inside, pH ≈ 8 outside). At t = 20 s, Gramicidin D was introduced as a protonophore to achieve a concentration of 0.1 mol% relative to lipid (this step was only performed for coupling with Gramicidin D experiments). At t = 40 s, the test compound (5 mol% relative to lipid) was added from a MeOH stock solution (7.5 µL). For compound **2f**, a larger injection volume (150 µL) was used to maintain the same final concentration due to the deliverability issues discussed above. At t = 350 s, 50 µL of 10% Triton X-100 was introduced to fully disrupt the membrane and obtain the maximal fluorescence response. To account for baseline differences and to compare data across replicates, the fluorescence ratio was normalized by defining the ratio at the time of compound addition (40 s) as R_0, and the final detergent-lysed ratio as R_d. The fractional fluorescence response (I_f) was calculated by the following equation:

$$I_f = \frac{R_t - R_0}{R_d - R_0}$$

The results are shown in **Figure S83-Figure S94.**

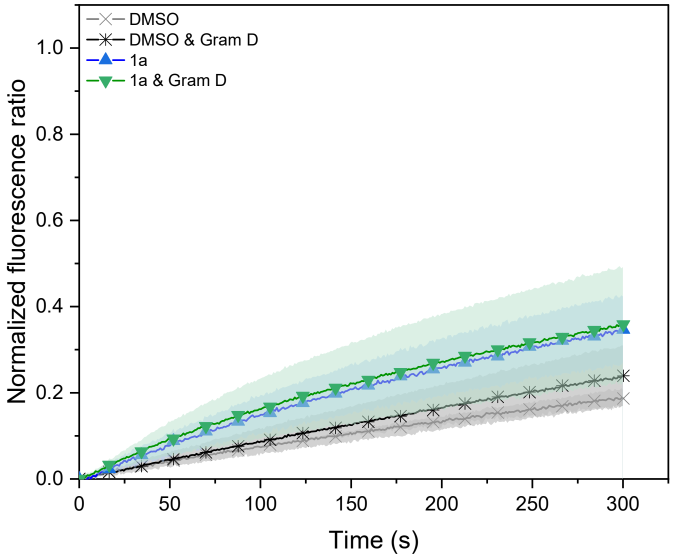

Figure S83. HPTS transport assay of transporter **1a** in BSA-treated POPC vesicles (100 mM NMDG-Cl, 10 mM HEPES, pH 7.0). A base pulse was added to increase the external pH to ~8, followed by the addition of Gramicidin D (0.1 mol% relative to lipid) and subsequently **1a** (5 mol% relative to lipid). Fluorescence is reported as the normalized ratio (dimensionless) versus time (s). Data represents the average of three independent experiments, and shaded regions show the standard deviation.

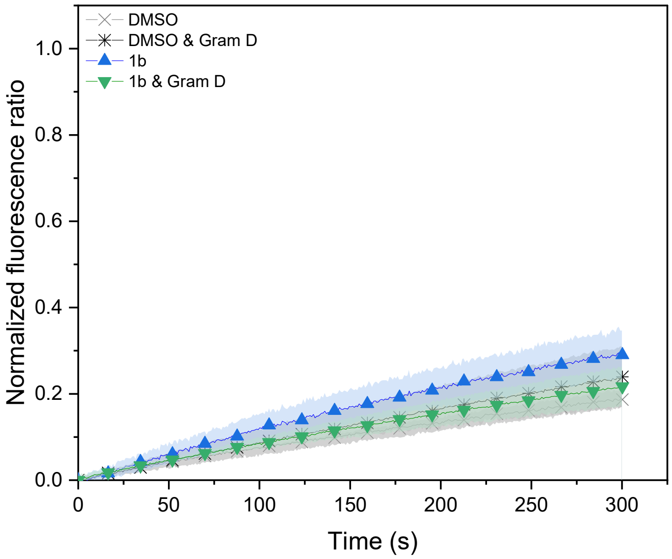

Figure S84. HPTS transport assay of transporter **1b** in BSA-treated POPC vesicles (100 mM NMDG-Cl, 10 mM HEPES, pH 7.0). A base pulse was added to increase the external pH to ~8, followed by the addition of Gramicidin D (0.1 mol% relative to lipid) and subsequently **1b** (5 mol% relative to lipid). Fluorescence is reported as the normalized ratio (dimensionless) versus time (s). Data represents the average of three independent experiments, and shaded regions show the standard deviation.

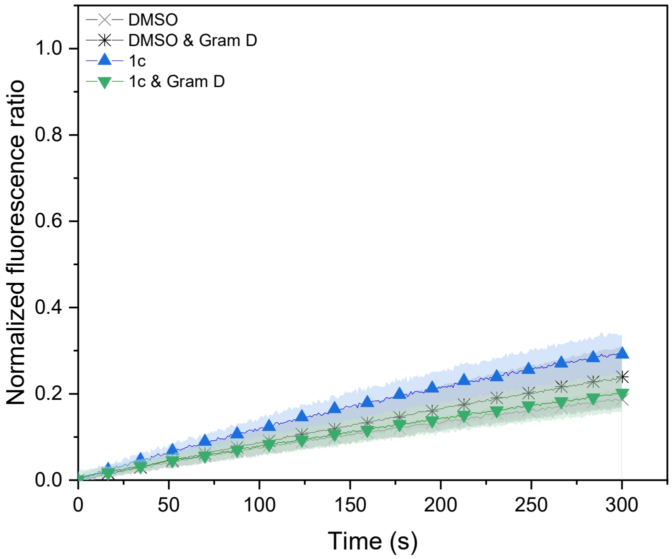

Figure S85. HPTS transport assay of transporter **1c** in BSA-treated POPC vesicles (100 mM NMDG-Cl, 10 mM HEPES, pH 7.0). A base pulse was added to increase the external pH to ~8, followed by the addition of Gramicidin D (0.1 mol% relative to lipid) and subsequently **1c** (5 mol% relative to lipid). Fluorescence is reported as the normalized ratio (dimensionless) versus time (s). Data represents the average of three independent experiments, and shaded regions show the standard deviation.

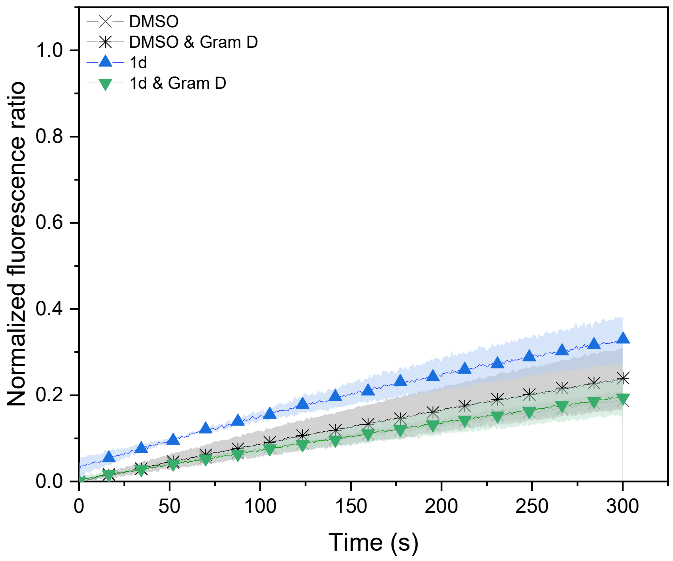

Figure S86. HPTS transport assay of transporter **1d** in BSA-treated POPC vesicles (100 mM NMDG-Cl, 10 mM HEPES, pH 7.0). A base pulse was added to increase the external pH to ~8, followed by the addition of Gramicidin D (0.1 mol% relative to lipid) and subsequently **1d** (5 mol% relative to lipid). Fluorescence is reported as the normalized ratio (dimensionless) versus time (s). Data represents the average of three independent experiments, and shaded regions show the standard deviation.

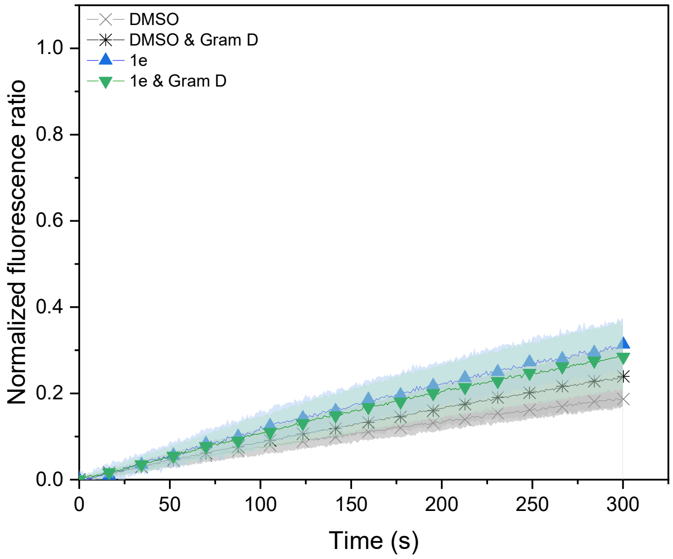

Figure S87. HPTS transport assay of transporter **1e** in BSA-treated POPC vesicles (100 mM NMDG-Cl, 10 mM HEPES, pH 7.0). A base pulse was added to increase the external pH to ~8, followed by the addition of Gramicidin D (0.1 mol% relative to lipid) and subsequently **1e** (5 mol% relative to lipid). Fluorescence is reported as the normalized ratio (dimensionless) versus time (s). Data represents the average of three independent experiments, and shaded regions show the standard deviation.

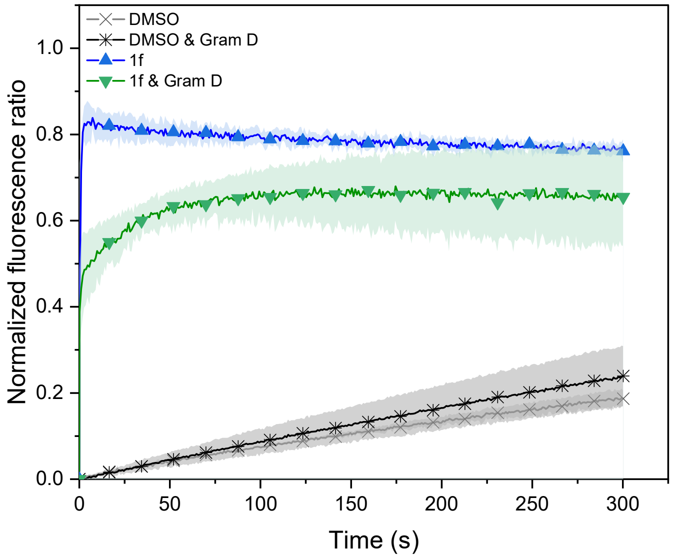

Figure S88. HPTS transport assay of transporter **1f** in BSA-treated POPC vesicles (100 mM NMDG-Cl, 10 mM HEPES, pH 7.0). A base pulse was added to increase the external pH to ~8, followed by the addition of Gramicidin D (0.1 mol% relative to lipid) and subsequently **1f** (5 mol% relative to lipid). Fluorescence is reported as the normalized ratio (dimensionless) versus time (s). Data represents the average of three independent experiments, and shaded regions show the standard deviation.

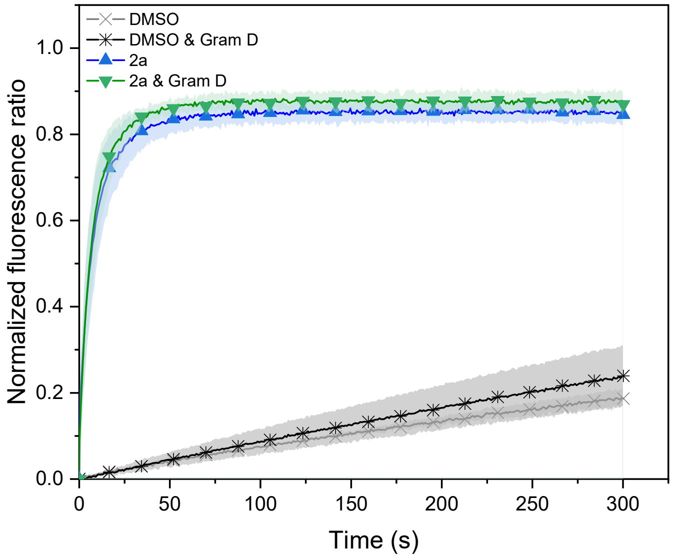

Figure S89. HPTS transport assay of transporter **2a** in BSA-treated POPC vesicles (100 mM NMDG-Cl, 10 mM HEPES, pH 7.0). A base pulse was added to increase the external pH to ~8, followed by the addition of Gramicidin D (0.1 mol% relative to lipid) and subsequently **2a** (0.5 mol% relative to lipid). Fluorescence is reported as the normalized ratio (dimensionless) versus time (s). Data represents the average of three independent experiments, shaded regions show the standard deviation.

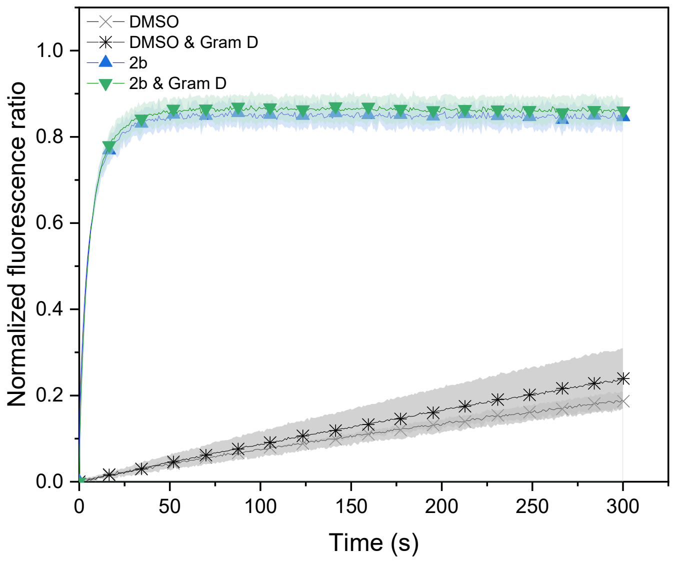

Figure S90. HPTS transport assay of transporter **2b** in BSA-treated POPC vesicles (100 mM NMDG-Cl, 10 mM HEPES, pH 7.0). A base pulse was added to increase the external pH to ~8, followed by the addition of Gramicidin D (0.1 mol% relative to lipid) and subsequently **2b** (0.5 mol% relative to lipid). Fluorescence is reported as the normalized ratio (dimensionless) versus time (s). Data represents the average of three independent experiments, and shaded regions show the standard deviation.

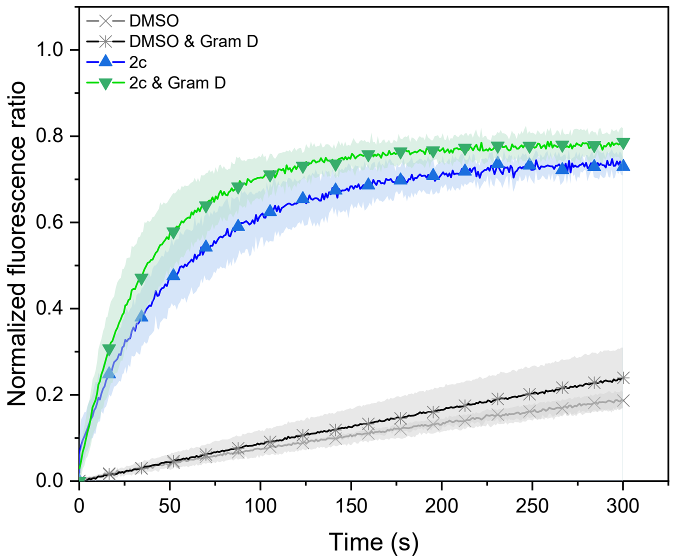

Figure S91. HPTS transport assay of transporter **2c** in BSA-treated POPC vesicles (100 mM NMDG-Cl, 10 mM HEPES, pH 7.0). A base pulse was added to increase the external pH to ~8, followed by the addition of Gramicidin D (0.1 mol% relative to lipid) and subsequently **2c** (5 mol% relative to lipid). Fluorescence is reported as the normalized ratio (dimensionless) versus time (s). Data represents the average of three independent experiments, and shaded regions show the standard deviation.

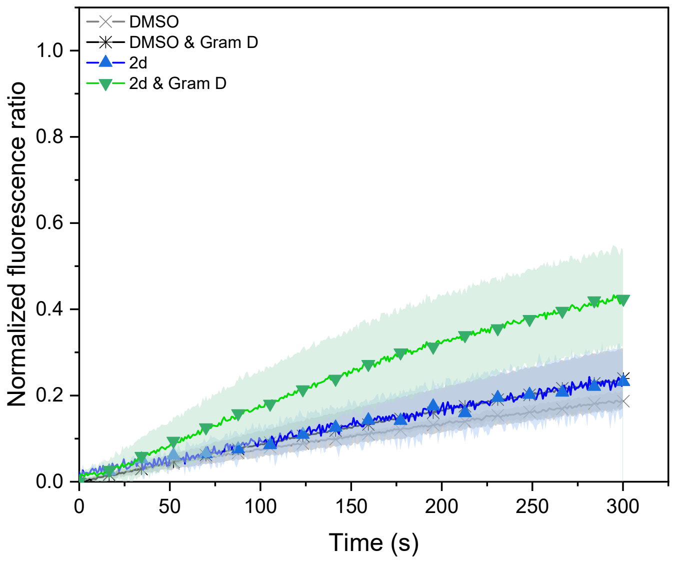

Figure S92. HPTS transport assay of transporter **2d** in BSA-treated POPC vesicles (100 mM NMDG-Cl, 10 mM HEPES, pH 7.0). A base pulse was added to increase the external pH to ~8, followed by the addition of Gramicidin D (0.1 mol% relative to lipid) and subsequently **2d** (5 mol% relative to lipid). Fluorescence is reported as the normalized ratio (dimensionless) versus time (s). Data represents the average of three independent experiments, and shaded regions show the standard deviation.

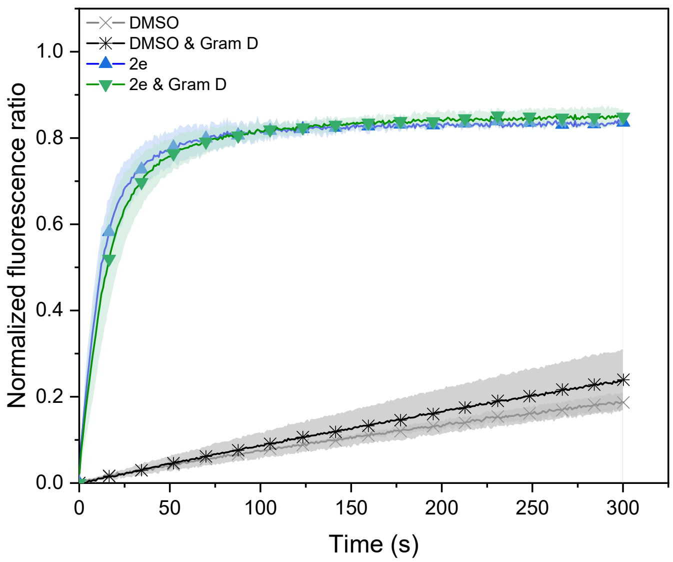

Figure S93. HPTS transport assay of transporter **2e** in BSA-treated POPC vesicles (100 mM NMDG-Cl, 10 mM HEPES, pH 7.0). A base pulse was added to increase the external pH to ~8, followed by the addition of Gramicidin D (0.1 mol% relative to lipid) and subsequently **2e** (5 mol% relative to lipid). Fluorescence is reported as the normalized ratio (dimensionless) versus time (s). Data represents the average of three independent experiments, and shaded regions show the standard deviation.

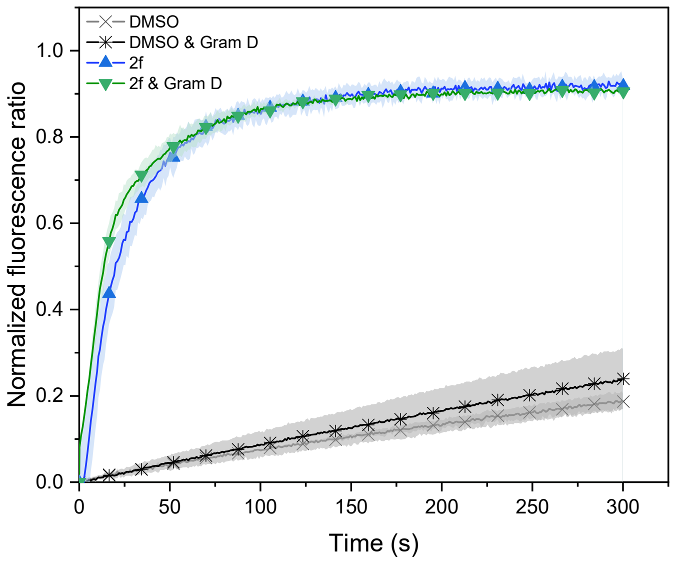

Figure S94. HPTS transport assay of transporter **2f** in BSA-treated POPC vesicles (100 mM NMDG-Cl, 10 mM HEPES, pH 7.0). A base pulse was added to increase the external pH to ~8, followed by the addition of Gramicidin D (0.1 mol% relative to lipid) and subsequently **2f** (5 mol% relative to lipid). Fluorescence is reported as the normalized ratio (dimensionless) versus time (s). Data represents the average of three independent experiments, and shaded regions show the standard deviation.

S6.4.4 Cationophore Coupled Assay

Small molecules can mediate chloride transport across lipid bilayers via electrogenic or electroneutral mechanisms. Electrogenic transport involves a net charge flow across the membrane, whereas electroneutral transport maintains charge balance either through counter-transport of an ion with the same charge (e.g., Cl^-/OH^- exchange) or cotransport with an oppositely charged ion, such as H^+/Cl^- cotransport. In electroneutral transport, these events are intrinsically coupled. The natural ionophores valinomycin and monensin are used to probe these transport mechanisms.[11] Coupling to valinomycin suggest electrogenic Cl^- transport, whereas coupling to monensin suggests electroneutral Cl^- transport (Cl^-/OH^- or H^+/Cl^-).

POPC was stored as a chloroform solution at -20°C until use. For each experiment, a lipid film was prepared in a round-bottom flask from the chloroform solution under reduced pressure using a rotary evaporator and further dried under vacuum for at least 4 hours. The dried lipid film was hydrated with 300 mM KCl solution buffered to pH 7.4 using 5 mM HEPES. The hydrated lipid suspension was subjected to nine freeze–thaw cycles (alternating between liquid nitrogen and warm water) and equilibrated at room temperature for 30 min. The suspension was then extruded 25 times through a 200 nm polycarbonate membrane (Nucleopore™) using an Avanti mini-extruder. To remove unencapsulated KCl, the liposome suspension was dialyzed overnight

(Spectra/Por® 2 Membrane, MWCO 12–14 kD) against an external buffer (300 mM potassium gluconated in 5 mM HEPES buffer at pH 7.4). After dialysis, the obtained LUVs were diluted in the external gluconate buffer to achieve a final lipid concentration of 1 mM. 5 mL of this suspension was transferred to a 20 mL vial with stir bar, and a chloride ion selective electrode (ISE) was inserted into the solution. The suspension was stirred at 350 rpm for 1 minute to equilibrate, at which point a DMSO solution was added containing either the test compound (5 mol% relative to lipid; except **2a** and **2b** which were used at 0.5 mol%), valinomycin (0.1 mol%), monensin (0.1 mol%), or a combination of test compound and valinomycin or monensin. To overcome the deliverability limitations, 250 µl of **2f** from the stock solution was added to achieve a final DMSO concentration of 5% (v/v) in the sample. Chloride efflux was monitored for 5 minutes upon transporter addition using the ISE. Subsequently, the vesicles were lysed with 50 µL of 10% Triton X-100, and a final measurement was taken at 7 minutes. Electrode readings were converted to external chloride concentration ($[Cl^-]$) using a standard calibration curve obtained according to the manufacturer's instructions. The initial chloride concentration, $[Cl^-]_0$ (after 1 minute equilibration), was defined as 0% chloride efflux, and the final concentration, $[Cl^-]_{final}$ (after 7 minutes), was defined as 100% chloride efflux. All intermediate data points were expressed as percentages relative to these two values. The % chloride efflux was calculated using the following equation:

$$\%\ chloride\ efflux = \frac{[Cl^-] - [Cl^-]_0}{[Cl^-]_{final} - [Cl^-]_0}$$

Experiments were done at least in triplicate on different batches of liposomes. The results are shown in **Figure S95 -Figure S106.**

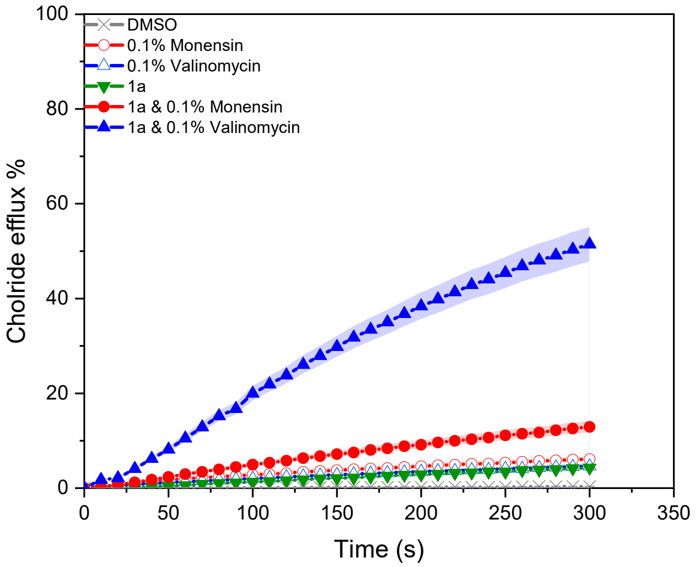

Figure S95. Cationophore-coupled chloride efflux mediated by compound **1a** (5 mol% relative to lipid) in the absence and presence of valinomycin and monensin (each 0.1 mol% relative to lipid). Experiments were performed as described above. Data represents the average of three independent experiments; shaded areas indicate the standard deviation.

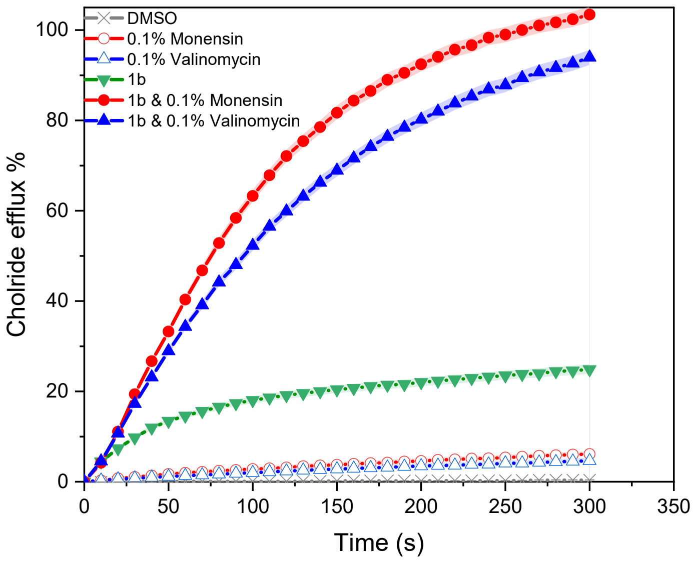

Figure S96. Cationophore-coupled chloride efflux mediated by compound **1b** (5 mol% relative to lipid) in the absence and presence of valinomycin and monensin (each 0.1 mol% relative to lipid). Experiments were performed as described above. Data represents the average of three independent experiments; shaded areas indicate the standard deviation.

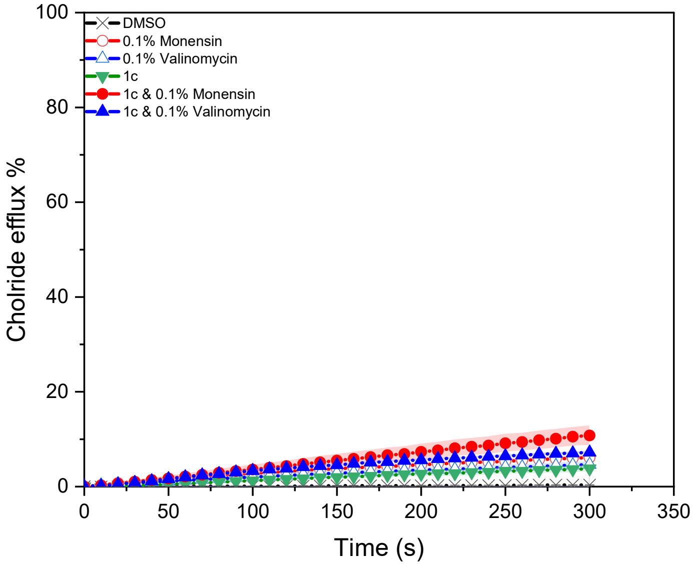

Figure S97. Cationophore-coupled chloride efflux mediated by compound **1c** (5 mol% relative to lipid) in the absence and presence of valinomycin and monensin (each 0.1 mol% relative to lipid). Experiments were performed as described above. Data represents the average of three independent experiments; shaded areas indicate the standard deviation.

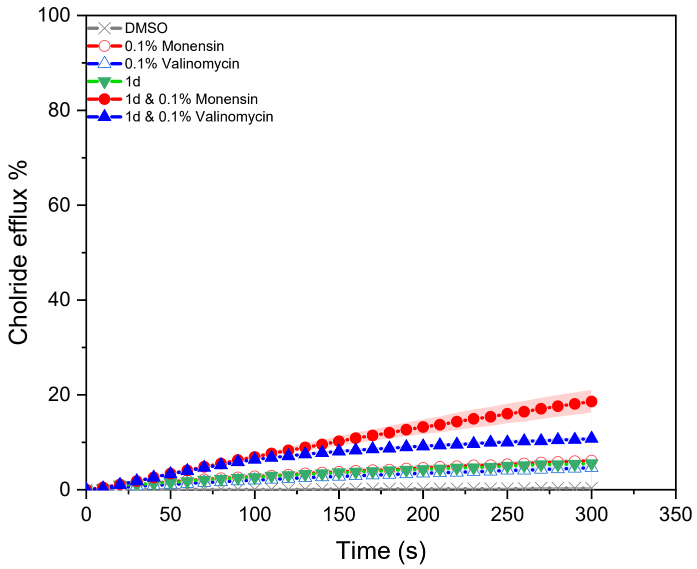

Figure S98. Cationophore-coupled chloride efflux mediated by compound **1d** (5 mol% relative to lipid) in the absence and presence of valinomycin and monensin (each 0.1 mol% relative to lipid). Experiments were performed as described above. Data represents the average of three independent experiments; shaded areas indicate the standard deviation.

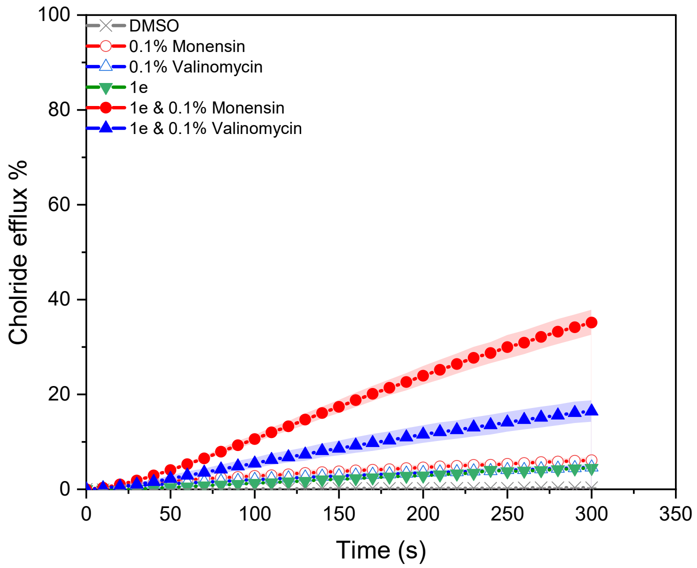

Figure S99. Cationophore-coupled chloride efflux mediated by compound **1e** (5 mol% relative to lipid) in the absence and presence of valinomycin and monensin (each 0.1 mol% relative to lipid). Experiments were performed as described above. Data represents the average of three independent experiments; shaded areas indicate the standard deviation.

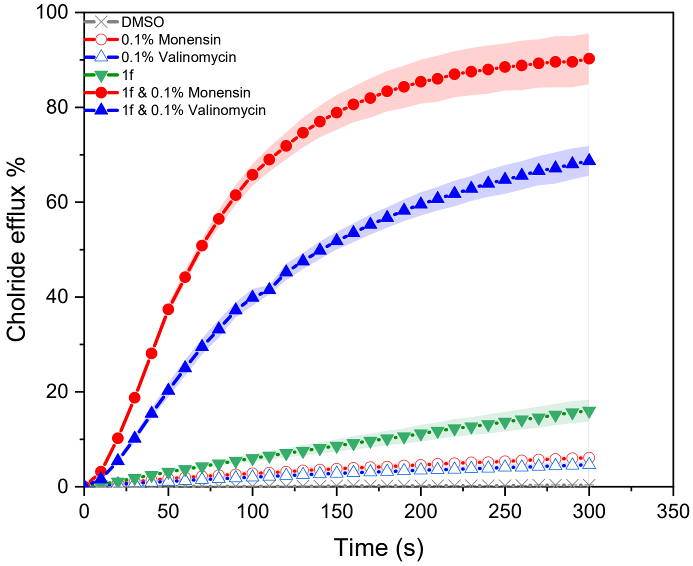

Figure S100. Cationophore-coupled chloride efflux mediated by compound **1f** (5 mol% relative to lipid) in the absence and presence of valinomycin and monensin (each 0.1 mol% relative to lipid). Experiments were performed as described above. Data represents the average of three independent experiments; shaded areas indicate the standard deviation.

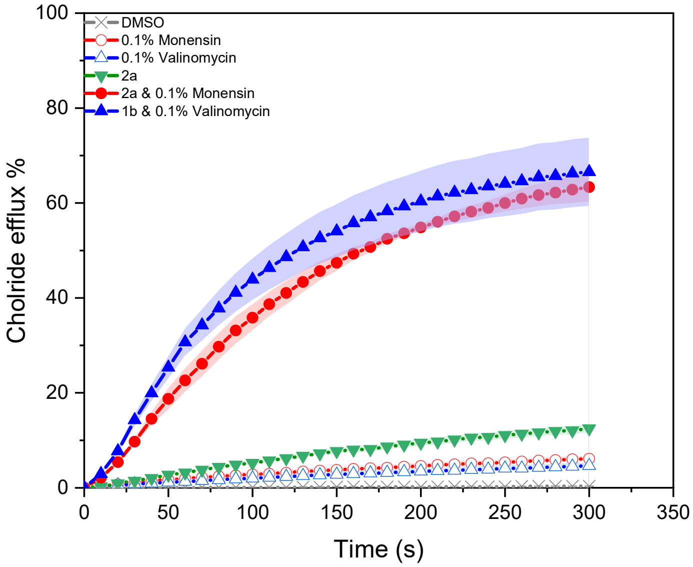

Figure S101. Cationophore-coupled chloride efflux mediated by compound **2a** (0.5 mol% relative to lipid) in the absence and presence of valinomycin and monensin (each 0.1 mol% relative to lipid). Experiments were performed as described above. Data represents the average of three independent experiments; shaded areas indicate the standard deviation.

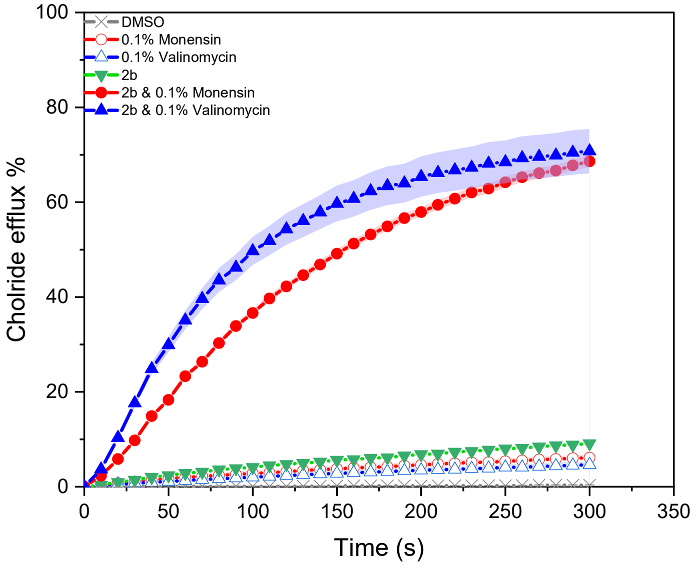

Figure S102. Cationophore-coupled chloride efflux mediated by compound **2b** (0.5 mol% relative to lipid) in the absence and presence of valinomycin and monensin (each 0.1 mol% relative to lipid). Experiments were performed as described above. Data represents the average of three independent experiments; shaded areas indicate the standard deviation.

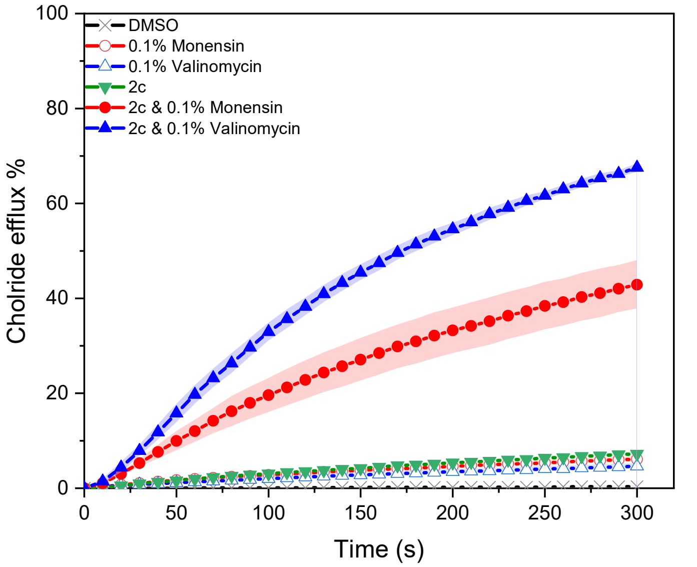

Figure S103. Cationophore-coupled chloride efflux mediated by compound **2c** (5 mol% relative to lipid) in the absence and presence of valinomycin and monensin (each 0.1 mol% relative to lipid). Experiments were performed as described above. Data represents the average of three independent experiments; shaded areas indicate the standard deviation.

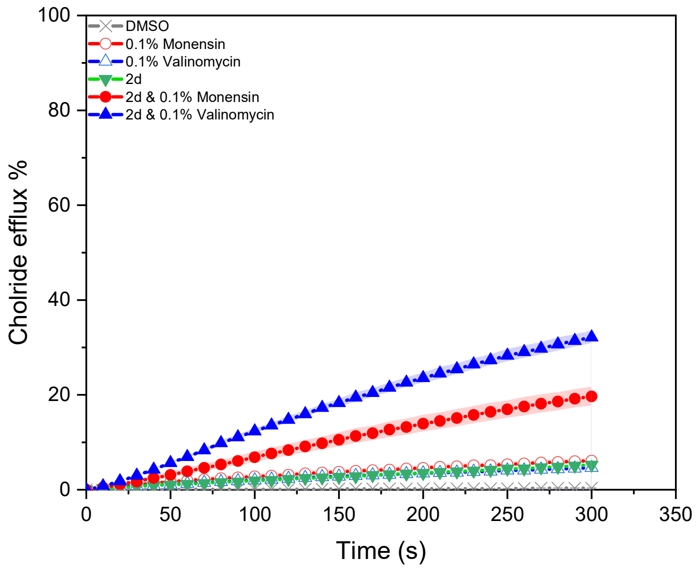

Figure S104. Cationophore-coupled chloride efflux mediated by compound **2d** (5 mol% relative to lipid) in the absence and presence of valinomycin and monensin (each 0.1 mol% relative to lipid). Experiments were performed as described above. Data represents the average of three independent experiments; shaded areas indicate the standard deviation.

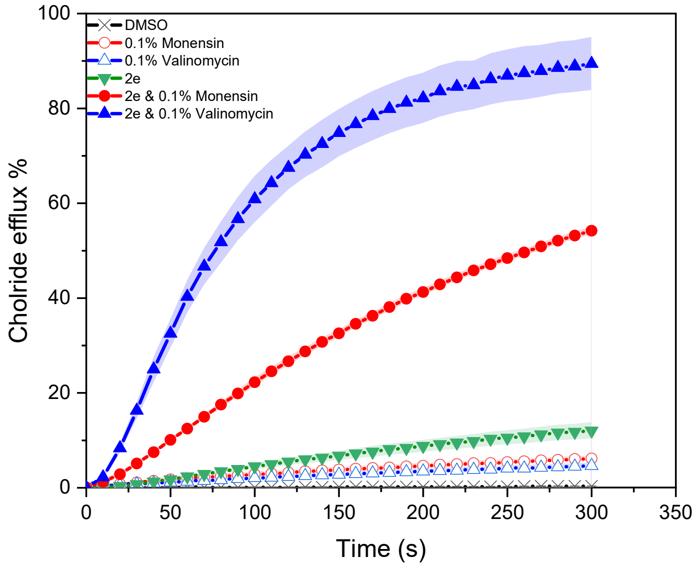

Figure S105. Cationophore-coupled chloride efflux mediated by compound **2e** (5 mol% relative to lipid) in the absence and presence of valinomycin and monensin (each 0.1 mol% relative to lipid). Experiments were performed as described above. Data represents the average of three independent experiments; shaded areas indicate the standard deviation.

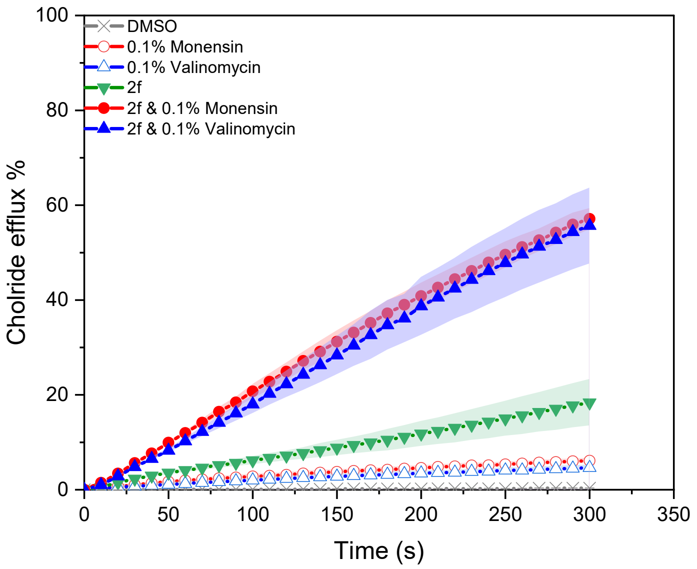

Figure S106. Cationophore-coupled chloride efflux mediated by compound **2f** (5 mol% relative to total lipid; compound added as a DMSO stock solution to achieve a final DMSO concentration of 5% v/v to ensure deliverability) in the absence and presence of valinomycin and monensin (each 0.1 mol% relative to lipid). Experiments were performed as described above. Data represents the average of three independent experiments; shaded areas indicate the standard deviation.

S6.5 pH Dependent Transport

To investigate the chloride transport ability of both the protonated and deprotonated forms of the guanidinium compounds, we employed an ion-selective electrode (ISE) assay. This method was chosen over the lucigenin assay because high OH^- concentrations interfere with the fluorescence signal and the lucigenin assay cannot be used to measure chloride transport for pH > 10.[12] Therefore, to assess how pH influences the chloride transport behavior of these compounds, we performed ISE measurements across a range of pH values from 5.4 to 10.4 (lower pHs lead to instability of the liposomes).

POPC was stored as a chloroform solution at -20°C until use. For each experiment, a lipid film was prepared in a round-bottom flask from the chloroform solution under reduced pressure using a rotary evaporator and further dried under vacuum for at least 4 hours. The dried lipid film was hydrated with 500 mM NaCl solution buffered to the desired pH (internal solution). The buffer composition (10 mM) varied according to pH and was selected using an online buffer calculator: MES buffer for pH 5.4 and 6.4, HEPES buffer for pH 7.4 and 8.4, TAPS buffer for pH 9.4, and phosphate buffer for pH 10.4. Buffers were always prepared fresh using UltraPure water. The hydrated lipid suspension was subjected to nine freeze–thaw cycles (alternating between liquid nitrogen

and warm water) and equilibrated at room temperature for 30 min. The suspension was then extruded 25 times through a 200 nm polycarbonate membrane (Nucleopore™) using an Avanti mini-extruder. To remove unencapsulated NaCl, the liposome suspension was dialyzed overnight (Spectra/Por® 2 Membrane, MWCO 12–14 kD) against an external nitrate buffer. The external buffer composition was adjusted to match the pH of the internal solution, consisting of 500 mM $NaNO_3$ and the corresponding buffering agent (e.g., 10 mM HEPES for pH 7.4). After dialysis, the obtained LUVs were diluted in the external nitrate buffer to achieve a final lipid concentration of 1 mM. 5 mL of this suspension was transferred to a 20 mL vial with stir bar, and the chloride ISE was inserted into the solution. The suspension was stirred at 350 rpm for 1 minute to equilibrate. A methanol (MeOH) solution of the test compound was then added at a concentration of 5 mol% relative to the total POPC lipid concentration, except for compounds **2a** and **2b**, which were used at 0.5 mol%, and **2f**, which was used at 1 mol%. To reach the desired concentration, the compound was dissolved in MeOH, and 50 µL of the stock solution was added to 5 mL of the LUV suspension. For compound **2f,** due to its lower deliverability, 250 µL of the MeOH stock solution was added to the 5 mL sample to achieve 1 mol% relative to the lipid concentration. Chloride efflux was monitored for 5 minutes upon transporter addition using a ISE. Subsequently, the vesicles were lysed with 50 µL of 10% Triton X-100, and a final measurement was taken at 7 minutes. Electrode readings were converted to external chloride concentration ($[Cl^-]$) using a standard calibration curve obtained according to the manufacturer's instructions. The initial chloride concentration, $[Cl^-]_0$ (after 1 minute equilibration), was defined as 0% chloride efflux, and the final concentration, $[Cl^-]_{final}$ (after 7 minutes), was defined as 100% chloride efflux. All intermediate data points were expressed as percentages relative to these two values. The percentage of chloride efflux was calculated using the following equation:

$$\%\, chloride\ efflux = \frac{[Cl^-] - [Cl^-]_0}{[Cl^-]_{final} - [Cl^-]_0}$$

Experiments were done at least in triplicate on different batches of liposomes. The results are shown in **Figure S107-Figure S118.**

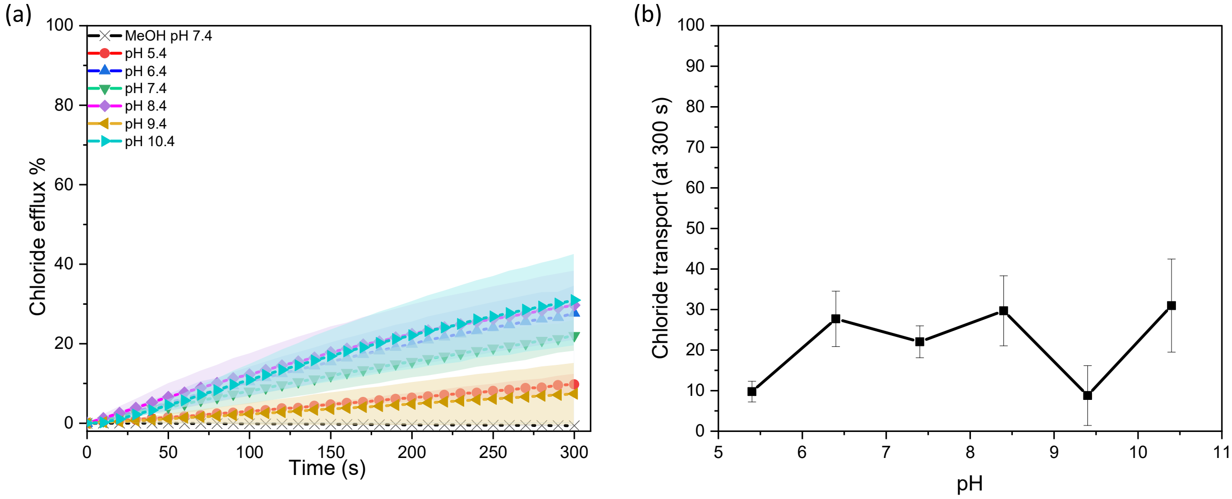

Figure S107. (a) pH-dependent chloride efflux of compound **1a** (5 mol% relative to lipid) measured using POPC liposomes over the pH range 5.4–10.4. (b) Chloride efflux at 300 s plotted against pH, highlighting the limited dependence of transport activity on pH.

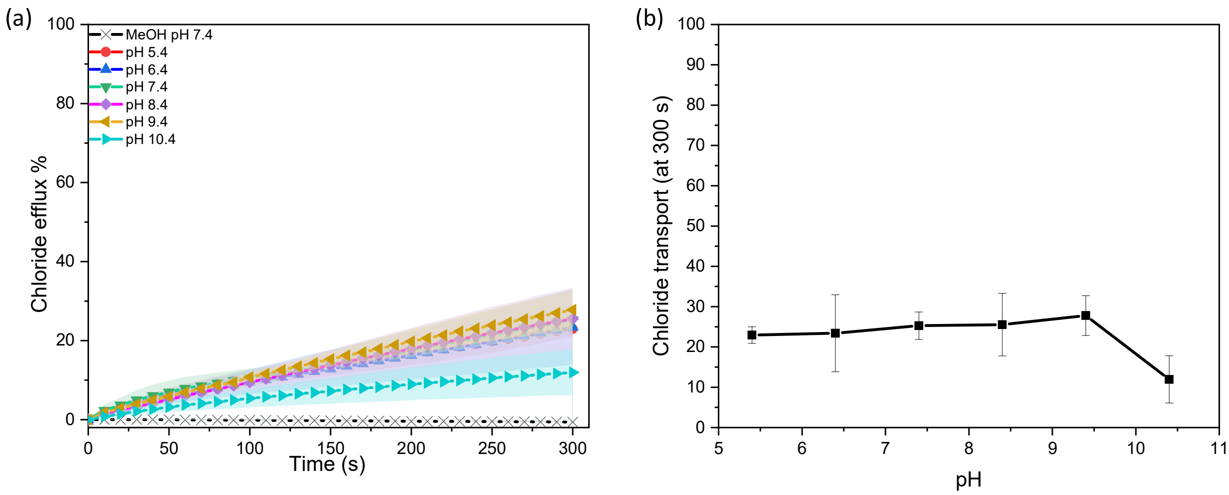

Figure S108. (a) pH-dependent chloride efflux of compound **1b** (5 mol% relative to lipid) measured using POPC liposomes over the pH range 5.4–10.4. (b) Chloride efflux at 300 s plotted against pH, highlighting the limited dependence of transport activity on pH.

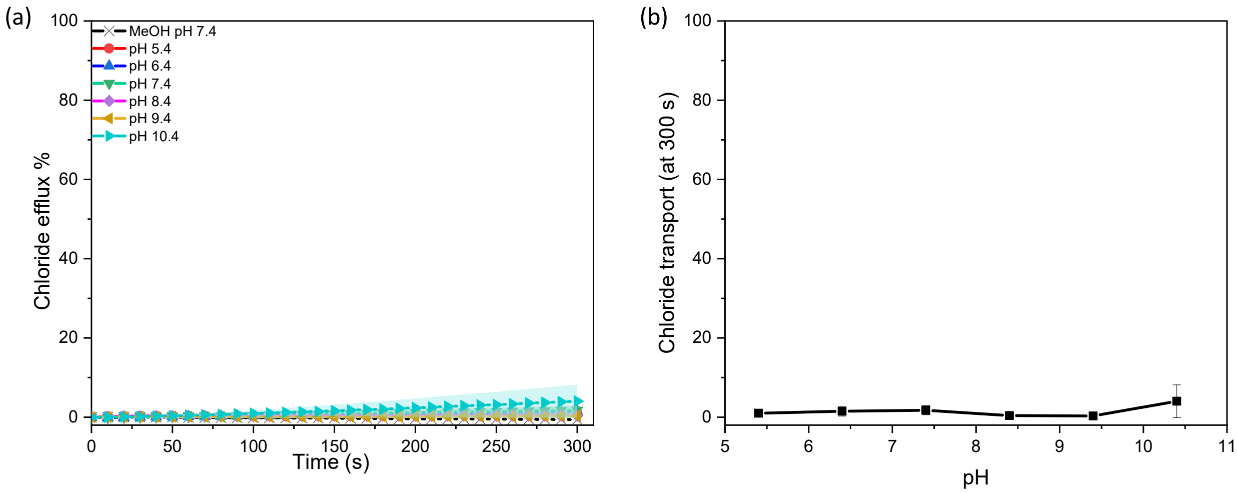

Figure S109. (a) pH-dependent chloride efflux of compound **1c** (5 mol% relative to lipid) measured using POPC liposomes over the pH range 5.4–10.4. (b) Chloride efflux at 300 s plotted against pH, highlighting the dependence of transport activity on pH.

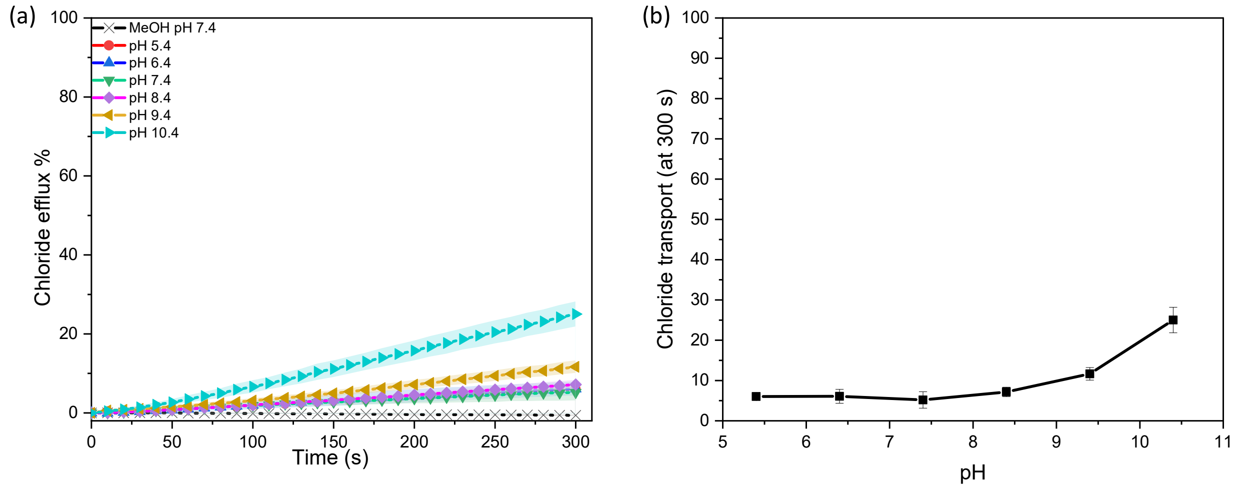

Figure S110. (a) pH-dependent chloride efflux of compound **1d** (5 mol% relative to lipid) measured using POPC liposomes over the pH range 5.4–10.4. (b) Chloride efflux at 300 s plotted against pH, highlighting the dependence of transport activity on pH.

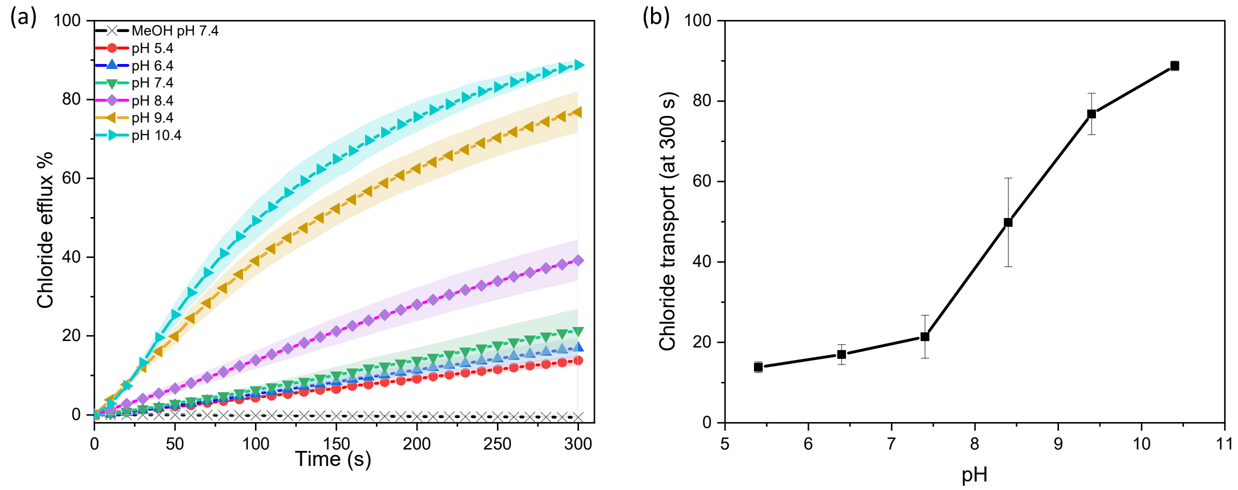

Figure S111. (a) pH-dependent chloride efflux of compound **1e** (5 mol% relative to lipid) measured using POPC liposomes over the pH range 5.4–10.4. (b) Chloride efflux at 300 s plotted against pH, highlighting the dependence of transport activity on pH.

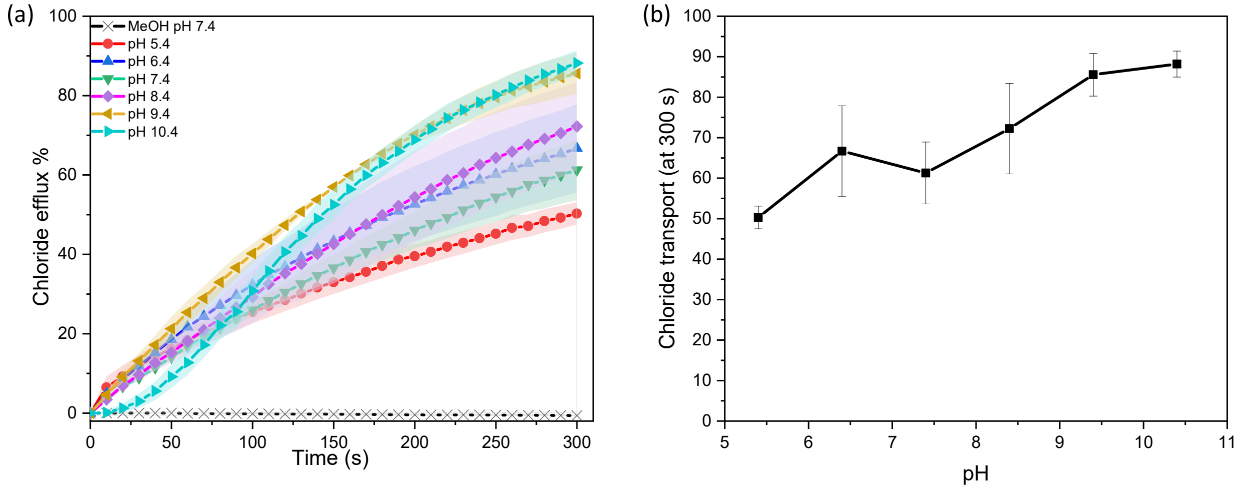

Figure S112. (a) pH-dependent chloride efflux of compound **1f** (5 mol% relative to lipid) measured using POPC liposomes over the pH range 5.4–10.4. (b) Chloride efflux at 300 s plotted against pH, highlighting the dependence of transport activity on pH.

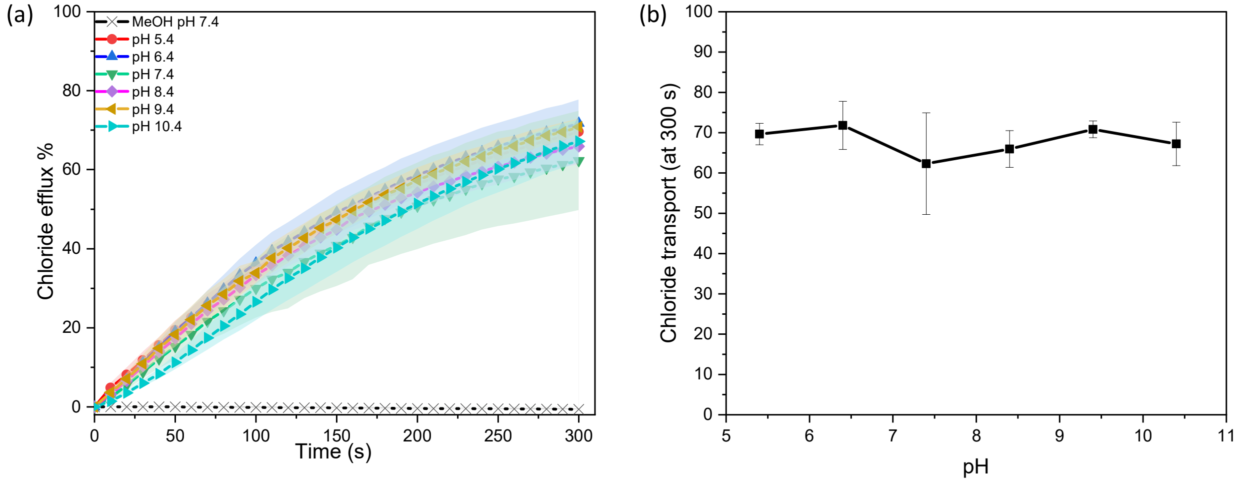

Figure S113. (a) pH-dependent chloride efflux of compound **2a** (0.5 mol% relative to lipid) measured using POPC liposomes over the pH range 5.4–10.4. (b) Chloride efflux at 300 s plotted against pH, highlighting the limited dependence of transport activity on pH.

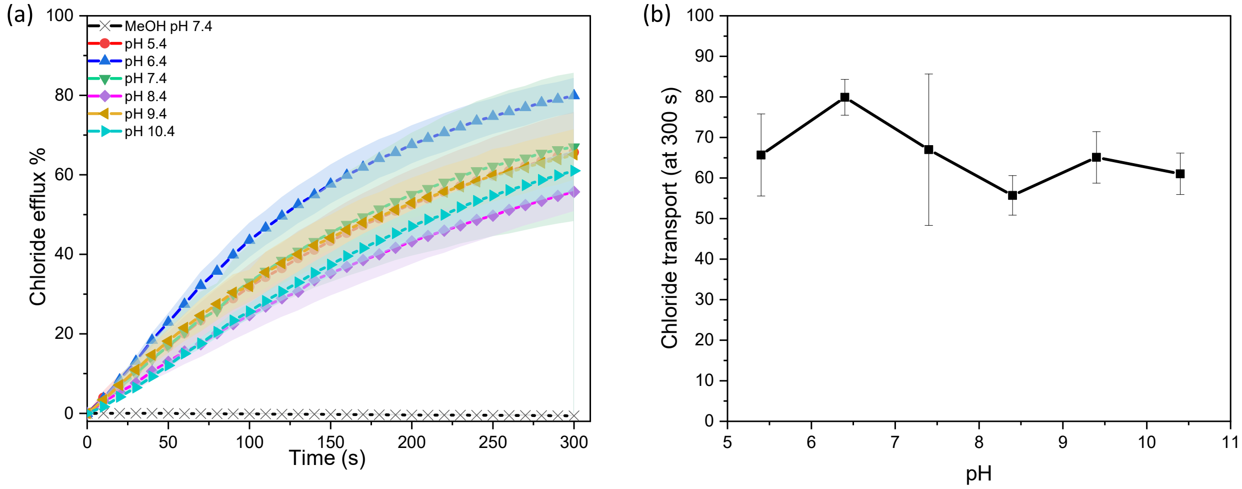

Figure S114. (a) pH-dependent chloride efflux of compound **2b** (0.5 mol% relative to lipid) measured using POPC liposomes over the pH range 5.4–10.4. (b) Chloride efflux at 300 s plotted against pH, highlighting the limited dependence of transport activity on pH.

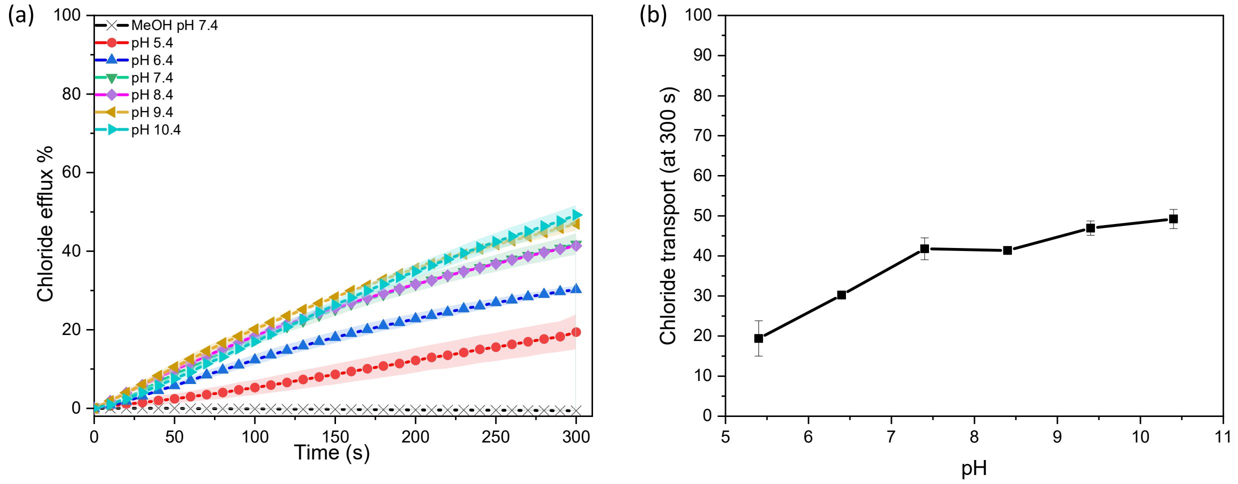

Figure S115. (a) pH-dependent chloride efflux of compound **2c** (5 mol% relative to lipid) measured using POPC liposomes over the pH range 5.4–10.4. (b) Chloride efflux at 300 s plotted against pH, highlighting the dependence of transport activity on pH.

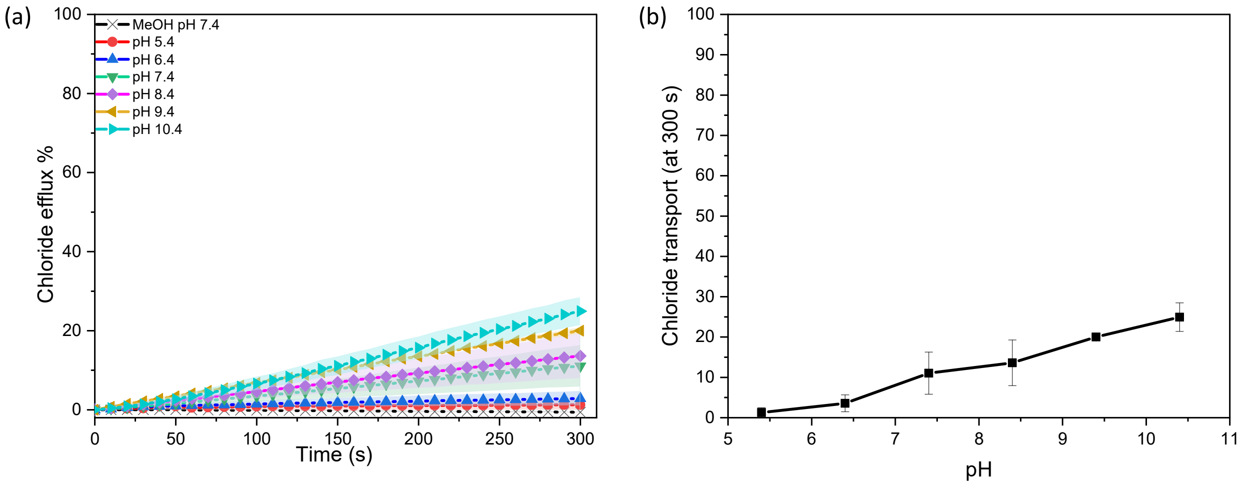

Figure S116. (a) pH-dependent chloride efflux of compound **2d** (5 mol% relative to lipid) measured using POPC liposomes over the pH range 5.4–10.4. (b) Chloride efflux at 300 s plotted against pH, highlighting the dependence of transport activity on pH.

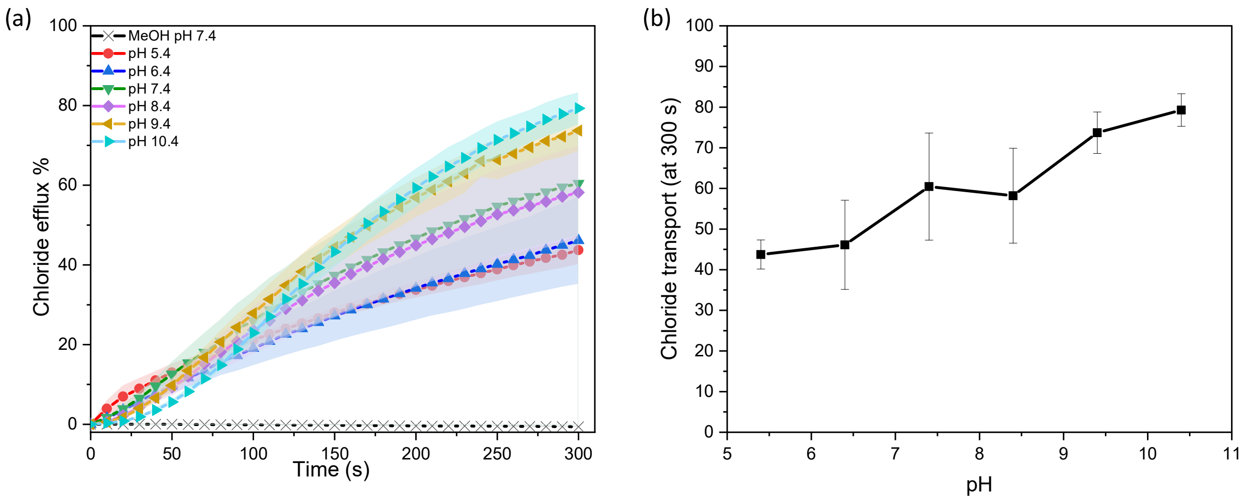

Figure S117. (a) pH-dependent chloride efflux of compound **2e** (5 mol% relative to lipid) measured using POPC liposomes over the pH range 5.4–10.4. (b) Chloride efflux at 300 s plotted against pH, highlighting the dependence of transport activity on pH.

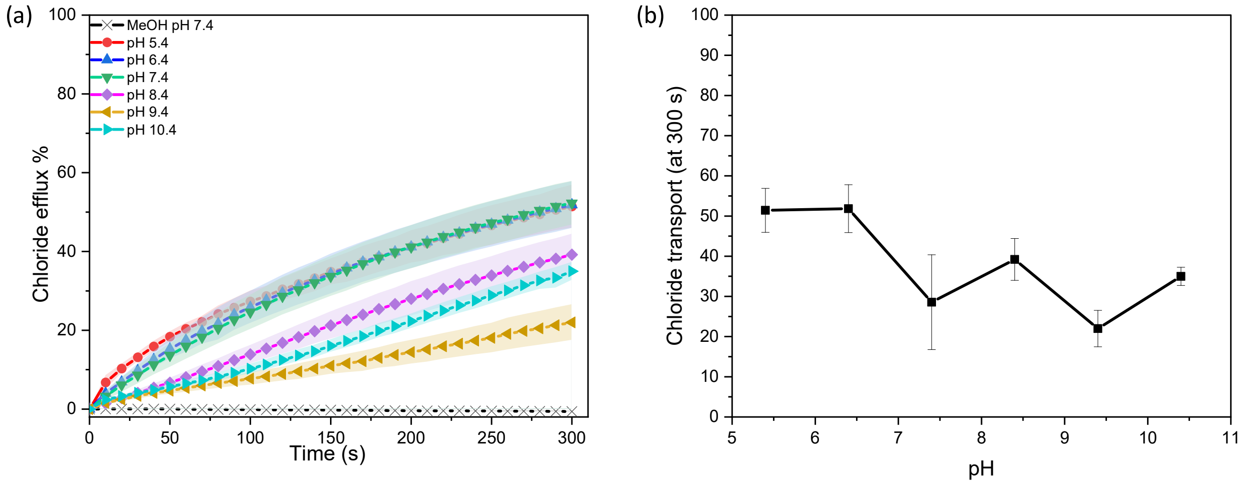

Figure S118. (a) pH-dependent chloride efflux of compound **2f** (1 mol% relative to lipid) measured using POPC liposomes over the pH range 5.4–10.4, with 5% (v/v) MeOH added to improve deliverability. (b) Chloride efflux at 300 s plotted against pH, highlighting the dependence of transport activity on pH.

Compound **2f** exhibited behavior opposite to that observed for the other compounds across different pH values. While the other transporters generally showed enhanced transport activity at higher pH, compound **2f** appeared to be more active at lower pH. We hypothesized that this behavior could arise from a deliverability issue, particularly at higher pH values where the compound is more neutral, less polar, and therefore more lipophilic. To investigate this, the assay was

repeated under the standard conditions used for the other compounds, using a lower solvent volume without additional solvent to improve deliverability, and the transport activity was evaluated at 5 mol% transporter loading. The results are shown in **Figure S119** . Under these conditions, transport activity was significantly lower at higher pH and greater at lower pH, consistent with our hypothesis that deliverability strongly influences the apparent transport behavior of compound **2f**.

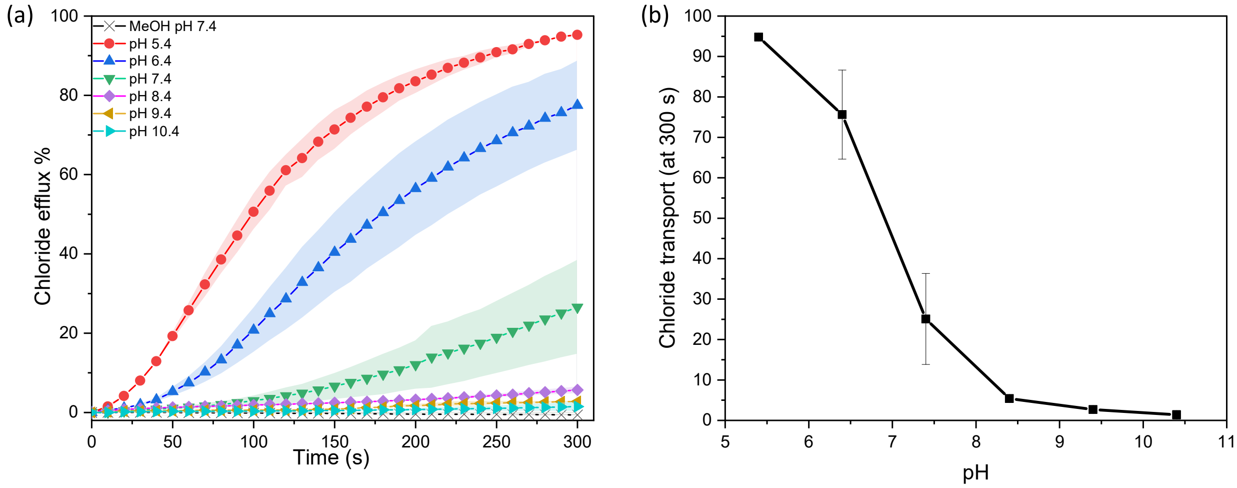

Figure S119. (a) pH-dependent chloride efflux of compound **2f** (5 mol% relative to lipid) measured using POPC liposomes over the pH range 5.4–10.4. (b) Chloride efflux at 300 s plotted against pH, highlighting the dependence of transport activity on pH.

To ensure that the results are due to the intrinsic properties of the transporters and not due to degradation of the compounds at high or low pH, a stability study was also performed. Compounds **2a**, **2b**, **2d** and **2f** were dissolved at a concentration of 100 µM in 1:9 DMSO:buffer (buffer = 500 mM $NaNO_3$, 10 mM MES for pH 5.4, 10 mM HEPES for pH 7.4, or 10 mM phosphate buffer for pH 10.4) and their LC-MS spectra were obtained immediately upon preparation of the samples and again after 3 days. LC-MS traces were collected on a Thermo Fisher Scientific Vanquish Flex UHPLC with variable wavelength detector and ISQ EC mass spectrometer, using a Hypersil GOLD C18 column (150 mm length, 3.0 mm diameter, 3 µm particle size). ‘Solvent A’ was 0.1% (v/v) HCOOH in water and ‘solvent B’ was 0.1% (v/v) HCOOH in acetonitrile. Gradient was from 10% B to 90% B in 15 min, followed by 4 minutes at 90% B. The detection wavelength was set at 250 nm. The results are shown in **Figure S120-S123**. No significant changes in the HPLC traces were observed and no new peaks appeared, indicating that the transporters remain stable under the conditions of the transport assays.

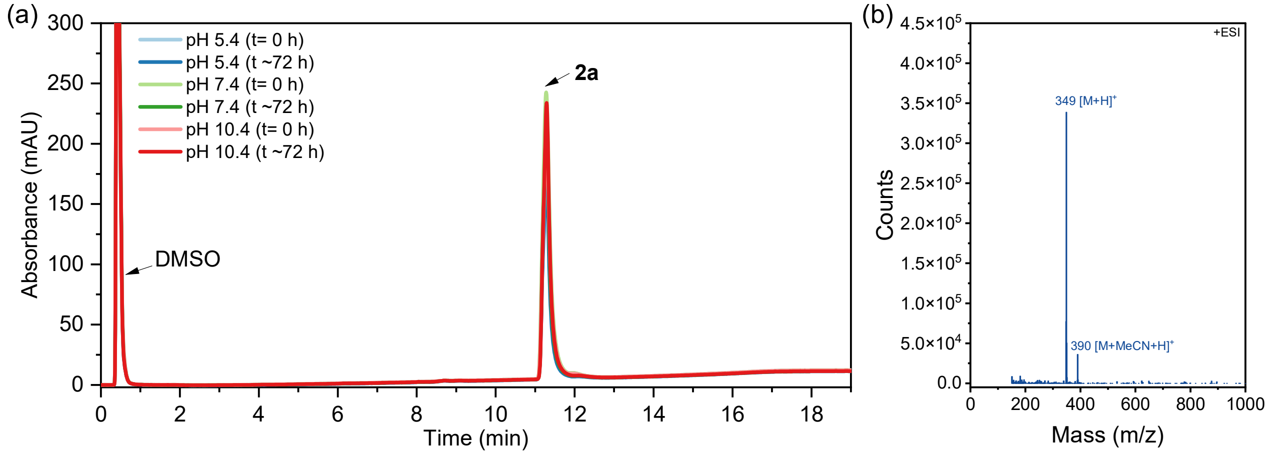

Figure S120. (a) Reverse-phase HPLC trace of **2a** dissolved in 1:9 DMSO:buffer (buffer at the indicated pH) obtained immediately after dissolving the compound (0 h) and after 3 days in solution (72 h). (b) Low resolution mass spectrum (ESI+) of the peak with a retention time of *t* = 11-12 min. Expected mass for **2a**: *m/z* = 349 $[M+H]^+$.

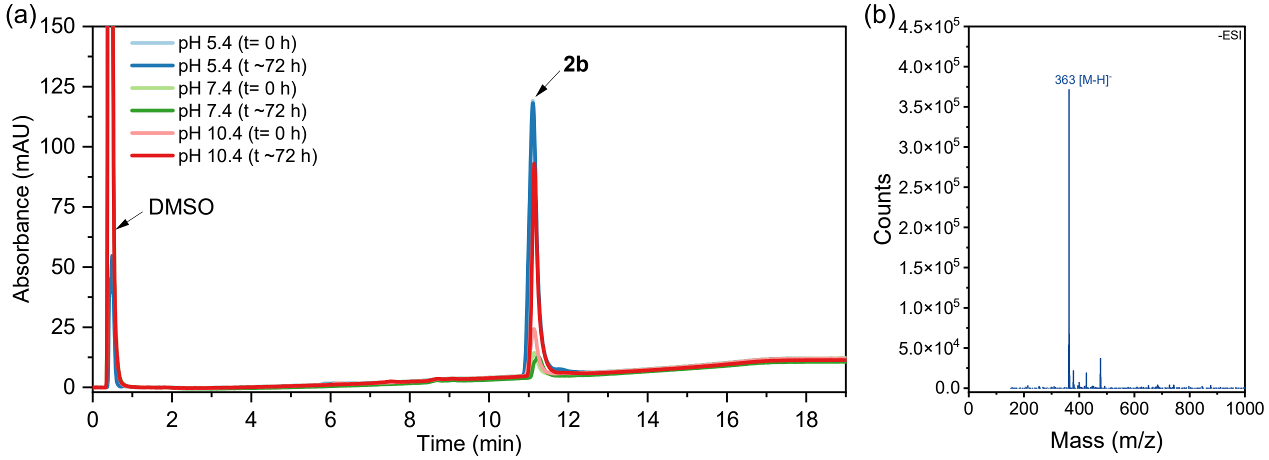

Figure S121. (a) Reverse-phase HPLC trace of **2b** dissolved in 1:9 DMSO:buffer (buffer at the indicated pH) obtained immediately after dissolving the compound (0 h) and after 3 days in solution (72 h). (b) Low resolution mass spectrum (ESI-) of the peak with a retention time of *t* = 11-12 min. Expected mass for **2b**: *m/z* = 363 $[M-H]^-$.

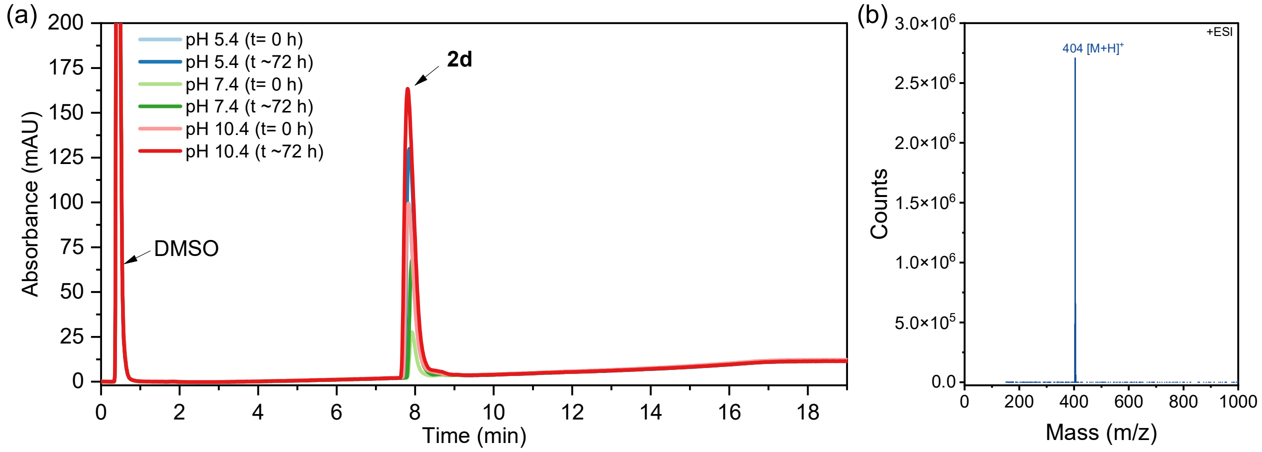

Figure S122. (a) Reverse-phase HPLC trace of **2d** dissolved in 1:9 DMSO:buffer (buffer at the indicated pH) obtained immediately after dissolving the compound (0 h) and after 3 days in solution (72 h). (b) Low resolution mass spectrum (ESI+) of the peak with a retention time of *t* = 7.5-8.5 min. Expected mass for **2d**: *m/z* = 404 $[M+H]^+$.

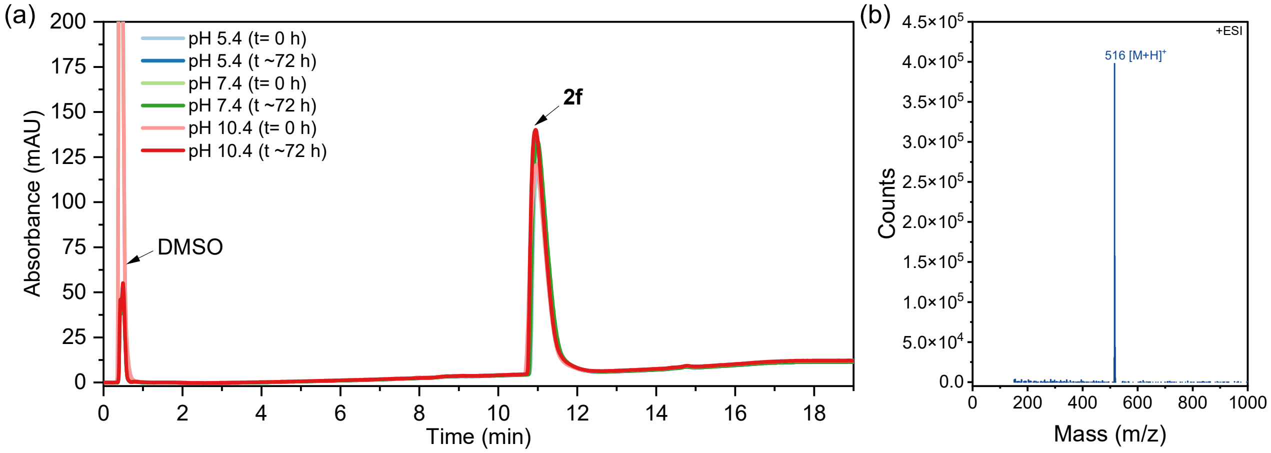

Figure S123. (a) Reverse-phase HPLC trace of **2f** dissolved in 1:9 DMSO:buffer (buffer at the indicated pH) obtained immediately after dissolving the compound (0 h) and after 3 days in solution (72 h). (b) Low resolution mass spectrum (ESI+) of the peak with a retention time of *t* = 11-12 min. Expected mass for **2f**: *m/z* = 516 $[M+H]^+$.

S6.6 Lipid Dependent Transport

Based on the results described in *Section 6.5*, chloride transport was further examined at pH 5.4, 7.4, and 10.4 to investigate the influence of both pH and lipid composition on transport efficiency. These pH values were selected to represent the predominantly protonated (pH 5.4), physiological (pH 7.4), and deprotonated (pH 10.4) states of the compounds. Liposomes were prepared using three different lipid compositions: (i) POPC alone, (ii) 3:7 cholesterol:POPC mixtures, and (iii) negatively charged POPG. This comparison aimed to elucidate how variations in membrane

composition affect compound-membrane interactions and chloride transport. The experiments were conducted as described in *Section 6.5*, except that the initial lipid that was dried corresponds to either 3:7 cholesterol:POPC or POPG. The results are shown in **Figure S124-Figure S126**.

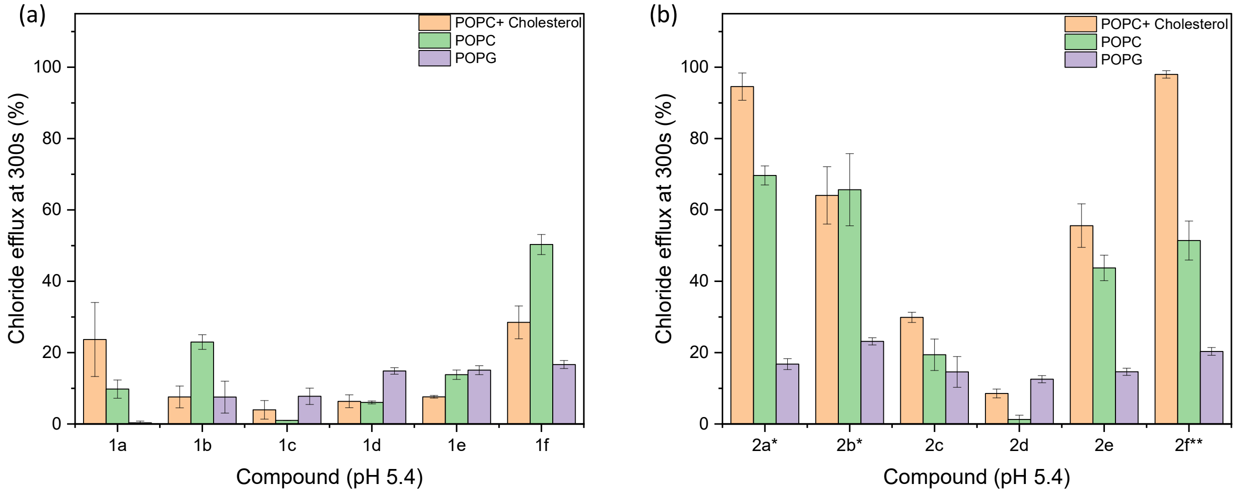

Figure S124. Chloride efflux at pH 5.4 using liposomes prepared with three different lipid compositions: 7:3 POPC:cholesterol mixture, POPC alone, and negatively charged POPG. Experiments were conducted as described in *Sections S6.5* and *S6.6*. (a) Non-fluorinated compounds **1a**–**1f** (5 mol% relative to total lipid). (b) Fluorinated compounds **2a** and **2b** (*0.5 mol% compared to the lipid composition), **2c**–**2e** (5 mol% compared to the lipid composition), and **2f** (** 1 mol% compared to the lipid composition).

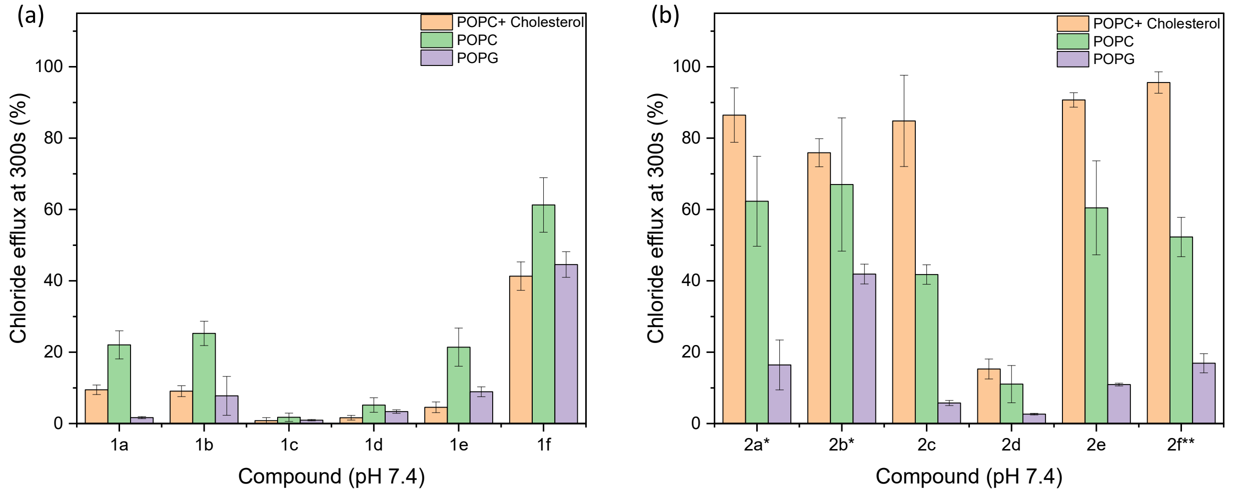

Figure S125. Chloride efflux at pH 7.4 using liposomes prepared with three different lipid compositions: 7:3 POPC/cholesterol mixture, POPC alone, and negatively charged POPG. Experiments were conducted as described in *Sections S6.5* and *S6.6*. (a) Non-fluorinated compounds **1a**–**1f** (5 mol% relative to total lipid). (b) Fluorinated compounds **2a** and **2b** (*0.5 mol% compared to the lipid composition), **2c**–**2e** (5 mol% compared to the lipid composition), and **2f** (** 1 mol% compared to the lipid composition).

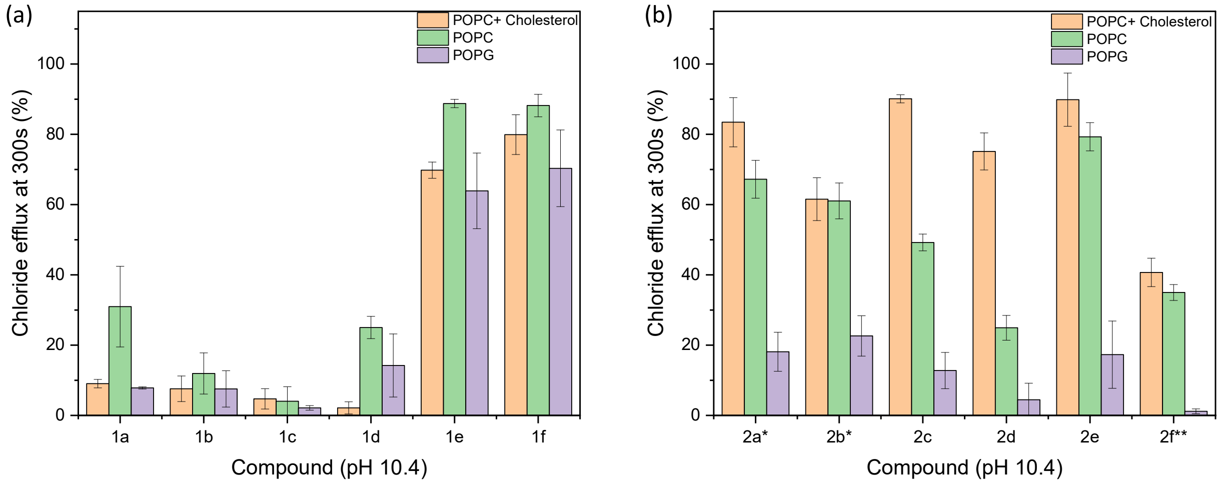

Figure S126. Chloride efflux at pH 10.4 using liposomes prepared with three different lipid compositions: 7:3 POPC/cholesterol mixture, POPC alone, and negatively charged POPG. Experiments were conducted as described in *Sections S6.5* and *S6.6*. (a) Non-fluorinated compounds **1a**–**1f** (5 mol% relative to total lipid). (b) Fluorinated compounds **2a** and **2b** (*0.5 mol% compared to the lipid composition), **2c**–**2e** (5 mol% compared to the lipid composition), and **2f** (** 1 mol% compared to the lipid composition).

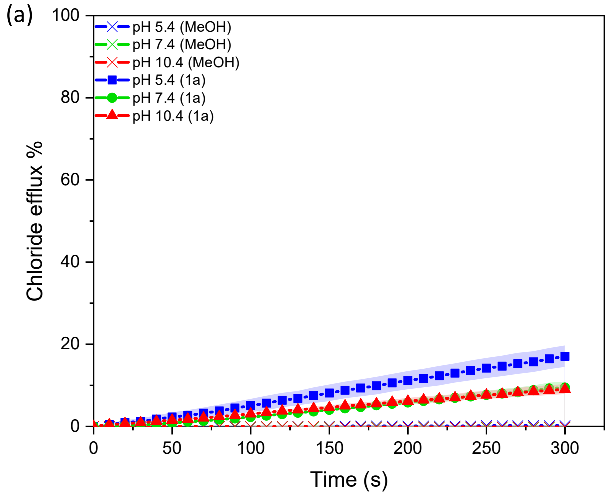

Figure S127. Chloride efflux of compound **1a** measured using an ISE assay (5 mol% relative to lipid) at pH 5.4, 7.4, and 10.4 using liposomes prepared from (a) a 7:3 POPC/cholesterol mixture and (b) POPG. Repeats for all compounds in POPC liposomes are shown in Figure S107-Figure S118.

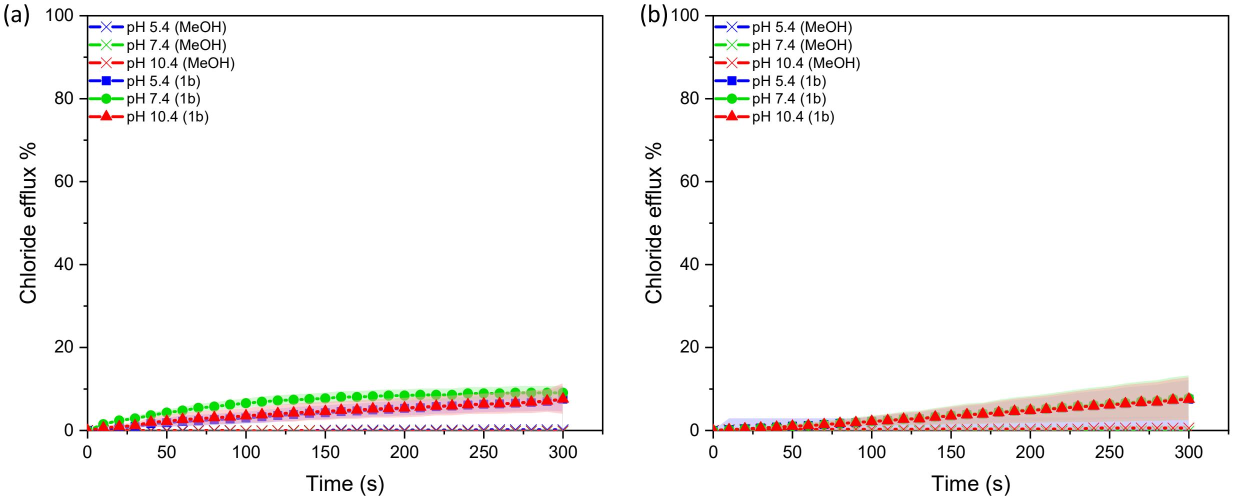

Figure S128. Chloride efflux of compound **1b** measured using an ISE assay (5 mol% relative to lipid) at pH 5.4, 7.4, and 10.4 using liposomes prepared from (a) a 7:3 POPC/cholesterol mixture and (b) POPG. Repeats for all compounds in POPC liposomes are shown in Figure S107-Figure S118.

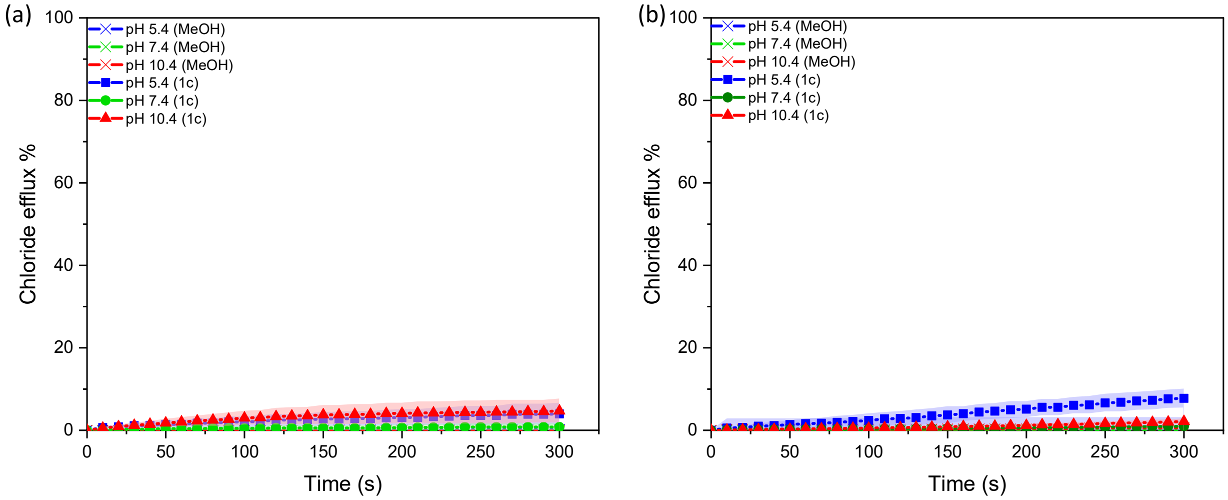

Figure S129. Chloride efflux of compound **1c** measured using an ISE assay (5 mol% relative to lipid) at pH 5.4, 7.4, and 10.4 using liposomes prepared from (a) a 7:3 POPC/cholesterol mixture and (b) POPG. Repeats for all compounds in POPC liposomes are shown in Figure S107-Figure S118.

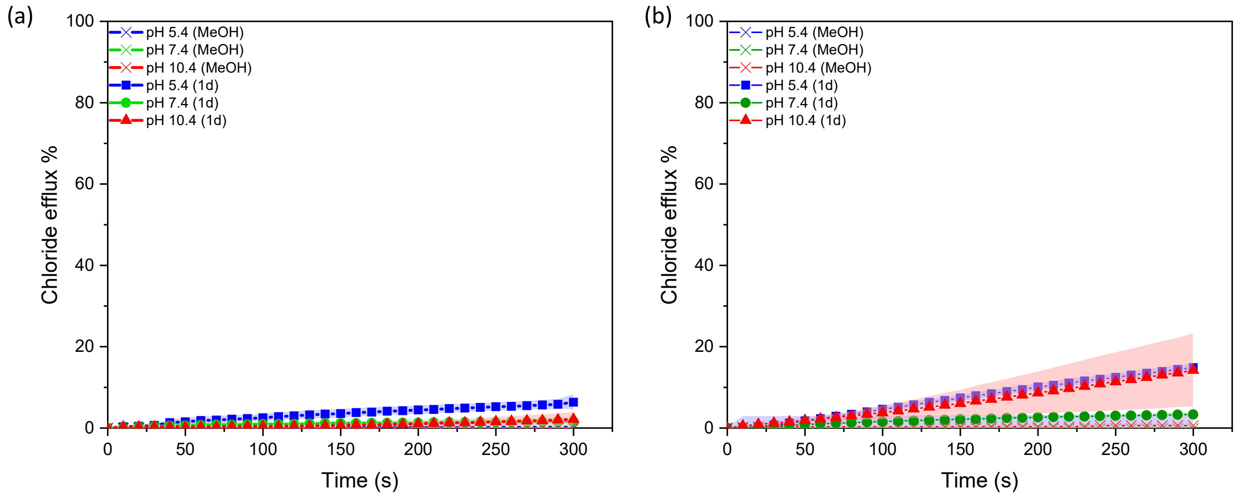

Figure S130. Chloride efflux of compound **1d** measured using an ISE assay (5 mol% relative to lipid) at pH 5.4, 7.4, and 10.4 using liposomes prepared from (a) a 7:3 POPC/cholesterol mixture and (b) POPG. Repeats for all compounds in POPC liposomes are shown in Figure S107-Figure S118.

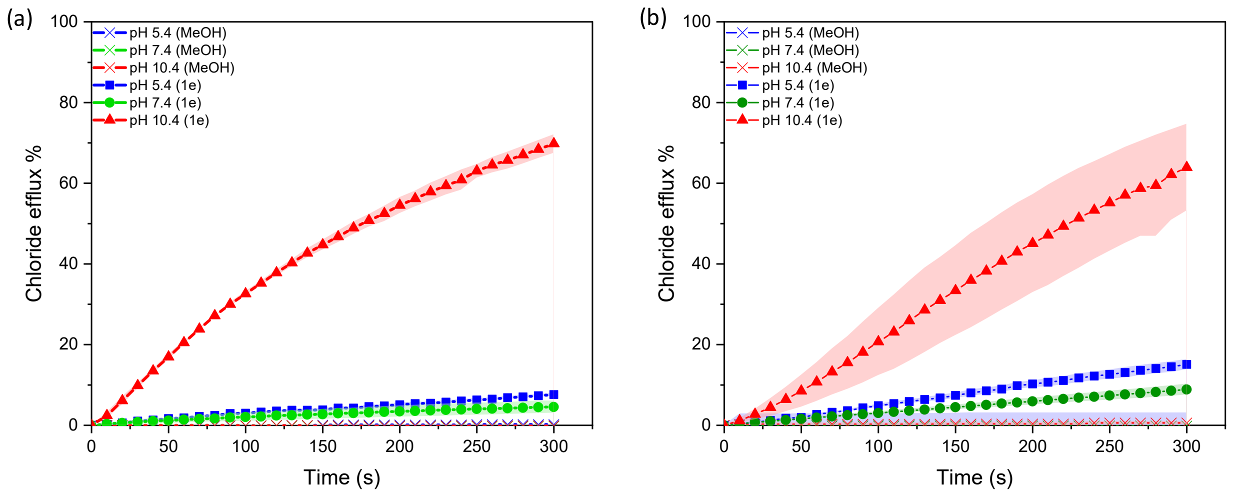

Figure S131. Chloride efflux of compound **1e** measured using an ISE assay (5 mol% relative to lipid) at pH 5.4, 7.4, and 10.4 using liposomes prepared from (a) a 7:3 POPC/cholesterol mixture and (b) POPG. Repeats for all compounds in POPC liposomes are shown in Figure S107-Figure S118.

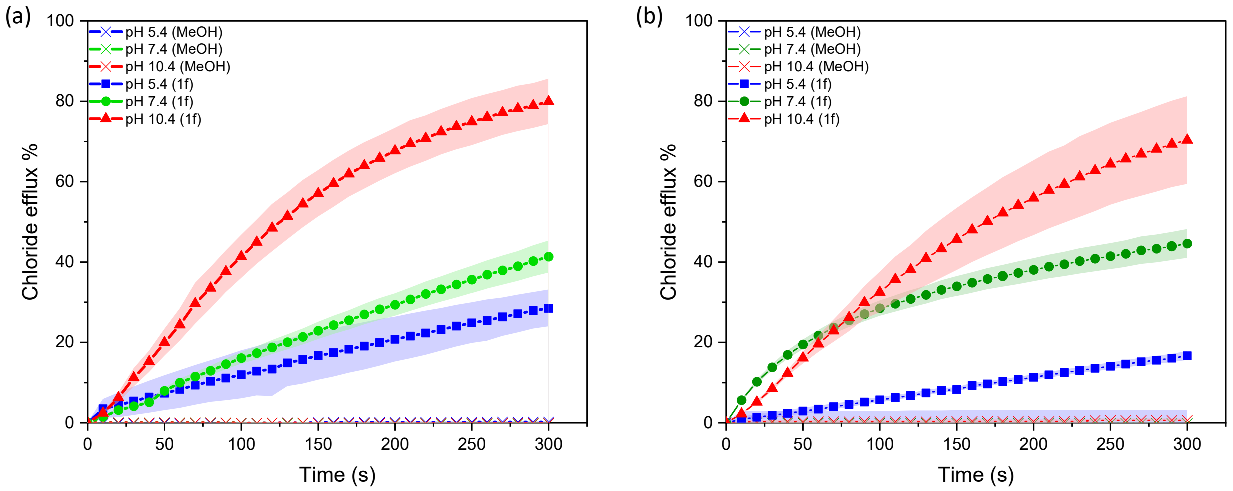

Figure S132. Chloride efflux of compound **1f** measured using an ISE assay (5 mol% relative to lipid) at pH 5.4, 7.4, and 10.4 using liposomes prepared from (a) a 7:3 POPC/cholesterol mixture and (b) POPG. Repeats for all compounds in POPC liposomes are shown in Figure S107-Figure S118.

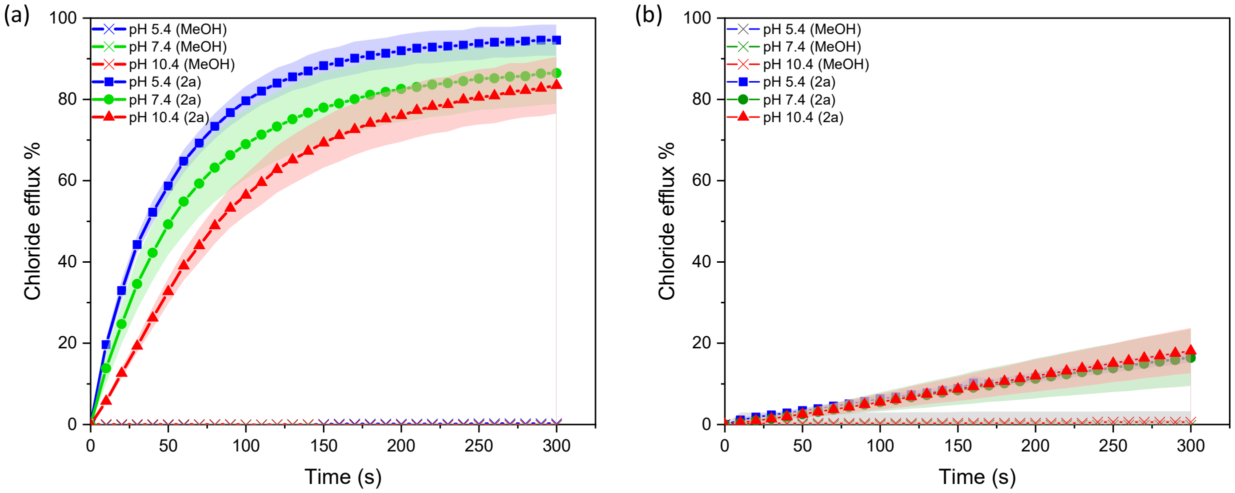

Figure S133. Chloride efflux of compound **2a** measured using an ISE assay (0.5 mol% relative to lipid) at pH 5.4, 7.4, and 10.4 using liposomes prepared from (a) a 7:3 POPC/cholesterol mixture and (b) POPG. Repeats for all compounds in POPC liposomes are shown in Figure S107-Figure S118.

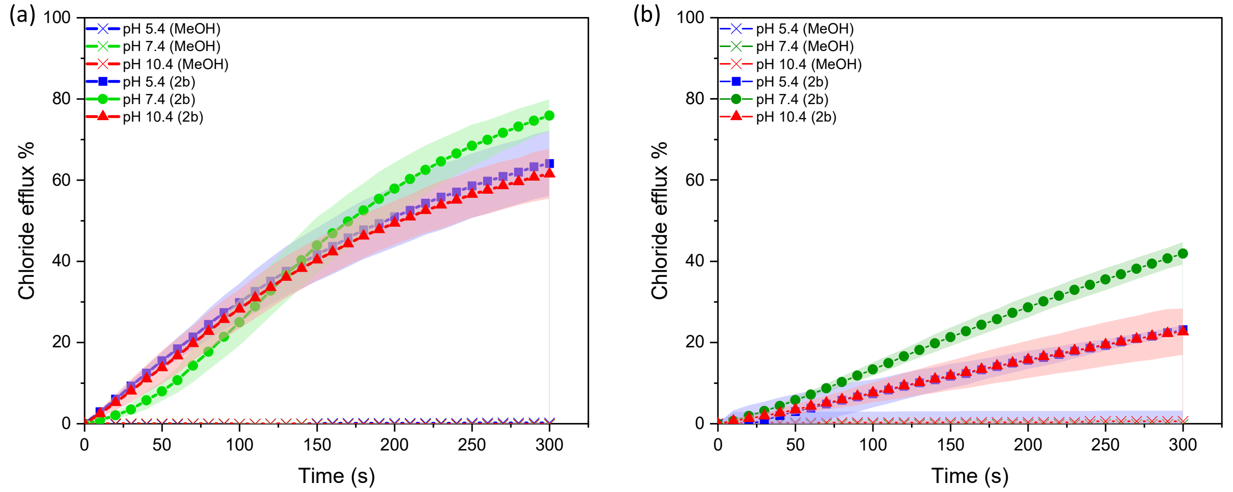

Figure S134. Chloride efflux of compound **2b** measured using an ISE assay (0.5 mol% relative to lipid) at pH 5.4, 7.4, and 10.4 using liposomes prepared from (a) a 7:3 POPC/cholesterol mixture and (b) POPG. Repeats for all compounds in POPC liposomes are shown in Figure S107-Figure S118.

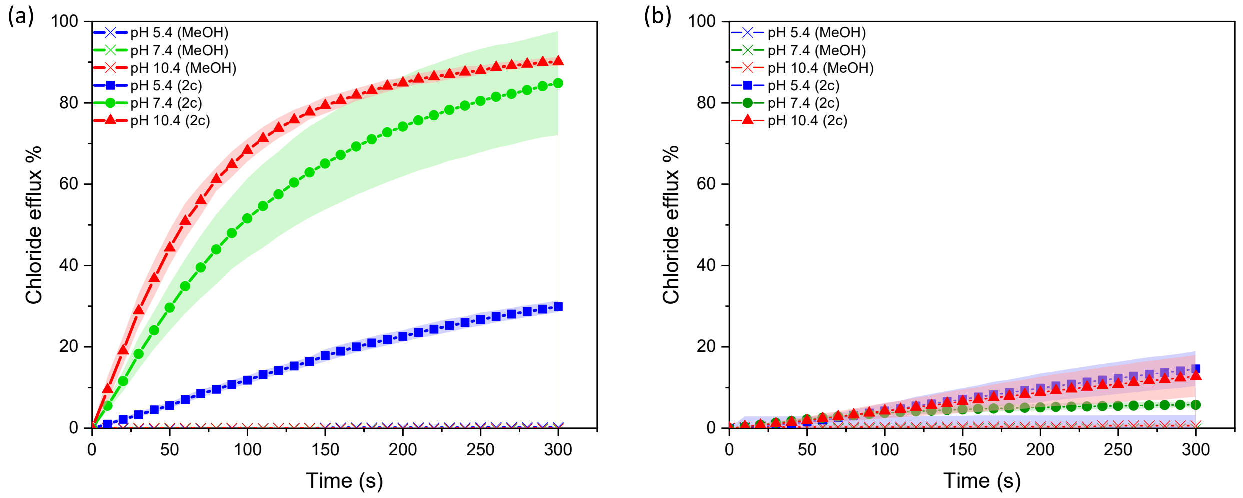

Figure S135. Chloride efflux of compound **2c** measured using an ISE assay (5 mol% relative to lipid) at pH 5.4, 7.4, and 10.4 using liposomes prepared from (a) a 7:3 POPC/cholesterol mixture and (b) POPG. Repeats for all compounds in POPC liposomes are shown in Figure S107-Figure S118.

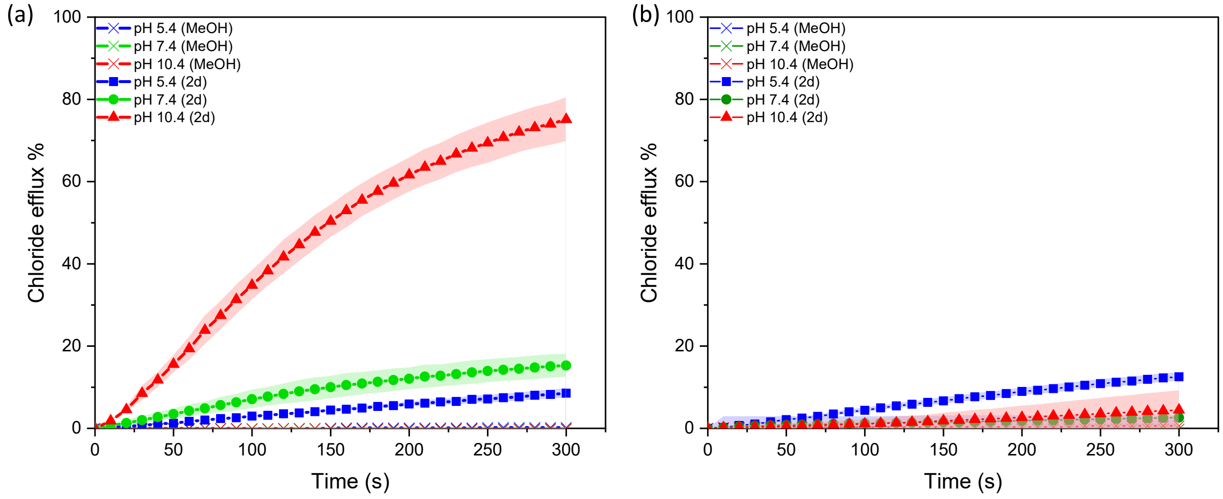

Figure S136. Chloride efflux of compound **2d** measured using an ISE assay (5 mol% relative to lipid) at pH 5.4, 7.4, and 10.4 using liposomes prepared from (a) a 7:3 POPC/cholesterol mixture and (b) POPG. Repeats for all compounds in POPC liposomes are shown in Figure S107-Figure S118.

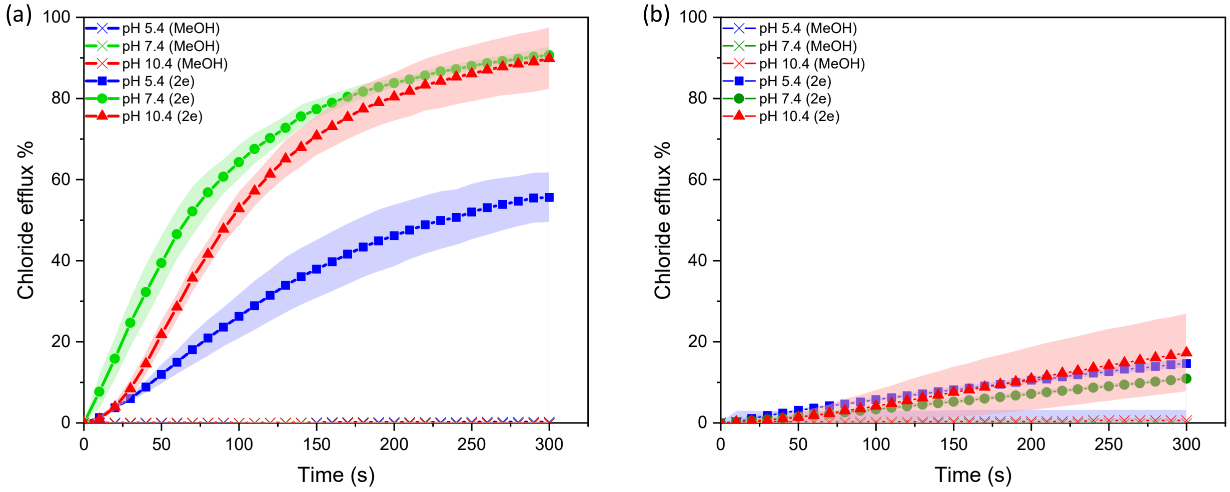

Figure S137. Chloride efflux of compound **2e** measured using an ISE assay (5 mol% relative to lipid) at pH 5.4, 7.4, and 10.4 using liposomes prepared from (a) a 7:3 POPC/cholesterol mixture and (b) POPG. Repeats for all compounds in POPC liposomes are shown in Figure S107-Figure S118.

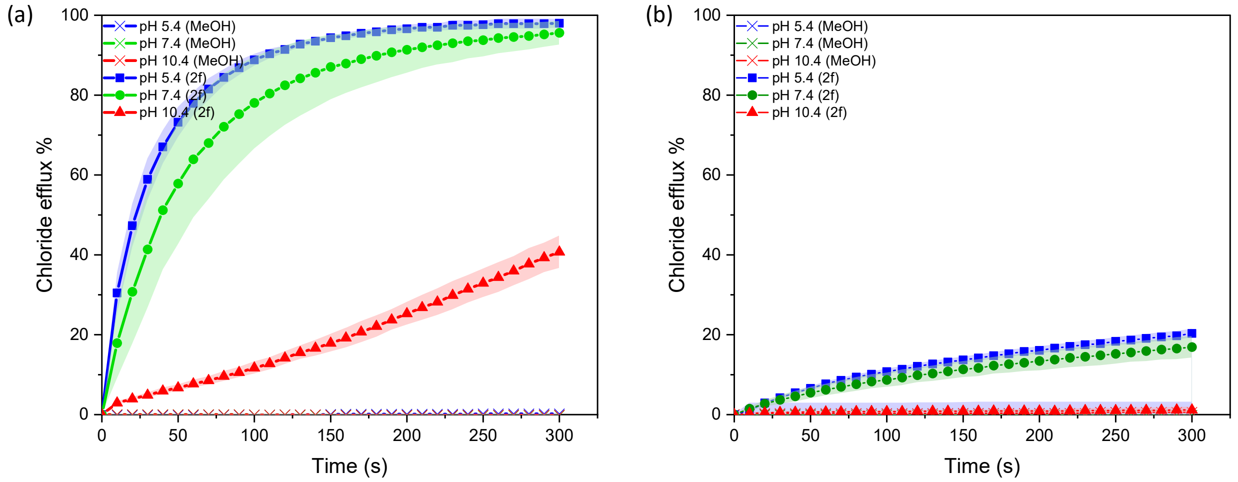

Figure S138. Chloride efflux of compound **2f** measured using an ISE assay (1 mol% relative to lipid; compound added as a MeOH stock solution to achieve a final MeOH concentration of 5% v/v to ensure deliverability) at pH 5.4, 7.4, and 10.4 using liposomes prepared from (a) a 7:3 POPC/cholesterol mixture and (b) POPG. Repeats for all compounds in POPC liposomes are shown in Figure S107-Figure S118.

S7 Lipid Binding Assay

To asses lipid binding, we used our previously reported NBD-assay (NBD = 7-nitrobenzo-2-oxa-1,3-diazole).[13, 14] This assay uses NBD-labelled lipids that can be quenched by small molecules via a Stern-Volmer relationship. The amount of quenching will depend on the inherent Stern-Volmer constant of the NBD dye for the small molecule, and on the concentration of the small molecule in the membrane (which depends on partitioning coefficients and thus lipid binding). When compounds have similar inherent Stern-Volmer constants towards the NBD dye, the measured Stern-Volmer constant in liposomes can be directly compared to identify differences in partitioning. Before measuring lipid binding, we therefore first need to determine the inherent Stern-Volmer constants for the transporters against NBD fluorophores.

S7.1.1 Fluorescence Titrations with N-Propyl-7-nitro-2,1,3-benoxadiazol-4-amine

To determine the inherent quenching abilities of the transporters towards NBD dyes, we synthesized NBD–*N*-propyl, following a previously reported procedure.[15] Titrations were carried out by adding aliquots of the transporters (in MeCN) to a 1:1 buffer:acetonitrile solution containing 0.5 µM NBD–*N*-propyl under continuous stirring (buffer = 10 mM Tris, 150 mM NaCl, pH 7.4). Fluorescence emission at 530 nm (with excitation at 470 nm) was recorded on an Agilent Cary Eclipse spectrofluorometer upon each addition. A control titration using equivalent volumes of MeCN was performed under identical conditions. To determine the inherent Stern-Volmer

constants, F_0/F values (where F_0 is the fluorescence at 530 nm for the control MeCN titration and F is the fluorescence after each transporter addition) were plotted against transporter concentration, and linear fits were generated using OriginPro 2023 to give the Stern-Volmer constants as the slope. The results are summarized in **Figure S139**. The results indicate that all compounds exhibit comparable Stern-Volmer constants with NBD–*N*-propyl in solution, and therefore that the NBD-assay can be used to compare the lipid binding ability of these compounds.

(a)

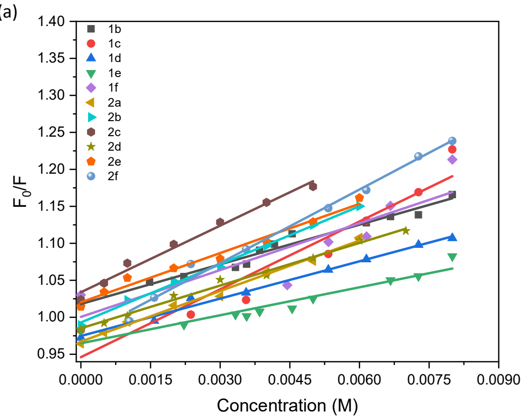

(b)

Equation	y = a + b*x										
Plot	1b	1c	1d	1e	1f	2a	2b	2c	2d	2e	2f
Weight	No Weighting										
Intercept	1.01795 ± 0.00457	0.94613 ± 0.02286	0.975 ± 0.00364	0.965 ± 0.00693	1.00 ± 0.0357	0.967 ± 0.00274	0.993 ± 0.00226	1.03 ± 0.00471	0.985 ± 0.00266	1.02 ± 0.00443	0.974 ± 0.00739
Slope	17.87258 ± 0.94179	30.57466 ± 4.26189	16.8 ± 0.721	12.6 ± 1.38	21.1 ± 6.27	22.8 ± 0.812	26.2 ± 0.627	30.0 ± 1.68	19.2 ± 0.674	22.2 ± 1.31	33.1 ± 1.46
Residual Sum of Squares	5.68243E-4	0.00443	1.78E-4	8.13E-4	0.00605	1.33E-4	5.50E-5	2.94E-4	1.59E-4	3.46E-4	6.28E-4
Pearson's r	0.9864	0.9547	0.995	0.955	0.859	0.996	0.999	0.992	0.996	0.990	0.994
R-Square (COD)	0.97298	0.91145	0.989	0.912	0.739	0.992	0.997	0.985	0.991	0.979	0.988
Adj. R-Square	0.97028	0.89374	0.987	0.901	0.673	0.991	0.997	0.982	0.990	0.976	0.987

Figure S139. Fluorescence titration of compounds **1b–2f** (compound 1a was not soluble in experimental conditions) into a solution of 0.5 µM *N*-propyl-7-nitro-2,1,3-benzoxadiazol-4-amine (NBD–*N*-propyl) in a 1:1 acetonitrile:buffer mixture. Excitation wavelength: 470 nm, emission wavelength: 530 nm. (a) Stern–Volmer plots for compounds **1b–2f**. (b) Linear fits of the Stern–Volmer plots, where the slope corresponds to the Stern–Volmer constant (K_{SV}).

S7.1.2 Fluorescence Titrations with POPG and POPC

To determine the interactions between the transporters and POPC or POPG lipids in membranes, our previously reported NBD assay was used. [13, 14] In these experiments, aliquots of the transporters in MeOH are added to an aqueous solution of 200 nm POPC or POPG large unilamellar vesicles (LUVs) containing NBD-labeled lipids (18:1-6:0 NBD-PC or 18:1-6:0 NBD-PG). To prepare the LUVs, the unlabeled lipids were weighed into a small (25 mL) round-bottom flask, and 1 mol% NBD-labeled lipid (relative to the total unlabeled lipid) was added from a 1 mg/mL chloroform stock. The lipids were dissolved in chloroform to obtain a homogeneous mixture. The solvent was subsequently removed using a rotary evaporator, and the resulting lipid film was further dried

overnight under high vacuum. The dry film was then hydrated with Tris buffer (10 mM Tris, 150 mM NaCl, pH 7.4) and vortexed until the lipid was fully suspended. The suspension was subjected to nine freeze–thaw cycles, alternating between submersion in liquid nitrogen and thawing in warm water. After allowing the suspension to rest at room temperature for 30 minutes, it was extruded 25 times through a 200 nm polycarbonate membrane (Nucleopore) using the Avanti Mini-Extruder set (Avanti Polar Lipids, Inc.). For each titration, the lipid stock solution was diluted with Tris buffer to yield 2.5 mL of a 25 µM lipid solution in a fluorescence cuvette.

Fluorescence emission spectra were recorded using an Agilent Cary Eclipse fluorescence spectrophotometer (excitation wavelength = 470 nm). Aliquots of the transporters in MeOH were added sequentially, and spectra were recorded after each addition. A small stir bar was used to ensure proper mixing. The total MeOH content did not exceed 1.5% of the total volume (except for **2f**, where up to 5% was required). A control titration with the same volume of MeOH was also performed. For the data work-up, fluorescence intensity was normalized by dividing the intensity at each wavelength by the fluorescence intensity at 530 nm from the corresponding control titration with MeOH. Where significant fluorescence quenching was observed, Stern–Volmer analysis was conducted: F_o/F values (where F_o is the fluorescence intensity at 530 nm for the control experiment with matching MeOH content, and F is the intensity at 530 nm upon transporter addition) were plotted against the transporter concentration, and a linear fit was obtained using OriginPro 2023. Each titration was independently repeated at least three times. The Stern–Volmer constants obtained from each repeat were averaged to give the final reported value. **Figure S140-Figure S163** show the fluorescence titrations performed using POPC containing 1 mol% NBD-PC (to assess PC binding) and POPG containing 1 mol% NBD-PG (to assess PG binding).

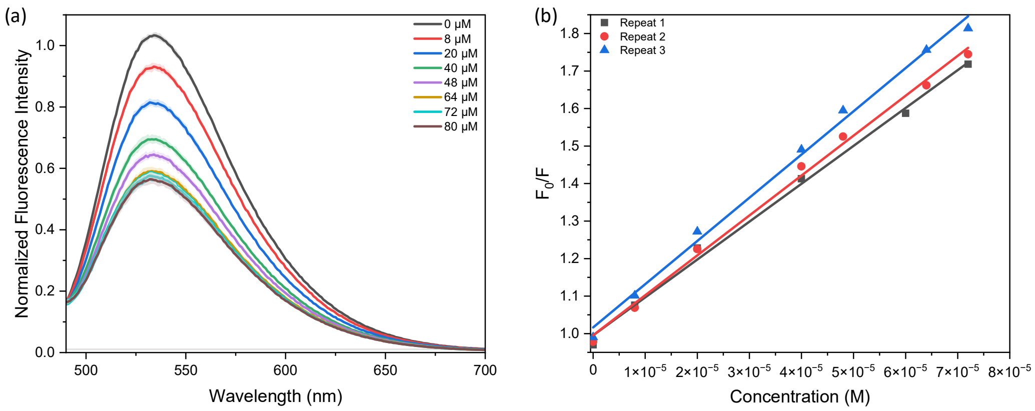

(c)

Equation	y = a + b*x		
Plot	Repeat 1	Repeat 2	Repeat 3
Weight	No Weighting		
Intercept	0.995 ± 0.0146	0.996 ± 0.0137	1.02 ± 0.0166
Slope	1.01E+4 ± 344	1.06E+4 ± 310	1.15E+4 ± 377
Residual Sum of Squares	0.00198	0.00220	0.00325
Pearson's r	0.998	0.998	0.997
R-Square (COD)	0.995	0.996	0.995
Adj. R-Square	0.994	0.995	0.994

Figure S140. Fluorescence titration of compound **1a** into a solution of 200 nm LUVs (POPC containing 1 mol% NBD-PC). Excitation wavelength = 470 nm. (a) Normalized fluorescence spectra, average of all repeats, with shaded areas indicating standard deviations. (b) Stern-Volmer plot for all individual repeats. (c) Results of the linear fit of the Stern-Volmer plots. The slope corresponds to the Stern-Volmer constant K_{SV}.

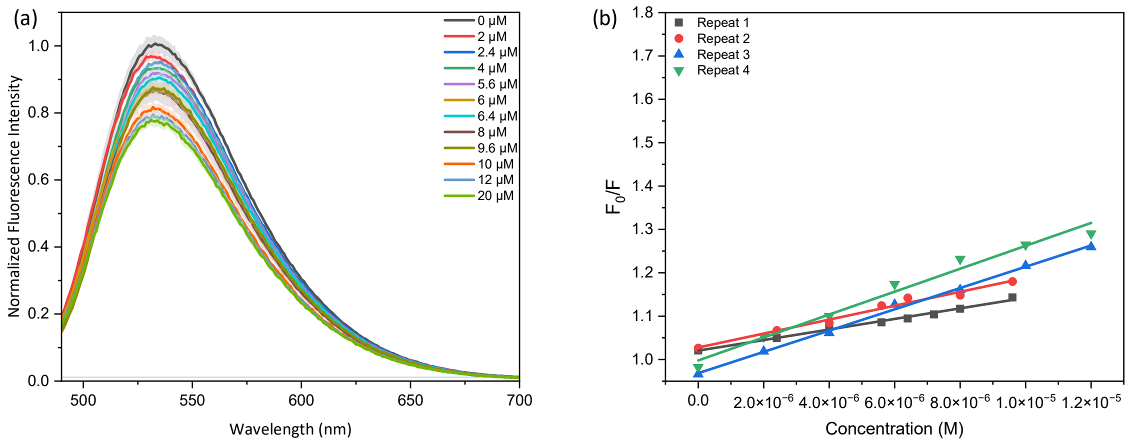

(c)

Equation	y = a + b*x			
Plot	Repeat 1	Repeat 2	Repeat 3	Repeat 4
Weight	No Weighting			
Intercept	1.02 ± 0.00320	1.03 ± 0.00579	0.968 ± 0.00424	0.998 ± 0.0125
Slope	1.22E+4 ± 521	1.60E+4 ± 968	2.45E+4 ± 588	2.64E+4 ± 1.73E+3
Residual Sum of Squares	1.12E-4	3.05E-4	1.94E-4	0.00168
Pearson's r	0.995	0.991	0.999	0.989
R-Square (COD)	0.989	0.982	0.997	0.979
Adj. R-Square	0.987	0.979	0.997	0.975

Figure S141. Fluorescence titration of compound **1a** into a solution of 200 nm LUVs (POPG containing 1 mol% NBD-PG). Excitation wavelength = 470 nm. (a) Normalized fluorescence spectra, average of all repeats, with shaded areas indicating standard deviations. (b) Stern-Volmer plots for all individual repeats. (c) Results of the linear fit of the Stern-Volmer plots. The slope corresponds to the Stern-Volmer constant K_{SV}.

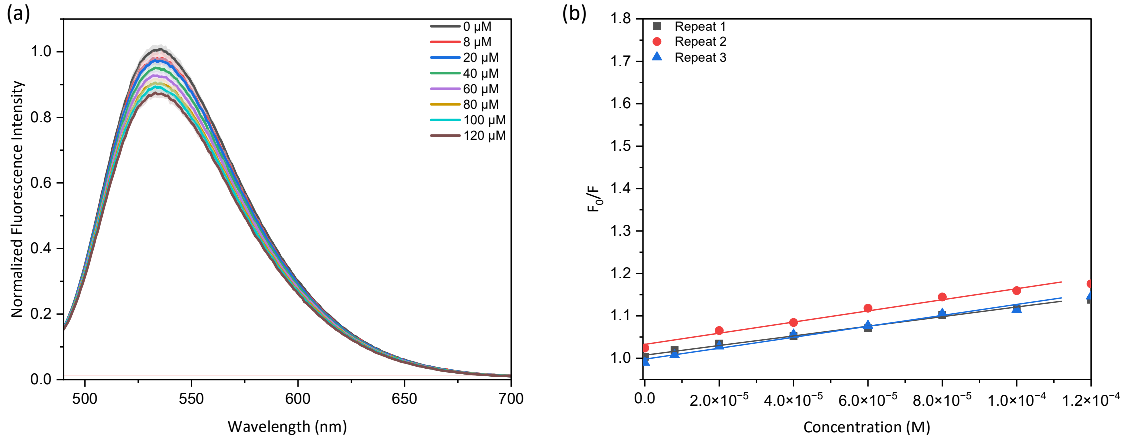

(c)

Equation	y = a + b*x		
Plot	Repeat 1	Repeat 2	Repeat 3
Weight	No Weighting		
Intercept	1.01 ± 0.00266	1.03 ± 0.00478	0.998 ± 0.00422
Slope	1.13E+3 ± 40.5	1.31E+3 ± 68.0	1.29E+3 ± 64.2
Residual Sum of Squares	1.24E-4	2.38E-4	3.11E-4
Pearson's r	0.996	0.993	0.993
R-Square (COD)	0.992	0.987	0.985
Adj. R-Square	0.991	0.984	0.983

Figure S142. Fluorescence titration of compound **1b** into a solution of 200 nm LUVs (POPC containing 1 mol% NBD-PC). Excitation wavelength = 470 nm. (a) Normalized fluorescence spectra, average of all repeats, with shaded areas indicating standard deviations. (b) Stern-Volmer plots for all individual repeats. (c) Results of the linear fit of the Stern-Volmer plots. The slope corresponds to the Stern-Volmer constant K_{SV}.

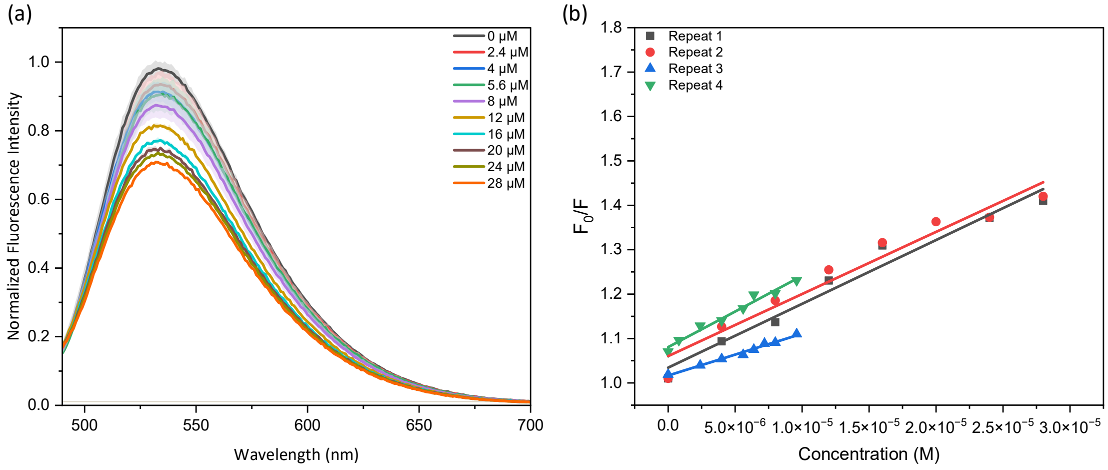

(c)

Equation	y = a + b*x			
Plot	Repeat 1	Repeat 2	Repeat 3	Repeat 4
Weight	No Weighting			
Intercept	1.03 ± 0.0185	1.06 ± 0.0214	1.02 ± 0.00265	1.08 ± 0.00579
Slope	1.44E+4 ± 1.14E+3	1.40E+4 ± 1.28E+3	9.43E+3 ± 431	1.60E+4 ± 1.03E+3
Residual Sum of Squares	0.00412	0.00659	7.68E-5	5.23E-4
Pearson's r	0.985	0.976	0.994	0.988
R-Square (COD)	0.969	0.952	0.988	0.976
Adj. R-Square	0.963	0.944	0.986	0.972

Figure S143. Fluorescence titration of compound **1b** into a solution of 200 nm LUVs (POPG containing 1 mol% NBD-PG). Excitation wavelength = 470 nm. (a) Normalized fluorescence spectra, average of all repeats, with shaded areas indicating standard deviations. (b) Stern-Volmer plots for all individual repeats. (c) Results of the linear fit of the Stern-Volmer plots. The slope corresponds to the Stern-Volmer constant K_{SV}.

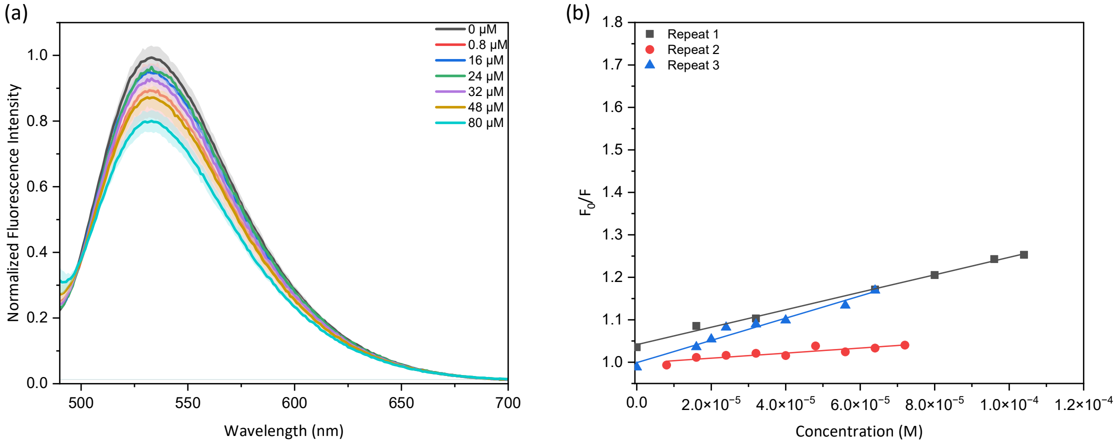

(c)

Equation	y = a + b*x		
Plot	Repeat 1	Repeat 2	Repeat 3
Weight	No Weighting		
Intercept	1.04137 ± 0.00436	0.99765 ± 0.00525	0.99903 ± 0.00754
Slope	2057.25804 ± 64.67397	596.99164 ± 116.7297	2617.32991 ± 202.44685
Residual Sum of Squares	2.06125E-4	3.66262E-4	7.74611E-4
Pearson's r	0.99754	0.88819	0.98252
R-Square (COD)	0.99508	0.78888	0.96535
Adj. R-Square	0.9941	0.75872	0.95957

Figure S144. Fluorescence titration of compound **1c** into a solution of 200 nm LUVs (POPC containing 1 mol% NBD-PC). Excitation wavelength = 470 nm. (a) Normalized fluorescence spectra, average of all repeats, shaded areas indicating standard deviations. (b) Stern-Volmer plots for all individual repeats. (c) Results of the linear fit of the Stern-Volmer plots. The slope corresponds to the Stern-Volmer constant K_{SV}.

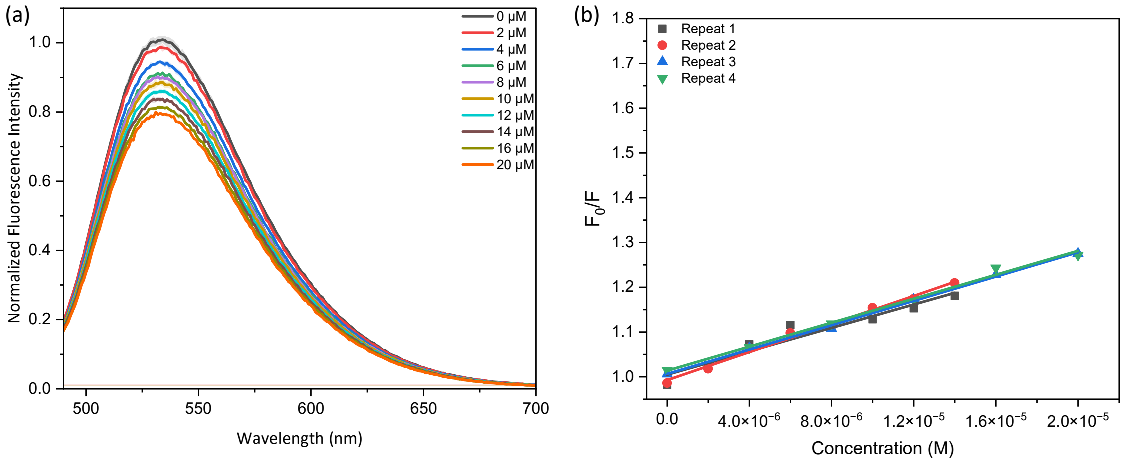

(c)

Equation	y = a + b*x			
Plot	Repeat 1	Repeat 2	Repeat 3	Repeat 4
Weight	No Weighting			
Intercept	1.00 ± 0.0118	0.993 ± 0.00513	1.01 ± 0.00355	1.01 ± 0.00669
Slope	1.30E+4 ± 1.41E+3	1.57E+4 ± 613	1.36E+4 ± 293	1.34E+4 ± 552
Residual Sum of Squares	0.00200	3.79E-4	9.60E-5	3.41E-4
Pearson's r	0.967	0.995	0.999	0.997
R-Square (COD)	0.935	0.991	0.998	0.993
Adj. R-Square	0.924	0.989	0.998	0.992

Figure S145. Fluorescence titration of compound **1c** into a solution of 200 nm LUVs (POPG containing 1 mol% NBD-PG). Excitation wavelength = 470 nm. (a) Normalized fluorescence spectra, average of all repeats, shaded areas indicating standard deviations. (b) Stern-Volmer plots for all individual repeats. (c) Results of the linear fit of the Stern-Volmer plots. The slope corresponds to the Stern-Volmer constant K_{SV}.

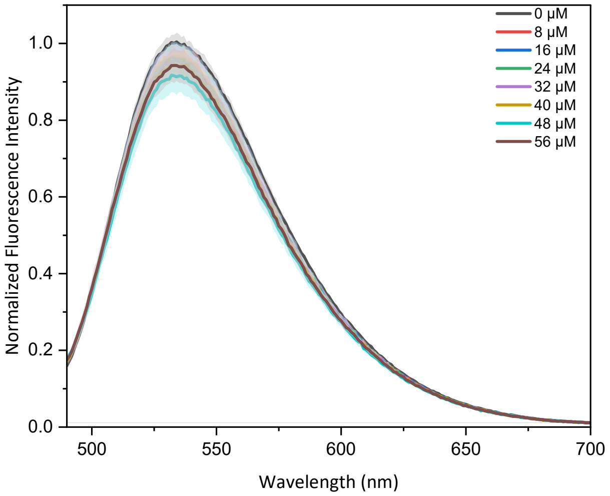

Figure S146. Fluorescence titration of compound **1d** into a solution of 200 nm LUVs (POPC containing 1 mol% NBD-PC). Excitation wavelength = 470 nm. No significant change in fluorescence intensity was observed. Results are the average of minimum 3 independent repeats and shaded areas represent standard deviations.

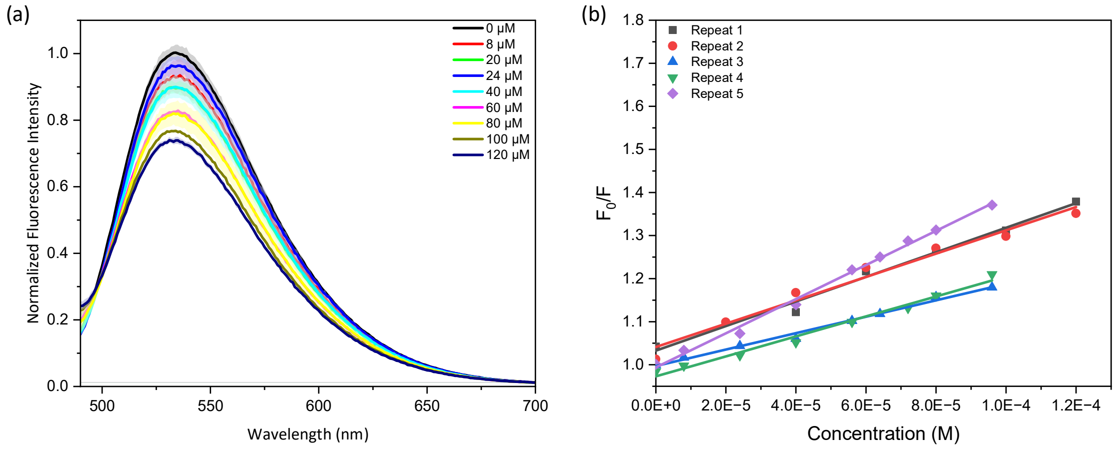

(c)

Equation	y = a + b*x				
Plot	Repeat 1	Repeat 2	Repeat 3	Repeat 4	Repeat 5
Weight	No Weighting				
Intercept	1.03 ± 0.0124	1.04 ± 0.0142	0.997 ± 0.00408	0.973 ± 0.00666	0.994 ± 0.00604
Slope	2.85E+3 ± 161	2.71E+3 ± 197	1.91E+3 ± 72.9	2.32E+3 ± 116	3.96E+3 ± 104
Residual Sum of Squares	9.63E-4	0.00217	2.60E-4	6.91E-4	6.65E-4
Pearson's r	0.994	0.987	0.996	0.993	0.998
R-Square (COD)	0.987	0.974	0.991	0.985	0.995
Adj. R-Square	0.984	0.969	0.990	0.983	0.995

Figure S147. Fluorescence titration of compound **1d** into a solution of 200 nm LUVs (POPG containing 1 mol% NBD-PG). Excitation wavelength = 470 nm. (a) Normalized fluorescence spectra, average of all repeats, shaded areas indicating standard deviations. (b) Stern-Volmer plots for all individual repeats. (c) Results of the linear fit of the Stern-Volmer plots. The slope corresponds to the Stern-Volmer constant K_{SV}.

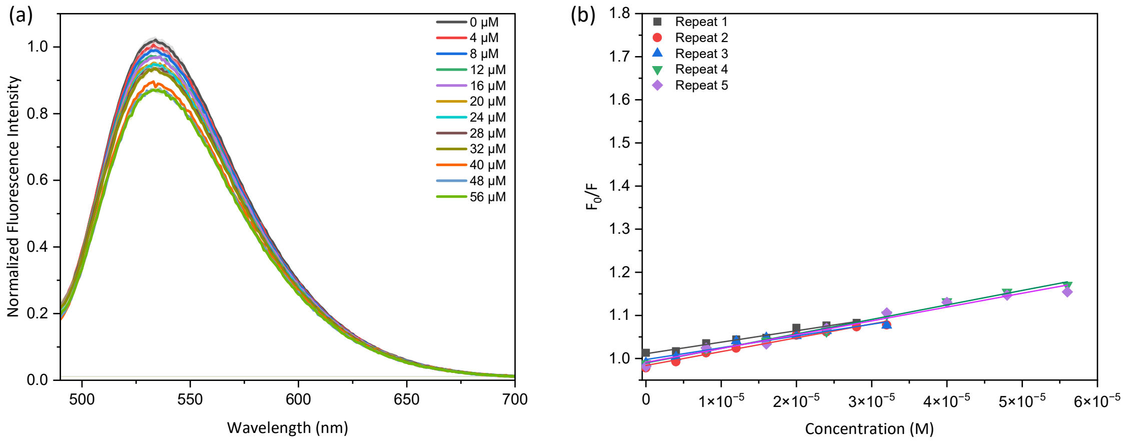

(c)

Equation	y = a + b*x				
Plot	Repeat 1	Repeat 2	Repeat 3	Repeat 4	Repeat 5
Weight	No Weighting				
Intercept	1.01 ± 0.00307	0.984 ± 0.00334	0.998 ± 0.00442	0.990 ± 0.00403	0.991 ± 0.00727
Slope	2.66E+3 ± 183	3.20E+3 ± 175	2.74E+3 ± 251	3.36E+3 ± 120	3.20E+3 ± 217
Residual Sum of Squares	1.35E-4	2.07E-4	3.02E-4	2.34E-4	7.62E-4
Pearson's r	0.986	0.990	0.976	0.996	0.986
R-Square (COD)	0.972	0.979	0.952	0.992	0.973
Adj. R-Square	0.968	0.977	0.944	0.991	0.969

Figure S148. Fluorescence titration of compound **1e** into a solution of 200 nm LUVs (POPC containing 1 mol% NBD-PC). Excitation wavelength = 470 nm. (a) Normalized fluorescence spectra, average of all repeats, with shaded areas indicating standard deviations. (b) Stern-Volmer plots for all individual repeats. (c) Results of the linear fit of the Stern-Volmer plots. The slope corresponds to the Stern-Volmer constant K_{SV}.

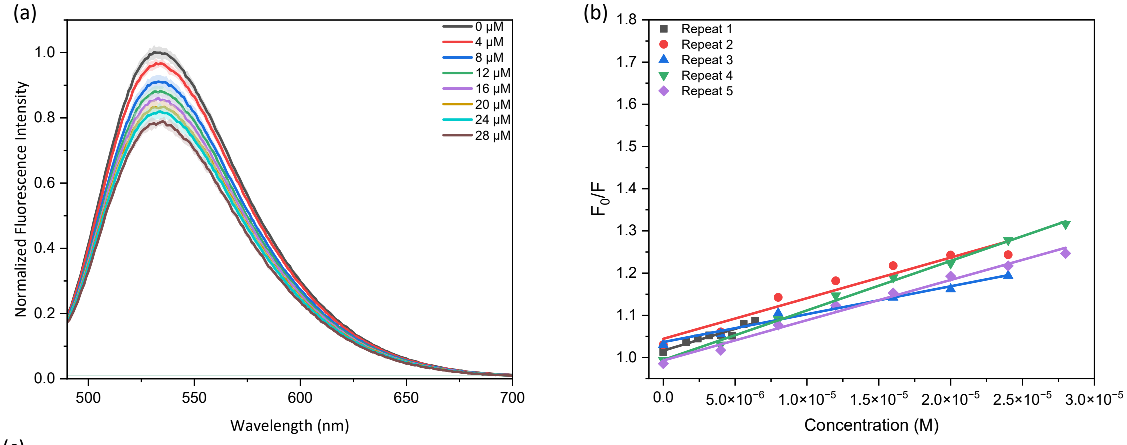

(c)

Equation	y = a + b*x				
Plot	Repeat 1	Repeat 2	Repeat 3	Repeat 4	Repeat 5
Weight	No Weighting				
Intercept	1.02 ± 0.00510	1.04 ± 0.0168	1.04 ± 0.00625	0.994 ± 0.00530	0.993 ± 0.00791
Slope	1.03E+4 ± 1.26E+3	9.63E+3 ± 1.16E+3	6.61E+3 ± 434	1.17E+4 ± 317	9.54E+3 ± 473
Residual Sum of Squares	3.06E-4	0.00302	4.21E-4	4.05E-4	9.02E-4
Pearson's r	0.958	0.965	0.989	0.998	0.993
R-Square (COD)	0.918	0.932	0.979	0.996	0.985
Adj. R-Square	0.904	0.919	0.975	0.995	0.983

Figure S149. Fluorescence titration of compound **1e** into a solution of 200 nm LUVs (POPG containing 1 mol% NBD-PG). Excitation wavelength = 470 nm. (a) Normalized fluorescence spectra, average of all repeats, with shaded areas indicating standard deviations. (b) Stern-Volmer plots for all individual repeats. (c) Results of the linear fit of the Stern-Volmer plots. The slope corresponds to the Stern-Volmer constant K_{SV}.

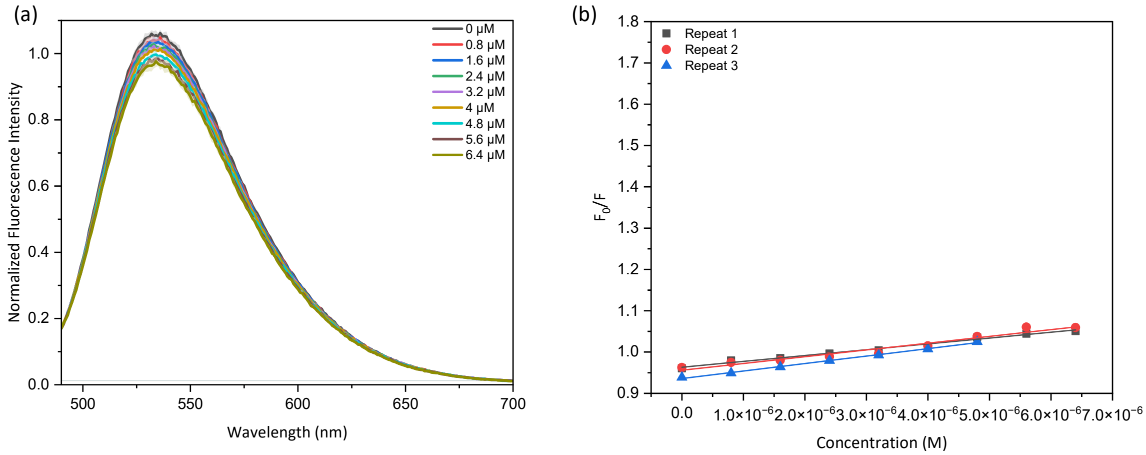

(c)

Equation	y = a + b*x		
Plot	Repeat 1	Repeat 2	Repeat 3
Weight	No Weighting		
Intercept	0.963 ± 0.00230	0.956 ± 0.00516	0.936 ± 0.00131
Slope	1.41E+4 ± 607	1.64E+4 ± 1.35E+3	1.81E+4 ± 454
Residual Sum of Squares	8.34E-5	4.93E-4	1.85E-5
Pearson's r	0.995	0.977	0.998
R-Square (COD)	0.989	0.954	0.997
Adj. R-Square	0.987	0.948	0.996

Figure S150. Fluorescence titration of compound **1f** into a solution of 200 nm LUVs (POPC containing 1 mol% NBD-PC). Excitation wavelength = 470 nm. (a) Normalized fluorescence spectra, average of all repeats, with shaded areas indicating standard deviations. (b) Stern-Volmer plots for all individual repeats. (c) Results of the linear fit of the Stern-Volmer plots. The slope corresponds to the Stern-Volmer constant K_{SV}.

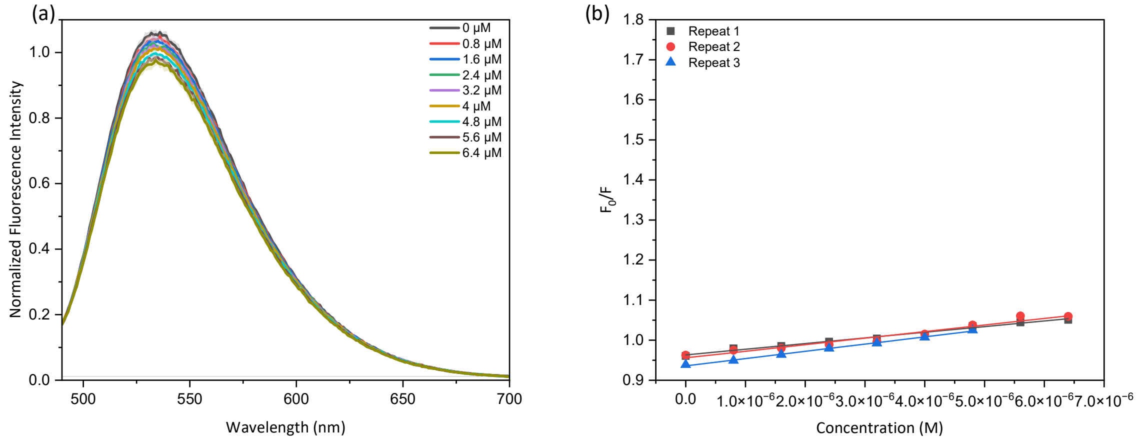

(c)

Equation	y = a + b*x		
Plot	Repeat 1	Repeat 2	Repeat 3
Weight	No Weighting		
Intercept	0.961 ± 0.0145	1.01 ± 0.0107	0.958 ± 0.0106
Slope	6.53E+4 ± 5.01E+3	5.07E+4 ± 3.72E+3	4.45E+4 ± 3.43E+3
Residual Sum of Squares	0.00225	0.00124	0.00132
Pearson's r	0.986	0.987	0.985
R-Square (COD)	0.971	0.974	0.971
Adj. R-Square	0.966	0.969	0.965

Figure S151. Fluorescence titration of compound **1f** into a solution of 200 nm LUVs (POPG containing 1 mol% NBD-PG). Excitation wavelength = 470 nm. (a) Normalized fluorescence spectra, average of all repeats, with shaded areas indicating standard deviations. (b) Stern-Volmer plots for all individual repeats. (c) Results of the linear fit of the Stern-Volmer plots. The slope corresponds to the Stern-Volmer constant K_{SV}.

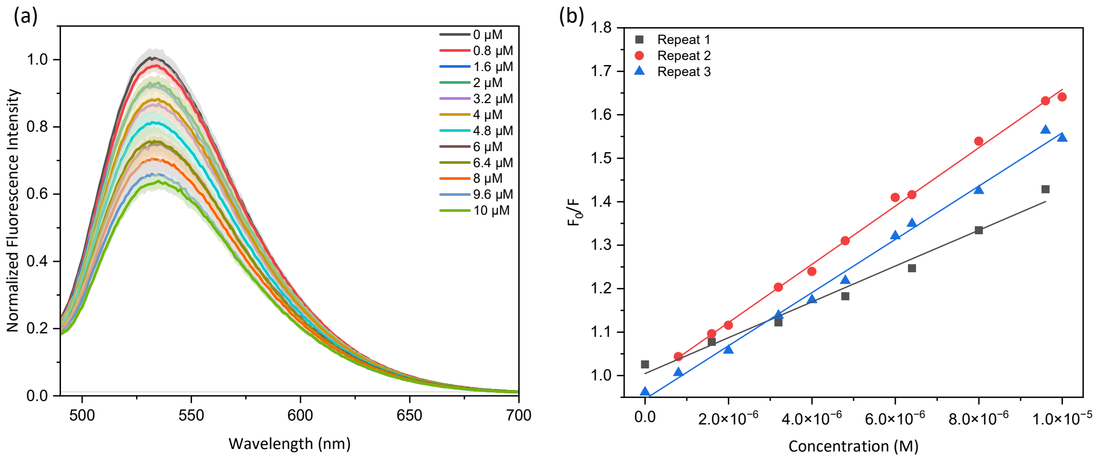

(c)

Equation	y = a + b*x		
Plot	Repeat 1	Repeat 2	Repeat 3
Weight	No Weighting		
Intercept	1.00 ± 0.0147	0.988 ± 0.00693	0.946 ± 0.00953
Slope	4.12E+4 ± 2.55E+3	6.70E+4 ± 1.17E+3	6.12E+4 ± 1.61E+3
Residual Sum of Squares	0.00234	0.00123	0.00265
Pearson's r	0.991	0.999	0.997
R-Square (COD)	0.981	0.997	0.994
Adj. R-Square	0.977	0.997	0.993

Figure S152. Fluorescence titration of compound **2a** into a solution of 200 nm LUVs (POPC containing 1 mol% NBD-PC). Excitation wavelength = 470 nm. (a) Normalized fluorescence spectra, average of all repeats, with shaded areas indicating standard deviations. (b) Stern-Volmer plots for all individual repeats. (c) Results of the linear fit of the Stern-Volmer plots. The slope corresponds to the Stern-Volmer constant K_{SV}.

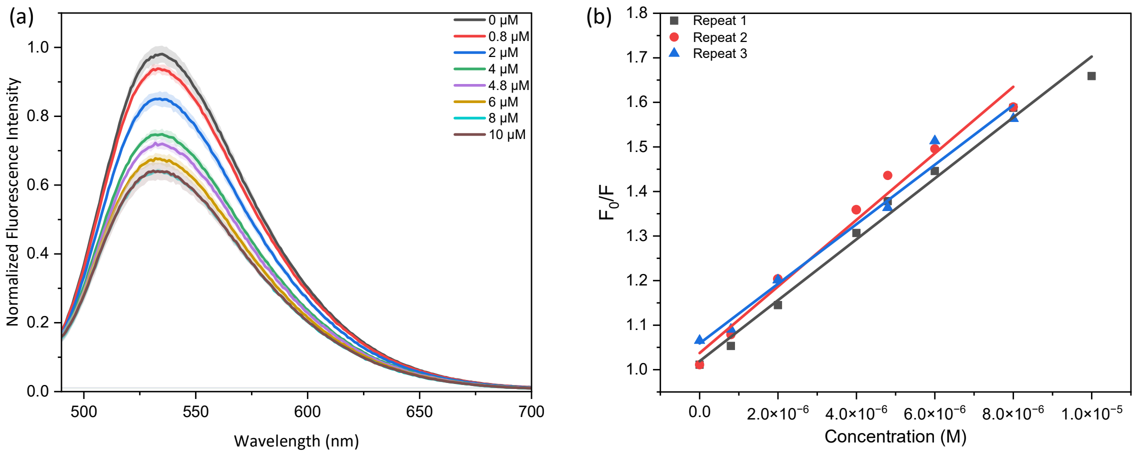

(c)

Equation	y = a + b*x		
Plot	Repeat 1	Repeat 2	Repeat 3
Weight	No Weighting		
Intercept	1.02 ± 0.0163	1.04 ± 0.0214	1.06 ± 0.0222
Slope	6.84E+4 ± 2.95E+3	7.47E+4 ± 4.73E+3	6.69E+4 ± 4.81E+3
Residual Sum of Squares	0.00444	0.00560	0.00463
Pearson's r	0.994	0.990	0.990
R-Square (COD)	0.989	0.980	0.980
Adj. R-Square	0.987	0.976	0.975

Figure S153. Fluorescence titration of compound **2a** into a solution of 200 nm LUVs (POPG containing 1 mol% NBD-PG). Excitation wavelength = 470 nm. (a) Normalized fluorescence spectra, average of all repeats, with shaded areas indicating standard deviations. (b) Stern-Volmer plots for all individual repeats. (c) Results of the linear fit of the Stern-Volmer plots. The slope corresponds to the Stern-Volmer constant K_{SV}.

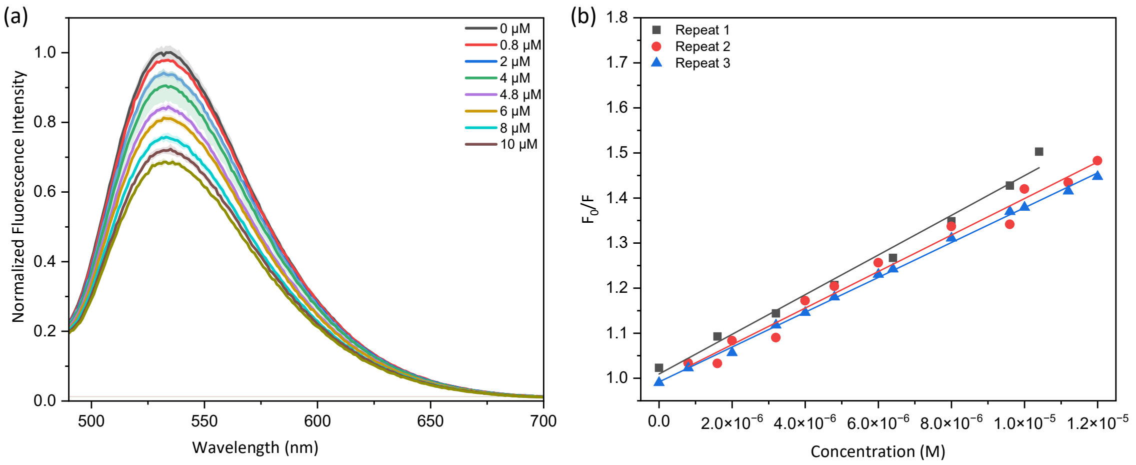

(c)

Equation	y = a + b*x		
Plot	Repeat 1	Repeat 2	Repeat 3
Weight	No Weighting		
Intercept	1.01 ± 0.0137	0.994 ± 0.0129	0.992 ± 0.00360
Slope	4.40E+4 ± 2.10E+3	4.05E+4 ± 1.79E+3	3.87E+4 ± 507
Residual Sum of Squares	0.00262	0.00551	5.33E-4
Pearson's r	0.993	0.990	0.999
R-Square (COD)	0.987	0.981	0.998
Adj. R-Square	0.984	0.979	0.998

Figure S154. Fluorescence titration of compound **2b** into a solution of 200 nm LUVs (POPC containing 1 mol% NBD-PC). Excitation wavelength = 470 nm. (a) Normalized fluorescence spectra, average of all repeats, with shaded areas indicating standard deviations. (b) Stern-Volmer plots for all individual repeats. (c) Results of the linear fit of the Stern-Volmer plots. The slope corresponds to the Stern-Volmer constant K_{SV}.

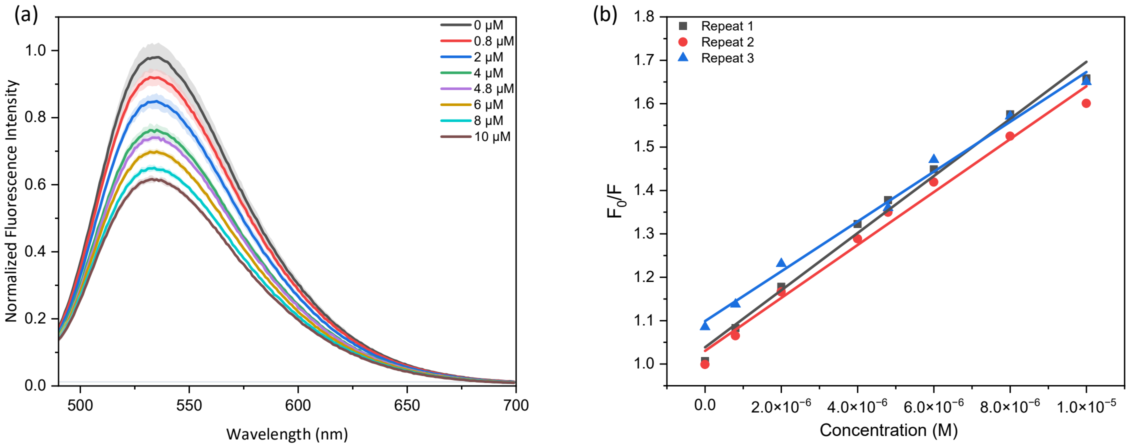

(c)

Equation	y = a + b*x		
Plot	Repeat 1	Repeat 2	Repeat 3
Weight	No Weighting		
Intercept	1.04 ± 0.0153	1.03 ± 0.0163	1.10 ± 0.0129
Slope	6.58E+4 ± 2.77E+3	6.10E+4 ± 2.95E+3	5.74E+4 ± 2.26E+3
Residual Sum of Squares	0.00393	0.00444	0.00218
Pearson's r	0.995	0.993	0.996
R-Square (COD)	0.989	0.986	0.992
Adj. R-Square	0.988	0.984	0.991

Figure S155. Fluorescence titration of compound **2b** into a solution of 200 nm LUVs (POPG containing 1 mol% NBD-PG). Excitation wavelength = 470 nm. (a) Normalized fluorescence spectra, average of all repeats, with shaded areas indicating standard deviations. (b) Stern-Volmer plots for all individual repeats. (c) Results of the linear fit of the Stern-Volmer plots. The slope corresponds to the Stern-Volmer constant K_{SV}.

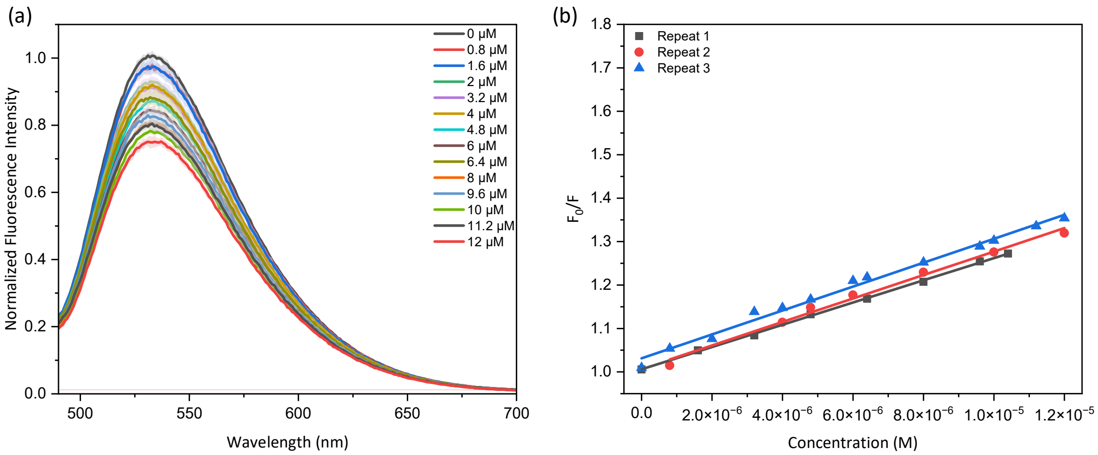

(c)

Equation	y = a + b*x		
Plot	Repeat 1	Repeat 2	Repeat 3
Weight	No Weighting		
Intercept	1.01 ± 0.00202	1.01 ± 0.00841	1.03 ± 0.00588
Slope	2.56E+4 ± 310	2.70E+4 ± 1.14E+3	2.75E+4 ± 828
Residual Sum of Squares	5.70E-5	5.60E-4	0.00142
Pearson's r	1.000	0.996	0.995
R-Square (COD)	0.999	0.991	0.990
Adj. R-Square	0.999	0.989	0.989

Figure S156. Fluorescence titration of compound **2c** into a solution of 200 nm LUVs (POPC containing 1 mol% NBD-PC). Excitation wavelength = 470 nm. (a) Normalized fluorescence spectra, average of all repeats, with shaded areas indicating standard deviations. (b) Stern-Volmer plots for all individual repeats. (c) Results of the linear fit of the Stern-Volmer plots. The slope corresponds to the Stern-Volmer constant K_{SV}.

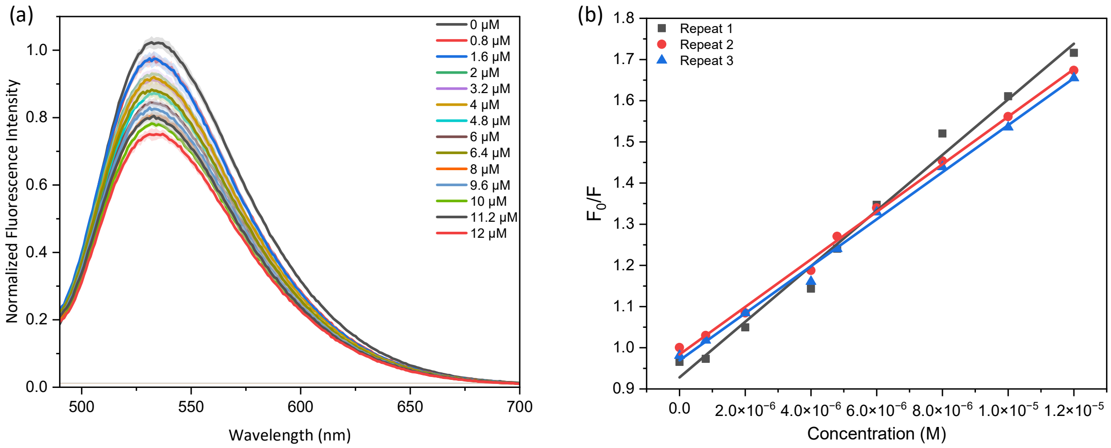

(c)

Equation	y = a + b*x		
Plot	Repeat 1	Repeat 2	Repeat 3
Weight	No Weighting		
Intercept	0.928 ± 0.0193	0.984 ± 0.00819	0.970 ± 0.00953
Slope	6.75E+4 ± 2.94E+3	5.77E+4 ± 1.25E+3	5.71E+4 ± 1.45E+3
Residual Sum of Squares	0.00825	0.00148	0.00201
Pearson's r	0.993	0.998	0.998
R-Square (COD)	0.987	0.997	0.995
Adj. R-Square	0.985	0.996	0.995

Figure S157. Fluorescence titration of compound **2c** into a solution of 200 nm LUVs (POPG containing 1 mol% NBD-PG). Excitation wavelength = 470 nm. (a) Normalized fluorescence spectra, average of all repeats, with shaded areas indicating standard deviations. (b) Stern-Volmer plots for all individual repeats. (c) Results of the linear fit of the Stern-Volmer plots. The slope corresponds to the Stern-Volmer constant K_{SV}.

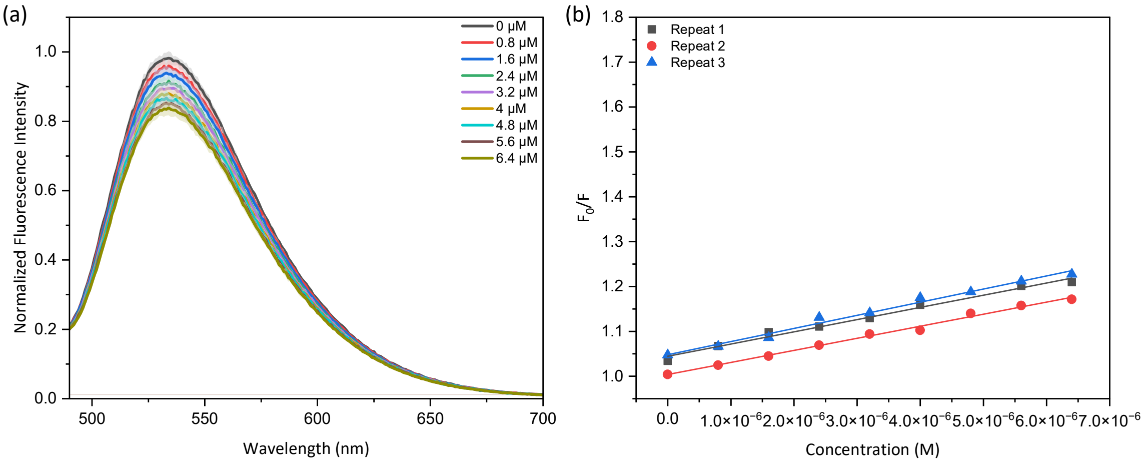

(c)

Equation	y = a + b*x		
Plot	Repeat 1	Repeat 2	Repeat 3
Weight	No Weighting		
Intercept	1.04 ± 0.00466	1.00 ± 0.00313	1.05 ± 0.00484
Slope	2.72E+4 ± 1.27E+3	2.69E+4 ± 822	2.93E+4 ± 1.27E+3
Residual Sum of Squares	3.44E-4	1.81E-4	4.34E-4
Pearson's r	0.994	0.997	0.993
R-Square (COD)	0.987	0.994	0.987
Adj. R-Square	0.985	0.993	0.985

Figure S158. Fluorescence titration of compound **2d** into a solution of 200 nm LUVs (POPC containing 1 mol% NBD-PC). Excitation wavelength = 470 nm. (a) Normalized fluorescence spectra, average of all repeats, with shaded areas indicating standard deviations. (b) Stern-Volmer plots for all individual repeats. (c) Results of the linear fit of the Stern-Volmer plots. The slope corresponds to the Stern-Volmer constant K_{SV}.

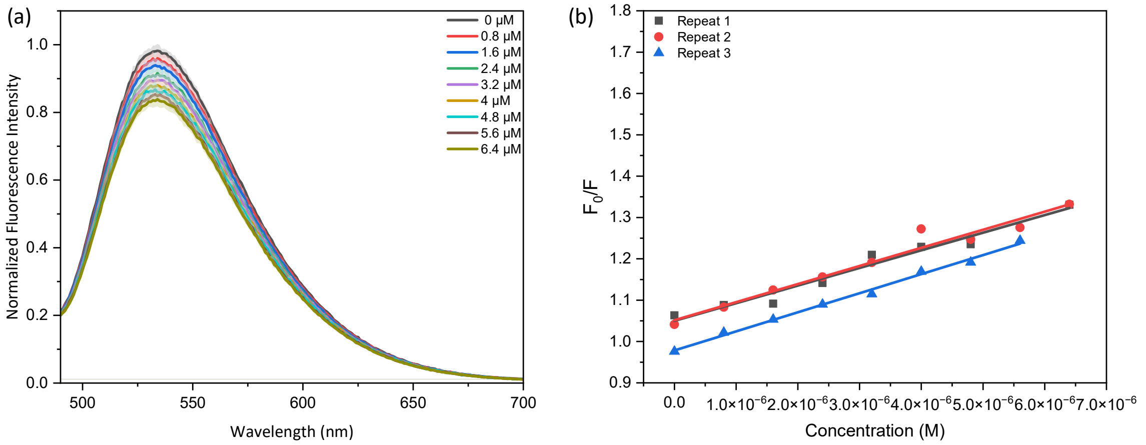

(c)

Equation	y = a + b*x		
Plot	Repeat 1	Repeat 2	Repeat 3
Weight	No Weighting		
Intercept	1.05 ± 0.0116	1.05 ± 0.0124	0.978 ± 0.00486
Slope	4.26E+4 ± 3.29E+3	4.38E+4 ± 3.27E+3	4.61E+4 ± 1.45E+3
Residual Sum of Squares	0.00208	0.00287	3.40E-4
Pearson's r	0.983	0.981	0.997
R-Square (COD)	0.965	0.962	0.994
Adj. R-Square	0.960	0.957	0.993

Figure S159. Fluorescence titration of compound **2d** into a solution of 200 nm LUVs (POPG containing 1 mol% NBD-PG). Excitation wavelength = 470 nm. (a) Normalized fluorescence spectra, average of all repeats, with shaded areas indicating standard deviations. (b) Stern-Volmer plots for all individual repeats. (c) Results of the linear fit of the Stern-Volmer plots. The slope corresponds to the Stern-Volmer constant K_{SV}.

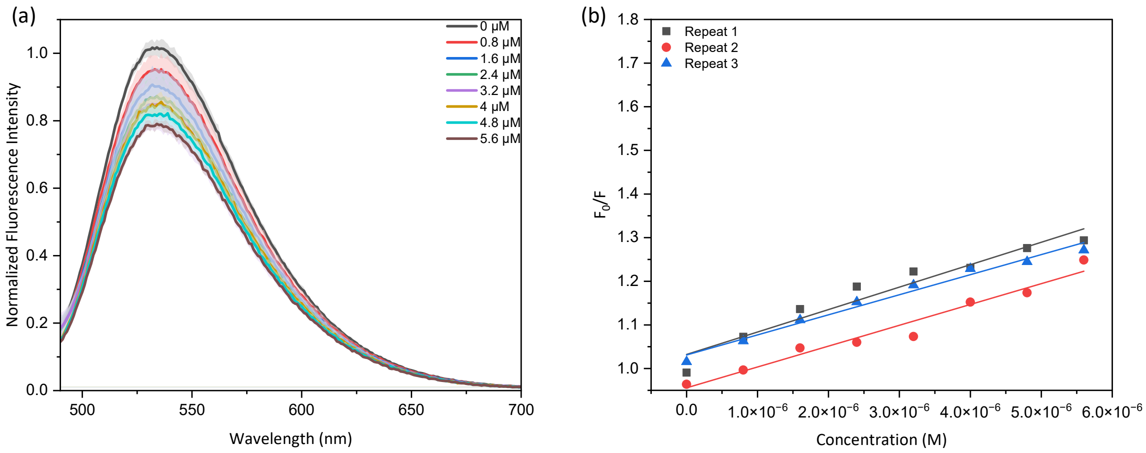

(c)

Equation	y = a + b*x		
Plot	Repeat 1	Repeat 2	Repeat 3
Weight	No Weighting		
Intercept	1.03 ± 0.0180	0.956 ± 0.0130	1.03 ± 0.00891
Slope	5.14E+4 ± 5.37E+3	4.77E+4 ± 3.89E+3	4.60E+4 ± 2.66E+3
Residual Sum of Squares	0.00465	0.00244	0.00114
Pearson's r	0.969	0.981	0.990
R-Square (COD)	0.939	0.962	0.980
Adj. R-Square	0.928	0.955	0.977

Figure S160. Fluorescence titration of compound **2e** into a solution of 200 nm LUVs (POPC containing 1 mol% NBD-PC). Excitation wavelength = 470 nm. (a) Normalized fluorescence spectra, average of all repeats, with shaded areas indicating standard deviations. (b) Stern-Volmer plots for all individual repeats. (c) Results of the linear fit of the Stern-Volmer plots. The slope corresponds to the Stern-Volmer constant K_{SV}.

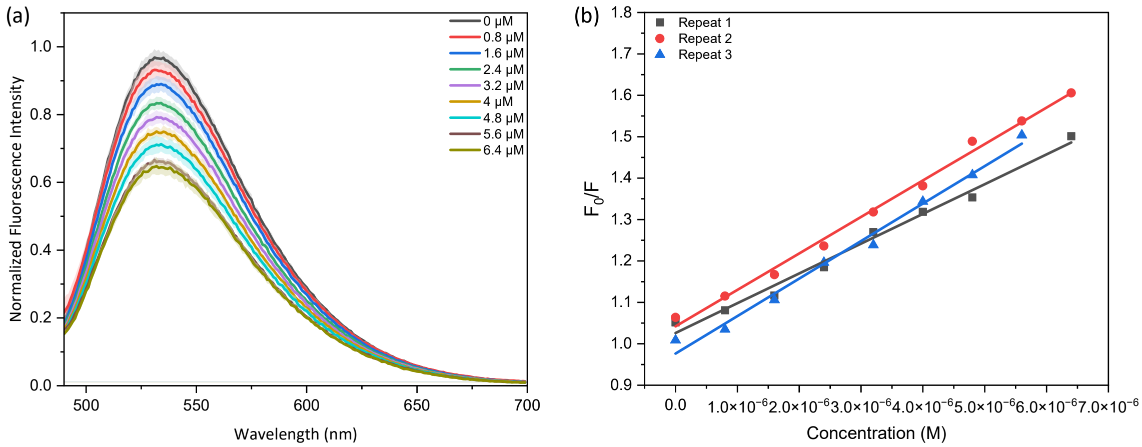

(c)

Equation	y = a + b*x		
Plot	Repeat 1	Repeat 2	Repeat 3
Weight	No Weighting		
Intercept	1.03 ± 0.0119	1.04 ± 0.00991	0.976 ± 0.0137
Slope	7.19E+4 ± 3.39E+3	8.81E+4 ± 2.60E+3	9.06E+4 ± 4.08E+3
Residual Sum of Squares	0.00220	0.00182	0.00269
Pearson's r	0.993	0.997	0.994
R-Square (COD)	0.987	0.994	0.988
Adj. R-Square	0.985	0.993	0.986

Figure S161. Fluorescence titration of compound **2e** into a solution of 200 nm LUVs (POPG containing 1 mol% NBD-PG). Excitation wavelength = 470 nm. (a) Normalized fluorescence spectra, average of all repeats, with shaded areas indicating standard deviations. (b) Stern-Volmer plots for all individual repeats. (c) Results of the linear fit of the Stern-Volmer plots. The slope corresponds to the Stern-Volmer constant K_{SV}.

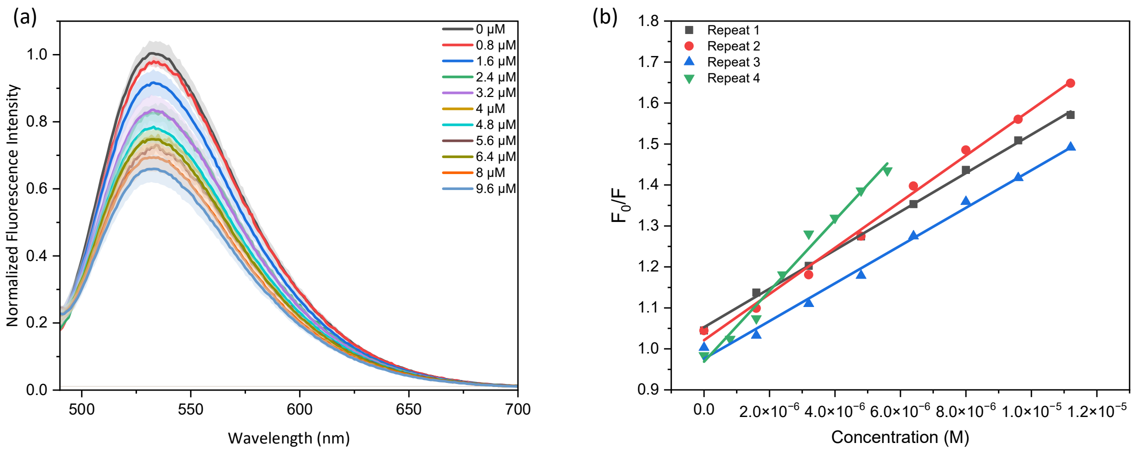

(c)

Equation	y = a + b*x			
Plot	Repeat 1	Repeat 2	Repeat 3	Repeat 4
Weight	No Weighting			
Intercept	1.05 ± 0.00473	1.02 ± 0.0113	0.976 ± 0.0111	0.969 ± 0.0147
Slope	4.70E+4 ± 706	5.63E+4 ± 1.68E+3	4.60E+4 ± 1.66E+3	8.64E+4 ± 4.40E+3
Residual Sum of Squares	3.22E-4	0.00183	0.00177	0.00312
Pearson's r	0.999	0.997	0.996	0.992
R-Square (COD)	0.999	0.995	0.992	0.985
Adj. R-Square	0.998	0.994	0.991	0.982

Figure S162. Fluorescence titration of compound **2f** into a solution of 200 nm LUVs (POPC containing 1 mol% NBD-PC). Excitation wavelength = 470 nm. (a) Normalized fluorescence spectra, average of all repeats, with shaded areas indicating standard deviations. (b) Stern-Volmer plots for all individual repeats. (c) Results of the linear fit of the Stern-Volmer plots. The slope corresponds to the Stern-Volmer constant $K_{SV.}$

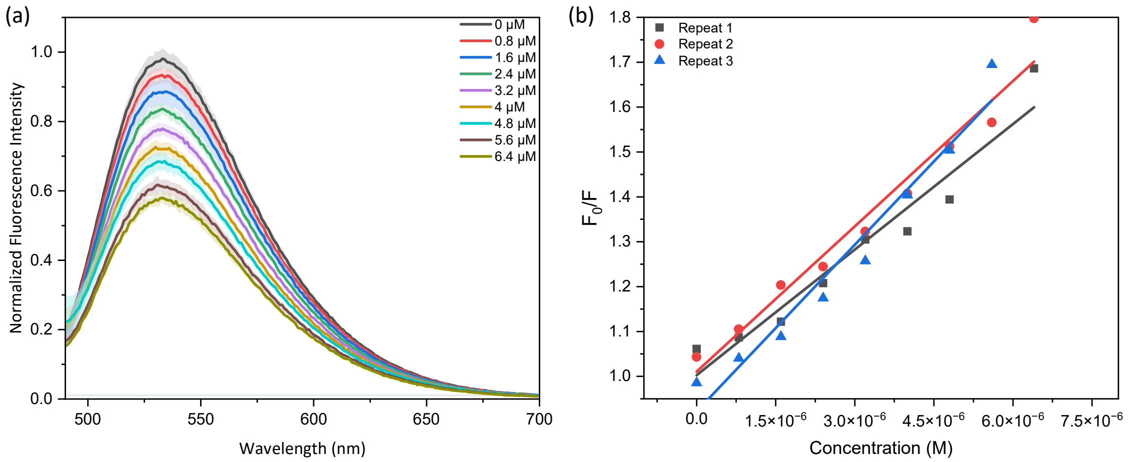

(c)

Equation	y = a + b*x		
Plot	Repeat 1	Repeat 2	Repeat 3
Weight	No Weighting		
Intercept	1.00 ± 0.0343	1.01 ± 0.0298	0.922 ± 0.0351
Slope	9.33E+4 ± 9.75E+3	1.08E+5 ± 7.83E+3	1.24E+5 ± 1.05E+4
Residual Sum of Squares	0.0182	0.0165	0.0177
Pearson's r	0.969	0.982	0.979
R-Square (COD)	0.938	0.965	0.959
Adj. R-Square	0.928	0.959	0.952

Figure S163. Fluorescence titration of compound **2f** into a solution of 200 nm LUVs (POPG containing 1 mol% NBD-PG). Excitation wavelength = 470 nm. (a) Normalized fluorescence spectra, average of all repeats, with shaded areas indicating standard deviations. (b) Stern-Volmer plots for all individual repeats. (c) Results of the linear fit of the Stern-Volmer plots. The slope corresponds to the Stern-Volmer constant $K_{SV.}$

S8 Hill plots (EC_{50} POPG)

For Hill plot analysis, ion-selective electrode (ISE) assays were performed as described in *Section S6.5* using POPG liposomes at varying concentrations of transporters **1f**, **2a**, **2b**, and **2f**, which showed significant activity at 5 mol% (*Section S6.6*). For transporter **2f**, 5% MeOH (150 µL) was included to enhance deliverability. Chloride efflux (%) values at 300 s were plotted against transporter concentration (mol%) and fitted to the Hill equation using Origin 2023

$$y = \frac{V_{max} \cdot x^n}{k^n + x^n}$$

where y is the percent chloride efflux 300 s after the addition of transporter and x is the transporter concentration (mol% with respect to lipid). V_{max}, k and n are the parameters to be fitted. V_{max} is the maximum value for y and is fixed to 100, n is the Hill coefficient, and k is the EC_{50} or inflection point of the graph. EC_{50} values are defined as the transporter concentration (mol% transporter to lipid) needed to obtain half maximum chloride efflux after 300 s. The results are shown in **Figure S164-Figure S167.**

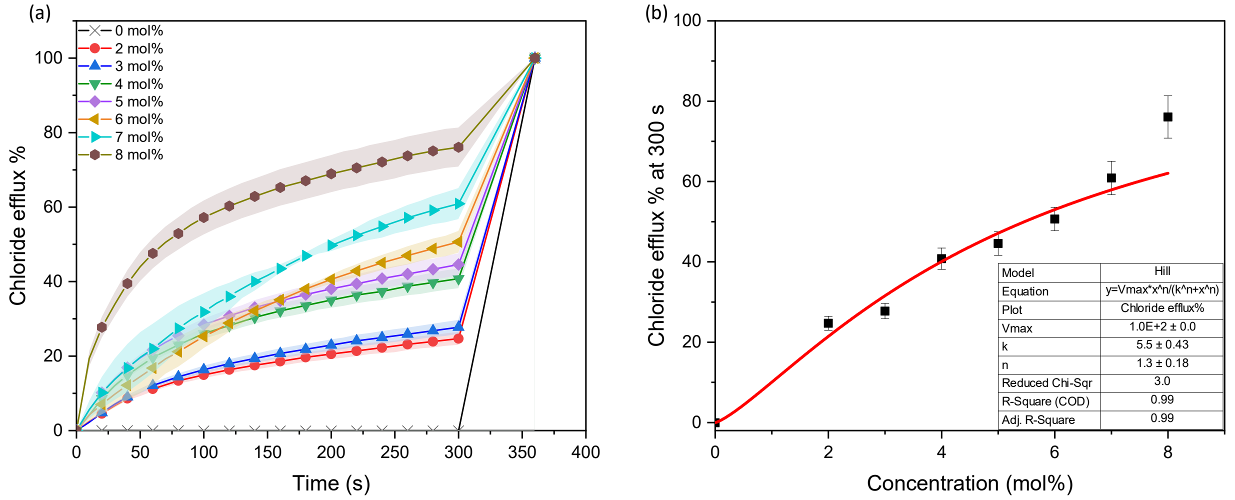

Figure S164. (a) Percentage chloride efflux mediated by transporter **1f** (various mol% transporter with respect to lipid) from 200 nm unilamellar POPG vesicles loaded with 500 mM NaCl, 10 mM HEPES at pH 7.4, and suspended in 500 mM $NaNO_3$, 10 mM HEPES at pH 7.4. The experiments were performed as described in *Section S6.5.* and is the average of the minimum of 3 repeats, whereby the shaded areas represent standard deviations. (b) Hill plots from the chloride efflux values at 300 s mediated by transporter **1f**.

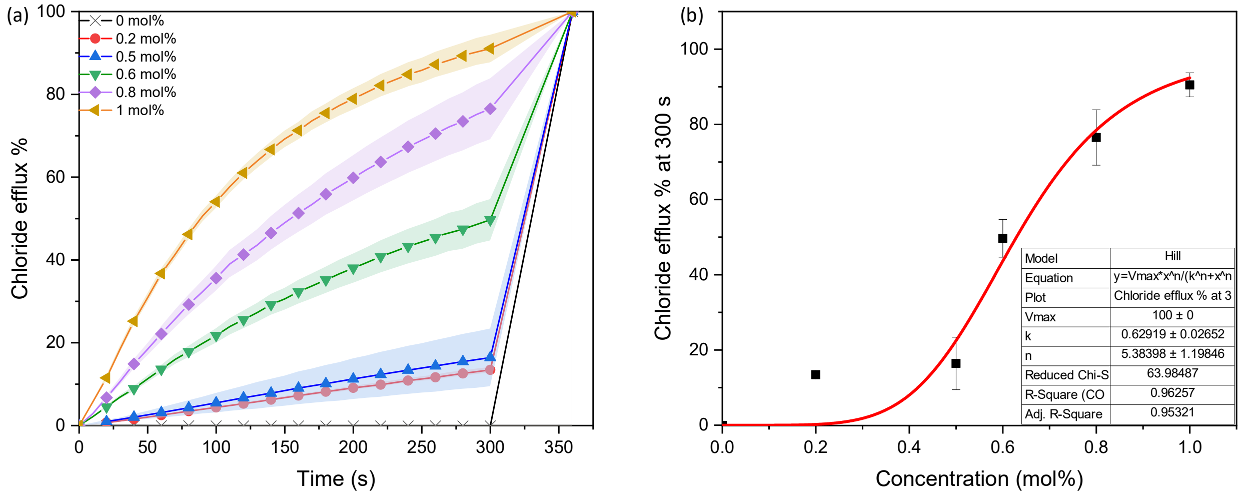

Figure S165. (a) Percentage chloride efflux mediated by transporter **2a** (various mol% transporter with respect to lipid) from 200 nm unilamellar POPG vesicles loaded with 500 mM NaCl, 10 mM HEPES at pH 7.4, and suspended in 500 mM $NaNO_3$, 10 mM HEPES at pH 7.4. The experiments were performed as described in *Section S6.5.* and is the average of the minimum of 3 repeats, whereby the shaded areas represent standard deviations. (b) Hill plots from the chloride efflux values at 300 s mediated by transporter **2a**.

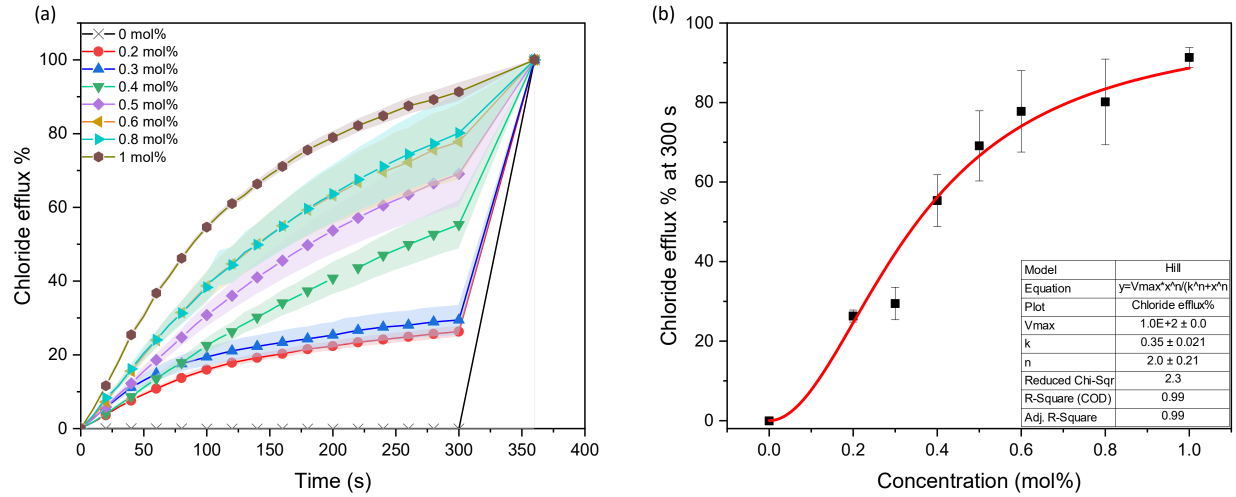

Figure S166. (a) Percentage chloride efflux mediated by transporter **2b** (various mol% transporter with respect to lipid) from 200 nm unilamellar POPG vesicles loaded with 500 mM NaCl, 10 mM HEPES at pH 7.4, and suspended in 500 mM $NaNO_3$, 10 mM HEPES at pH 7.4. The experiments were performed as described in *Section S6.5.* and is the average of the minimum of 3 repeats, whereby the shaded areas represent standard deviations. (b) Hill plots from the chloride efflux values at 300 s mediated by transporter **2b**.

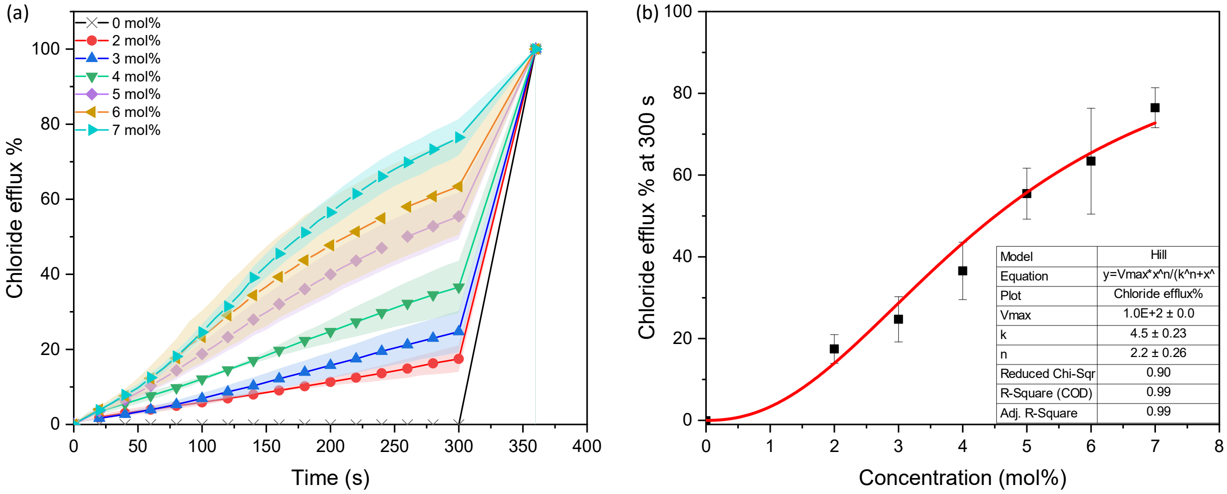

Figure S167. (a) Percentage chloride efflux mediated by transporter **2f** (various mol% transporter with respect to lipid; compound added as a MeOH stock solution to achieve a final MeOH concentration of 5% v/v to ensure deliverability) from 200 nm unilamellar POPG vesicles loaded with 500 mM NaCl, 10 mM HEPES at pH 7.4, and suspended in 500 mM $NaNO_3$, 10 mM HEPES at pH 7.4. The experiments were performed as described in *Section S6.5.* and is the average of the minimum of 3 repeats, whereby the shaded areas represent standard deviations. (b) Hill plots from the chloride efflux values at 300 s mediated by transporter **2f**.

S9 Antibacterial Activity

S9.1 Minimum Inhibitory Concentrations

The minimum inhibitory concentrations (MICs) against four Gram-positive bacterial strains (*B. subtilis*, *E. faecalis*, *S. aureus*) and two Gram-negative strains (*P. aeruginosa* and *E. coli*) were determined using the broth microdilution method recommended by the Clinical and Laboratory Standards Institute.[16] All the bacterial strains were obtained from the American Type Culture Collection. (*B. subtilis* - ATCC6051, *S. aureus* - ATCC 25923, *E. faecalis* - ATCC 29212, *P. aeruginosa* - ATCC 27853, *E. coli* - ATCC 25922) and stored in glycerol stocks at -80°C. For each experiment, a small amount of the glycerol stock was streaked onto a Müller-Hinton agar plate (Sigma-Aldrich #70191) and the agar plate was incubated for 24 hours at 35 °C. The obtained colonies were aseptically transferred into sterile cation-adjusted Müller-Hinton broth (Sigma-Aldrich #90922) and vortexed briefly. Colonies were added until the inoculum solution achieved an OD_{600} value corresponding to 1 x 10^8 CFU/mL. OD_{600} values were determined using a Biowave CO8000 Cell Density meter and 17x100 mm polystyrene culture tubes (VWR #60818-703). The inoculum was subsequently diluted to 5 x 10^5 CFU/mL in sterile cation-adjusted Müller-Hinton broth. 192 µL of this inoculum was transferred to the wells of a sterile flat-bottom polystyrene non-tissue culture treated 96-well plate (Falcon #351172) and 8 µL of a DMSO stock solution of compound was

added to achieve the desired final concentration. The 96-well plate was covered with a Breathe-Easy sealing membrane (Sigma-Aldrich # Z380059) and incubated at 35 °C for 24 hours in a VWR 1585 Incubator (no shaking). The MIC value was defined as the minimum concentration of compound that resulted in complete inhibition of bacterial growth over the full 24 hours, and was determined by visual inspection of the 96-well plates (clear wells indicate no bacterial growth). The experiment was conducted over 3 independent x 2 technical repeats and the results are given in **Table S9** as the range of obtained MIC obtained over all experiments. Clindamycin was used for quality control reasons, and counting plates were prepared each time to ensure that the initial bacteria solution was indeed 5 x 10^5 CFU/mL.

Table S9. MIC (µM) of the various compounds against a range of Gram-positive and Gram-negative bacteria

Compound	*B. subtilis*	*S. aureus*	*E. faecalis*	*E. coli*	*P. aeruginosa*
**1a**	>128	>128	>128	>128	>128
**1b**	>128	>128	>128	>128	>128
**1c**	>128	>128	>128	>128	>128
**1d**	>128	>128	>128	>128	>128
**1e**	>128	>128	>128	>128	>128
**1f**	16-32	32	16	>128	>128
**2a**	1-2	0.25-0.5	0.5-1	>128	>128
**2b**	1-2	1-2	1-2	>128	>128
**2c**	64-128	32-64	32	>128	>128
**2d**	>128	>128	>128	>128	>128
**2e**	-[a]	-[a]	-[a]	-[a]	-[a]
**2f**	-[a]	-[a]	-[a]	-[a]	-[a]

[a] MIC could not be determined because the compounds precipitated in the broth.

S9.2 Membrane Depolarization

A membrane depolarization assay was conducted using the fluorescent probe DiSC$_3$(5) (3,3′-dipropylthiadicarbocyanine iodide), following a previously reported procedure.[17] For each experiment, *Bacillus subtilis* from a glycerol stock was streaked onto Müller Hinton agar and incubated at 35 °C for 18–24 hours. Individual colonies were then transferred aseptically into sterile cation-adjusted Müller–Hinton broth and diluted to an initial optical density of OD_{600} = 0.2. The cultures were grown at 35 °C until reaching mid-logarithmic phase (typically OD_{600} ~ 0.6), followed by centrifugation at 3000 rpm for 5 minutes. The resulting pellets were resuspended and diluted in Müller–Hinton broth containing 0.5 mg/mL bovine serum albumin (BSA) to achieve an OD_{600} of 0.2. Subsequently, 172 µL of the bacterial suspension was transferred into each well of a sterile, black, flat-bottom 96-well fluorescence microplate (Brand, Cat. #7816668). Fluorescence was recorded for 3 minutes to establish baseline readings. After baseline collection, 8 µL of DiSC$_3$(5) dissolved in DMSO was added to each well to yield a final concentration of 1 µM dye and 4% DMSO.

Fluorescence measurements were then continued for an additional 10 minutes. Following this step, 20 µL of compound stock solutions or control antibiotics (prepared as 10× stocks in Müller–Hinton broth containing 4% DMSO) were added to each well, and fluorescence changes were monitored for 1 hour. All compounds were tested at approximately 2x MIC concentration.

All fluorescence measurements were performed using a BioTek Cytation 5 Cell Imaging Multi-Mode Reader under the following conditions: 35 °C, excitation wavelength of 610 nm, emission wavelength of 660 nm, readings at 23-second intervals, and orbital shaking for 5 seconds at 548 rpm (2 mm amplitude) before each measurement. Gramicidin (2 µM), a known membrane-disrupting agent, served as the positive control[18], while the ribosome-targeting antibiotic clindamycin (4 µg/mL) and untreated cells were used as negative controls. The results are shown in **Figure S168.** Compounds **2a**, **2b**, **2c** and **1f** exhibited strong depolarization effects, displaying fluorescence responses comparable to that of the positive control, gramicidin.

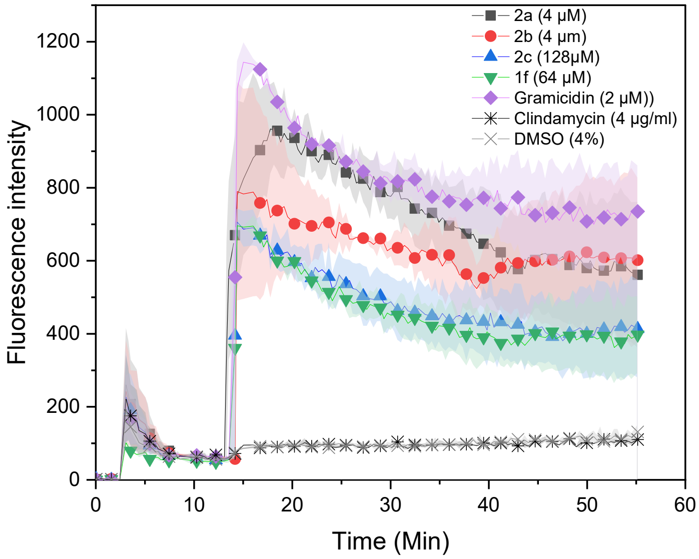

Figure S168. Membrane depolarization of *B. subtilis* induced by compounds **2a**, **2b**, **2c**, and **2d**. Fluorescence intensity of Disc$_3$(5) in *B. subtilis* upon the addition of DMSO (4%, blank), clindamycin (4 µg/mL, negative control), gramicidin (2 µM, positive control), **2a** (4 µM), **2b** (4 µM), **2c** (128 µM), and **1f** (64 µM). Disc$_3$(5) was added at t = 3 min and antibiotics were added at t = 13 min. Results are the average of 2 technical x 2 biological repeats, and shaded areas represent standard deviations.

S9.3 Sytox Green Influx

To evaluate whether the active compounds **1f**, **2a**, **2b**, and **2c** induce the formation of large pores or otherwise disrupt bacterial membrane integrity, a fluorescence-based assay using Sytox Green was performed.[19] For each experiment, *Bacillus subtilis* from a glycerol stock was streaked onto Müller–Hinton agar and incubated at 35 °C for 18–24 hours. Individual colonies were then aseptically inoculated into sterile cation-adjusted Müller–Hinton broth and diluted to an initial OD_{600}

of 0.2. The cultures were grown at 35 °C until they reached mid-logarithmic phase (typically OD_{600} ≈ 0.6), centrifuged at 3000 rpm for 5 minutes, washed twice with phosphate-buffered saline (PBS), and finally resuspended in PBS to an OD_{600} of 0.2. Sytox Green, prepared in DMSO, was added to the bacterial suspension to achieve a final concentration of 1 µM dye and 4% DMSO. The mixture was incubated at 35 °C for 15 minutes to allow dye equilibration. Subsequently, 180 µL of the stained bacterial suspension was transferred into each well of a sterile, black, flat-bottom 96-well fluorescence microplate (Brand, Cat. #7816668), and fluorescence was monitored for 4 minutes to establish a baseline. Afterward, 20 µL of compound stock solutions or control antibiotics (prepared at 10× the desired concentration in PBS containing 4% DMSO) were added, and fluorescence changes were recorded over a 2-hour period. All compounds were tested at approximately 2xMIC concentration. At the end of the experiment (130 min) 20 µL Triton X-100 (10%) was added to rupture the bacteria and establish a reference for maximal Sytox Green fluorescence.

All fluorescence readings were obtained using a BioTek Cytation 5 Cell Imaging Multi-Mode Reader under the following conditions: 35 °C, excitation wavelength 485 nm, emission wavelength 520 nm, data acquisition every 23 seconds, with 5 seconds of orbital shaking at 548 rpm (2 mm amplitude) before each read. Nisin (5 µM), a peptide known to form large membrane pores permitting Sytox Green entry, was employed as a positive control.[17, 20, 21] The ribosome-targeting antibiotic clindamycin (4 µg/mL), gramicidin (2 µM),[20,21] and untreated cells served as negative controls. The experimental outcomes are presented in **Figure S169.**

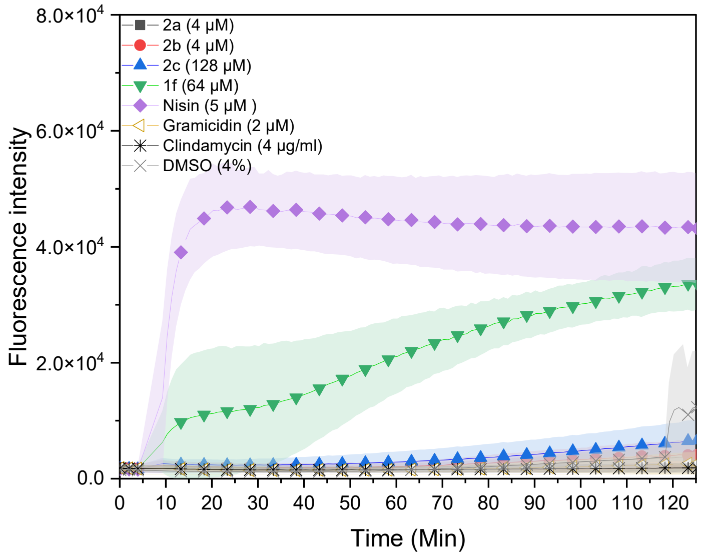

Figure S169. Fluorescence intensity of Sytox Green in *B. subtilis* in the presence of DMSO (4%, blank), clindamycin (4 µg/mL, negative control), gramicidin (2 µM, negative control), nisin (5 µM, positive control), and transporters at 2xMIC; **2a** (4 µM), **2b** (4 µM), **2c** (128µM), and **1f** (64 µM). Antibiotics were added at *t* = 4 min, and Triton X-100 was added at *t* = 130 min. Results are the average of 2 technical x 2 biological repeats, and shaded areas represent standard deviations.

S9.4 Disc$_3$(5) and Sytox Green Microscopy

To further characterize the effects of the compounds on the membrane, the membrane potential-sensitive dye Disc$_3$(5) was combined with Sytox Green for fluorescence microscopy. The assay was performed according to the procedure described in Popp *et al.*[22] *B. subtilis* was grown as described above to the mid-logarithmic phase (typically OD_{600} ≈ 0.6), centrifuged at 3000 rpm for 5 minutes, washed twice with sterile cation-adjusted Müller–Hinton broth, and the OD_{600} was adjusted to 0.2. Cells were supplemented with BSA (0.5 mg/mL). Subsequently, 200 µL of the cell suspension was transferred to a 2 mL Eppendorf tube and mixed with Disc$_3$(5) (1 µM final concentration) and Sytox Green (50 nM final concentration). The tube was incubated in an Eppendorf Thermomixer with the lid open to allow sufficient aeration at 37 °C and 1,000 rpm for 5 minutes. Antibiotics were then added, and incubation continued for an additional 10 minutes. After treatment, 2 µL of the cell suspension was transferred onto an agarose pad (1% UltraPure Agarose, Invitrogen). Fluorescence images were acquired using a BioTek Cytation 5 Cell Imaging Multi-Mode Reader equipped with the Texas Red filter set for Disc$_3$(5) and the GFP filter set for Sytox Green. Identical exposure settings were used for all samples (LED intensity = 3, shutter speed = 824 ms, camera gain = 30). Image overlays were generated using the Gen5 software accompanying the BioTek Cytation 5 system. The cellular images are shown in the main article. Healthy, polarized cells show red fluorescence in the Disc$_3$(5) channel, and no fluorescence on the Sytox Green channel (e.g., DMSO). Nisin forms large pores and therefore both depolarizes the cells and causes influx of Sytox Green (no fluorescence in the Disc$_3$(5) channel and strong fluorescence on the Sytox Green channel). Gramicidin, on the other hand, can depolarize the cells but does not create large enough pores for Sytox Green influx (no fluorescence observed in the Disc$_3$(5) or the Sytox Green channel). Compounds **2a**, **2b,** and **2c** behave similarly to gramicidin, causing depolarization but no membrane lysis. Compound **1f**, on the other hand, behaved more like nisin and also showed Sytox Green influx, suggesting its antibacterial activity might be more detergent-like in its mechanism (or form large pores).

S9.5 MQAE Chloride Influx Assay

The MQAE chloride influx assay was conducted using a modification of a previously published method.[23] For each experiment, a small amount of *B. subtilis* glycerol stock was streaked onto a Müller-Hinton agar plate and incubated for 24 hours at 35 °C. Single colonies were then aseptically transferred into sterile cation-adjusted Müller-Hinton broth and diluted to an OD_{600} of 0.2. The bacterial culture was incubated at 35 °C until reaching mid-logarithmic phase (OD_{600} ≈ 0.6). Cells were then centrifuged at 3000 rpm for 5 minutes, washed twice with phosphate-buffered saline (PBS), and resuspended in PBS to an OD_{600} of 0.2. The suspension was then treated with *N*-ethoxycarbonylmethyl-6-methoxyquinolinium bromide (MQAE) to a final concentration of 10 mM. The bacteria containing the dye were incubated at 35 °C for 40–45 minutes (incubation time

should not exceed 45 minutes to avoid reduced bacterial viability). Following incubation, cultures were centrifuged again at 3000 rpm for 5 minutes and washed twice with PBS to remove excess dye. The final cell suspension was adjusted to an OD_{600} of 0.2 in PBS. Subsequently, 180 µL of the bacterial suspension was transferred into the wells of a sterile, black, flat-bottom 96-well microplate (Brand, Cat. No. 7816668) for fluorescence measurements. At this stage, 20 µL of stock antibiotic solutions (prepared at 10× the desired final concentration in PBS containing 1% DMSO) were added, and fluorescence intensity was recorded 10 min after mixing the transporters using a BioTek Cytation 5 Cell Imaging Multi-Mode Reader (λ_{exc} = 350 nm, λ_{em} = 460 nm). The results are shown in the main article.

S10 Calcein Leakage Assay

The Sytox Green assay results described in *Section S9.3.* indicated membrane disruption by compound **1f**, and a calcein leakage assay was conducted to determine the concentration at which such disruption occurs. The leakage assay was performed at three different concentrations: 5 mol%, 100 mol%, and 200 mol% with respect to lipid. The first concentration (5 mol%) corresponds to the concentration used for most anion transport experiments, while the higher concentrations were chosen to better represent the high concentrations that correspond to the relatively high MIC value of **1f** against Gram-positive bacteria.

A thin lipid film of POPC was prepared by evaporating a chloroform solution of the lipid under reduced pressure, followed by drying under high vacuum for at least 8 hours. The resulting film was hydrated with buffer containing 70 mM calcein, 225 mM $NaNO_3$, and 10 mM HEPES (pH 7.4) and vortexed thoroughly. The lipid suspension was subjected to nine freeze–thaw cycles, alternating between immersion in liquid nitrogen and thawing in a warm water bath (<34 °C). The suspension was then equilibrated at room temperature for 30 min and extruded 25 times through a 200 nm polycarbonate membrane using an Avanti Mini Extruder (Avanti Polar Lipids, Inc.). Unencapsulated calcein was removed by size-exclusion chromatography using a Sephadex G-25 column equilibrated with 225 mM $NaNO_3$ and 10 mM HEPES buffer (pH 7.4). The final lipid concentration of the vesicle suspension was adjusted to 0.5 mM. The dye-loaded liposomes (0.5 mM lipid) were transferred into a 3 mL quartz cuvette equipped with a small magnetic stir bar and placed in the sample compartment of an Agilent Cary Eclipse fluorescence spectrometer equipped with temperature control. At t = 0 s, the fluorescence kinetic run was initiated. At t = 40 s, 120 µL of the compound solution in MeOH was added, using three different stock concentrations to achieve 5 mol%, 100 mol%, or 200 mol%. The fluorescence emission of encapsulated calcein (λ_{ex} = 490 nm, λ_{em} = 520 nm) was monitored for 5 min. At t = 5 min, 75 µL of 10% Triton X-100 was added to lyse the vesicles completely. Control experiments were performed using MeOH only. The percentage of calcein leakage was calculated using the following equation, where F_t is

the fluorescence intensity at time t, F_0 is the initial fluorescence intensity, and F_{final} is the intensity after detergent addition:

$$\text{Calcein leakage (\%)} = \frac{F_t - F_0}{F_{final} - F_0} \times 100$$

As shown in **Figure S172**, Significant membrane disruption was only observed at higher transporter concentrations (approximately 200 mol%), indicating that at concentrations relevant to chloride transport, the compounds do not cause substantial membrane leakage, but high concentrations do cause membrane disruption.

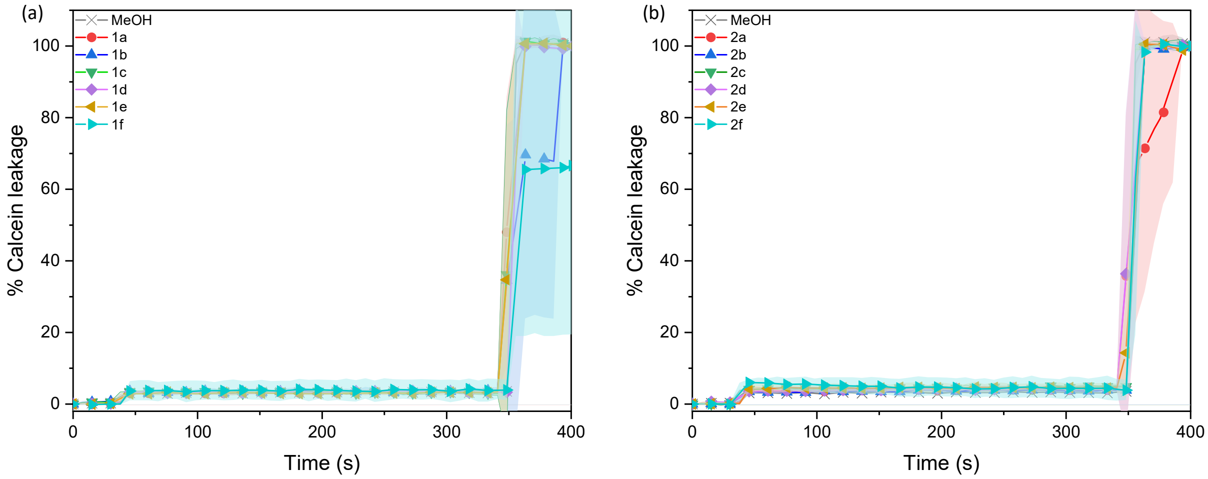

Figure S170. Calcein efflux promoted by (a) non-fluorinated transporters **1a**-**1f** (5 mol% to lipid) and (b) fluorinated transporters **2a**-**2f** (5 mol% to lipid). The experiment was performed as described in the *Section S10*. and is the average of the minimum of 3 repeats (shaded areas represent standard deviations).

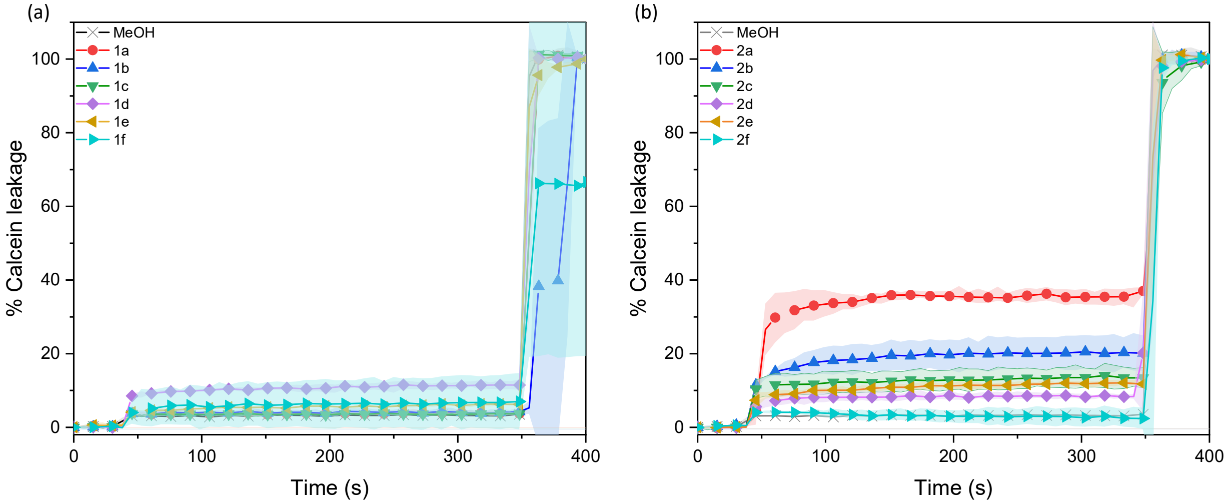

Figure S171. Calcein efflux promoted by non-fluorinated transporters (a) non-fluorinated transporters **1a**-**1f** (100 mol% to lipid) and (b) fluorinated transporters **2a**-**2f** (100 mol% to lipid). The experiment was performed as described in *Section S10*. and is the average of the minimum of 3 repeats (shaded areas represent standard deviations).

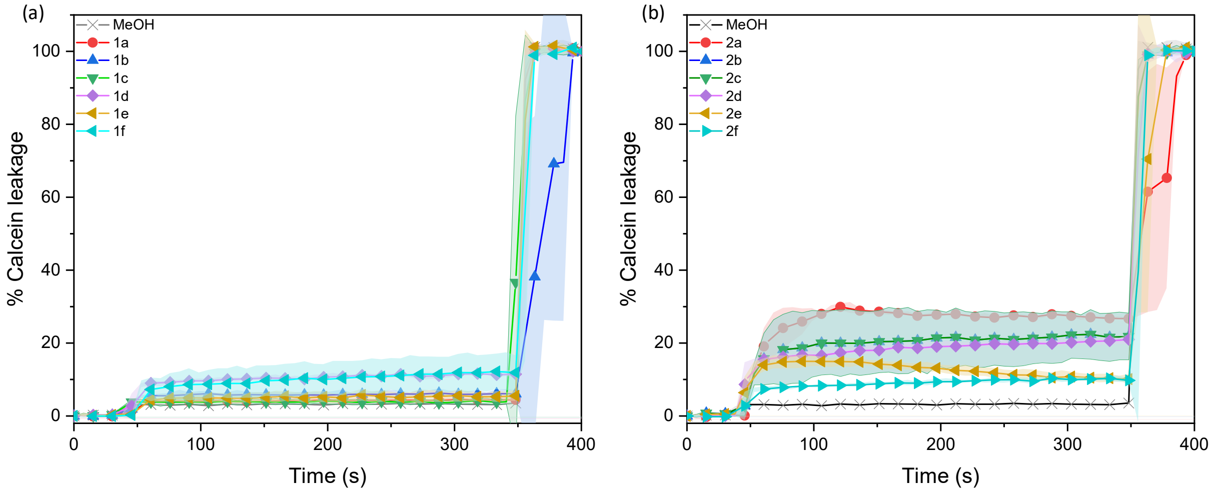

Figure S172. Calcein efflux promoted by non-fluorinated transporters (a) non-fluorinated transporters **1a**-**1f** (200 mol% to lipid) and (b) fluorinated transporters **2a**-**2f** (200 mol% to lipid). The experiment was performed as described in the section S10. and is the average of the minimum of 3 repeats (shaded areas represent standard deviations).

S11 Hemolytic activity

The following procedure was modified from a previously established method used for testing antimicrobial peptides.[24] Human red blood cells obtained from a single donor were washed twice with and resuspended in 1× phosphate-buffered saline (PBS) to achieve a final concentration of 2 × 10^7 cells/mL. The assay was conducted in a 96-well plate with a total volume of 200 µL per well, containing various concentrations of the test compounds. Specifically, 8 µL of each compound dissolved in DMSO was added to 192 µL of the RBC suspension and mixed thoroughly by pipetting, resulting in a final DMSO concentration of 4%. Triton X-100 (1%) served as the positive control, while 4% DMSO in PBS was used as the negative control. The plates were sealed with a protective film to prevent evaporation and incubated for 1 hour at 37 °C in a VWR 1585 Incubator (no shaking). After incubation, the 96 well plates were centrifuged at 3900 rpm for 5 min. 50 µL of each well's supernatant was then carefully transferred to a new 96 well plate, which was then centrifuged to remove any bubbles, and the absorbance was measured at 414 nm. The percent hemolysis was calculated by the following formula:

$$\%\,Hemolysis = \frac{Abs\ (Sample) - Abs(Neg)}{Abs\ (Pos) - Abs\ (Neg)} \times 100$$

Where Abs(Sample) is the absorbance of each sample at 414 nm, Abs (Neg) is the average absorbance of the negative control at 414 nm, and Abs (Pos) is the average absorbance of the positive control at 414 nm. In most cases, 50% hemolysis was not reached even at the highest

concentrations tested. As these maximum concentrations are significantly higher (≥10-fold) than those used in transport or antibacterial (MIC) assays, the compounds can be considered non-hemolytic under the conditions studied.

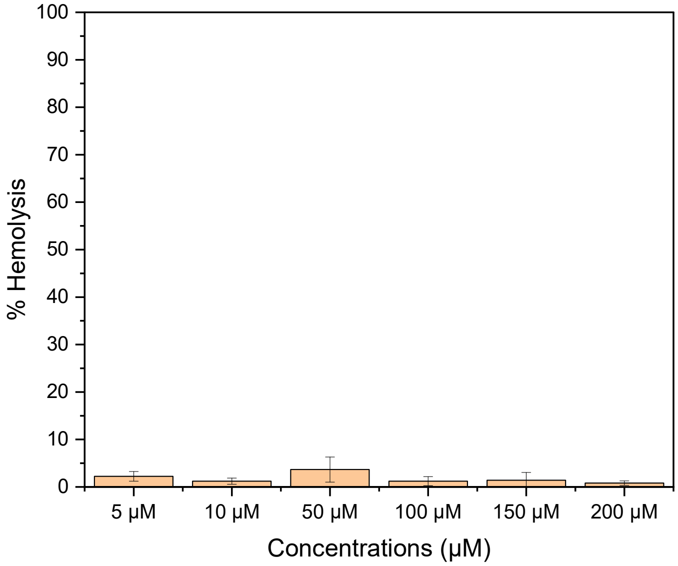

Figure S173. Normalized hemolytic activity of compound **1a** across varying concentrations. Data represents the average of at least two biological replicates, each performed with two technical repeats, and error bars indicate standard deviations. 50% hemolysis was not reached at the highest concentration tested (200 µM), which reflects the maximum soluble concentration under the assay conditions.

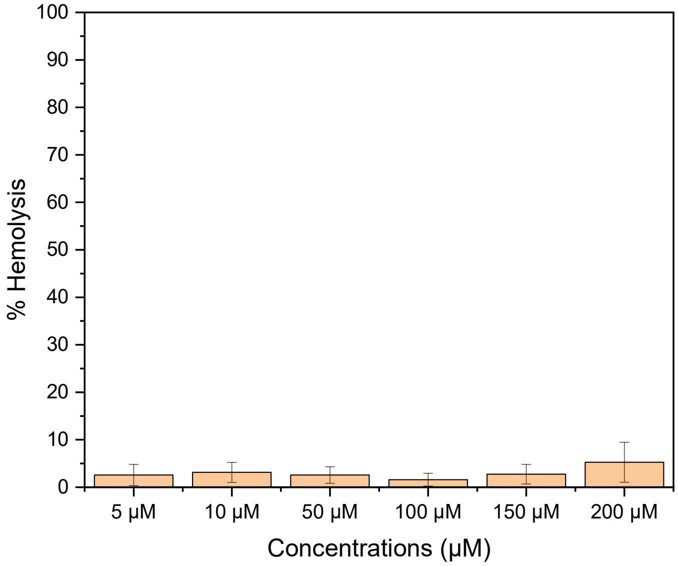

Figure S174. Normalized hemolytic activity of compound **1b** across varying concentrations. Data represents the average of at least two biological replicates, each performed with two technical repeats, and error bars indicate standard deviations. 50% hemolysis was not reached at the highest concentration tested (200 µM), which reflects the maximum soluble concentration under the assay conditions.

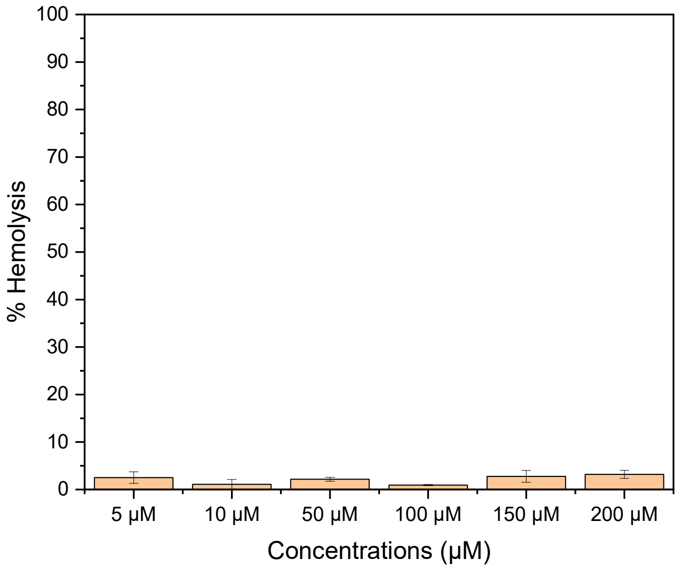

Figure S175. Normalized hemolytic activity of compound **1c** across varying concentrations. Data represents the average of at least two biological replicates, each performed with two technical repeats, and error bars indicate standard deviations. 50% hemolysis was not reached at the highest concentration tested (200 µM), which reflects the maximum soluble concentration under the assay conditions.

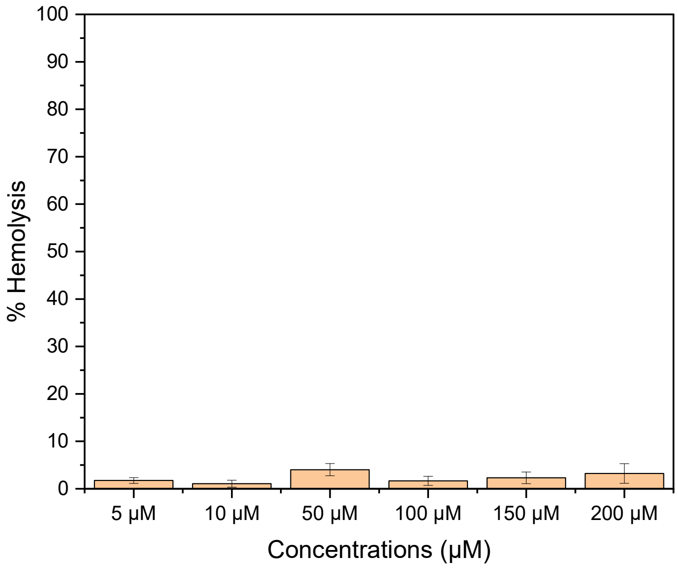

Figure S176. Normalized hemolytic activity of compound **1d** across varying concentrations. Data represents the average of at least two biological replicates, each performed with two technical repeats, and error bars indicate standard deviations. 50% hemolysis was not reached at the highest concentration tested (200 µM), which reflects the maximum soluble concentration under the assay conditions.

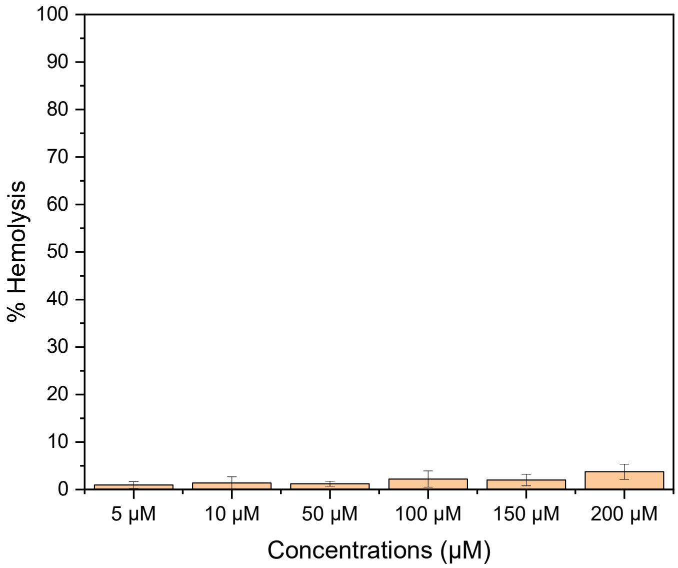

Figure S177. Normalized hemolytic activity of compound **1e** across varying concentrations. Data represents the average of at least two biological replicates, each performed with two technical repeats, and error bars indicate standard deviations. 50% hemolysis was not reached at the highest concentration tested (200 μM), which reflects the maximum soluble concentration under the assay conditions.

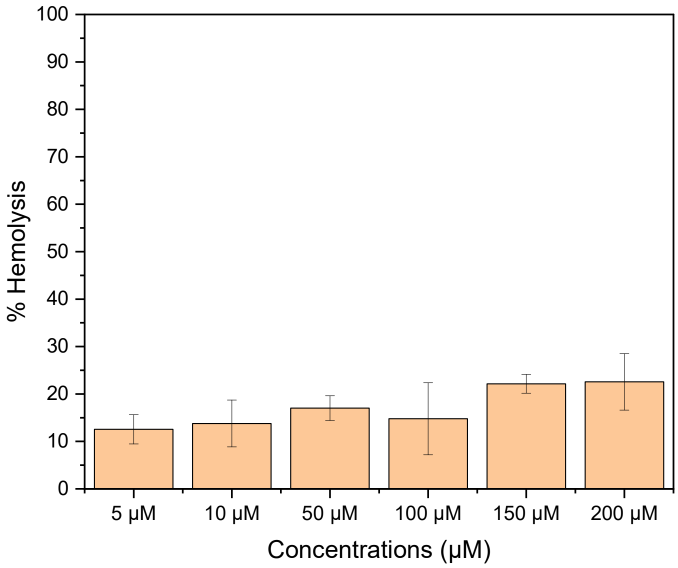

Figure S178. Normalized hemolytic activity of compound **1f** across varying concentrations. Data represents the average of at least two biological replicates, each performed with two technical repeats, and error bars indicate standard deviations. 50% hemolysis was not reached at the highest concentration tested (200 μM), which reflects the maximum soluble concentration under the assay conditions.

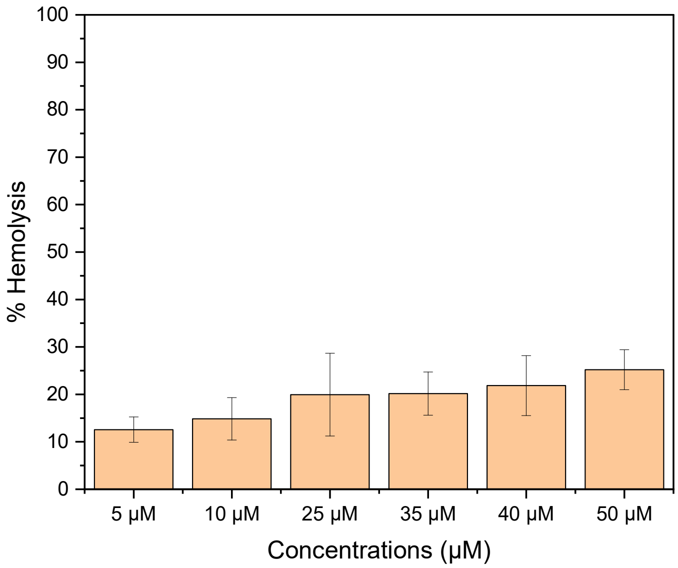

Figure S179. Normalized hemolytic activity of compound **2a** across varying concentrations. Data represents the average of at least two biological replicates, each performed with two technical repeats, and error bars indicate standard deviations. 50% hemolysis was not reached at the highest concentration tested (50 µM), which reflects the maximum soluble concentration under the assay conditions.

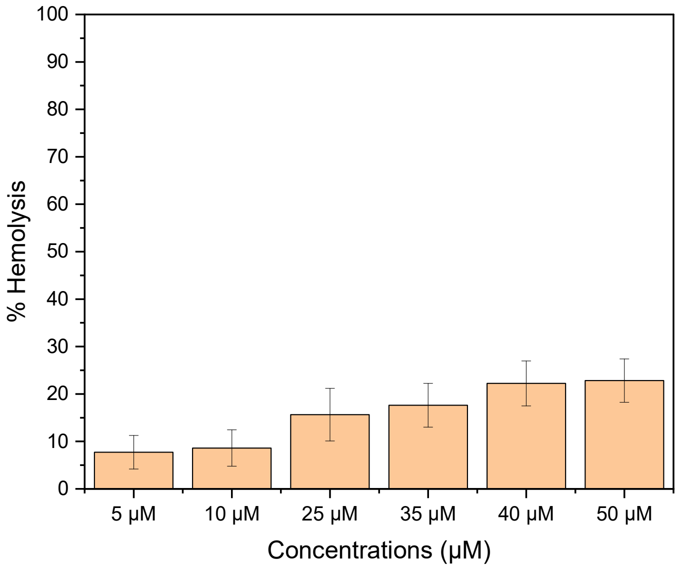

Figure S180. Normalized hemolytic activity of compound **2b** across varying concentrations. Data represents the average of at least two biological replicates, each performed with two technical repeats, and error bars indicate standard deviations. 50% hemolysis was not reached at the highest concentration tested (50 µM), which reflects the maximum soluble concentration under the assay conditions.

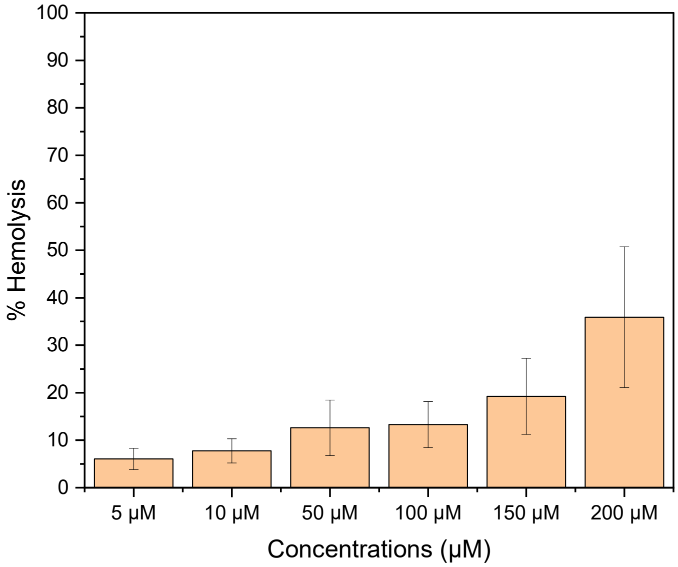

Figure S181. Normalized hemolytic activity of compound **2c** across varying concentrations. Data represents the average of at least two biological replicates, each performed with two technical repeats, and error bars indicate standard deviations. 50% hemolysis was not reached at the highest concentration tested (200 µM), which reflects the maximum soluble concentration under the assay conditions.

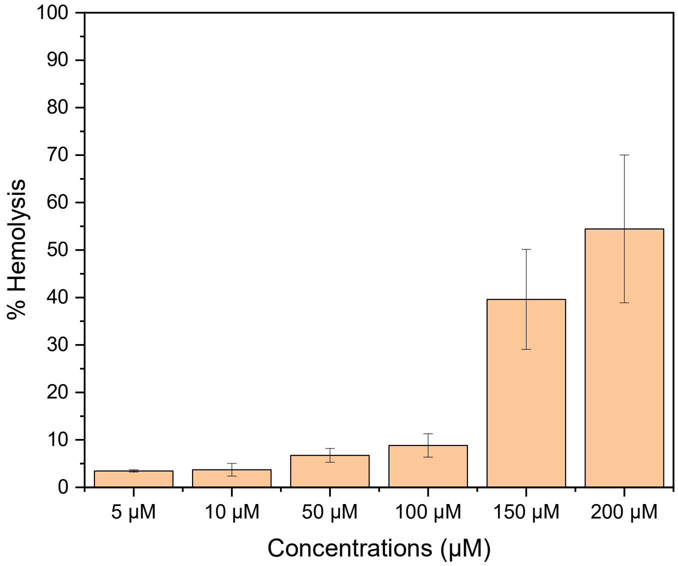

Figure S182. Normalized hemolytic activity of compound **2d** across varying concentrations. Data represents the average of at least two biological replicates, each performed with two technical repeats, and error bars indicate standard deviations. ~50% hemolysis was reached at 200 µM, which was the maximum soluble concentration under the assay conditions.

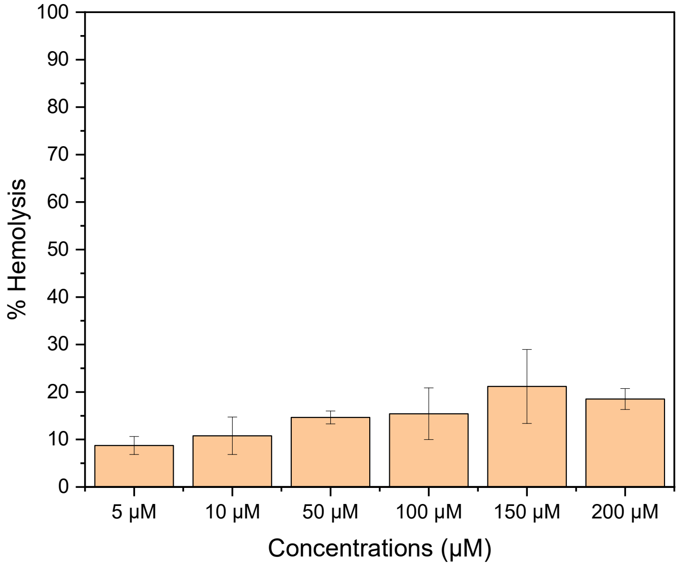

Figure S183. Normalized hemolytic activity of compound **2e** across varying concentrations. Data represents the average of at least two biological replicates, each performed with two technical repeats, and error bars indicate standard deviations. 50% hemolysis was not reached at the highest concentration tested (200 µM), which reflects the maximum soluble concentration under the assay conditions.

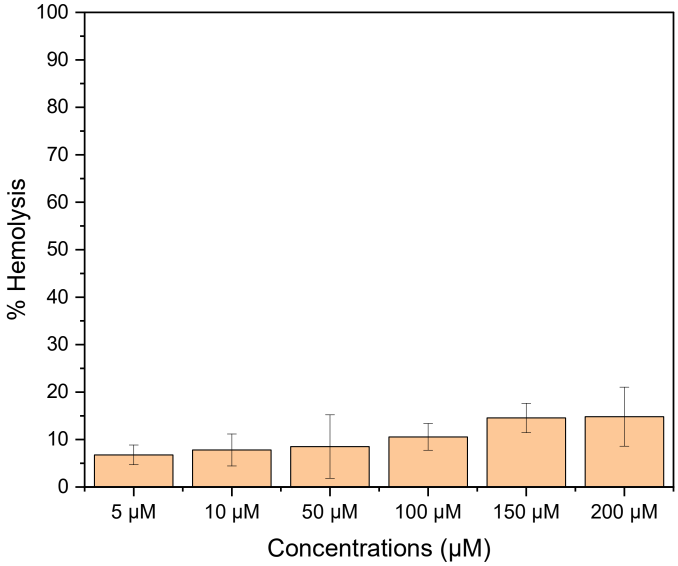

Figure S184. Normalized hemolytic activity of compound **2f** across varying concentrations. Data represents the average of at least two biological replicates, each performed with two technical repeats, and error bars indicate standard deviations. 50% hemolysis was not reached at the highest concentration tested (200 µM), which reflects the maximum soluble concentration under the assay conditions.

S12 References

1. E. R. Abdurakhmanova, D. Mondal, H. Jędrzejewska, P. Cmoch, O. Danylyuk, M. J. Chmielewski and A. Szumna, *Chem*, 2024, **10**, 1910-1924.
2. P. S. Dangate and K. G. Akamanchi, *Tetrahedron Letters*, 2012, **53**, 6765-6767.
3. Bruker, *Journal*.
4. L. Krause, R. Herbst-Irmer, G. M. Sheldrick and D. Stalke, *J. Appl. Crystallogr.*, 2015, **48**, 3-10.
5. G. Sheldrick, *Acta Crystallogr., Sect. A*, 2015, **71**, 3-8.
6. G. Sheldrick, *Acta Crystallographica Section C*, 2015, **71**, 3-8.
7. C. R. Groom, I. J. Bruno, M. P. Lightfoot and S. C. Ward, *Acta Crystallographica Section B*, 2016, **72**, 171-179.
8. M. Chvojka, A. Singh, A. Cataldo, A. Torres-Huerta, M. Konopka, V. Šindelář and H. Valkenier, *Analysis & Sensing*, 2024, **4**, e202300044.
9. N. Busschaert, I. L. Kirby, S. Young, S. J. Coles, P. N. Horton, M. E. Light and P. A. Gale, *Angew. Chem. Int. Ed.*, 2012, **51**, 4426-4430.
10. A. M. Gilchrist, P. Wang, I. Carreira-Barral, D. Alonso-Carrillo, X. Wu, R. Quesada and P. A. Gale, *Supramolecular Chemistry*, 2021, **33**, 325-344.
11. X. Wu, L. W. Judd, E. N. Howe, A. M. Withecombe, V. Soto-Cerrato, H. Li, N. Busschaert, H. Valkenier, R. Perez-Tomas and D. N. Sheppard, *Chem*, 2016, **1**, 127-146.
12. M. Chvojka, A. Singh, A. Cataldo, A. Torres-Huerta, M. Konopka, V. Šindelář and H. Valkenier, *Analysis & Sensing*, 2024, **4**, e202300044.
13. S. R. Herschede, H. Gneid, T. Dent, E. B. Jaeger, L. B. Lawson and N. Busschaert, *Org. Biomol. Chem.*, 2021, **19**, 3838-3843.
14. E. O. Ojah, H. Gneid, S. R. Herschede and N. Busschaert, *Chem. Eur. J.*, 2024, **30**, e202402698.
15. S. M. Hickey, T. D. Ashton, G. Boer, C. A. Bader, M. Thomas, A. G. Elliott, C. Schmuck, H. Y. Yu, J. Li and R. L. Nation, *European journal of medicinal chemistry*, 2018, **160**, 9-22.
16. P. Wayne, 2011.
17. J. D. Te Winkel, D. A. Gray, K. H. Seistrup, L. W. Hamoen and H. Strahl, *Frontiers in cell and developmental biology*, 2016, **4**, 29.
18. D. A. Kelkar and A. Chattopadhyay, *Biochimica et Biophysica Acta (BBA)-Biomembranes*, 2007, **1768**, 2011-2025.
19. B. L. Roth, M. Poot, S. T. Yue and P. J. Millard, *Applied and environmental microbiology*, 1997, **63**, 2421-2431.
20. Z. AlKhatib, M. Lagedroste, J. Zaschke, M. Wagner, A. Abts, I. Fey, D. Kleinschrodt and S. H. Smits, *Microbiologyopen*, 2014, **3**, 752-763.
21. K. M. Scherer, J.-H. Spille, H.-G. Sahl, F. Grein and U. Kubitscheck, *Biophysical journal*, 2015, **108**, 1114-1124.
22. P. F. Popp, A. Benjdia, H. Strahl, O. Berteau and T. Mascher, *Frontiers in microbiology*, 2020, **11**, 151.
23. L. E. Brennan, L. K. Kumawat, M. E. Piatek, A. J. Kinross, D. A. McNaughton, L. Marchetti, C. Geraghty, C. Wynne, H. Tong and O. N. Kavanagh, *Chem*, 2023, **9**, 3138-3158.
24. N. Molchanova, P. R. Hansen and H. Franzyk, *Molecules*, 2017, **22**, 1430.
